# Cooperative light-induced breathing of soft porous crystals via azobenzene buckling

**DOI:** 10.1038/s41467-022-29149-z

**Published:** 2022-04-12

**Authors:** Simon Krause, Jack D. Evans, Volodymyr Bon, Stefano Crespi, Wojciech Danowski, Wesley R. Browne, Sebastian Ehrling, Francesco Walenszus, Dirk Wallacher, Nico Grimm, Daniel M. Többens, Manfred S. Weiss, Stefan Kaskel, Ben L. Feringa

**Affiliations:** 1 Centre for Systems Chemistry, Stratingh Institute for Chemistry, University of Groningen, Nijenborgh 4, 9747 AG Groningen, The Netherlands; 2 Faculty of Chemistry and Food Chemistry, Technische Universität Dresden, Bergstrasse 66, 01062 Dresden, Germany; 3 Nanochemistry Department, Max-Planck-Institute for Solid State Research, Heisenbergstraße 1, 70569 Stuttgart, Germany; 4 Centre for Advanced Nanomaterials and Department of Chemistry, The University of Adelaide, North Terrace, Adelaide, South Australia 5000 Australia; 5 Helmholtz-Zentrum Berlin für Materialien und Energie, Hahn-Meitner-Platz 1, 14109 Berlin, Germany

**Keywords:** Metal-organic frameworks, Molecular dynamics

## Abstract

Although light is a prominent stimulus for smart materials, the application of photoswitches as light-responsive triggers for phase transitions of porous materials remains poorly explored. Here we incorporate an azobenzene photoswitch in the backbone of a metal-organic framework producing light-induced structural contraction of the porous network in parallel to gas adsorption. Light-stimulation enables non-invasive spatiotemporal control over the mechanical properties of the framework, which ultimately leads to pore contraction and subsequent guest release via negative gas adsorption. The complex mechanism of light-gated breathing is established by a series of in situ diffraction and spectroscopic experiments, supported by quantum mechanical and molecular dynamic simulations. Unexpectedly, this study identifies a novel light-induced deformation mechanism of constrained azobenzene photoswitches relevant to the future design of light-responsive materials.

## Abstract (web summary)

The application of photoswitches as light-responsive triggers for phase transitions of porous materials remains poorly explored. Here, the authors report a light-responsive flexible metal-organic framework which undergoes pore contraction upon combined application of light irradiation and adsorption stress via a buckling process of the framework-embedded azobenzene photoswitch.

## Introduction

The design of responsive porous materials, in which the porosity can be modulated externally and non-invasively by light to control adsorption, transport, and release properties offers fascinating opportunities. Azobenzene (AB) molecular photoswitches^1^ (PS) undergo light-activated *E-Z* isomerization and are frequently applied in light-responsive actuators^2^ membranes^3^, smart materials^4^, and single-molecule optical memories^5^. Pendent AB-switches grafted onto the backbone of porous metal-organic frameworks (MOFs) were demonstrated to reversibly control the separation and release of guest molecules by manipulating the porosity and host-guest interactions via photoswitching^6–8^. However, pendent AB switches occupy pore space, which could be used for guest inclusion, and lack cooperativity that would be highly beneficial for the efficiency and selectivity of adsorption processes^9,10^. Soft porous crystals^11^ (SPCs) exhibit cooperative framework deformation dictated by the crystal structure. As a result, SPCs show adsorption phenomena such as gate-opening (pore expansion)^12^, breathing (pore contraction)^13^, and negative gas adsorption (NGA, gas release upon pore contraction)^14^ that show potential for improved diffusion^15,16^, storage^17^ and separation^15^ of gases and gas mixtures. Currently the contraction and expansion of the porous network of SPCs are primarily guest-induced and energetically driven via adsorption^18^. To date, the chemical modification of building blocks and framework topology are the dominant strategy to alter the guest-responsive behavior of SPCs^19,20^. The use of diarylethene PS in the framework backbone is a promising strategy to manipulate cooperative framework transitions^21–24^. However, the observed effects are very small, compared to the response due to guest-induced deformations of SPCs, and the initiation of massive framework deformations in SPCs by the application of both light- and guest-interactions is unprecedented. The large geometric change upon *E-Z* isomerization of AB is expected to result in a much stronger framework deformation when incorporated in the framework backbone. Until now, photoswitching is observed to be either suppressed due to framework constraints^25,26^ or causes irreversible bond-breaking and degradation of the extended framework^27–30^. The fundamental challenges of how to accommodate the large geometric change of AB upon *E-Z* isomerization and establish photoinduced cooperative transitions, in the absence of framework disintegration, requires uncompromised/robust photoswitching, sufficient mechanical softness, enhanced porosity, and long-range order. Furthermore, it remains unexplored whether geometric constrains of framework-embedded PS might result in alternative photoswitching pathways, unknown for unconstrained molecular PS.

In this work we demonstrate the design and analysis of DUT-163, a MOF with framework-embedded azobenzene photoswitch. DUT-163 exhibits structural contraction by combined application of light irradiation and adsorption-stress via gas adsorption. Our work is based on a detailed theoretical analysis of the energy landscape of DUT-163 followed by in depth in situ experimental analysis using a range of spectroscopic and diffraction methods. From this data we derive that unexpectedly the contraction mechanism in DUT-163 is based on a buckling process of the ligand, previously unknown for molecular AB photo-switches. This mechanism is further supported by a series of computational simulations that detail the photochemistry and adsorption mechanism. Our analysis highlights the impact of framework-constraint on the behavior of framework-embedded photo-switches and postulates framework softening via light irradiation as the underlying mechanism responsible for framework transitions in DUT-163. Furthermore, we show that the light activation is applied locally allowing to use this process in light-responsive nanoscopic pneumatic systems and gas-releasing devices.

## Results and discussion

### Modeling of molecular photoswitch and framework

**Fig. 1:**
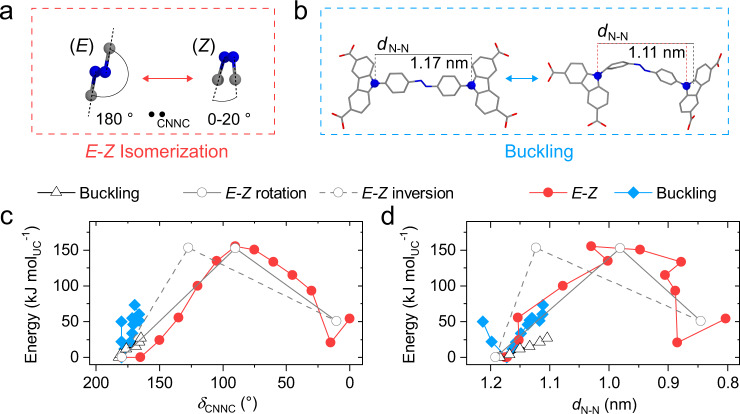
Single-molecule deformation. **a** Molecular mechanism of E-Z isomerization and **b** molecular mechanism of ligand buckling. **c**, **d** Single ligand energetics as a function of *δ*_CNNC_ (**c**) and *d*_N-N_ (**d**) for framework constrained dacdc (red, blue) and unconstrained Me_4_dacdc (gray open symbols. *E-Z* isomerization via rotation and inversion as well as buckling of single ligands is given as open circles with solid line, dashed line, and open triangles, respectively.

We selected the 49th MOF material discovered at the Dresden University of Technology (DUT-49)^31^ as a blueprint for our new photoresponsive SPC design because of its ability to accommodate large changes in ligand configuration and framework structure without disintegration following substantial framework contraction^14^. The three-dimensional (3D) framework of DUT-49 is based on the linkage of tetra-connective carbazole-based ligands to copper(II) dimers. By using (*E*)-9, 9’-(diazene-1, 2-diylbis(4, 1-phenylene))bis(9*H*-carbazole-3, 6-dicarboxylic acid ((*E*)-H_4_dacdc) we are able to establish the structurally related framework of DUT-163 which contains an AB functionality in the backbone. We conducted density functional theory (DFT) simulations of (*E*)-H_4_dacdc and its methylester ((*E*)-Me_4_dacdc), to probe the energetics upon buckling^32^ and *E-Z*-isomerization as a function of the distance between two AB-bridged carbazole-nitrogen atoms (*d*_N-N_) and the dihedral angle of the azo-unit (*δ*_CNNC_) (Fig. 1).

**Fig. 2:**
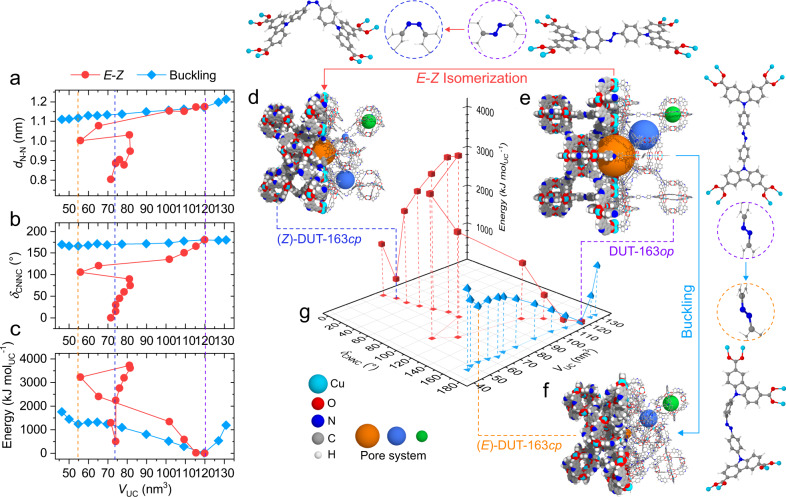
Framework energy landscape. **a**–**c** Framework contraction via *E-Z* isomerization (red squares) and ligand buckling (blue diamonds) as a function of *d*_N-N_ (**a**), *δ*_CNNC_ (**b**) and energy (**c**). Unit cell volumes of (*Z*)-DUT-163*cp* (blue), DUT-163*op* (purple), and (*E*)-DUT-163*cp* (orange) are given as dashed vertical lines. Framework contraction via *E-Z* isomerization and ligand buckling are given in red circles and blue diamonds, respectively. Framework structures of (*Z*)-DUT-163*cp* (**d**), DUT-163*op* (**e**), and (*E*)-DUT-163*cp* (**f**) including the corresponding ligand structures and diaza-conformation in dashed circles. **g** 3D energy landscape of DUT-163 as a function of unit cell volume (*V*_UC_), *δ*_CNNC_ with energy normalized to the *op* state.

Regardless of the *E-Z* isomerization mechanism chosen (i.e. rotation or inversion^33,34^), the energy barrier of *E-Z* isomerization at the ground state is over five times larger than the barrier of buckling (*E*)-Me_4_dacdc. This result is to be expected since buckling is a conformational change while *E-Z* isomerization involves the breaking of the azo π-bond in the ligand backbone. To investigate how the constraints imposed by the incorporation in a framework impact the energetics of *E*-*Z* isomerization and buckling, we computed the contraction mechanism of DUT-163 as a function of unit cell volume (*V*_UC_) for buckling and *E-Z* isomerization of the ligand by molecular dynamics (MD) simulations (Fig. 2).

Similar to the analysis of the unconstrained Me_4_dacdc ligand, *E-Z* isomerization of dacdc in DUT-163 exhibits a much larger energy barrier compared to the buckling transition (Fig. 2). However, the associated contraction mechanism of the DUT-163 framework exhibit two very different trajectories. The energy landscape of DUT-163 as a function of *V*_UC_ exhibits the global minimum at *V*_UC_ = 120 nm^3^ corresponding to the open pore (*op*) state (DUT-163*op*) (Fig. 2f). A metastable state with buckled ligand in *E* conformation at *V*_UC_ = 54 nm^3^ is observed which is assigned to a contracted pore (*cp*) state, further denoted as (*E*)-DUT-163*cp* (See supplementary videos 1 and 2). This state is very similar to DUT-161*cp* which contains a stilbene instead of an AB unit in the ligand backbone^35^.

To probe the framework energetics upon *E-Z* isomerization of dacdc, we investigated the evolution of *V*_UC_ and the framework geometry as a function of *φ*_CNNC_ (See supplementary video 3 and 4). Interestingly, this energy landscape presents a local minimum at *V*_UC_ = 74 nm^3^, which is assigned to a contracted framework with dacdc in *Z*-configuration, (*Z*)-DUT-163*cp*. The energy barrier for contraction (*E*^*‡*^_op-cp_) per unit cell (UC) between DUT-163*op* and (*E*)-DUT-163*cp* (E^*‡*^_op-(*E*)cp_ = 1250 kJ mol_UC_^−1^) is ca. three times smaller compared to the barrier between DUT-163*op* and (*Z*)-DUT-163*cp* (E^*‡*^_op-(*Z*)cp_ = 3900 kJ mol_UC_^−1^) (Fig. 2). Based on this data it can be concluded that DUT-163 is theoretically able to undergo contraction via buckling or *E-Z* isomerization, with buckling being the energetically more favorable mechanism at the ground state.

### Photoswitching of the molecular ligand

**Fig. 3:**
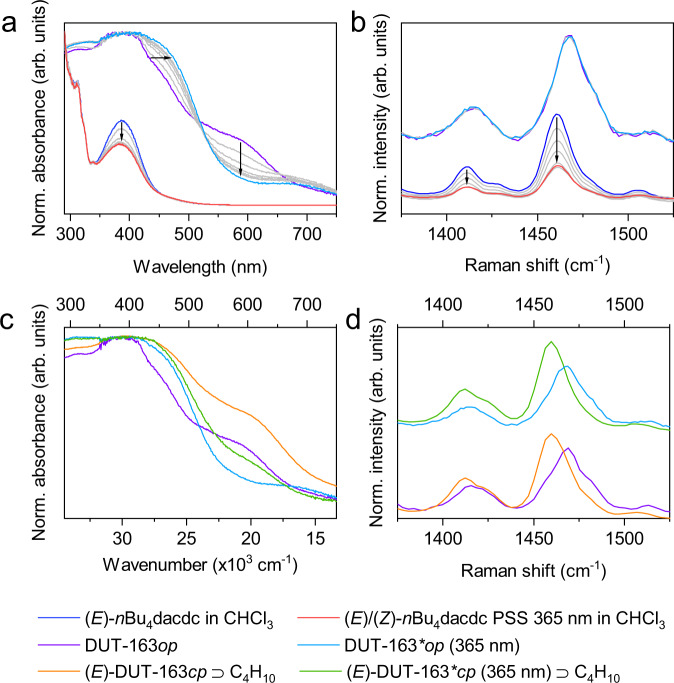
In situ irradiation of ligand and MOF. **a** UV-Vis absorption spectra upon 365 nm irradiation of *n*Bu_4_dacdc in CHCl_3_ (blue to red) and DRUV-Vis absorption spectra of DUT-163 (purple to light blue), **b** Raman spectra of *n*Bu_4_dacdc in CHCl_3_ (red to blue) and solid DUT-163 (purple to light blue) upon 365 nm irradiation, **c** DRUV-Vis absorption spectra of DUT-163 (purple) upon loading with *n*-butane (orange), 365 nm irradiation (light blue) and irradiation at 365 nm in the presence of 2-methylpropane (green), **d** and Raman spectra upon loading with 2-methylpropane and irradiation at 365 nm.

(*E*)-H_4_dacdc was synthesized using an established strategy (see Supplementary Information for details)^36^. Upon irradiation at 365 nm (295–298 K) we observed changes in the UV-Vis absorption, Raman (Fig. 3) and ^1^H nuclear magnetic resonance spectra (Supplementary Figs. 7–10) of (*E*)-H_4_dacdc and the corresponding *n*-butyl ester ((*E*)-*n*Bu_4_dacdc), typical for light-induced *E-Z* isomerization^33,37^.

For both molecules, we observed a photostationary state (PSS) composed of a ca. 1:1 *E-Z* mixture at 293 K. Upon irradiation at 455 nm the *Z*-isomer was partially reverted to a PSS comprising of ca. 25% of the *Z*-isomer. Thermal *Z*-*E* isomerization was observed by heating above 338 K for over 5 h and the system showed excellent photochemical and thermal reversibility in solution.

### Synthesis of DUT-163

The solvothermal reaction of H_4_dacdc with Cu(NO_3_)_2_·3H_2_O in DMF at 80 °C yields DUT-163 as a brown microcrystalline powder with a mean crystal size of 2.6 µm (Supplementary Fig. 28). The single-crystal structure of DUT-163 with cubic *Fm*$\bar{3}$*m* symmetry and cell dimensions of *a* = 49.240(6) Å and a unit cell volume of *V*_UC_ = 119386(41) Å^3^ was determined by synchrotron-based single-crystal X-ray diffraction (Supplementary Table 8), in line with the in silico optimized *op* structure. In DUT-163*op*, dacdc exhibits a linear (*E*)-configuration in which the AB-unit is disordered due to symmetrical restrictions (Supplementary Fig. 24). The porous framework is characterized by a geometrical surface area, pore volume and pore diameters of GSA = 5112 m^2^ g^−1^, *V*_p_(sim) = 3.2 cm^3^ g^−1^, and *d*_p_ = 0.9–2.7 nm, respectively which were simulated from the single crystal structure (Supplementary Fig. 97). Desolvation of DUT-163 was achieved using supercritical carbon dioxide, a protocol previously described for DUT-49^36^. Permanent porosity was investigated by N_2_-adsorption experiments at 77 K from which a *V*_p_ of 2.84 cm^3^ g^−1^ (at *p/p*_0_ = 0.98) was determined. The reduction in pore volume compared to the computed values might be based on crystal size effects previously observed for DUT-49^38^.

### Spectroscopic analysis of structural contraction

The light-responsiveness of DUT-163 was investigated by in situ PXRD, diffuse reflectance infrared Fourier transform spectroscopy (DRIFTS), solid state diffuse reflectance UV-Vis (DRUV-Vis) spectroscopy, and Raman spectroscopy experiments under dry nitrogen atmosphere with 365 nm irradiation at 293 K. These conditions were previously found to promote *E-Z* isomerization in solutions of both *n*Bu_4_dacdc and H_4_dacdc. Interestingly, we observed no significant changes in the Raman and DRIFT spectra as well as PXRD patterns of DUT-163 upon elongated 365 nm irradiation (Fig. 3), indicating the absence of *E-Z* isomerization of the ligand and structural contraction of the framework. This is further supported by nitrogen adsorption experiments at 77 K, which showed no change in porosity after 365 nm irradiation (Supplementary Fig. 29). However, upon 365 nm irradiation, we observed a bathochromic shift of the absorption in the DRUV-Vis spectrum of DUT-163 corresponding to the AB-functionality, and decrease of the signal assigned to the absorption of the Cu^2+^-dimer at 550–600 nm (Fig. 3a). The original spectra were not restored upon irradiation at 455 nm (Supplementary Fig. 84). Rather than *E-Z* isomerization which would cause pronounced changes in the PXRD patterns as well as Raman and DRIFT spectra, we propose a photoinduced charge transfer from the AB-functionality to the Cu^2+^ site, which is reported in other metal-AB-complexes^39^. The absence of changes upon irradiation in the DRUV-Vis spectrum of DUT-49 (Supplementary Fig. 80), which does not contain an oxidizable AB-unit, supports such mechanism. Furthermore, spin-flip DFT calculations on Cu_2_dacdc indicate that the HOMO is located on the AB backbone while the LUMO is localized on the Cu^2+^-dimer, which would support the feasibility of photoinduced charge transfer (Supplementary Fig. 104).

### Adsorption-induced structural contraction

Although undeformable by light, the framework of DUT-163 dynamically responds to gas adsorption. Methane (111, 115 K), *n*-butane (298, 303 K) and CCl_4_ (298 K) adsorption isotherms of DUT-163, in contrast to nitrogen (77 K), exhibit hysteresis and NGA steps (Supplementary Fig. 33), both evidence for adsorption-induced contraction well studied in DUT-49 and related frameworks^14,35,40,41^. Similar behavior is observed upon adsorption of 2-methylpropane (MP) in the temperature range of 262–297 K. indicating occurring contraction. However, at elevated temperatures of 299–307 K isotherms exhibit no sign of adsorption-induced contraction (Supplementary Figs. 34–42). This observation is in agreement with adsorption of methane at 120 K and matches previous reports of DUT-49, where adsorption-induced contraction was observed in a narrow range of temperature and pressure for a given gas with a very strong change in adsorption behavior in only a narrow temperature range^42^. In situ PXRD in parallel to adsorption of *n*-butane at 298 K (Supplementary Fig. 44), MP at 262 K (Supplementary Fig. 43) and methane at 115 K show the formation of a new set of reflections assigned to the *cp* phase emerging at a relative pressure of 0.14. For MP and methane adsorption reversible reopening is observed at a relative pressure beyond 0.6. The *op→cp* phase transition is accompanied by a strong reduction in diffraction intensity which is again increased at higher relative pressures upon reopening, in line with previous studies on related solids^14,40,41^. The loss in diffraction intensity can be assigned to enhanced disorder of the framework in the *cp* state and the presence of disordered gas present in the pores^43^. Reversible reopening and cycling after guest removal (Supplementary Fig. 32) demonstrate the integrity of framework connectivity upon framework contraction and expansion. The crystal structure of (*E*)-DUT-163*cp* could be determined by Rietveld refinement using PXRD data recorded at 262 K and *p*/*p*_0_ = 0.25 of MP (Supplementary Fig. 78). The experimental structure is in good agreement with the previously described simulation of (*E*)-DUT-163*cp* (Fig. 1) demonstrating the validity of the MD simulations of this system. To further analyze the adsorption behavior of DUT-163 we simulated MP and methane isotherms for (*E*)-DUT-163*cp* and DUT-163*op* at various temperatures by grand canonical Monte Carlo (GCMC) methods(Supplementary Fig. 98). This approach allows the characterization of thermodynamic conditions for adsorption-induced contraction upon MP adsorption using the grand canonical ensemble^42^. Interestingly, adsorption-induced contraction becomes thermodynamically unfeasible at increasing temperature (Supplementary Fig. 99) due to decreasing adsorption interactions. This explains the experimental upper temperature limit around 299 K and 120 K for contraction upon adsorption of MP and methane in DUT-163, respectively.

### Adsorption- and photo-induced structural contraction

**Fig. 4:**
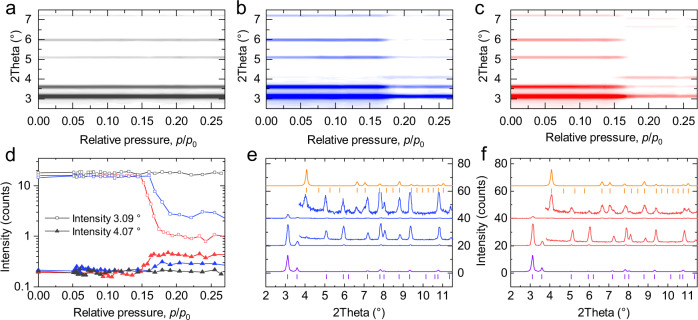
In situ diffraction analysis. **a**–**c** Contour plots of in situ PXRD data recorded in parallel to adsorption of MP at 300 K (gray) (**a**), at 300 K with 365 nm irradiation (blue) (**b**) and at 296 K (red) (**c**) and corresponding evolution of peak intensity of the (111) reflection of DUT-163*op* (open squares) and (*E*)-DUT-163*cp* (filled triangles) at 3.1° and 4.02°, respectively (**d**). **e**, **f** Selected PXRD patterns and magnified wide angle region as inset before (middle bottom) and after (middle top) structural contraction at 299 K upon irradiation with 365 nm (**e**) and at 295 K without irradiation (**f**) in comparison to simulated PXRD patterns and peak positions for DUT-163*op* (purple) and (*E*)-DUT-163*cp* (orange).

Although light-driven contraction via *E-Z* isomerization was not observed in guest-free DUT-163, we reasoned that additional adsorption interactions might help to stabilize a contracted (*Z*)-DUT-163*cp* state and trigger a structural response by parallel application of light and gas adsorption. In initial experiments we recorded MP physisorption isotherms of two individual samples of DUT-163 in the temperature range of 307-295 K for which one sample was irradiated at 365 nm throughout the whole experiment, while the other was kept under light exclusion. Still, no differences between the adsorption isotherms of irradiated and non-irradiated DUT-163 samples could be detected, neither in the temperature range above nor below 299 K (Fig. 4a–c and Supplementary Figs. 34–42) indicating the absence of light-induced contraction of the porous material in this experimental setup.

However, the large sample amount (>10 mg) required for accurate gas adsorption experiments causes light scattering and absorption leading to inhomogeneous illumination of the entire bulk solid. Consequently, adsorption experiments only provide little information on structural transitions that potentially occur only for a small part of the bulk sample.

In order to explore in more detail, the light-responsive structural behavior of DUT-163 in parallel to adsorption we designed in situ experimental setups that allow exposing small sample amounts (<0.2 mg) to defined gas pressure while irradiating with light of defined wavelengths under isothermal conditions and tracking structural transitions by synchrotron-based PXRD. Initial experiments used a setup designed for cryogenic temperatures with a flat sample bed in reflection geometry encapsulated in an insulated sample cell connected to glass fiber and gas capillary for irradiation and gas dosing, respectively. This setup was used to analyze methane adsorption at 120 K with and without UV irradiation. In fact, a partial contraction was observed which reversibly transformed back to the pristine *op* state. Yet, a more detailed study proved difficult due to insufficient light intensity and penetration depth of the flat sample bed (Supplementary Fig. 45). Consequently, a second setup in which sample filled capillaries are directly exposed to UV-light in parallel to the adsorption process proved more suitable and reliable (Supplementary Fig. 51). In a series of experiments, we probed the adsorption-induced structural transition upon MP adsorption at 296 K and 300 K (Fig. 4f).

At 296 K and relative pressure of 0.15–0.16, we observed an *op→cp* transition (Fig. 4f), demonstrating the ability to generate, observe, and identify the nature of adsorption-induced structural changes in DUT-163 with this setup. In a second experiment we raised the temperature to 300 K, beyond the upper temperature limit for adsorption-induced contraction. As expected, no structural contraction is observed (Fig. 4a) in line with the gas adsorption experiments at 299 K (Supplementary Fig. 38). In a third experiment, we used the same conditions (300 K) on the same sample, but this time irradiated the sample, throughout the whole adsorption process, with 365 nm light. Interestingly, at a relative pressure of 0.17–0.18, we observed a strong decrease in diffraction intensity at 2*θ* = 3.09 ° and appearance of new peaks at 2*θ* = 4.07° and 6.66° (Fig. 4e, f), which we can assign to the formation of (*E*)-DUT-163*cp*. Reversible reopening of the structure was not observed in the investigated pressure range but is expected to occur at increasing relative pressures similar to the experiment conducted at 262 K without and methane at 120 K with irradiation (Supplementary Fig. 49). Repetition of the experiments at 300 K on three individual samples confirmed the initial observations and the light-responsive behavior (Supplementary Figs. 57–61). In all experiments, the temperature recorded in close proximity to the sample was stable at 300 K, with fluctuations below ±0.2 K. We observed no change in the diffraction patterns of (*E*)-DUT-163*cp* upon irradiation with 365 nm and 455 nm light (Supplementary Fig. 54), reflecting the absence of light-induced *op→cp* transition by potential *Z-E* photoisomerization. In one experiment, 365 nm irradiation was applied only in the relative pressure range of 0.16–0.28, 1 min before the *op-cp* transition occurred, demonstrating that prolonged irradiation is not essential and the light application allows for temporal control of the process.

### Spatial control over light-induced contraction

**Fig. 5:**
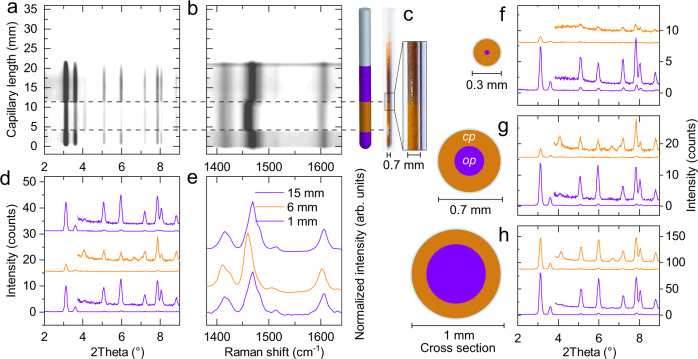
Analysis of local photo-induced transition. **a**–**e** Axial PXRD (**a**) and Raman scan (**b**) of a capillary with a diameter of 0.7 mm, image and illustration of the investigated capillary (**c**), selected PXRD patterns at 1 mm (bottom), 6 mm (middle), and 13 mm (top) (**d**), Raman spectra at selected positions (**e**), **f**–**h** PXRD patterns of non-irradiated and irradiated areas for capillaries with diameters of 0.3 mm (**f**), 0.7 mm (**g**), and 1 mm (**h**). Irradiated regions are marked in orange, non-irradiated areas in purple, inset enlarged patterns in **f**–**h** of 2Theta range 3.8-9° represent 20 times magnification.

Because only a 6-mm length section of the sample-filled capillary was irradiated in the experiments described above, we performed an axial PXRD scan along the capillary to determine the spatial phase composition (Fig. 5).

Only DUT-163 powder in the irradiated area exhibited structural contraction, supporting that light is indeed the trigger for the transition and demonstrating the spatial applicability of light-initiated contraction in DUT-163 (Fig. 5a, d). However, in all irradiation experiments, the residual *op* phase detected by PXRD indicates that only part of the sample undergoes a contraction. As the dense packing and high absorptivity of DUT-163 in the range of 200-600 nm can filter the light stimulus, we tested the light penetration depth by analyzing DUT-163-filled quartz capillaries with diameters of 0.3 mm, 0.7 mm, and 1 mm (wall thickness 0.01 mm) (Fig. 5f–h). We observed that an estimation of 88% (0.3 mm), 79% (0.7 mm), and 26% (1 mm) of the bulk sample in the detected area underwent contraction, considering the change in intensity of the (111) reflection of DUT-163*op* at 2*θ* = 3.09°. Thus, we evaluated that the penetration depth of the applied Light-emitting diode (LED) light is in the range of 0.1–0.15 mm for a non-compressed sample bed of DUT-163 powder. Although a more powerful light source might initiate contraction in a denser or thicker sample bed, the applied low power 15 mW LED used for irradiation in these experiments is sufficient to trigger structural contraction in microscopic or nanoscopic single crystals or thin films.

### In situ adsorption-light spectroscopic experiments

Although the presence of diffraction peaks of (*E*)-DUT-163*cp* clearly shows that the MP adsorption-induced structural transition at low temperature (262 K, 296 K) is comparable to the light-gated transition at higher temperature (300 K), the strong loss in crystallinity upon contraction might conceal the formation of states such as (*Z*)-DUT-163*cp*. We thus complemented PXRD experiments by DRUV-Vis and Raman spectroscopy in parallel to adsorption of MP at 300 K with and without irradiation (Fig. 3) complemented by DRIFT spectroscopy upon adsorption of *n*-butane and CCl_4_ at 295 K (Supplementary Figs. 87–92). In contrast to irradiation of guest-free DUT-163, adsorption-induced contraction strongly altered the spectra of DUT-163 (Supplementary Fig. 82) similar to experiments carried out on DUT-49 (Supplementary Fig. 79). DUT-163 samples exhibited similar contraction behavior with and without 365 nm irradiation in the presence of MP (Supplementary Fig. 84), supporting the crystallographic findings: DUT-163*op* transforms into (*E*)-DUT-163*cp* via ligand buckling in response to adsorption-induced structural contraction, that can be promoted by 365 nm irradiation. Notably, this occurs at higher adsorption temperature at which adsorption stress alone is insufficient to trigger a contraction. Light-induced *E-Z* isomerization of dacdc and the formation of (*Z*)-DUT-163*cp* based on the combination of diffraction and spectroscopic experimental observations is excluded as the underlying mechanism for the light-responsive framework contraction.

### Modeling of photoexcited state

**Fig. 6:**
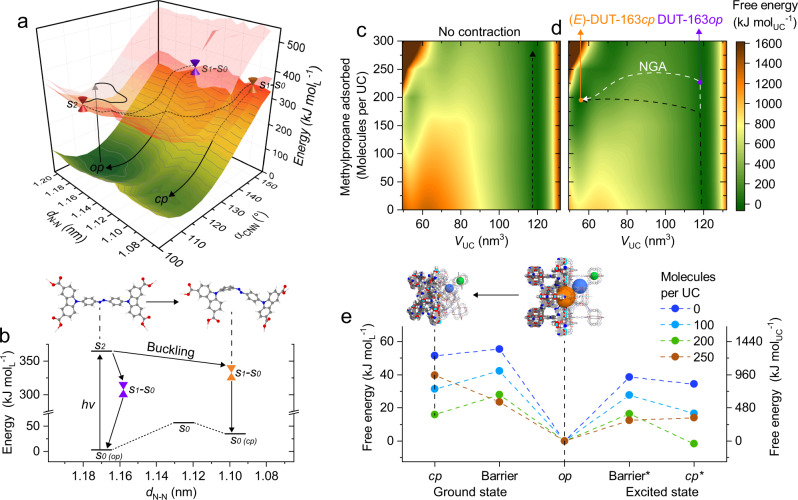
Energy landscape of dacdc and DUT-163. **a** Energy landscape in 3 dimensions (**a**) and a 2D projection (**b**) of ground state S_0_ and excited state S_1_ and S_2_ of H_4_dacdc as a function of *d*_N-N_. Crossing points and potential energy trajectories are marked as cones and dashed lines, respectively. Ligand conformations are given for the *op* and *cp* state. **c**–**e** Energy landscape at different loadings of MP at 300 K as contour plots of DUT-163 (**c**) and DUT-163* (**d**) as a function of unit cell volume normalized to the energy of the op state; **e** 2D energy diagram for selected loadings of the *op* and *cp* state for DUT-163 and DUT-163* including transition barriers. Trajectories in D for contraction without and with NGA transition is indicated as black and white lines, respectively.

To postulate a mechanism of how irradiation can promote contraction via buckling we computed the photoexcitation process of framework-constrained dacdc. Ligand geometries upon buckling were extracted from the MD simulations of the DUT-163 contraction (Fig. 2f). The energy landscapes of the ground state S_0_ and excited states S_1_ and S_2_ for H_4_dacdc were determined by TD-DFT calculations as a function of *d*_N-N_ distances and *α*_CNN_ angles^44^ (Fig. 6).

Interestingly, the energy landscape of framework-constrained dacdc exhibits a trajectory that promotes the formation of a metastable buckled state in *E*-conformation with reduced *d*_N-N_ and φ_CNNC_ ≈ 175° (Fig. 5a) which represents a novel photochemical deformation so far unexplored in AB. This channel resembles the one promoting the nonproductive deactivation of the AB excited state, after population of S_2_^33,45^. Although this analysis shows that both buckling or *E*-*Z* isomerization provide potential mechanism for light-induced contraction, the majority of individual ligands in the lattice of a single crystal would need to be instantaneously excited and transformed to drive cooperative structural contraction by light. The potential lack of collective excitation, supposedly short lifetimes of the excited states of individual ligands, and undersaturation of the bulk sample by the applied light source thus prevent observable photo-initiated contraction of guest-free DUT-163. However, upon adsorption of MP the energy landscape of DUT-163 changes. MD simulations of DUT-163 at 300 K at different loadings of MP per UC show that the energy difference between DUT-163*op* and (*E*)-DUT-163*cp* as well as the contraction barrier is reduced with increasing loading of MP. This observation is in line with previous analysis of the methane adsorption process in DUT-49^46^. The contraction barrier is decreased by 50% at a loading of 200 molecules of MP per UC, at which the pores of (*E*)-DUT-163*cp*⊃200*i*-C_4_H_10_ are saturated. Following increasing loading, (*E*)-DUT-163*cp* is severely destabilized due to the lower *V*_p_ that cannot accommodate enhanced amounts of MP. Although (*E*)-DUT-163*cp*⊃200*i*-C_4_H_10_ still represents a metastable state at the highest level of adsorption-induced stabilization, the reduction in contraction-barrier can make this state more prone to light-induced contraction.

To probe the response of DUT-163 upon irradiation we modeled an excited state of DUT-163 (DUT-163*) using established classical potentials that resemble the mechanics of dacdc in a biradical or zwitterionic state (further denoted to as dacdc*). This state is also a good mechanical representation of the previously described photooxidized state upon charge transfer between AB and Cu^2+^. We investigated the free energy landscape of guest-free DUT-163* upon loading with MP using the same MD method applied in the analysis of the DUT-163 ground state. Interestingly, guest-free DUT-163* is found to exhibit a much lower barrier for contraction compared to DUT-163. The breakage of π-conjugation in the ligand backbone of DUT-163* is the origin for this softening, which is also found to occur in chemically modified DUT-49-type frameworks^35^. It is well reflected by the simulated bulk modulus of 4.8 GPa for guest-free DUT-163 and 4.1 GPa for guest-free DUT-163*, respectively. Because DUT-163* is mechanically softer compared to DUT-163 adsorption stress produces a greater change in volume. In fact, at a loading of 200 molecules MP per UC, (*E*)-DUT-163*cp⊃200*i*-C_4_H_10_ was found to be the thermodynamically stable state with a reduction in contraction barrier of 42% compared to DUT-163⊃200*i*-C_4_H_10_ under the same conditions. Although in this model of DUT-163* all ligands are simultaneously in the excited state, which might not occur in a real crystal, even partial photoexcitation or -oxidation is expected to soften the framework of DUT-163 significantly. The nature of the mechanism triggering the softening of the structure can hence be hypothesized to be buckling of the chromophore either via an excited state pathway, as described in Fig. 6a, a photooxidation of the azo group by the Cu^2+^ clusters (Supplementary Fig. 104), or a combination of both. Additional adsorption interactions lower the barrier for contraction and initiating a light/adsorption-induced cooperative contraction of the crystal. To further analyze the photooxidation and charge transfer mechanism we propose characterization of thin films or single crystals of DUT-163 by methods such as X-ray photoelectron, X-ray absorption near edge structure, or electron paramagnetic resonance spectroscopy.

This dual-stimulus approach provides several advantages over purely light- or adsorption-induced transitions: photoexcitation allows the framework to respond to lower adsorption-induced stress levels and even drive contraction of a metastable state beyond the upper temperature limit of adsorption-induced contraction. The observed contraction results in gas release by NGA in an extended temperature range with a potentially increased magnitude. In addition, it allows to trigger NGA by a physical stimulus that specifically interacts with the framework. This photostimulation can be applied orthogonally to other non-radiative processes and other chemical or physical stimuli. Finally, it provides the possibility to spatially and temporarily control the release of gas via NGA using light as a physical trigger.

In conclusion, we show a cooperative structural transition of a SPC by combined application of light and adsorption-stress. Although DUT-163 was initially designed for contraction via *E-Z* isomerization of the AB-backbone, this process was ruled out by a combination of in situ experiments and extensive computation. Instead, the contraction mechanism is based on a buckling process, previously unknown for molecular ABs, and highlights the impact of framework-constraint on the behavior of photo-switches. In DUT-163 photoexcitation causes framework softening, allowing to drive structural contraction at reduced adsorption stress levels. The effect is reproducible under different conditions and allows for spatial and temporal control over the framework contraction by light. As such, light-responsive gas release by NGA can be locally and temporarily activated in DUT-163 for the use in nanoscopic pneumatic systems and gas-releasing devices^47^. The postulated mechanism not only demonstrates a novel switching transition in AB and an unexplored way of initiating structural transitions in SPCs, it provides a novel strategy to physically alter the mechanical properties of extended molecular frameworks without the application of chemical functionalization, potentially allowing such frameworks to respond to other forms of stimuli such as electric or magnetic fields, temperature, or mechanical pressure which would result in a novel class of mechanical nanoscopic actuators. Furthermore, we believe the findings of this study go beyond the discovery of a novel mechanism of a light-induced cooperative transition in a SPC. Over the past years, many AB-doped materials were shown to exhibit light-induced changes of their properties upon irradiation^23,48^. In the vast majority of cases *E-Z*-photoisomerization was postulated as the primary origin for the observed behavior. The present study clearly illustrates that framework- or matrix-constrained photoswitches can exhibit properties and states very different to the unrestricted single molecular analog. We conclude that photochemical properties of self-assembled systems are also governed by the structure and nature of the assembly beyond the properties of the molecular building blocks. In-depth analysis of these effects will lead to new design principles and novel properties of smart materials which may give rise to unexpected responsive behavior.

## Methods

### Chemicals

For the synthesis and characterization the following commercial chemicals were used: 4-Bromoaniline (CAS: 106-40-1, 97%, Sigma Aldrich), Cu(NO_3_)_2_·3H_2_O (CAS: 10031-43-3, 98%, Sigma Aldrich), *N,N*-Dimethylethylenediamine (CAS: 108-00-9, 95%, Sigma Aldrich), 9*H*-Carbazole (CAS: 86-74-8, >95%, Sigma Aldrich), Copper(I) iodine (CAS: 7681-65-4 99%, Riedel-de Haen), N,N′-Dimethylformamid, (CAS: 68-12-2, 99%, Fischer Scientific). Solvents and stock chemicals were used with purities exceeding 98%.

### Solution/liquid-state NMR

Nuclear magnetic resonance (NMR) spectra were acquired on a *Bruker* AV III *600* spectrometer (600.16 MHz and 150.91 MHz for ^1^H and ^13^C, respectively)). All ^1^H and ^13^C NMR spectra are reported in parts per million (ppm) downfield of TMS and were measured relative to the residual signals of the solvents at 7.26 ppm (CHCl_3_) or 2.54 ppm (DMSO). Data for ^1^H NMR spectra are described as following: chemical shift (*δ* (ppm)), multiplicity (s, singlet; d, doublet; t, triplet; q, quartet; m, multiplet; br, broad signal), coupling constant *J* (Hz), integration corresponding to amount of C or CH. Data for ^13^C NMR spectra are described in terms of chemical shift (*δ* (ppm)) and functionality were derived from DEPT spectra.

### Mass spectrometry

Matrix-assisted laser desorption/ionization (MALDI) time of flight (TOF) mass spectrometry analysis was performed on a BRUKER Autoflex Speed MALDI TOF MS using dithranol as matrix.

### Elemental analysis

Elemental analysis was carried out on a VARIO MICRO-cube Elemental Analyzer by *Elementar Analysatorsysteme GmbH* in CHNS modus. The composition was determined as the average of three individual measurements on three individually prepared samples.

### DRIFT spectroscopy

Diffuse reflectance infrared Fourier transform (DRIFT) spectroscopy was performed on a BRUKER VERTEX 70 with a SPECAC Golden Gate DRIFT setup. Prior to the measurement 2 mg of sample were mixed with 10-15 mg dry KBr in a mortar and pressed in the DRIFT-cell. Assignments of peaks in wavenumber ν (cm^-1^) were categorized by strong (s), medium (m), weak (w).

### DRUV-Vis adsorption spectroscopy

Diffuse Reflectance Solid state UV-Vis (DRUV-Vis) spectra were recorded on a VARIAN CARY 4000. 2 mg of sample were mixed with 20′35 mg dry BaSO4 and pressed in the sample cell. To analyze MOF samples under inert atmosphere and in situ under various concentrations of *n*-butane, a HARRICK Praying Mantis reaction chamber was equipped with a dome containing UV-Vis-transparent quartz windows.

### Thermogravimetric analysis

Thermal analysis (TGA) was carried out in synthetic dry air using a NETZSCH STA 409 thermal analyser at a heating rate of 5 K min^−1^. Air sensitive MOF samples were prepared in an Ar-filled glovebox and inserted in the instrument with little exposure to ambient conditions.

### Powder X-ray diffraction

Powder X-ray diffraction (PXRD) patterns were collected in transmission geometry with a STOE STADI P diffractometer operated at 40 kV and 30 mA with monochromatic Cu-Kα_1_ (*λ* = 0.15405 nm) radiation, a scan speed of 30–15 s/step and a detector step size of 2*Ѳ* = 0.1–2°. The samples were placed between non-diffracting adhesive tape or in a glass capillary. “As made” samples were analysed while suspended in DMF. Desolvated samples were prepared under inert atmosphere in an Ar-filled glovebox. Theoretical PXRD patterns were calculated on the basis of crystal structures using Mercury 4.0 software package.

### SEM analysis of crystal size and morphology

Scanning electron microscopy (SEM) images of DUT-163 were taken with secondary electrons in a HITACHI SU8020 microscope using 1.0 kV acceleration voltage and 10.8 mm working distance. The powdered samples were prepared on a sticky carbon sample holder. To avoid degradation upon exposure to air, the samples were prepared under argon atmosphere. For each sample a series of images was recorded at different magnifications and for each sample three different spots on the sample holder were investigated. The crystal size refers to the edge length of the cubic crystals as they are the easiest to measure. The analysis of the SEM images was performed with ImageJ Software package^49^. Values for mean crystal size, as well as relative standard deviation (RSD) were obtained by using the ImageJ Analyse-Distribution function.

### Gas adsorption experiments

Low pressure (*p* < 110 kPa) volumetric adsorption experiments were carried out on a BELSORP-max instrument by MicrotracBEL Corp. The commercially available glass sample cells and the measuring routine of BELSORP-max control software was used. In general, equilibration conditions for each point were 1% pressure change within at least 350 s (exceptions are mentioned specifically). For adsorption experiments below the standard boiling point (CCl_4_ at 298 K) of the adsorptive 1% within 500 s was chosen. The dead volume was routinely determined using helium. Prior to the measurement powdered samples were transferred in the measuring cell in an Ar -filled glovebox and degassed at 353 K for at least 5 h in dynamic vacuum (*p* < 10^−4^ kPa). For reaching and stabilizing the desired adsorption temperatures different methods for were used: For analysis at 307–293 K a Julabo thermostat was used, liquid nitrogen was used for adsorption experiments at 77.4 K (N_2_-adsorption). In all cases the sample was placed in a BELSORP glass cell with a glass rod to reduce the dead volume. To reach adsorption temperatures of 216 K for methylpropane (MP) adsorption and 111 K for methane adsorption a closed cycle helium cryostat ARS DE-202AG was used. The desired temperatures were reached using a temperature controller LS-336 (Lake Shore) and the heat produced by the cryostat is removed from the system by a water-cooled helium compressor ARS-2HW. The sample was placed in a 3 cm long rod-shaped copper cell of 1 cm diameter, sealed from the exterior with a copper dome and insulated by dynamic vacuum (*p* < 10^−4^ kPa), and connected to the BELSORP-max adsorption instrument with a 0.5 mm copper capillary. The samples were degassed prior to the measurement at room temperature for at least 1 h in dynamic vacuum (*p* < 10^−7^ kPa).

### Light-emitting diodes for irradiation

For irradiation studies LEDs from THORLABS (M365FP1 Fiber-Coupled LED with 365 nm Nominal wavelength and M455F3 Fiber-Coupled LED with 455 nm Nominal wavelength) were used. The LEDs were controlled by a THORLABS T-Cube™ LED Driver with maximum current of 1.2 A and modulation mode of 0–5 kHz. The 365 nm LED was driven at 1.2 A, the 455 nm LED was driven at 1 A. For irradiation in parallel to UV-Vis (solid and solution), IR, and Raman spectroscopy and in situ PXRD the LEDs were mounted in close proximity to the sample. For in situ NMR studies a Ø400 µm fiber optic was used in a setup that was previously described in more detail^50^. Irradiation of the sample during gas adsorption with 365 nm was conducted with a CONSORT UV-lamp with 1800 µW/cm².

### Raman spectroscopy

Raman spectra in solution were recorded on a home-built system comprising of a sample holder with magnetic stirrer, 785 nm 400 mW laser (Cobolt, 08-NLDM) guided through a Raman probe and connected to a spectrograph (Andor^TM^ Technology, Kymera 193i) equipped with CCD camera (Andor^TM^ Technology, iDus-416). Solid samples were packed in Quartz capillaries and sealed under dry nitrogen atmosphere in a glovebox. Raman spectra of MOFs were recorded using a fiber coupled Raman microscope equipped with a 785 nm (50 mW) or 633 nm (300 mW) laser.

## Supplementary information

Supplementary Information

Description of Additional Supplementary Files

Supplementary Movie 1

Supplementary Movie 2

Supplementary Movie 3

Supplementary Movie 4

## Data Availability

Crystal structure of DUT-163*op* and (*E*)-DUT-163-cp are available at the CCDC database under CCDC-2040810 and CCDC-2040811, respectively. Additional experimental studies to support the findings of this manuscript are displayed in the supplementary information or attached as additional supplementary information files. Raw data is either provided in the supplementary information can be obtained upon request from the corresponding authors.
