# Supplementary material for: Cooperative light-induced breathing of soft porous crystals via azobenzene buckling
Source: Nat Commun. 2022 Apr 12;13:1951. doi: 10.1038/s41467-022-29149-z (PMC9005654; doi:10.1038/s41467-022-29149-z)
Supplement: Supplementary file 1 — Supplementary Information [file 41467_2022_29149_MOESM1_ESM.pdf]

Supporting Information for

Cooperative Light-Induced Breathing of Soft Porous Crystals via Azobenzene Buckling

Simon Krause[1,2]*, Jack D. Evans[2], Volodymyr Bon[2], Stefano Crespi[1], Wojciech Danowski[1], Wesley R. Browne[1], Sebastian Ehrling[2], Francesco Walenszus[2], Dirk Wallacher[3], Nico Grimm[3], Daniel M. Többens[3], Manfred S. Weiss[3], Stefan Kaskel[2]*, Ben L. Feringa[1]*

1) Centre for Systems Chemistry, Stratingh Institute for Chemistry, University of Groningen, Nijenborgh 4, 9747 AG Groningen, The Netherlands
2) Faculty of Chemistry and Food Chemistry, TU Dresden, Bergstrasse 66, 01069, Dresden, Germany
3) Helmholtz-Zentrum Berlin für Materialien und Energie, Hahn-Meitner-Platz 1, 14109, Berlin, Germany

Correspondance to:

Simon Krause: s.krause@fkf.mpg.de

Stefan Kaskel: stefan.kaskel@tu-dresden.de

Ben L. Feringa: b.l.feringa@rug.nl

Table of contents

1) Ligand synthesis and characterization 2
2) Photoisomerization of *n*-Bu_4dacdc and H_4dacdc 9
3) Synthesis of metal-organic framework DUT-163 19
4) Supercritical activation of MOF powder 19
5) Single crystal X-ray diffraction 20
6) Powder X-ray diffraction (PXRD) of MOF samples 22
7) Elemental analysis of MOF sample 22
8) Thermogravimetric analysis of MOF samples 23
9) Scanning electron microscopy of MOF samples 23
10) Gas adsorption experiments 24
11) In situ PXRD analysis under exclusion of light. 32
12) *In situ* PXRD analysis under light irradiation 34
13) Rietveld refinement of PXRD data 64
14) *In situ* UV/Vis spectroscopy of solid MOF samples 66
15) In situ DRIFT spectroscopy 71
16) In situ Raman spectroscopy 77
17) Computational analysis of porosity and adsorption properties 80
18) Computational analysis of framework mechanics 82
19) Computational analysis of single ligand ligand buckling 84
20) Computational analysis of the thermal *E-Z* isomerization of Me_4dacdc 84
21) Computational analysis of the charge transfer in Cu_2dacdc 93
22) TD-DFT analysis of the excited state of H_4dacdc 95
23) Supplementary references 96

1) Ligand synthesis and characterization

The *trans*-isomer of ligand (*E*)-H_4dacdc of this work was synthesized based on a strategy applied for the synthesis of a series of structurally related ligands which can be obtained from reference [1]. The azobenzene **1** was prepared by oxidative coupling of 4-bromoaniline according to a procedure previously described in reference [2]. The following synthesis procedure is a direct reproduction from reference [2]

1,2-bis(4-bromophenyl)diazene (azobenzene 1)

A homogeneous mixture of the oxidant was prepared by grinding gently the equal amount of KMnO4 (1 g) and $FeSO_4$-$7H_2O$ (1 g) in a morter. The amine, 4-bromoaniline (0.35 g, 2 mmol) in dichloromethane (20 mL) was taken in a 50 mL round bottomed flask. The oxidant (2.0 g) was added and the heterogeneous mixture stirred under reflux for 5 h. The progress of the reaction was monitored by tlc until no starting material could be detected. After cooling to room temperature the product was then filtered through celite and the residue washed thoroughly with dichloromethane (3 × 15 mL) and diethylether (3 × 15 mL) and dried over anhydrous sodium sulphate. Removal of the solvent and purification of the residue by column chromatography on silica gel gave the corresponding 1,2-bis(4-bromophenyl)diazene in 96% yield.

Azobenzene **1** was reacted in a Cu(I) catalysed Ullman reaction with *n*-butyl ester **2,** which can be obtained in a 5-step synthesis from 9*H*-carbazole following procedures published in reference [1,3] to obtain (*E*)-*nBu4dacdc* in good yield. Ester hydrolysis of (*E*)-*nBu4dacdc* yields (*E*)-H_4L (Supplementary Figure 1).

The following synthesis of 3,6-Dibromo-9H-carbazole, 9H-Carbazole-3,6-dicarbonitrile, 9H-Carbazole-3,6-dicarboxylic acid, and Dibutyl 9H-carbazole-3,6-dicarboxylate is a direct replication of the synthesis procedure as described in reference[1].

3,6-Dibromo-9H-carbazole

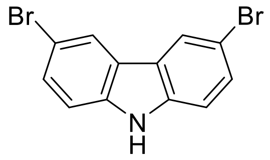

A 1 l flask was charged with 60 g (0.36 mol) 9*H*-carbazole which was previously recrystallized from a mixture of 900 ml toluene and 100 ml ethanol and dissolved in 800 ml anhydrous THF. To the solution 140 g (0.79 mol) *N*-bromosuccinimide was added over 30 min at room temperature and the solution was stirred for 28 h at 30 °C. The THF was removed in vacuum, the remaining solid dissolved in diethyl ether, and the solution extracted with water. The organic phases were collected, dried over $MgSO_4$ and the solvent removed in vacuum. The yellow powder was recrystallized from a mixture of 800 ml chloroform and 50 ml diethyl ether and dried in vacuum to yield 103 g (89%) of white product.

^{1}H NMR (500 MHz, CHLOROFORM-*d*) δ (ppm): 7.33 (d, *J*=8.51 Hz, 2 H) 7.53 (dd, *J*=8.51, 1.89 Hz, 2 H) 8.15 (d, *J*=1.89 Hz, 2 H) 8.33 (br. s., 1 H).

^{13}C NMR (151 MHz, CHLOROFORM-*d*) δ ppm 111.34 (s, 1 C) 113.69 (s, 1 CH) 123.45 (s, 1 CH) 123.99 (s, 1 C) 128.87 (s, 1 CH) 138.91 (s, 1 CH).

ASAP-MS (m/z): Calculated for $C_{12}H_7Br_2N$: 324; found: 324 $[M-H]^+$.

9*H*-Carbazole-3,6-dicarbonitrile

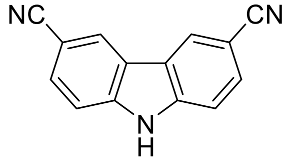

Due to the high toxicity of cyanide the reaction was performed in a sealed fume hood. Deactivation of glassware and tools used for the reaction were performed in a 5% H_2O_2 solution containing ammonia with a pH higher than 11. A 500 ml Schlenk flask was charged with 80 g (246 mmol) of bromide **5** and 656 mg (0.59 mmol) 1,1′-bis(diphenylphosphino)ferrocene. The flask was evacuated, flushed with Ar, and 250 ml DMF and 2.4 ml water were added. In a glovebox a flask with 640 mg (9.9 mmol) Zn powder, 34.5 g (294 mmol) anhydrous zink cyanide, 1.8 g (9.8 mmol) zinc acetate, and 460 mg (0.5 mmol) bis(dibenzylideneacetone)palladium(0) was prepared and the reactants added to the Schlenk flask. The reaction mixture was stirred at 100 °C for 72 h. Afterwards the brown suspension was cooled to room temperature and poured into an aqueous solution of 400 ml saturated ammonium chloride solution and 400 ml concentrated ammonia solution. The off white precipitate was filtered off and washed with the same amount of previously described aqueous solution followed by thorough

washing with water. The waste solutions were collected and deactivated as described above. The off white powder was dried, washed twice with 20 ml methanol, twice with 30 ml toluene, and dried in vacuum. The solid was recrystallized from DMF to yield 48.2 g (90%) of the white product.

^{1}H NMR (500 MHz, DMSO-d_6) δ (ppm): 7.72 (d, *J*=8.51 Hz, 2 H) 7.85 (dd, *J*=8.20, 1.58 Hz, 2 H) 8.79 (s, 2 H) 12.38 (s, 1 H).

^{13}C NMR (151 MHz, DMSO-d_6) δ ppm 101.59 (s, 1 CH) 112.69 (s, 1 CH) 119.95 (s, 1 C) 121.69 (s, 1 C) 126.26 (s, 1 CH) 129.77 (s, 1 CH) 142.16 (s, 1 C).

MS-ASAP (m/z): Calculated for $C_{14}H_7N_3$: 218; found: 218 $[M+H]^+$.

Elemental analysis: Calculated: C: 77.41%; H: 3.25%; N: 19.34%; found: C: 78.19%; H: 3.056%; N: 19.47%.

9*H*-Carbazole-3,6-dicarboxylic acid

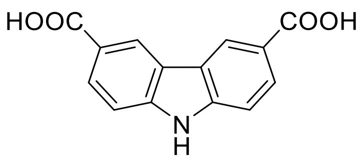

In a 2 l flask 30 g (0.13 mol) cyanide **6**, 90 g (2.25 mol) sodium hydroxide, and 300 mg (1.58 mmol) copper (I) iodide were dissolved in 1 L water and stirred under reflux for 24 h. the solution was cooled down to room temperature and filtered over Celite®. The solution was neutralized with 6 M hydrochloric acid, the white precipitate was filtered off, washed thoroughly with water, and dried at 80 °C to yield 33 g (93%) off white powder.

^{1}H NMR (500 MHz, DMSO-d_6) δ ppm 7.57 (d, *J*=8.51 Hz, 2 H) 8.03 (dd, *J*=8.51, 1.89 Hz, 2 H) 8.79 - 8.85 (m, 2 H) 12.02 (s, 1 H)

^{13}C NMR (126 MHz, DMSO-d_6) δ ppm 11.121 (s, 1 CH), 122.03 (s, 1 C) 122.33 (s, 1 C) 122.95 (s, 1 CH) 127.80 (s, 1 CH) 143.26 (s, 1 C) 167.07 (s, 1 C) 168.82 (s, 1 C).

MS-ASAP (m/z): Calculated for $C_{14}H_9NO_4$: 256; found: 256 $[M+H]^+$.

Elemental analysis: Calculated ($C_{14}H_9NO_4$ 0.55·H_2O): C: 63.42%; H: 3.84%; N: 5.28%; found: C: 63.44%; H: 3.681%; N: 5.08%.

DRIFT, KBr, 298 K (cm^{-1}): 3471 (w) , 2823 (w) , 2644 (w) , 1913 (w) , 1789 (m) , 1681 (s) , 1633 (m) , 1606 (s) , 1585 (m) , 1491 (w) , 1470 (m) , 1451 (m) , 1419 (s) , 1354 (m) , 1288 (s) , 1254 (s) , 1217 (m) , 1137 (w) , 1024 (m) , 931 (m) , 898 (m) , 851 (w) , 828 (m) , 802 (w) , 769 (s) , 724 (m) , 682 (w).

Dibutyl 9*H*-carbazole-3,6-dicarboxylate

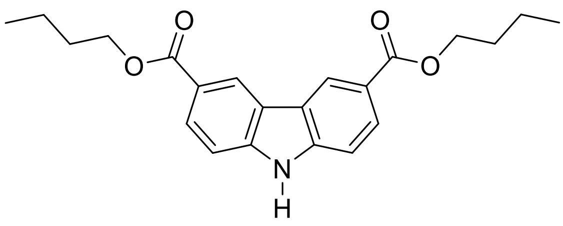

In a 1 L flask 33 g (0.13 mmol) H_2CDC **1** was suspended in 750 ml 1-butanol and 3 ml of sulfuric acid was added. The mixture was refluxed at 130 °C for 48 h to form a clear yellow solution. The 1-butanol was removed in vacuum, the resulting solid dissolved in chloroform, and extracted with diluted aqueous potassium carbonate solution. The organic phases were combined, dried over $MgSO_4$ and the solvent removed in vacuum. The obtained solid was recrystallized from ethyl acetate to obtain 45 g (95%) white powder.

^{1}H NMR (600 MHz, CHLOROFORM-*d*) δ (ppm): 1.04 (t, *J*=7.34 Hz, 6 H) 1.51 - 1.60 (m, 4 H) 1.81 - 1.88 (m, 4 H) 4.41 (t, *J*=6.59 Hz, 4 H) 7.48 (d, *J*=8.28 Hz, 2 H) 8.19 (dd, *J*=8.47, 1.69 Hz, 2 H) 8.57 (br. s., 1 H) 8.87 (d, *J*=1.51 Hz, 2 H).

^{13}C NMR (151 MHz, CHLOROFORM-*d*) δ (ppm): 13.83 (s, 1 CH_3) 19.36 (s, 1 CH_2) 30.93 (s, 1 CH_2) 64.82 (s, 1 CH_2) 110.47 (s, 1 CH) 122.75 (s, 1 C) 123.04 (s, 1 CH) 123.14 (s, 1 C) 128.13 (s, 1 CH) 142.66 (s, 1 C) 167.20 (s, 1 C).

MALDI-TOF-MS (m/z): Calculated for $C_{36}H_{20}Br_4N_2$: 294; found 294 $[M-C_4H_9OH]^+$.

Elemental analysis: Calculated: C: 71.91%; H: 6.86%; N: 3.81%; found: C: 72.01%; H: 6.443%; N: 3.68%.

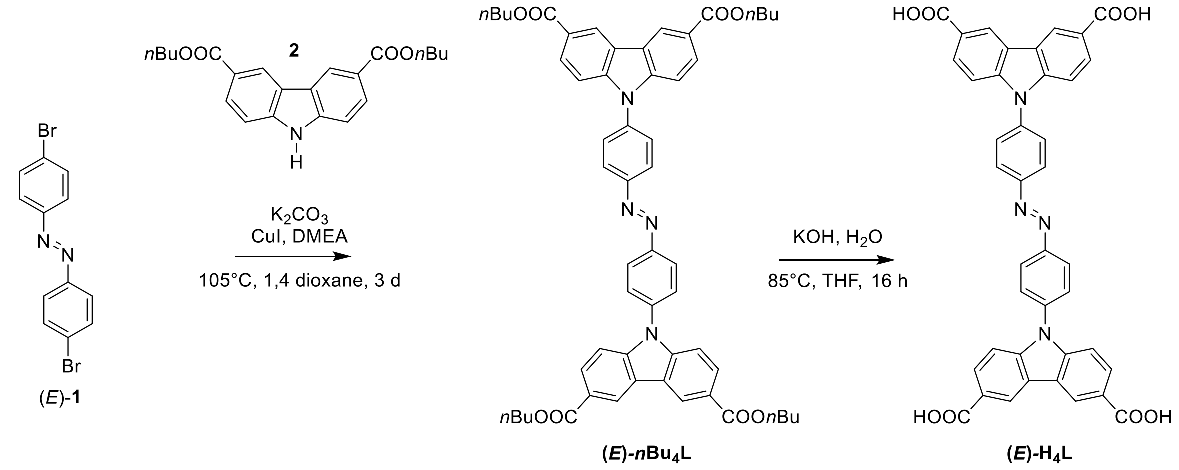

Supplementary Figure 1. Reaction scheme for the synthesis of (E)-H_4L.

Tetrabutyl 9,9'-(diazene-1,2-diylbis(4,1-phenylene))(*E*)-bis(9*H*-carbazole-3,6-dicarboxylate) ((*E*)-*nBu4dacdc*)

A Schlenk flask was charged with 0.95 g (2.79 mmol) bromine **1,** 3.1 g (8.38 mmol) ester **2**, 1.54 g (11.16 mmol) potassium carbonate, and 0.2 g (1.05 mmol) copper(I)iodine. To the mixture 40 ml of degassed 1,4 dioxane and 0.1 ml (0.91 mmol) *N,N'*-dimethylethylenediamine were given and Ar was bubbled through the suspension for 30 min under stirring. The reaction mixture was stirred at 105°C for 24 h 3 d and the reaction was cooled down to room temperature. The suspension was quenched with diluted (< 0.02 M) Hydrochloric acid and extracted with chloroform. The organic phases were collected, dried over $MgSO_4$, and the solvent removed in vacuum. The crude product was purified by flash column chromatography using a DCM : *iso*-hexane : ethyl acetate – 1 : 0.3 : 0.014 (R_f 0.43) and (*E*)-*nBu4dacdc* was obtained as a red solid which was stored under exclusion of light. Yield: 1.83 g (72%) red powder.

^{1}H NMR (600 MHz, CHLOROFORM-*d*) δ (ppm): 1.04 (t, *J*=7.37 Hz, 3 H)) 1.52 - 1.61 (m, 2 H) 1.81 – 1.86 (m, 2 H) 4.44 (t, *J*=6.59 Hz, 2 H) 7.53 (d, *J*=8.66 Hz, 1 H) 7.80 (d, *J*=8.66 Hz, 1 H) 8.18 (dd, *J*=8.66, 1.51 Hz, 1 H) 8.22 (dd, *J*=8.47, 1.69 Hz, 1 H) 8.29 (d, *J*=8.66 Hz, 1 H) 8.96 (s, 1 H).

^{13}C NMR (151 MHz, CHLOROFORM-*d*) δ (ppm): 13.84 (s, 1 CH_3) 19.38 (s, 1 CH_2) 30.94 (s, 1 CH_2) 64.94 (s, 1 CH_2) 109.80 (s, 1 CH) 123.09 (s, 1 CH) 123.46 (s, 1 C) 123.62 (s, 1 C) 124.86 (s, 1 CH) 127.61 (s, 1 CH) 127.92 (s, 1 C) 128.33 (s, 1 C) 128.38 (s, 1 CH) 143.82 (s, 1 C) 166.98 (s, 1 C).

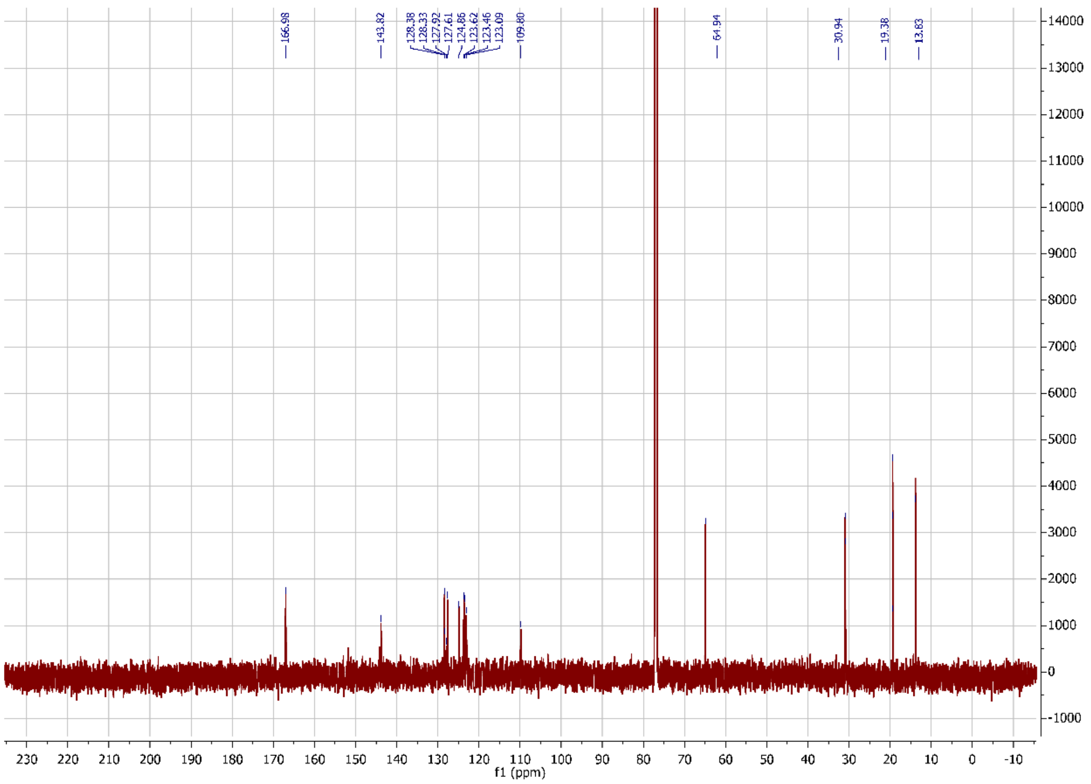

Supplementary Figure 2. ^{13}C NMR spectra of (*E*)-*n*-Bu_4dacdc

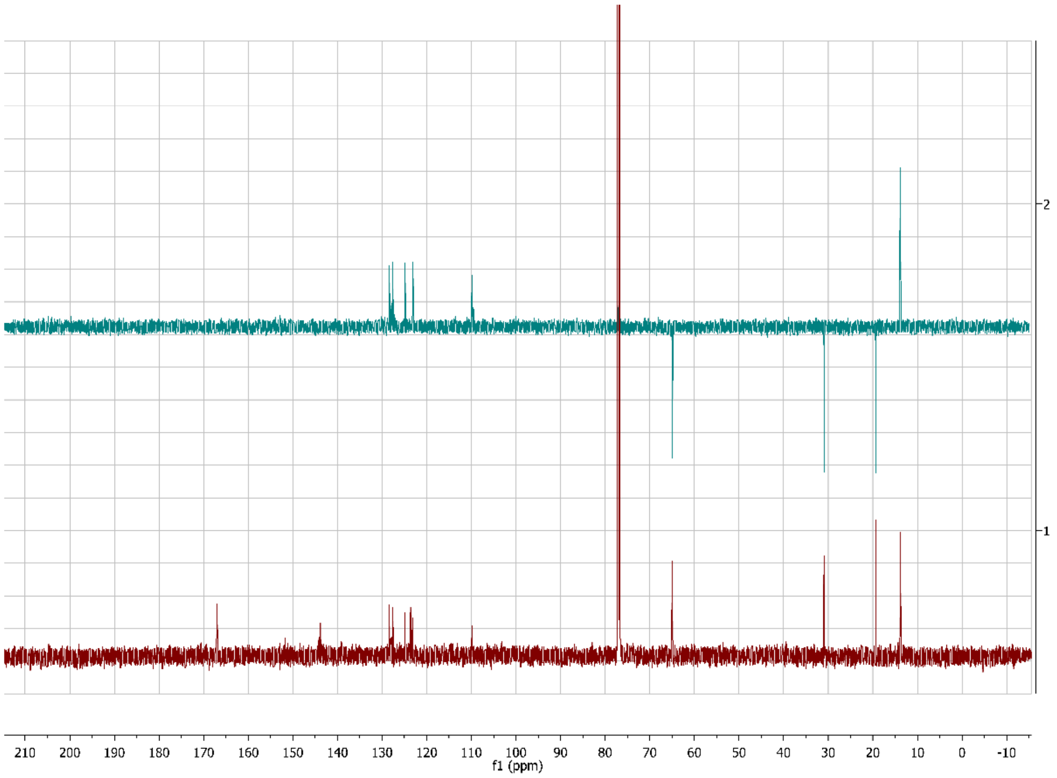

Supplementary Figure 3. ^{13}C DEPT NMR spectra of (*E*)-*n*-Bu_4dacdc

MALDI-TOF-MS (*m/z*): Calculated for $C_{56}H_{56}N_4O_8$: 839.3445; found 839.2616 $[M-C_2H_5OH]^+$.

Elemental analysis: Calculated: C: 73.66%; H: 6.18%; N: 6.14%; found: C: 73.12%; H: 6.013%; N: 6.02%.

(*E*)-9,9'-(diazene-1,2-diylbis(4,1-phenylene))bis(9*H*-carbazole-3,6-dicarboxylic acid) ((*E*)-H_4L)

A flask was charged with 1.1 g (1.2 mmol) ((*E*)-*nBu4dacdc*) which was dissolved in 45 ml THF 4 ml methanol at 85 °C. To the solution 0.55 g (9.78 mmol) potassium hydroxide and 2 ml of water were added and the mixture was stirred at 85 °C for 16 h. THF and methanol was removed in vacuum, the resulting solution was filtered, and neutralized with 2 M hydrochloric acid. The yellow precipitate was filtered off, washed with water and ethanol and dried in vacuum at room temperature to yield a red solid which was stored under exclusion of light. Yield: 0.81 g (97%) red powder.

^{1}H NMR (600 MHz, DMSO-d_6) δ ppm 7.61 (d, *J*=8.66 Hz, 1 H) 7.98 (d, *J*=8.28 Hz, 1 H) 8.13 (dd, *J*=8.47, 1.69 Hz, 1 H) 8.28 (d, *J*=8.66 Hz, 1 H) 9.00 (d, *J*=1.51 Hz, 1 H) 12.88 (br. s., 1 H).

^{13}C NMR (151 MHz, DMSO-d_6) δ ppm 110.21 (s, 1 CH) 122.89 (s, 1 CH) 123.14 (s, 1 C) 123.87 (s, 1 CH) 124.7 (s, 1 CH) 127.99(s, 1 C) 128.5 (s, 1 CH) 138.68 (s, 1 C) 143.21 (s, 1 C) 151.17 (s, 1 C) 167.65 (s, 1 C).

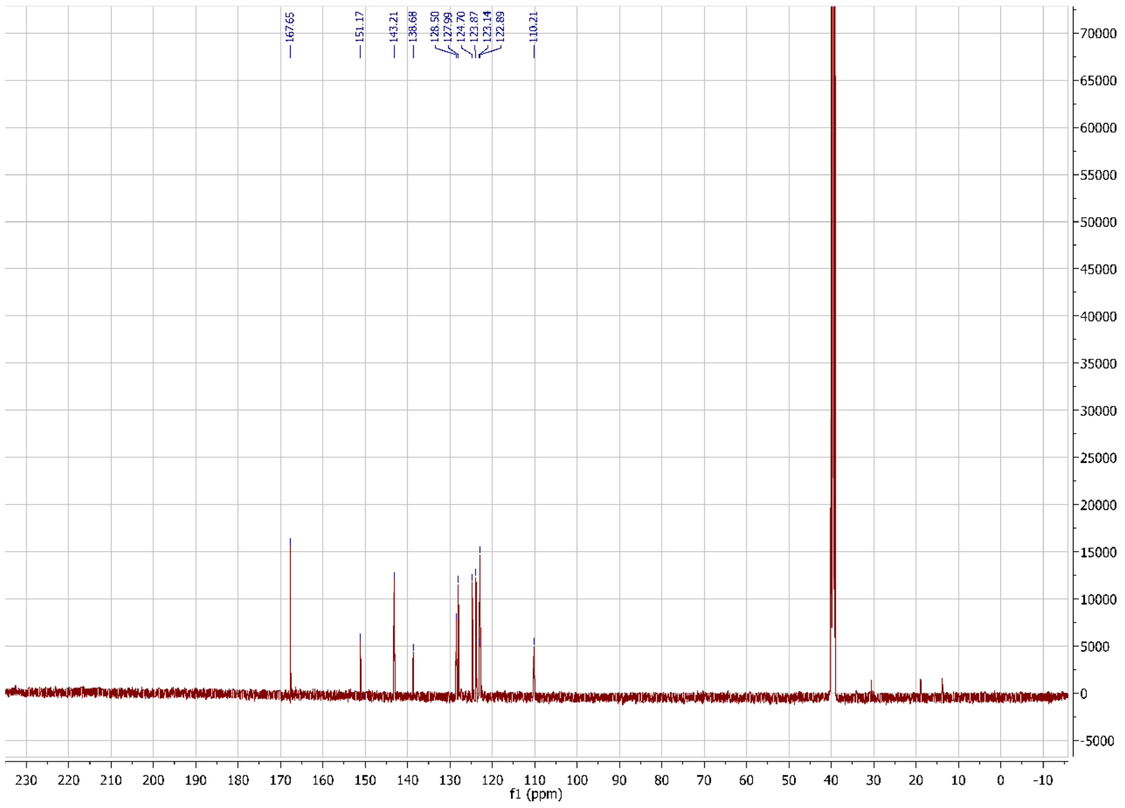

Supplementary Figure 4. ^{13}C NMR spectra of (*E*)-H_4dacdc

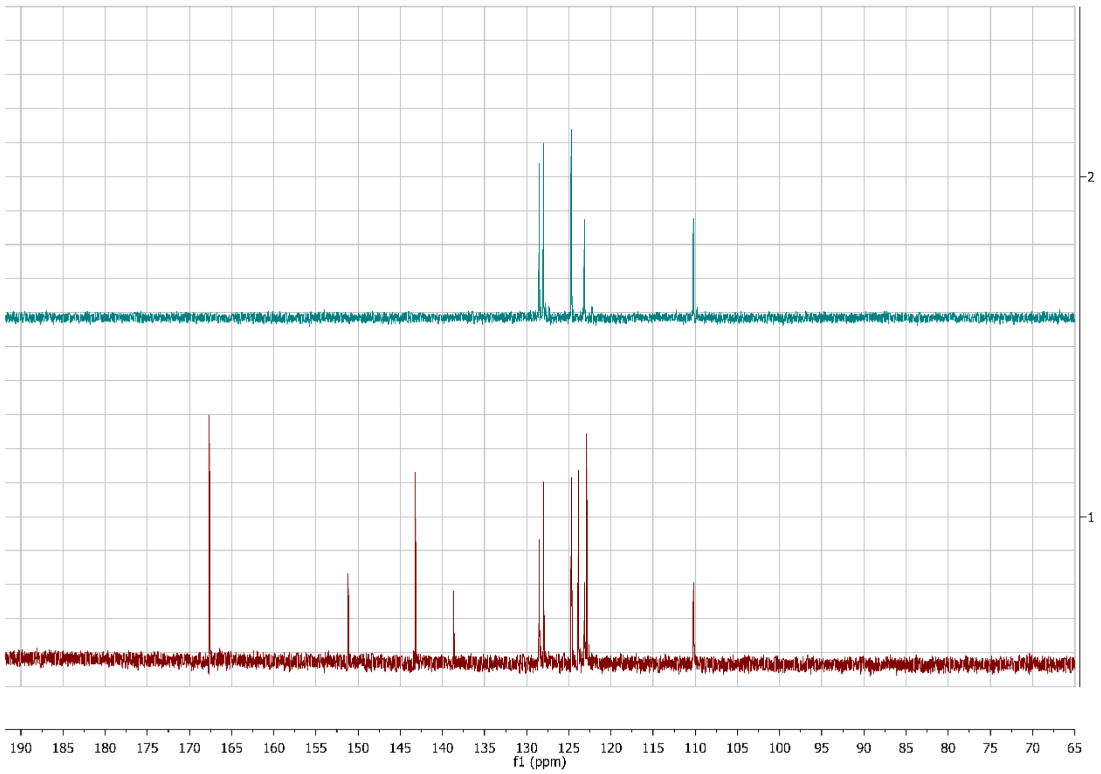

Supplementary Figure 5. ^{13}C DEPT NMR spectra of (*E*)-H_4dacdc

MALDI-TOF-MS (*m/z*): Calculated for $C_{40}H_{24}N_4O_8$: 687.1516; found 687.8055 $[M-H]^+$.

Elemental analysis: Calculated: C: 69.77%; H: 3.51%; N: 8.14%; found: C: 69.71%; H: 3.293%; N: 8.23%.

2) Photoisomerization of *n*-Bu₄dacdc and H₄dacdc

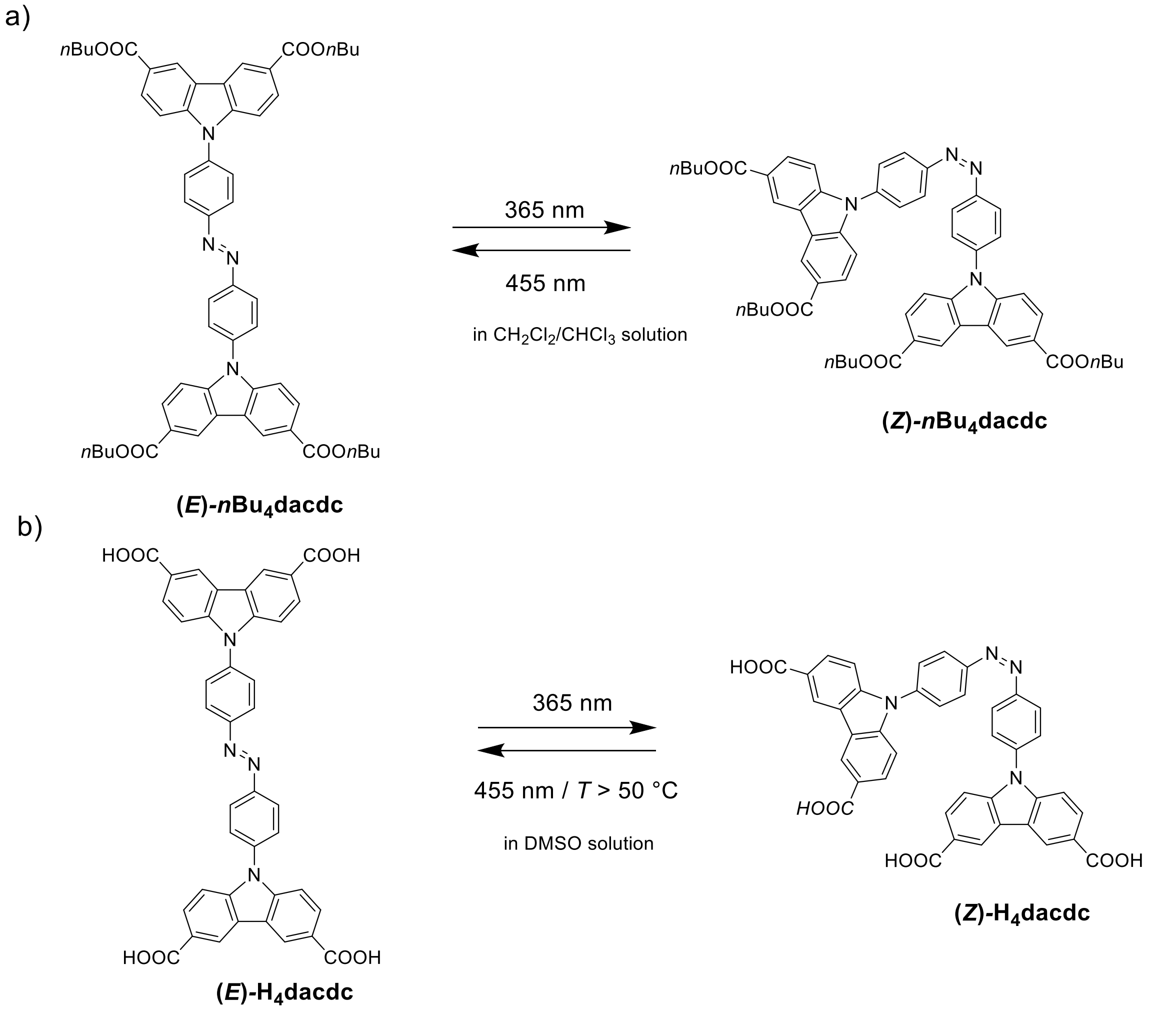

Supplementary Figure 6. a) *E*-*Z*-isomerization of (*E*)-nBu$_4$dacd*c* upon 365 nm irradiation into (*Z*)-nBu$_4$dacd*c*. b) *E*-*Z*-isomerization of (*E*)-H$_4$dacdc upon 365 nm irradiation into (*Z*)-H$_4$dacdc.

For all experiments described in this study irradiation at 365 nm was conducted with M365L2 Thorlabs LED and at 455 nm with M455L3-C5 Thorlabs LED. For UV-Vis spectroscopic analysis solutions with concentrations of 2.1×10^{-5} mol ml^{-1} ((*E*)-H$_4$dacdc) and 1.4×10^{-5} mol ml^{-1} ((*E*)-*n*Bu$_4$dacdc) were prepared in HPLC grade DMSO and Chloroform, respectively. Before spectroscopic analysis the solutions were degassed by bubbling with Ar for 5 min.

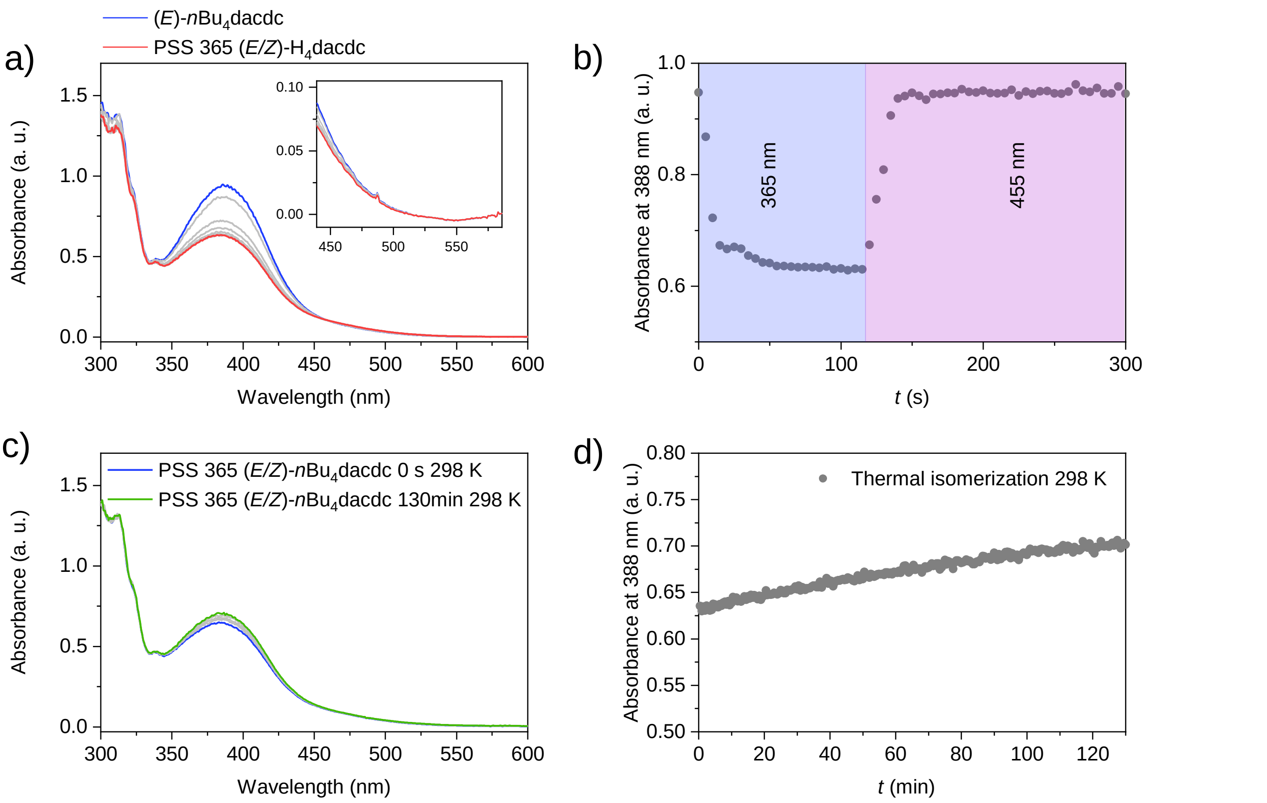

Supplementary Figure 7. UV-Vis absorption spectra of a) (*E*)-*n*-Bu_4dacdc (blue) in $CHCl_3$ upon irradiation at 365 nm (PSS in red), b) evolution of absorbance at 388 nm upon irradiation at 365 nm and 455 nm, c) UV-Vis absorption spectra of PSS at 298 K, d) evolution of absorbance at 388 nm upon thermal relaxation at 298 K.

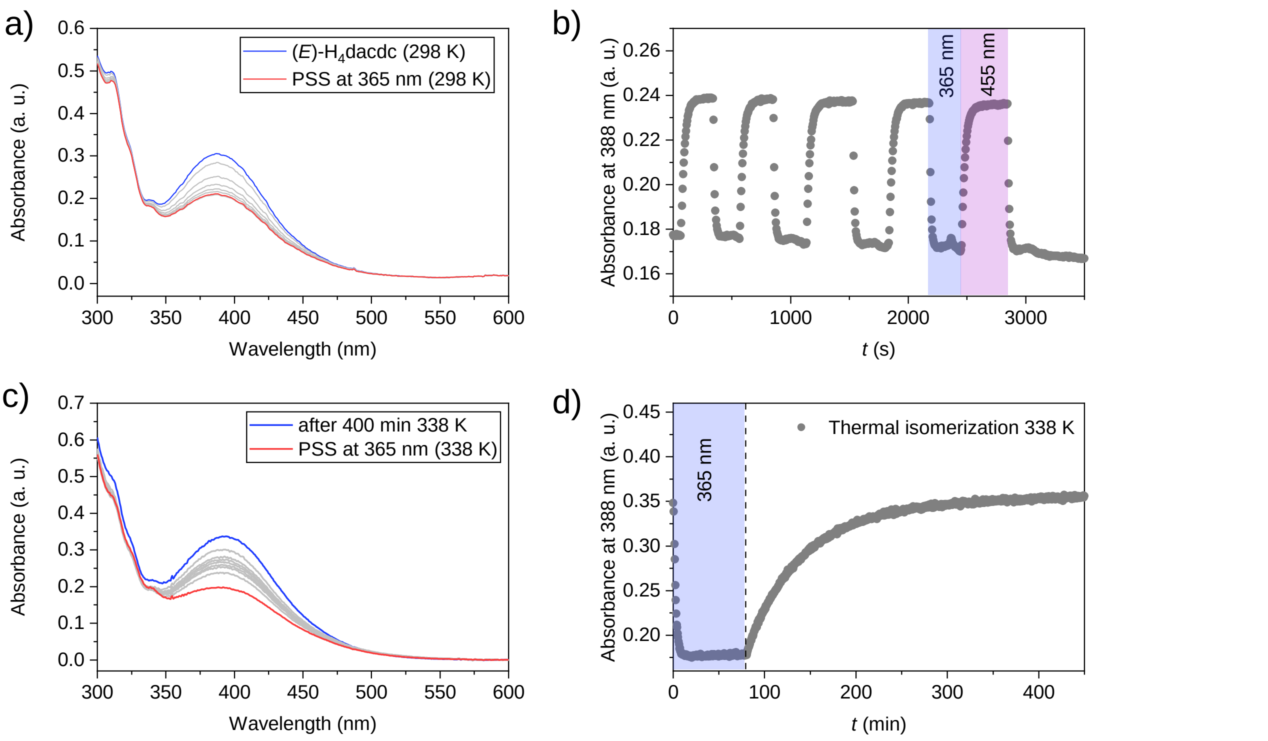

Supplementary Figure 8. UV-Vis absorption spectra of a) (*E*)-H_4dacdc (blue) in DMSO upon irradiation at 365 nm (PSS in red), b) evolution of absorbance at 388 nm upon repeated irradiation at 365 nm and 455 nm, c) UV-Vis absorption spectra of PSS at 338 K, d) evolution of absorbance at 388 nm upon thermal relaxation at 338 K.

For *in situ* NMR analysis solutions of 1 mg ml^{-1} in d^6-DMSO ((*E*)-H_4dacdc) and CD_2Cl_2 ((*E*)-*n*Bu_4dacdc) were prepared. Before spectroscopic analysis the solutions were degassed by bubbling with Ar for 5 min. NMR spectra of the phot stationary state were recorded after irradiation at 365 nm for 95 min. In the case of both ((*E*)-H_4dacdc and (*E*)-*n*Bu_4dacdc the photo stationary state (PSS) was found to contain 50% mixture of the *Z*-isomer. *Z-E* isomerization was conducted by irradiation for 30-40 min. The PSS was found to contain 25% mixture of the *Z*-isomer.

From the PSS of (*E/Z*)-*n*Bu_4dacdc the spectra of the two different isomers was determined:

^{1}H NMR **(*E*)-*nBu4dacdc*** (500 MHz, DICHLOROMETHANE-*d*$_2$) d ppm 1.08 (t, *J*=7.33 Hz, 3 H) 1.55 - 1.74 (m, 2 H) 1.83 - 1.96 (m, 2 H) 4.43 (t, *J*=6.59 Hz, 2 H) 7.60 (d, *J*=8.79 Hz, 1 H) 7.87 (d, *J*=8.42 Hz, 1 H) 8.25 (d, *J*=8.8 Hz, 1 H) 8.35 (d, *J*=8.42 Hz, 1 H) 9.01 (s, 1 H)

^{1}H NMR **(*Z*)-*nBu4dacdc*** (500 MHz, DICHLOROMETHANE-*d*$_2$) d ppm 1.08-1.10 (m, 3 H) 1.55 - 1.74 (m, 2 H) 1.83 - 1.96 (m, 2 H) 4.43 (m, 2 H) 7.31 (d, *J*=8.30 Hz, 1 H) 7.42 (d, *J*=8.79 Hz, 1 H) 7.64 (d, *J*=8.30 Hz, 1 H) 8.15 (d, *J*=8.79 Hz, 1 H) 8.25 (d, *J*=8.79 Hz, 1 H) 8.97 (s, 1 H)

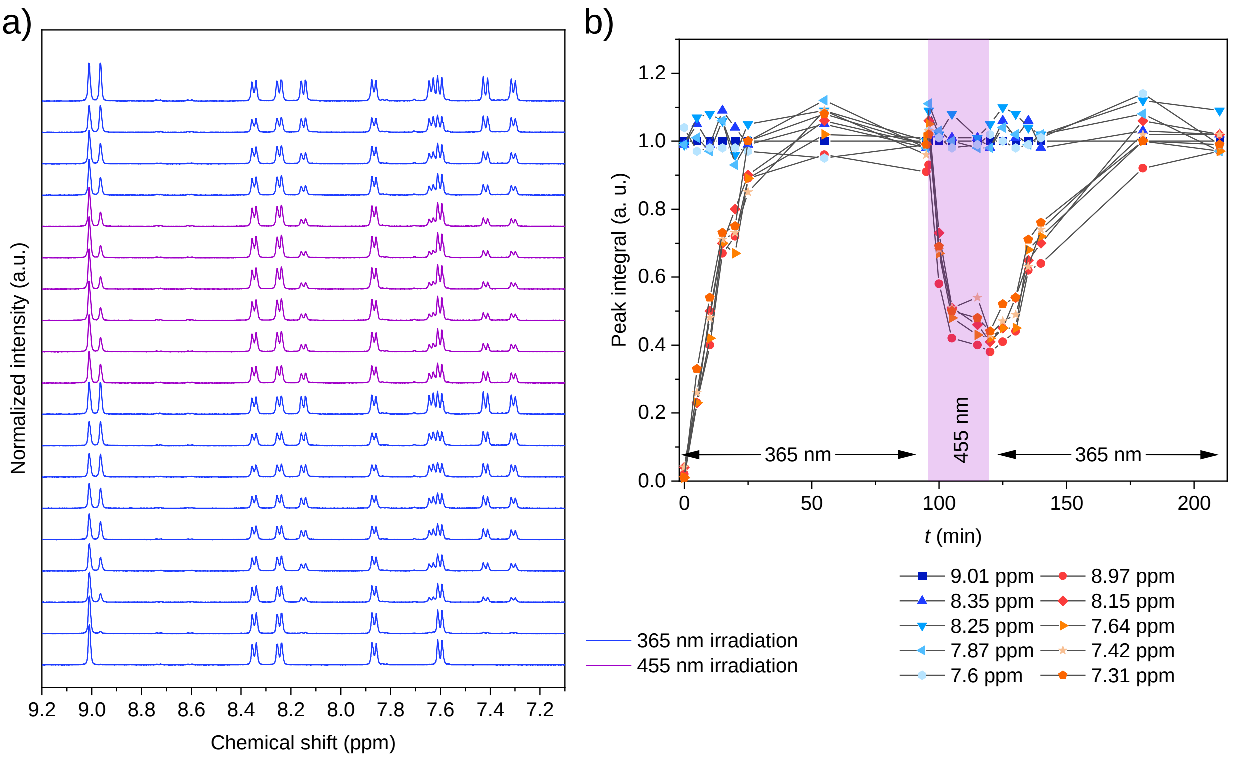

Supplementary Figure 9. a) Aromatic region of the ^{1}H NMR spectrum of (*E*)-*n*Bu_4dacdc upon irradiation at 365nm and 455 nm at 298 K. b) Evolution of the signal integrals upon irradiation, signals of (*E*)-*n*Bu_4dacdc in blue symbols, signals of (*Z*)-*n*Bu_4dacdc in orange symbols.

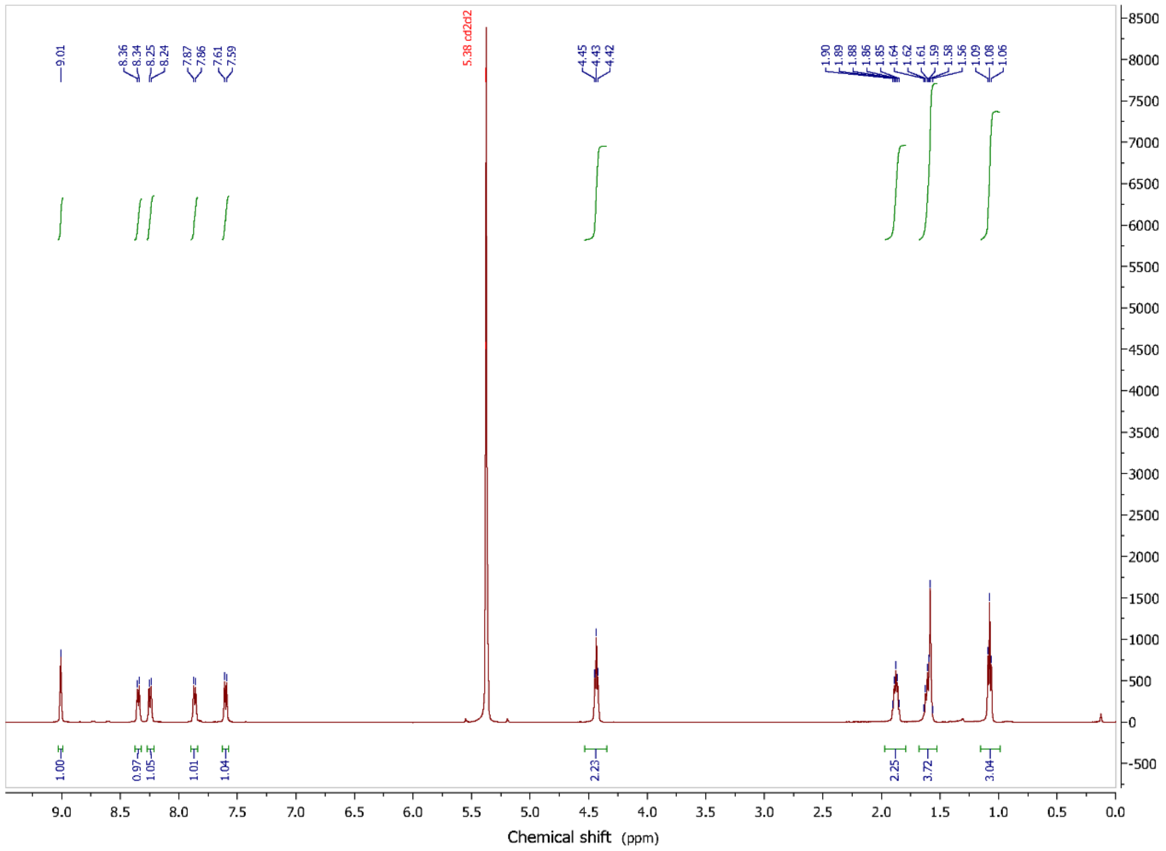

Supplementary Figure 10. ^{1}H NMR spectrum of (*E*)-*n*Bu$_4$dacdc under light exclusion at 298 K.

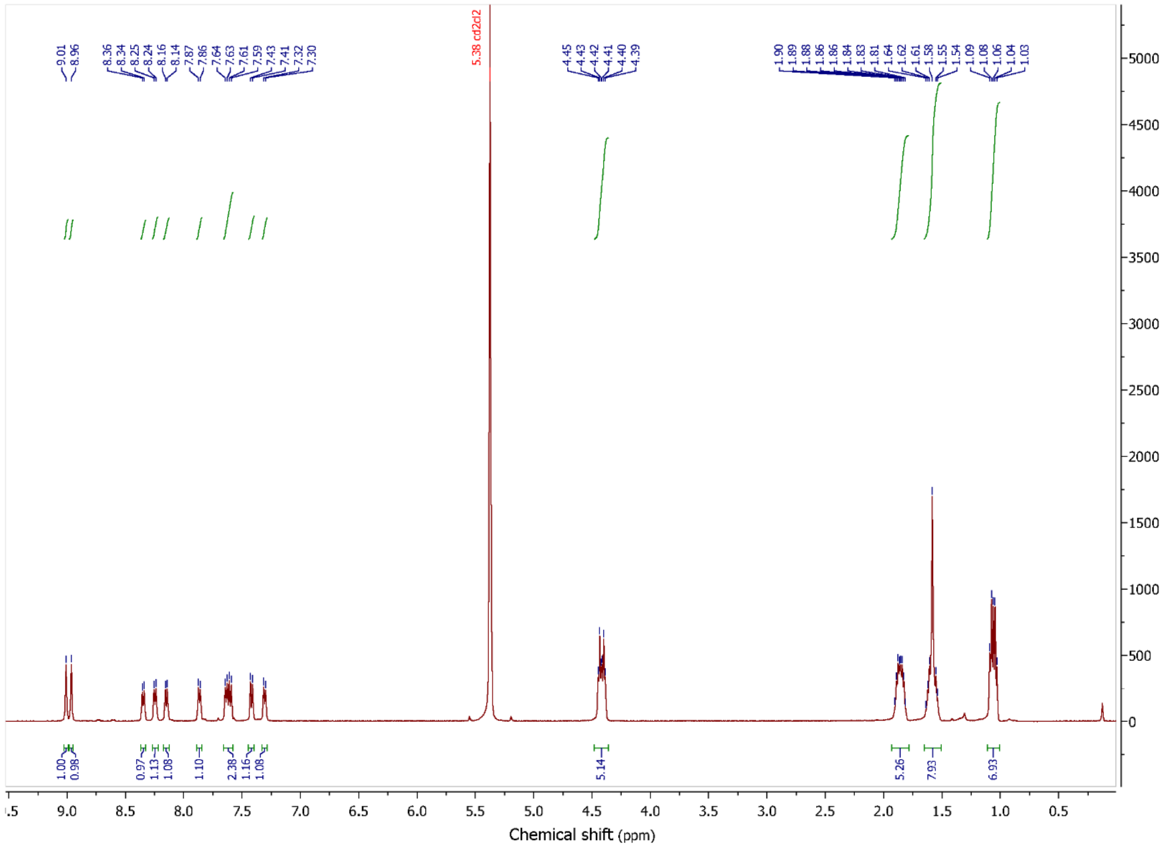

Supplementary Figure 11. ^{1}H NMR spectrum of (*E/Z*)-*n*Bu$_4$dacdc PSS upon irradiation at 365 nm for 95 min at 298 K.

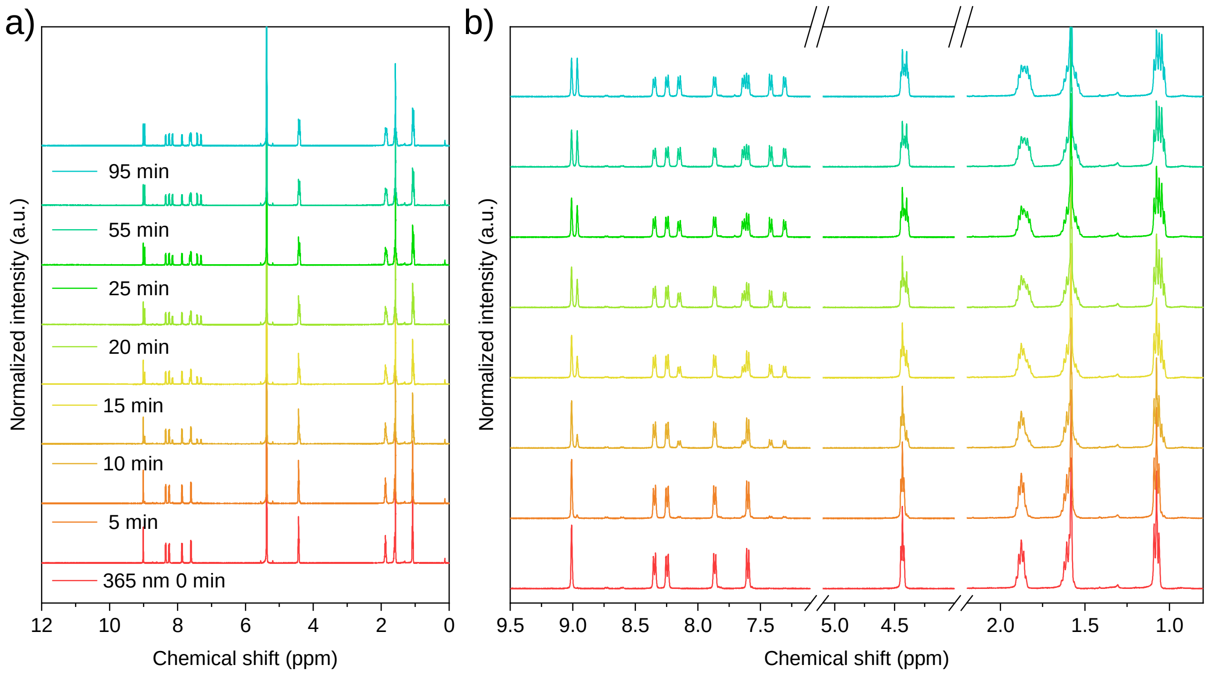

Supplementary Figure 12. ^{1}H NMR spectrum of (*E*)-*n*Bu_4dacdc upon irradiation at 365nm and 298 K.

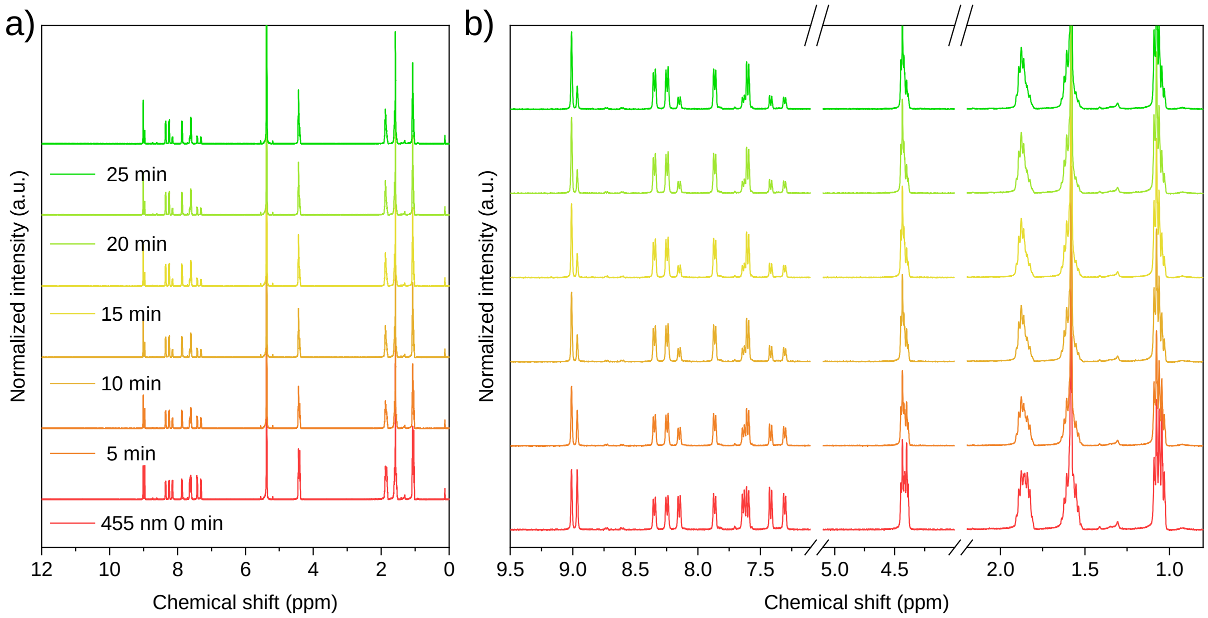

Supplementary Figure 13. ^{1}H NMR spectrum of (*E*)-*n*Bu_4dacdc upon irradiation at 455nm and 298 K from PSS after 95 min irradiation at 365 nm.

From the PSS of (*E/Z)*-H_4dacdc the spectra of the two different isomers was determined:

^{1}H NMR **(*E*)-H$_4$L** (500 MHz, DMSO-*d*$_6$) d ppm 7.63 (d, *J*=8.79 Hz, 1 H) 8.01 (d, *J*=8.30 Hz, 1 H) 8.15 (d, *J*=8.30 Hz, 1 H) 8.31 (d, *J*=8.30 Hz, 1 H) 9.03 (s, 1 H), 12.88 (br., 1 H).

^{1}H NMR **(*Z*)-H$_4$L** (500 MHz, DMSO-*d*$_6$) d ppm 7.34 (d, *J*=7.32 Hz, 1 H) 7.38 (d, *J*=8.30 Hz, 1 H) 7.76 (d, *J*=6.84 Hz, 1 H) 8.01 (m, *1* H) 8.97 (s, 1 H) 12.84 (br., 1 H).

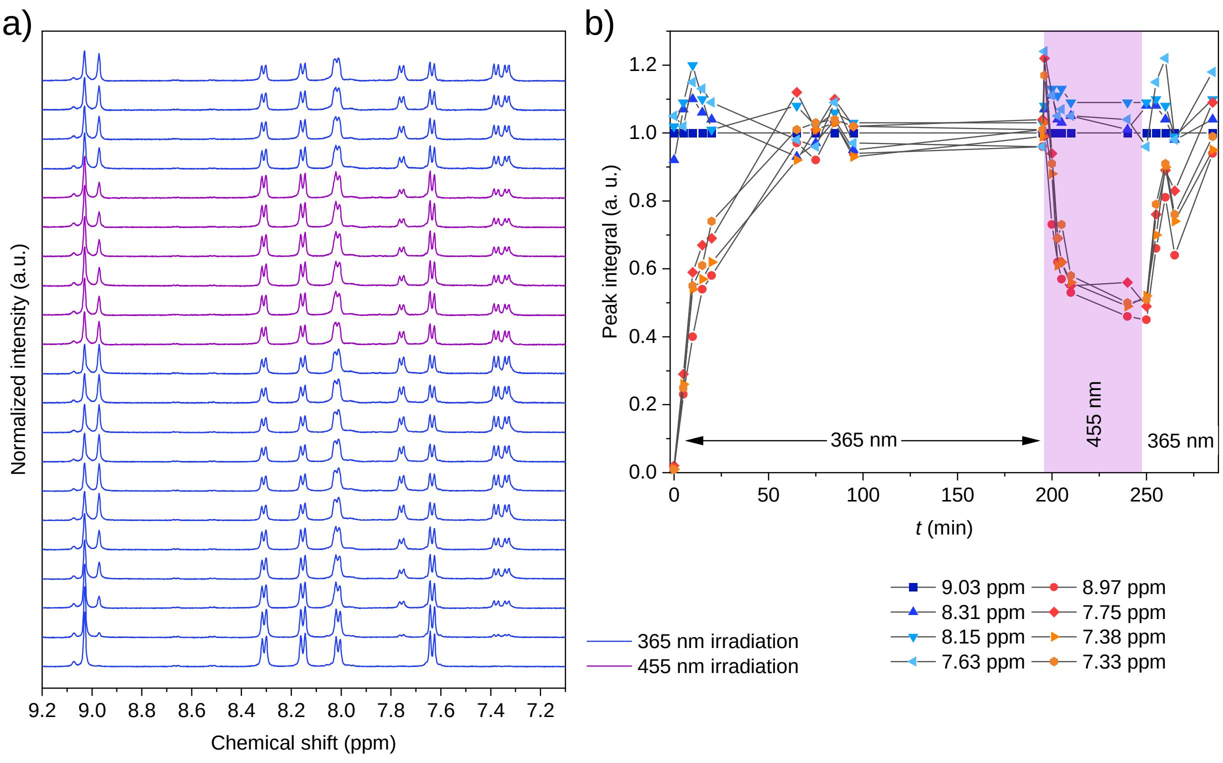

Supplementary Figure 14. a) Aromatic region of the ^{1}H NMR spectrum of (*E*)-H_4dacdc upon irradiation at 365nm and 455 nm at 298 K. b) Evolution of the signal integrals upon irradiation, signals of (*E*)-*n*Bu_4dacdc in blue symbols, signals of (*Z*)-*n*Bu_4dacdc in orange symbols. Peak integrals were normalized to the peak at 9.03 ppm.

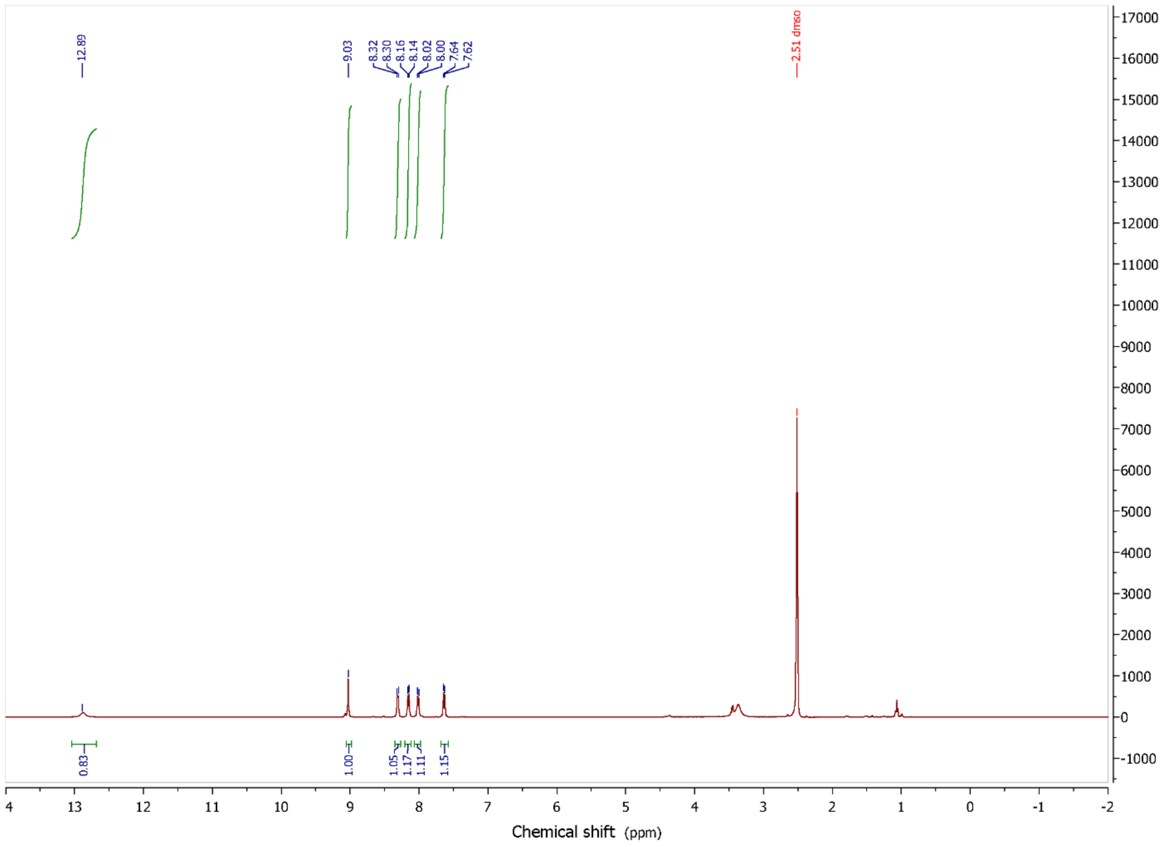

Supplementary Figure 15. ^{1}H NMR spectrum of (*E*)-H_4dacdc under light exclusion at 298 K.

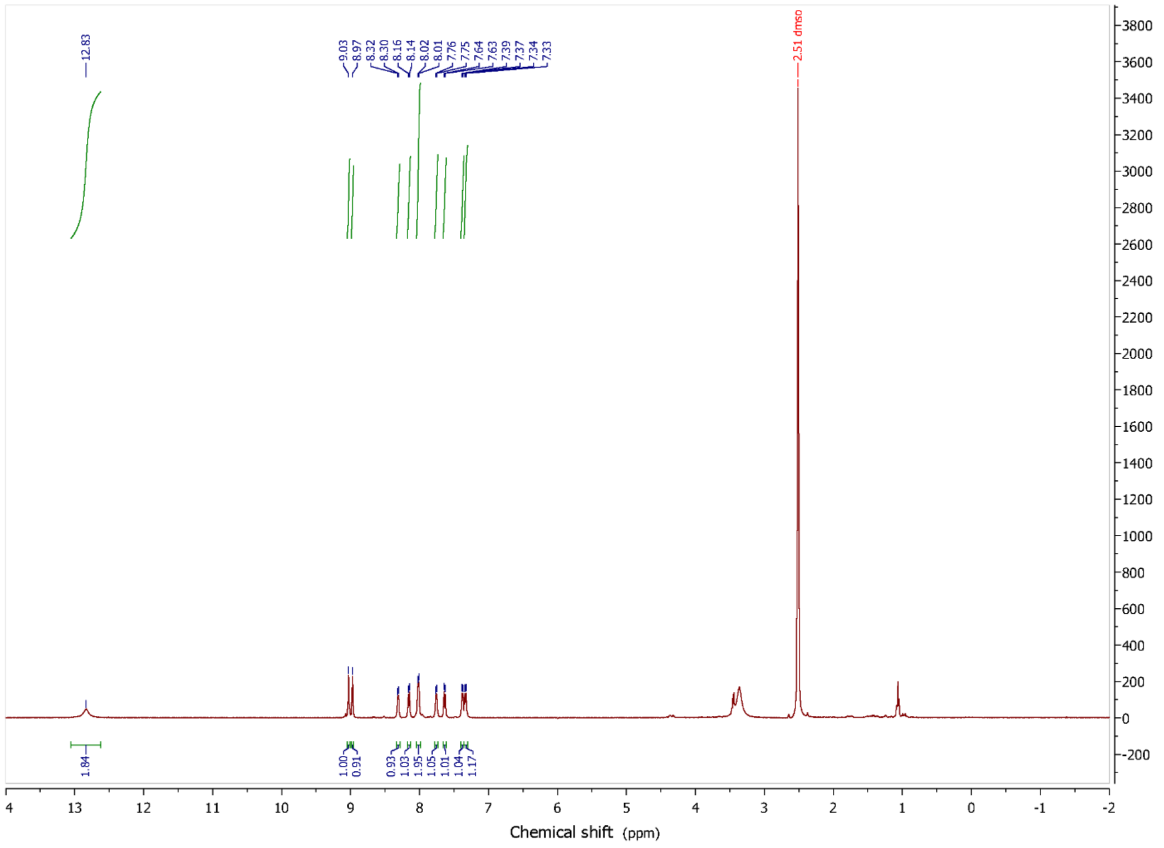

Supplementary Figure 16. ^{1}H NMR spectrum of (*E/Z*)-H_4dacdc PSS upon irradiation at 365 nm for 95 min at 298 K.

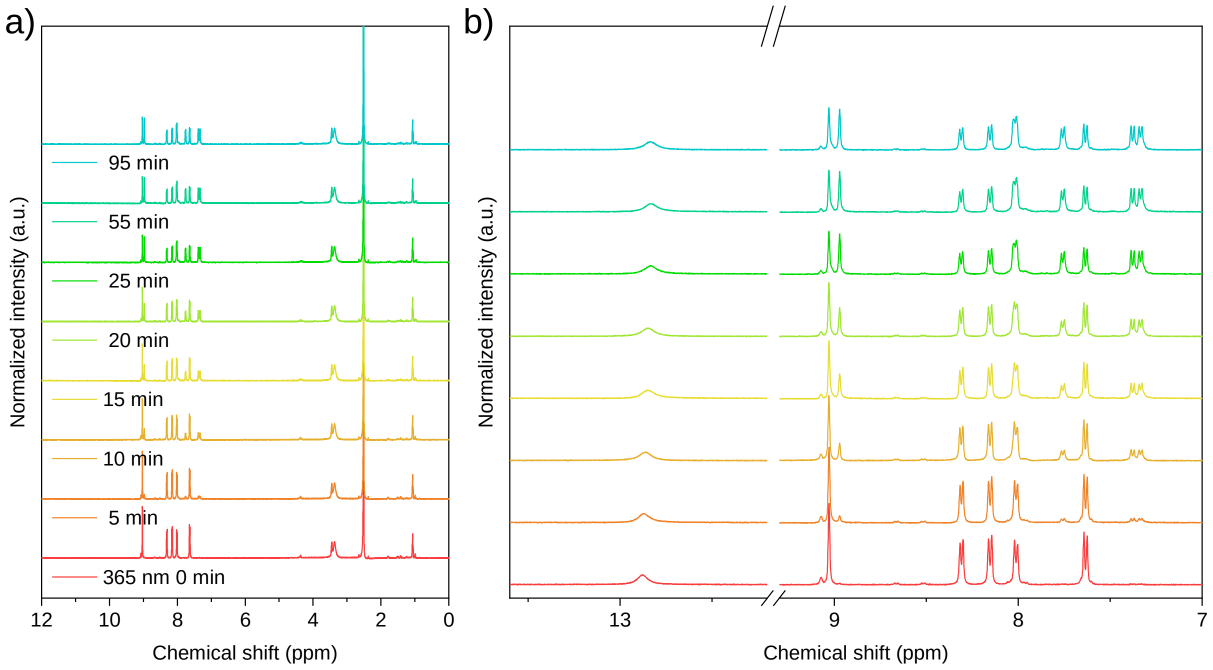

Supplementary Figure 17. a) Full ^{1}H NMR spectrum of (*E*)-H_4dacdc upon irradiation at 365nm and 298 K, b) magnification of the carboxylic and aromatic region.

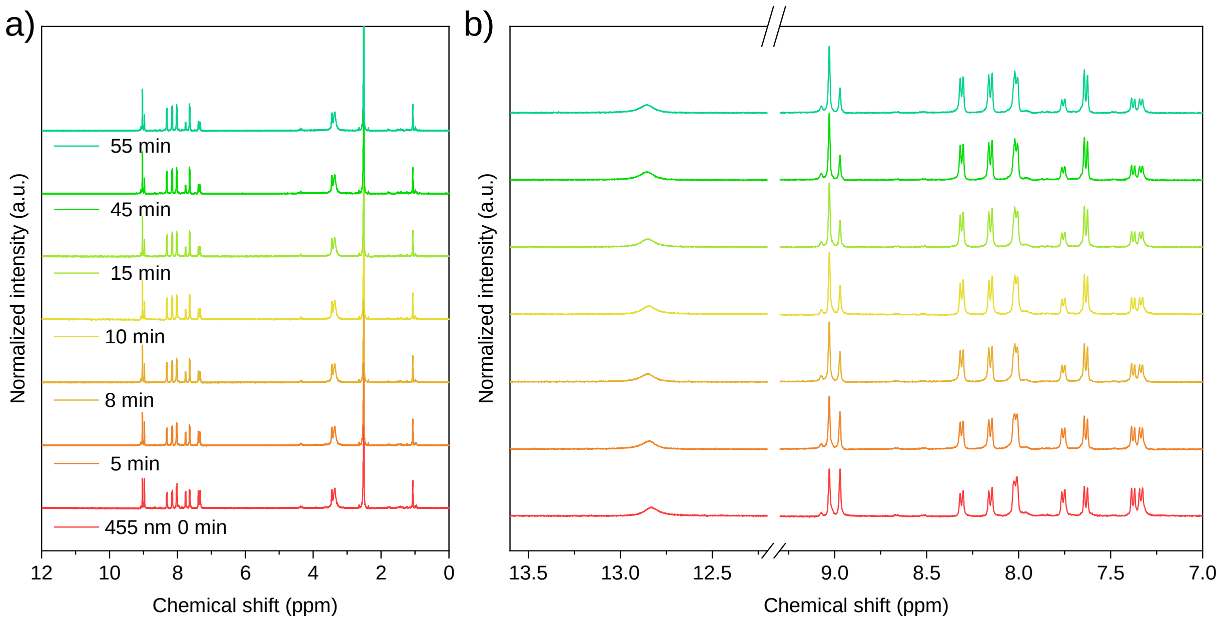

Supplementary Figure 18. a) full ^{1}H NMR spectrum of (*E*)-H_4dacdc upon irradiation at 455nm and 298 K from PSS after 95 min irradiation at 365 nm, b) magnification of the carboxylic and aromatic region.

Raman spectra of *n*-Bu_4dacdc were acquired in solution (5 mg ml^{-1}) in $CHCl_3$. Raman spectra were post-processed using Spectragryph software. For time series, spectra containing cosmic spikes were removed from the series and the remaining spectra were averaged in the groups of 16 to increase the signal to noise ratio. Upon irradiation of (*E*)-*n*-Bu_4dacdc at 365 nm a continuous decrease in the Raman intensity of several bands was observed. At the PSS after 60 min irradiation, the intensity of the relevant bands decreased by ca. half of the initial value. These data are consistent with the NMR data which showed that the PSS upon irradiation at 365 nm consists of approximately 1:1 of (*E)*- and (Z)-*n*-Bu_4dacdc. At the same time only a small increase in the Raman scattering was observed around 1520 cm^{-1}, which probably stems from lower polarizability of the *Z*-isomer. Since the Raman intensity is proportional to polarizability squared, even small differences in the polarizability between both isomers are drastically magnified. As a consequence, bands characteristic of (*Z*)-*n*-Bu_4dacdc could not be observed in the PSS mixture at the studied concentration. Subsequent irradiation of this mixture at 455 nm for 60 min resulted in an increase in the Raman intensity of the relevant bands up to ca. 75% of the initial value, in line with *Z*-*E* back isomerization. These observations are consistent with the determined composition of visible-light PSS upon irradiation with 455 nm determined by NMR spectroscopy (Supplementary Figure 13).

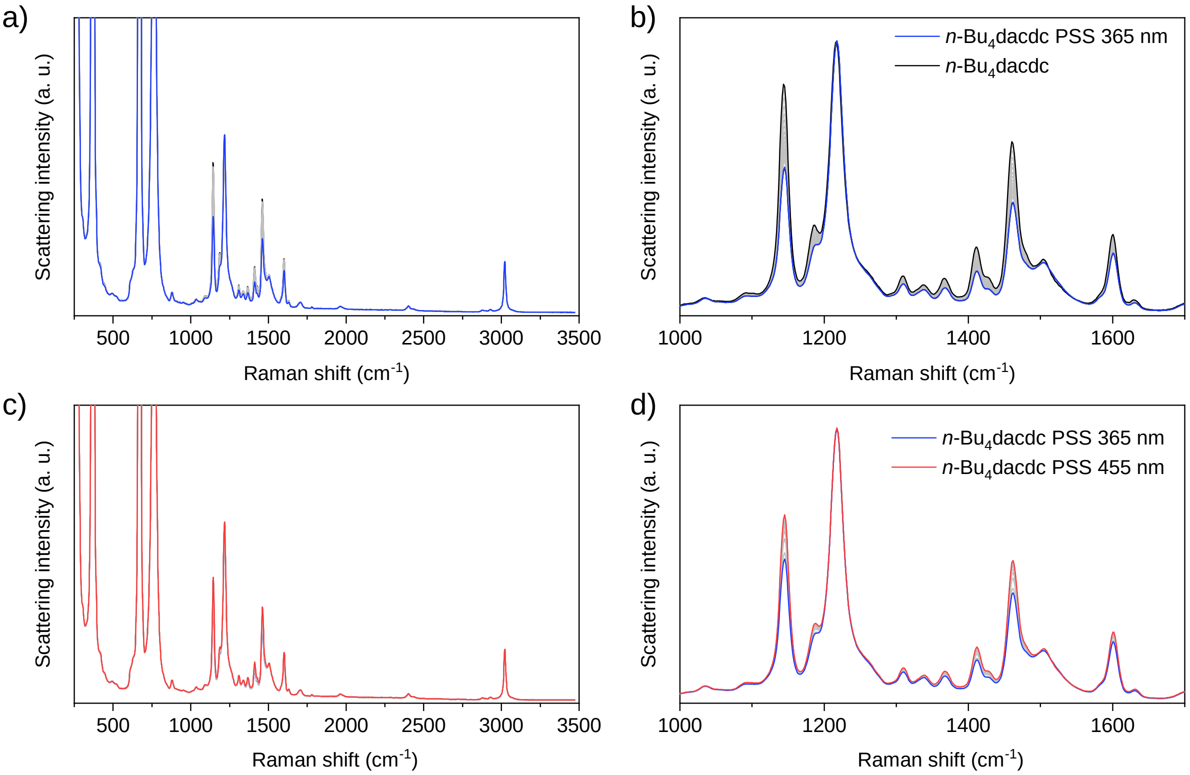

Supplementary Figure 19. a,b) Time-dependent *in situ* Raman spectroscopy of (*E*)-*n*-Bu₄dacdc (black) in $CHCl_3$ during irradiation with 365 nm until state PSS (blue) and c,d) irradiation at 455 nm from PSS-365 nm (blue) to PSS-455 nm (red).

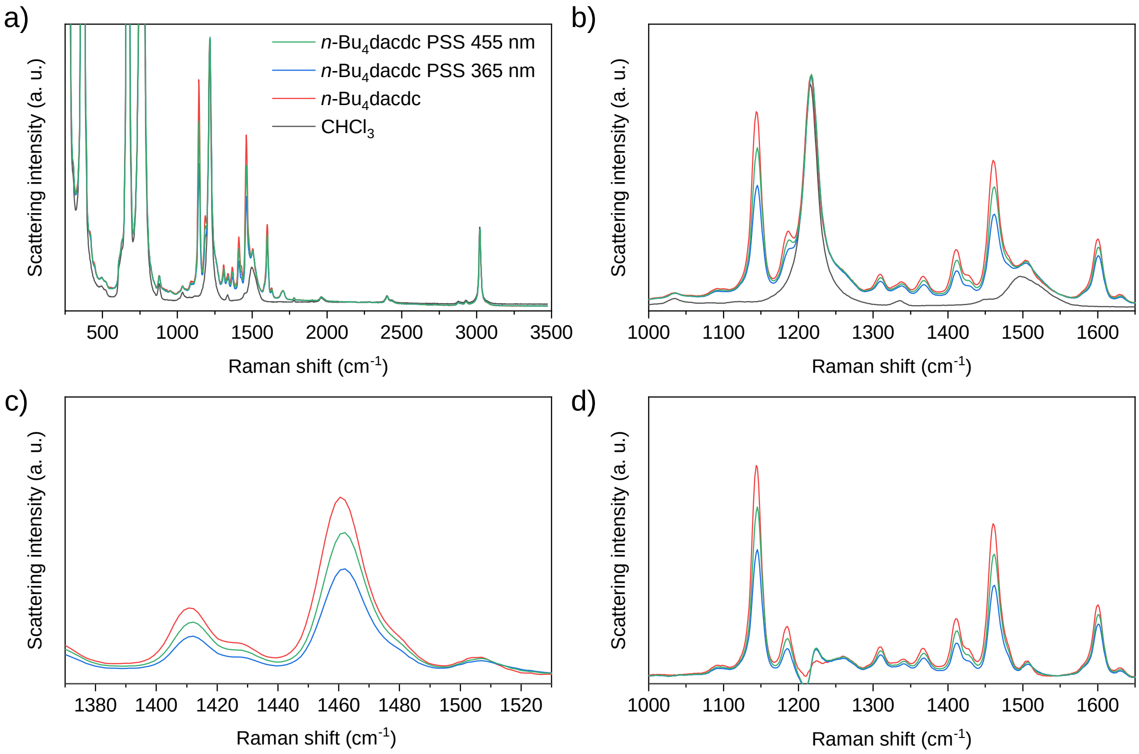

Supplementary Figure 20. In situ Raman spectroscopy of *n*-Bu₄dacdc in $CHCl_3$ during irradiation a,b) Raman spectra of CHCl3(black) (*E*)-*n*-Bu₄dacdc (red), photo stationary state (PSS) of *n*-Bu₄dacdc after irradiation at 365 nm (blue) and 455 nm (green). c,d) Solvent-subtracted spectra.

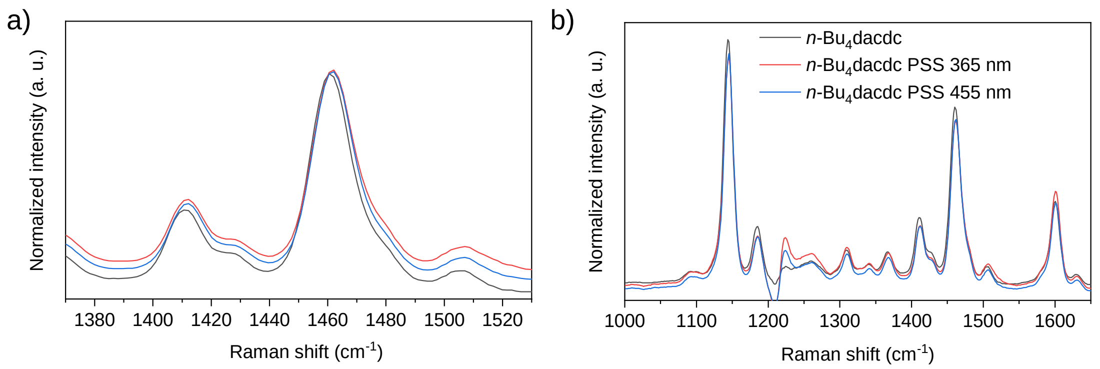

Supplementary Figure 21. a,b) Normalized (to the intensity of 1460 cm^{-1}) Raman spectra of (*E*)-*n*-Bu_4dacdc (black), photo stationary state (PSS) of *n*-Bu_4dacdc after irradiation at 365 nm (red) and 455 nm (blue).

Attenuated total reflection (ATR) Fourier-Transform-Infrared (FTIR) spectroscopy was used to further analyze the isomerization of *n*-Bu_4dacdc. For that small drops of solutions of (*E*)-*n*-Bu_4dacdc (1 mg ml^{-1}) in $CHCl_3$ before and after irradiation at 365 nm were placed on the sample stage of the spectrometer and the solvent was evaporated by a flow of nitrogen at ambient temperature. This way a spectrum of (*E*)-*n*-Bu_4dacdc and (*E*/*Z*)-*n*-Bu_4dacdc at the PSS from irradiation with 365 nm could be obtained, assuming no change in the conformation occurs upon evaporation of the solvent (the *Z*-isomer was found to be thermally stable at 298 K in solution). In two sets of experiments the sample amount was varied by subsequently dropping 0.05 ml and 0.15 ml of the same solution on the sample stage and evaporating the solvent by a flow of nitrogen.

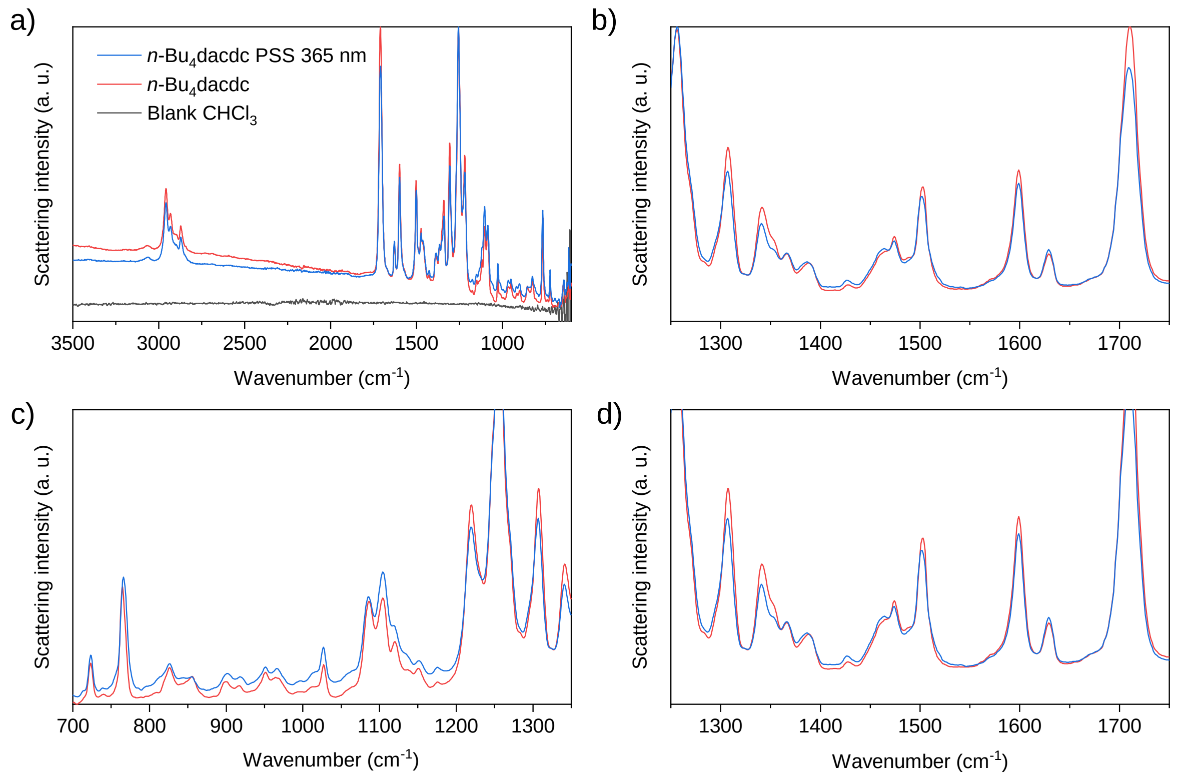

Supplementary Figure 22. a-d) ATR-FTIR spectra of (*E*)-*n*-Bu_4dacdc before (red) and after (blue) irradiation at 365 nm with a reference of evaporated $CHCl_3$ (black) of 0.05 ml sample amount. b,c,d) Magnification of different regions of the spectrum.

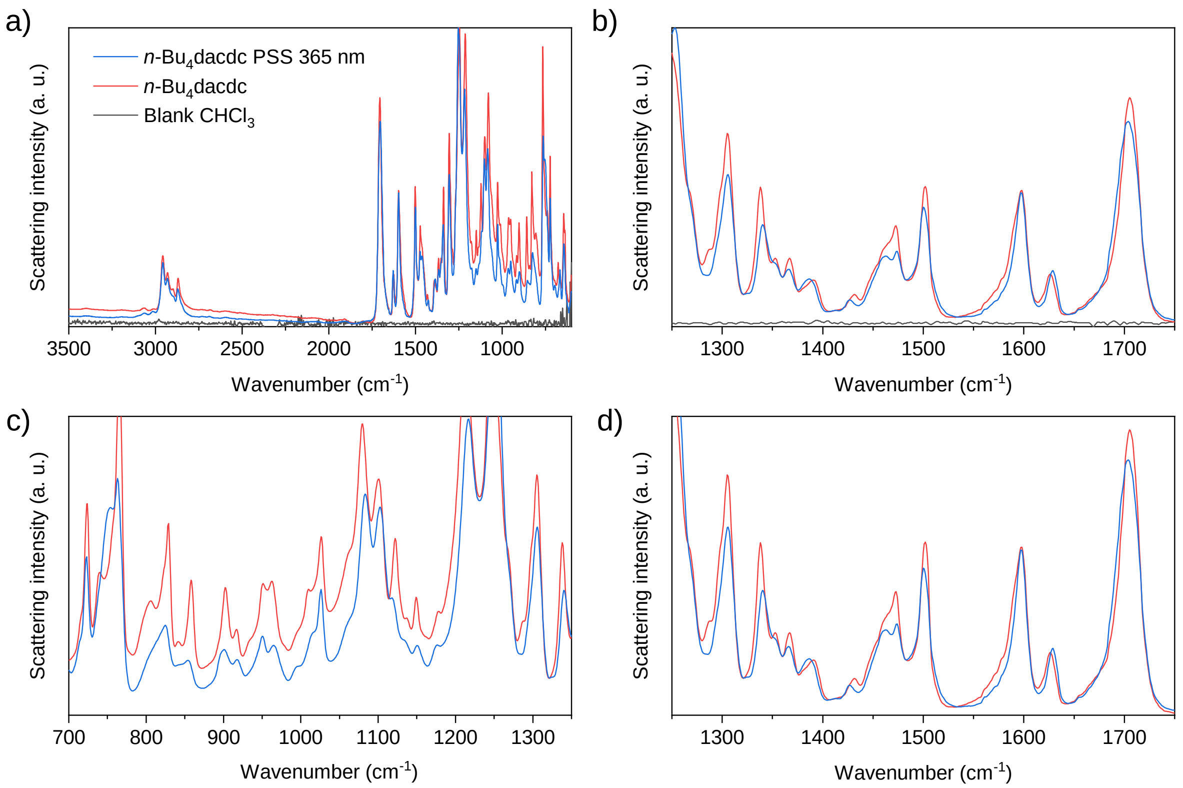

Supplementary Figure 23. a-d) ATR-FTIR spectra of (*E*)-*n*-Bu4dacdc before (red) and after (blue) irradiation at 365 nm with a reference of evaporated $CHCl_3$ (black) 0.15 ml sample amount. b,c,d) Magnification of different regions of the spectrum.

3) Synthesis of metal-organic framework DUT-163

Synthesis of DUT-163 (Dresden University of Technology No. 163) powder was conducted via solvothermal reaction of (*E*)-H_4L with $Cu(NO_3)_2 \cdot 3H_2O$ in DMF. In a round bottom flask 400 mg (0.58 mmol) (*E*)-H_4L were dissolved in 85 ml DMF and 3 ml acetic acid using an ultrasonic bath. To the yellow solution 351 mg (1.45 mmol) $Cu(NO_3)_2 \cdot 3H_2O$ were added and dissolved, the flask was closed and the reaction mixture stirred for 48 h at 80 °C. Afterwards the MOF powders were separated from the mother liquid via centrifugation and washed with fresh DMF for at least three times over three days.

To yield crystals large enough for single crystal analysis the synthesis procedure was modified. In a Pyrex tube 10 mg (0.014 mmol) (*E*)-H_4L were dissolved in 4 ml DMF and 0.3 ml acetic acid using an ultrasonic bath. To the yellow solution 9.7 (0.035 mmol) $Cu(NO_3)_2 \cdot 3H_2O$ were added and the tube was sealed and heated at 80 °C for 3 d to yield light green cuboctahedral crystals approximately 80 µm in diameter.

4) Supercritical activation of MOF powder

Drying (solvent removal from the pores) of DUT-163 powder was conducted using a protocol based on previous reports[1,4]: After synthesis the MOF powder suspended in fresh DMF was exchanged at least 6 times over a period of at least three days with anhydr. acetone. The material was dried using a protocol involving liquid/supercritical CO_2. The acetone suspended MOF powder was placed in glass filter frit in a Jumbo Critical Point Dryer 13200J AB (SPI Supplies) which was subsequently filled with liquid CO_2 (99.995% purity) at 288 K and 5 MPa. To ensure a complete substitution of the acetone by CO_2, the liquid in the autoclave was exchanged with fresh CO_2 at least 18 times over a period of 5 days using a valve at the bottom of the autoclave. The temperature and pressure were then risen beyond the supercritical point of CO_2 to 308 K and 10 MPa and kept until the temperature and pressure was stable. The supercritical CO_2 was steadily released over 3 h and

the dry brown powder was transferred and stored in an argon filled glove box under exclusion of light. To ensure complete removal of the solvent (especially from the open metal sites of the Cu-paddle-wheels) additional activation at 353 K in a Schlenk-tube under dynamic vacuum of 10^{-4} kPa for at least 24 h was performed.

5) Single crystal X-ray diffraction

As synthesized single crystal of DUT-163 with linear dimensions of 20 x 20 x 20 *µm* was prepared in a borosilicate glass capillary (d = 0.3 mm) with small amount of DMF, which was sealed with was afterwards. The dataset was collected at BESSY MX BL14.3 beamline of Helmholtz-Zentrum Berlin für Materialien und Energie[5] at 296 K. After short test scans, the crystal symmetry and scan range were determined in each particular case using iMosflm program[6]. The φ-scans with oscillation step of 0.5° were used for data collection. The dataset was processed automatically using XDSAPP 2.0 software[7]. Crystal structures were solved by direct methods and refined by full matrix least-squares on F^2 using SHELX-2014/7 program package[8]. All non-hydrogen atoms were refined in anisotropic approximation. Hydrogen atoms were refined in geometrically calculated positions using “riding model” with $U_{iso}(H)$=1.2$U_{iso}(C)$. During the refinement of the crystal structure, the disorder of the phenyl rings over two positions and disorder of the nitrogen atoms of azo group over four positions have been detected and treated in the refinement using corresponding distance restraints of 1.45(1) and 1.30(1) for C11-N2 and N2-N2$^{\#1}$ ($^{\#1}$ - 1.5-x, 1.5-y, 1-z). Disordered guest molecules could not be refined unambiguously from the difference Fourier map, hence, SQUEEZE routine in PLATON was used to generate the reflection intensities with subtracted solvent contribution[9]. Experimental data for DUT-163: $C_{40}H_{20}Cu_2N_4O_8(H_2O)_2$, F_w = 847.73 g mol^{-1}, cubic, $Fm\bar{3}m$ (No 225), a = 49.240(6) Å, V = 119386(41) Å^3, Z = 24, D_c = 0.282 g cm^{-3}, μ = 0.420 mm^{-1}, $F(000)$ = 10224, θ_{max} = 32.479°, reflections collected / unique: 57918 / 5606, R_{int} = 0.1415, 106 parameter refined, 2 restraints, $R1$ $(I > 2\sigma)$ = 0.0603, $wR2$ *(all data)* = 0.2251, S *(all data)* = 1.004, max / min residual electron density 0.717 / -0.338.

CCDC-2040810 contains the supplementary crystallographic data for DUT-163*op*. These data can be obtained free of charge from the Cambridge Crystallographic Data Centre via www.ccdc.cam.ac.uk/data_request/cif.

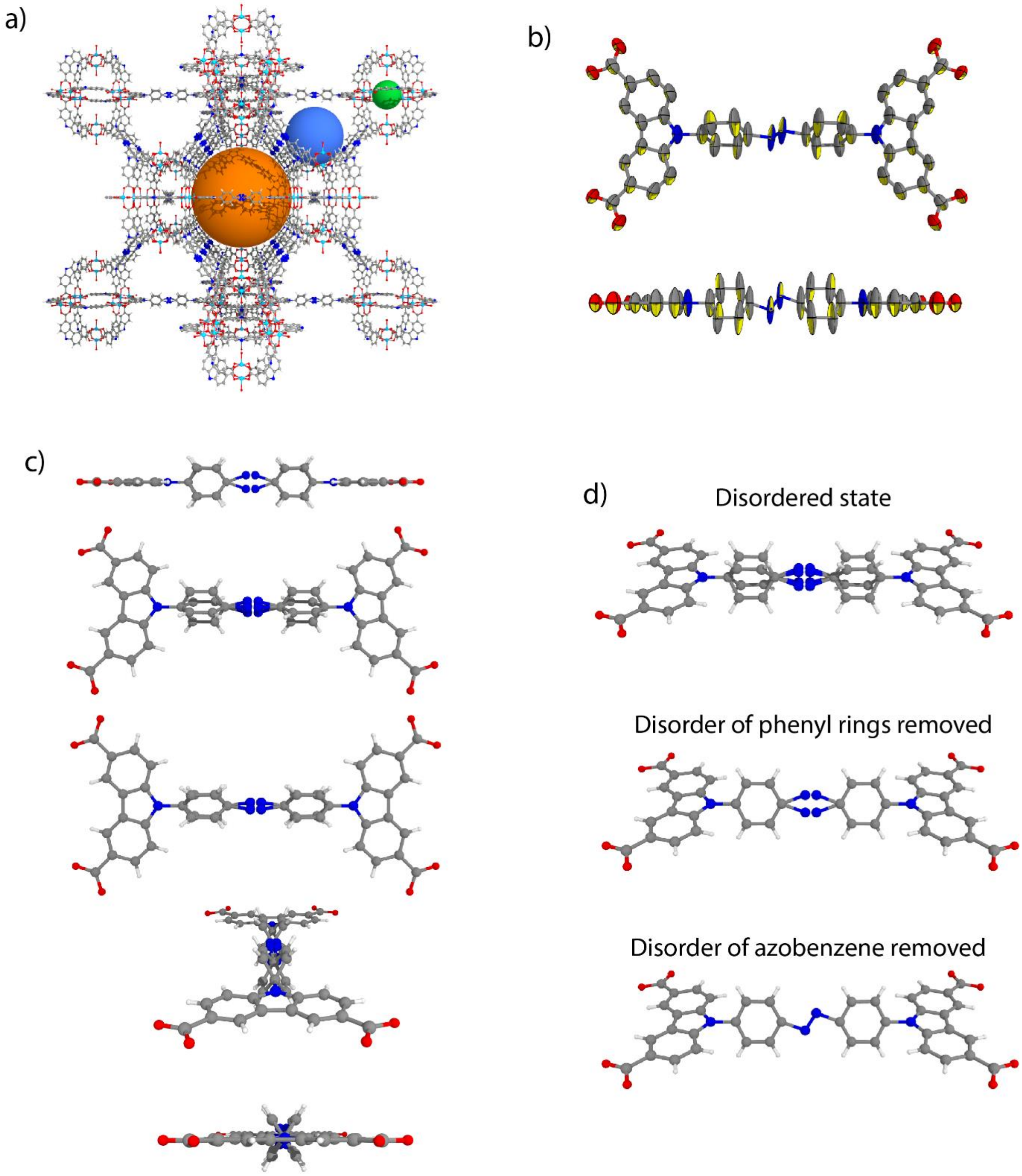

Supplementary Figure 24. a) Crystal structure of DUT-163 including the three different types of pores represented by orange, blue and green spheres. b) A singe conformation with removed disorder showing thermal ellipsoids with occupation probability of 50%, c) Disorder-containing ligand in different orientations, d) dacdc shown in stepwise removal of symmetry equivalent disorder.

6) Powder X-ray diffraction (PXRD) of MOF samples

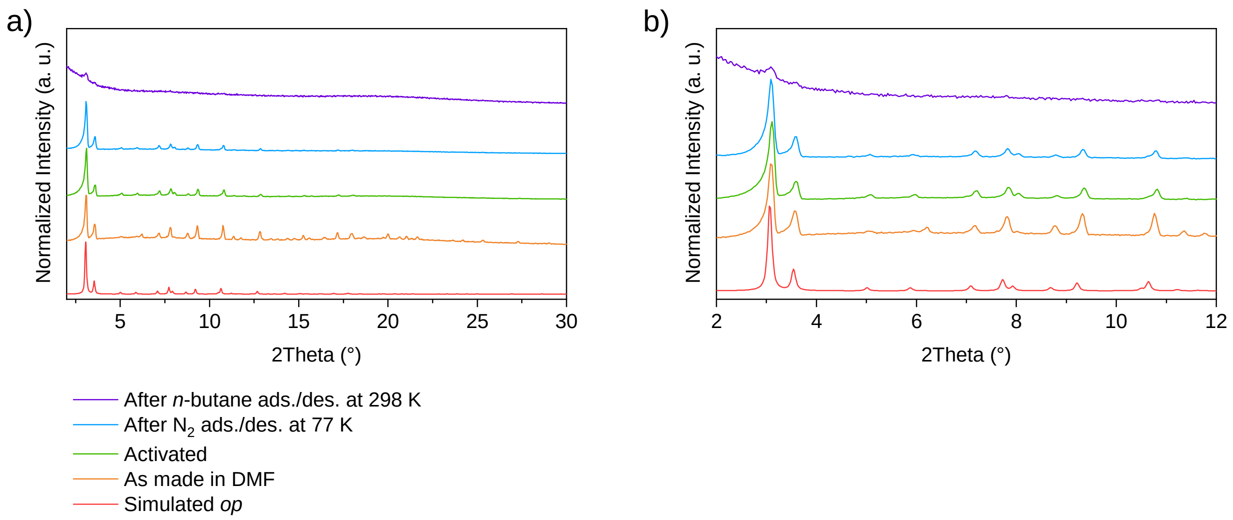

Supplementary Figure 25. PXRD patterns of DUT-163 under different conditions. Right panel represent magnified small angle region.

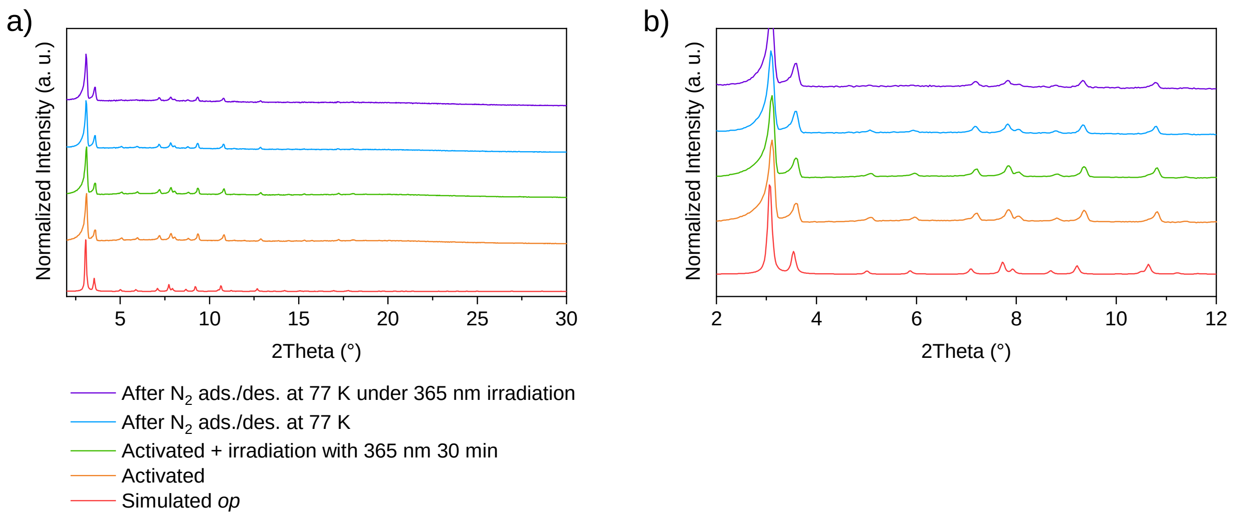

Supplementary Figure 26. PXRD patterns of DUT-163 with and without irradiation. Right panel represent magnified small angle region.

7) Elemental analysis of MOF sample

Desolvated samples of DUT-163 were placed in Sn-sample holders and sealed under inert atmosphere in an Ar-filled glovebox.

Elemental analysis for DUT-163 $Cu_2C_{40}H_{20}N_4O_8$: Calculated: C: 59.19%; H: 2.48%; N: 6.9%; found: C: 59.39%; H: 2.84%; N: 6.81%.

8) Thermogravimetric analysis of MOF samples

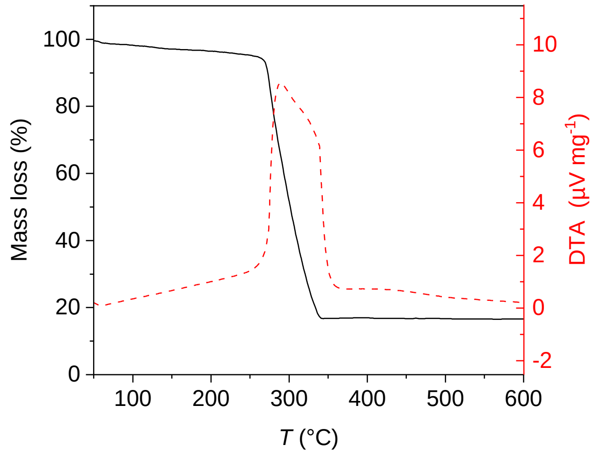

Supplementary Figure 27. Thermogravimetric analysis of guest free DUT-163 activated by supercritical carbon dioxide.

9) Scanning electron microscopy of MOF samples

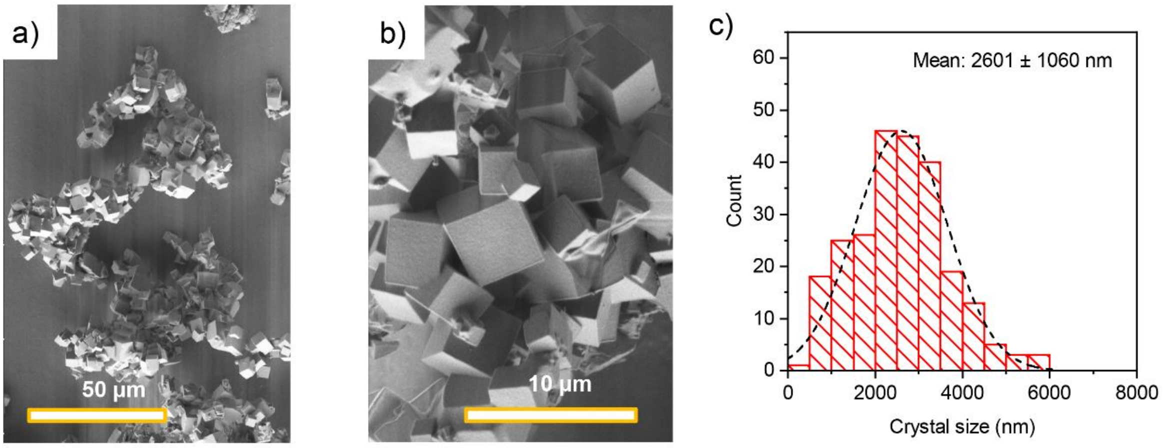

Supplementary Figure 28. a,b) Scanning electron microscopy images of DUT-163, c) Experimental crystal size distribution (red histogram) and distribution curve (black dashed line).

10) Gas adsorption experiments

For the conduced adsorption and in situ experiments various gases and vapours were chose. Nitrogen at 77 K is a classical adsorptive to probe porosity. Methane in the rane of 110-120 K and n-butane at 273-303 K are adsorptives heavily used to investigate NGA in DUT-49-type materials and thus allows to draw comparisons with other materials. MP was chosen because the upper temperature limit for framework contraction without irradiation is around ambient temperature, which makes it a probe easy to apply in near ambient experiments. CCl_4 vapor was chosen as a probe with low IR activity for DRIFT analysis.

Supplementary Table 1. Adsorption conditions used in this study (Vapor pressures were obtained from NIST Chemistry WebBook, SRD 69 https://webbook.nist.gov/chemistry/fluid/)

Gas	*T* (K)	Vapor pressure, p_0 (kPa)	Figure Adsorption isotherm
Nitrogen	77.2	99.8	Supplementary Figure 29
Methane	111,115,120	95.8, 132.2, 191,4	Supplementary Figure 33
MP	261	99.7	Supplementary Figure 42
MP	293	301	Supplementary Figure 41
MP	295	319	Supplementary Figure 40
MP	296	329	Simulated Isotherm/*in situ* PXRD
MP	297	339	Supplementary Figure 39
MP	299	359	Supplementary Figure 38
MP	300	370	Simulated Isotherm/*in situ* PXRD
MP	301	380	Supplementary Figure 37
MP	303	403	Supplementary Figure 36
MP	305	426	Supplementary Figure 35
MP	307	448	Supplementary Figure 34
	330		
n-Butane	298	242	Supplementary Figure 33
n-Butane	303	282	Supplementary Figure 33
CCl_4	298	13.5	Supplementary Figure 33

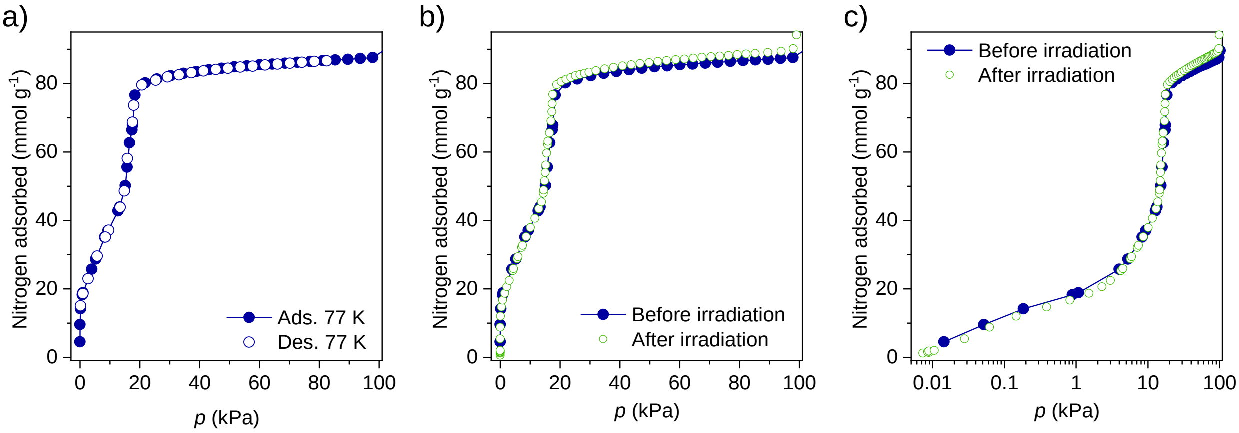

Supplementary Figure 29. a) Nitrogen physisorption isotherms of DUT-163 at 77 K (adsorption and desorption in filled and empty symbols, respectively), b,c) Nitrogen physisorption isotherms of DUT-163 at 77 K before (blue) and after (green) irradiation of the sample with 365 nm for 1 h using a CONSORT UV-lamp with 1800 µW/cm² (sample area ca. 2 cm², sample thickness ca. 1-2 mm). Irradiation was not performed in parallel to adsorption.

An experimental specific pore volume of V_p = 2.84 cm³ g⁻¹ was determined by nitrogen adsorption at 77 K at a relative pressure p/p_0 of 0.98 (p=97 kPa) using the Belsorp Max software package.

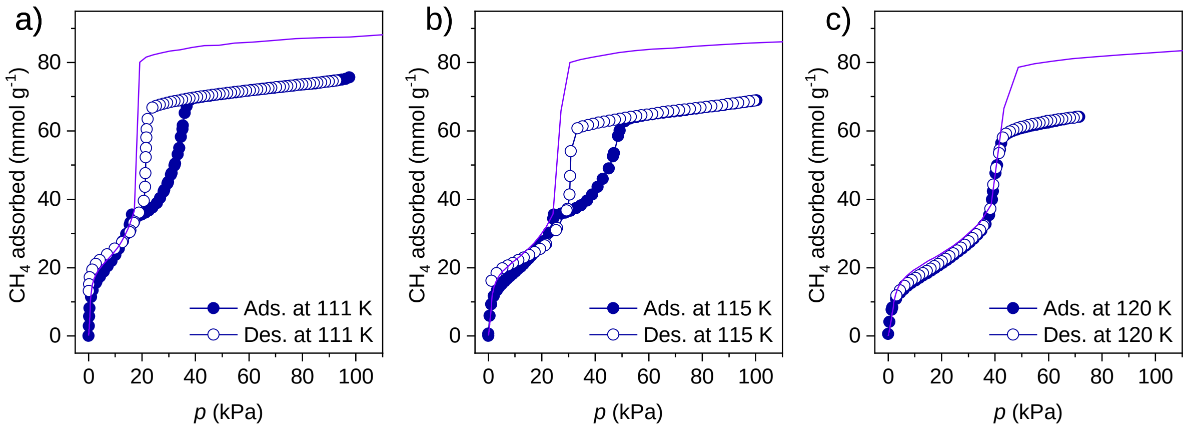

Supplementary Figure 30. a-c) Methane adsorption isotherms of DUT-163 at 111 K (a), 115 K (b), and 120 K (c). comparison to simulated isotherms of DUT-163*op* (purple). Adsorption and desorption in filled and empty symbols, respectively

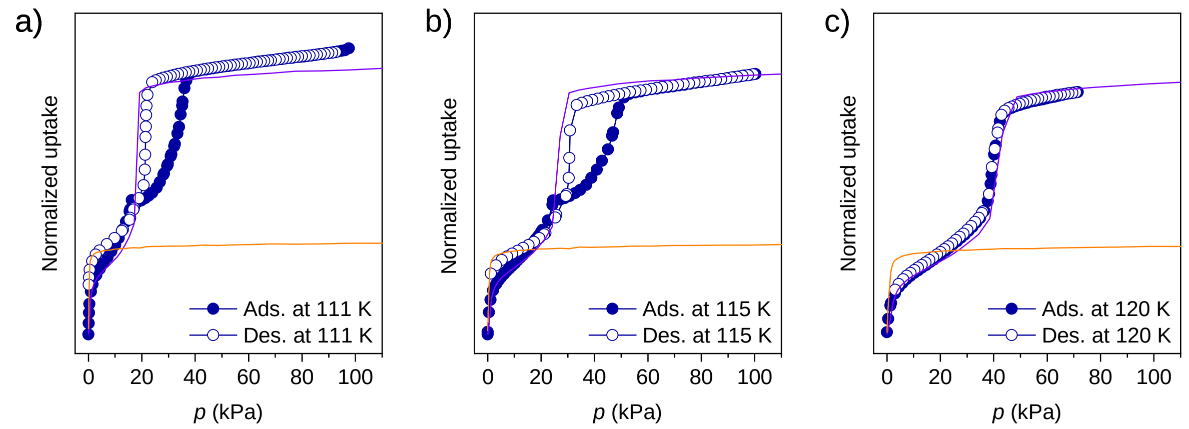

Supplementary Figure 31. a-c) Methane adsorption isotherms of DUT-163 at 111 K (a), 115 K (b), and 120 K (c). normalized experimental adsorption isotherm of a) in comparison to simulated isotherms of DUT-163*op* (purple) and DUT-163*cp* (orange) (Normalization to the uptake of the simulated isotherm by reduction of 22% in accordance to the previously mentioned deviation and in line with the other adsorption experiments).) Adsorption and desorption in filled and empty symbols, respectively.

Cyclability of the adsorption process was demonstrated by applying a routine previously established for DUT-49 and related materials[1]. In the case of DUT-49 and DUT-163, if adsorption-induced contraction occurs during the adsorption process it was likewise found to occur during the desorption process. Interestingly, under these conditions no reopening of the framework was observed upon desorption. However, the framework can be expanded again by readsorbing gas up to the saturation pressure. To desorb gas without structural contraction occuring one simply has to elevate the temperature beyond the temperature at which adsorption contraction no longer occurs. In the case of DUT-163 this temperature was found to be beyond 122 K upon methane adsorption. Isotherms in Supplementary Figure 32 were recorded by sequential methan adsorption. The first cycle was conducted by adsorbing and desorbing methane at 115K. The sample was then pressurerized again up to 110 kPa and the Temperature was elevated to 130 K. The pressure was subsequently released and the sample evacuated for 1 h under dynamic vacuum. The sample was then reexamined by recording a second adsorption isotherm which demonstrates the same hysteresis, NGA features and shows only slight loss in uptake.

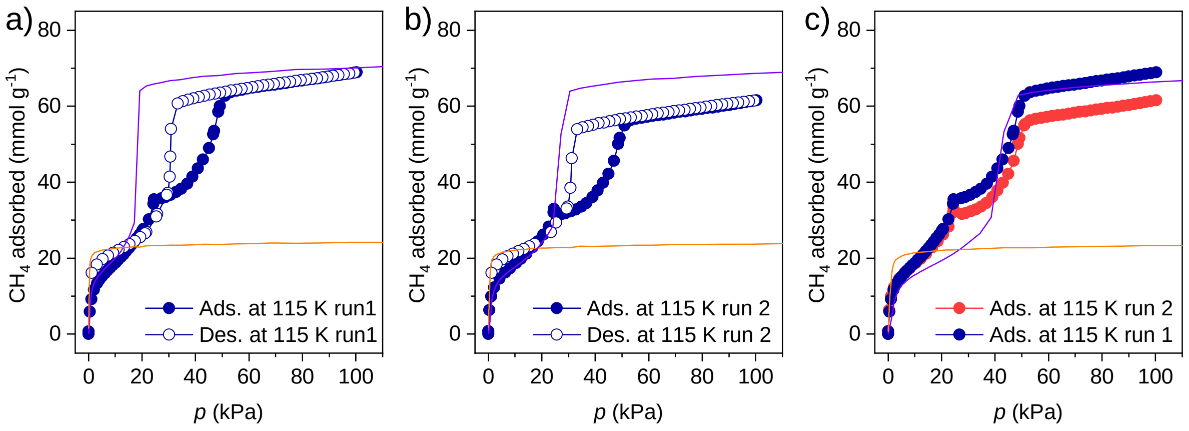

Supplementary Figure 32. a-c) Methane adsorption isotherms of DUT-163 at 115 K first cycle (a), 115 K after recycling (b), and direct comparison of both cylces. Simulated isotherms of DUT-163*op* (purple) and DUT-163*cp* (orange) (uptake of simulated isotherm reduced by 22% in accordance to the previously mentioned deviation and in line with the other adsorption experiments). Adsorption and desorption in filled and empty symbols, respectively.

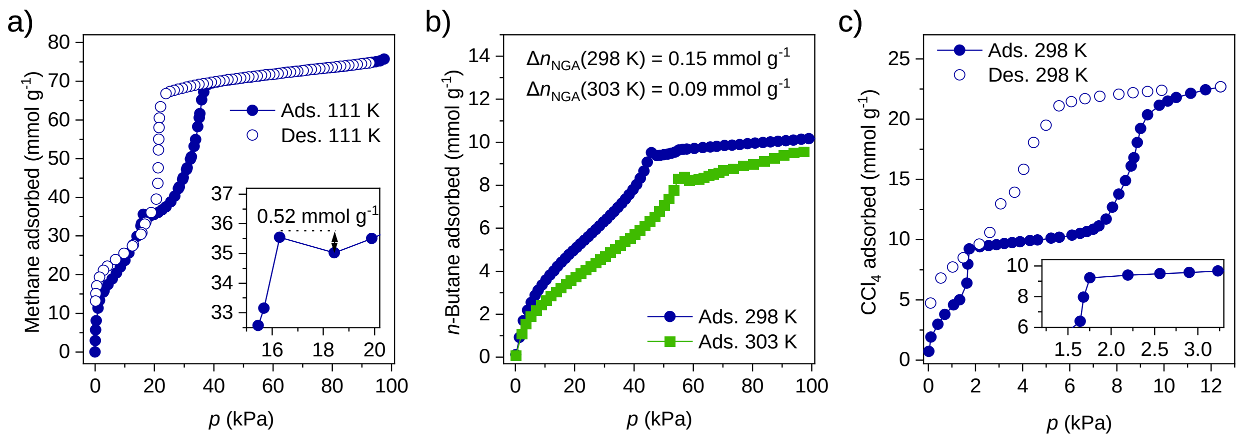

Supplementary Figure 33. Physisorption isotherms of DUT-163 for adsorption of a) methane at 111 K, b) *n*-butane at 298 K (blue) and 303 K (green), and CCl4 at 298 K. Adsorption and desorption in filled and empty symbols, respectively. Inset in a) and c) shows transition region and corresponding Δn_{NGA} values.

Detailed information on how NGA impacts the adsorption measurement and to what extend the gas release can be quantified is accessible from reference [10,11]. Additional information on NGA as a function of adsorption of different gases at different temperatures can be obtained from reference [12].

Although the shape of experimental and computational (based on perfect infinite crystals) adsorption isotherms matches well, the excess adsorbed amount is found to deviate by a constant factor of ca. 22% throughout all adsorption experiments and the whole pressure range. This effect was previously observed for DUT-49 in samples with reduced crystal size and related to enhanced surface effects/reduction in internal pore volume in small crystallites below around 1 µm[13] and may also be caused by partial pore collapse upon solvent removal previously observed for DUT-49 solids with reduced mechanical stability.[14] To better illustrate the adsorption-induced contraction and its effect on the adsorption behavior independent of these effects, isotherms in Supplementary Figure 34-Supplementary Figure 42 d) and in Figure 4 in the main text are given in non-numerical scale with adsorbed amount of experimental isotherms normalized to the simulated value in the relative pressure range of <0.15 which is not effected by structural transitions.

To investigate the effect of irradiation on the adsorption and transition behavior of DUT-163 ca. 10mg of powdered sample was placed in a special quartz sample cell with 2 mm diameter and connected to the regular

BELSORP-max instrument. In a second port another DUT-163 sample of comparable mass was placed in a regular sample cell and covered with aluminum foil to establish light exclusion. Both cells were placed in a water-filled quartz-glass beaker that was connected to a JULABO thermostat. Temperature of the bulk coolant was monitored and stabilized throughout the whole adsorption experiment. The quartz cell was continuously irradiated with a nominal wavelength of 365 nm throughout the whole experiment (including dead volume measurement) using a CONSORT UV-lamp with 1800 µW/cm² that was placed 5 cm away from the sample cell outside the water filled beaker. Irradiated and non-irradiated samples were analyzed in parallel using the BELSORP software setup for parallel sample analysis. A series of experiments with MP at different temperatures were performed on the same sample starting with adsorption at 307 K. After adsorption/desorption experiments at a given temperature the samples were evacuated in dynamic vacuum (<10-5 kPa) for 30 min during which the light source was switched off. The temperature was set to a new value and equilibrated before the next adsorption experiment was conducted following the same protocol. Detailed desorption branches were only recorded in a few of these experiments.

The experimental V_p of 2.84 cm^3 g^{-1} determined by nitrogen adsorption at 77 K shows a 18% deviation compared to the theoretical value which might be caused by crystal size effects or partial pore collapse upon solvent removal of the bulk powder previously observed in DUT-49.[13] Throughout the whole temperature range the shape of experimental and simulated isotherms shows good agreement, however a difference in adsorbed amount of 18-22% is observed. This deviation is in line with the difference of experimental and simulated V_p.

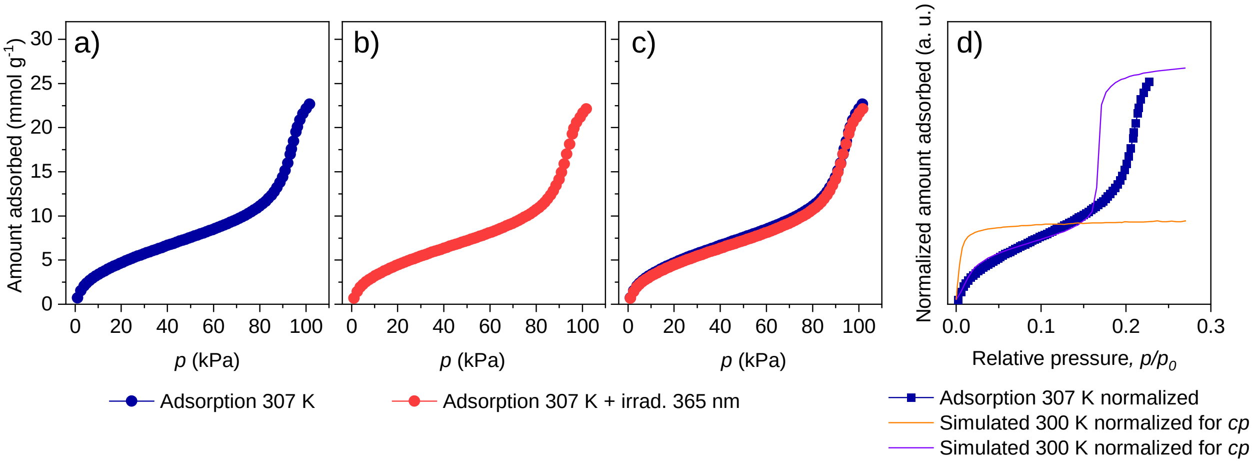

Supplementary Figure 34. MP physisorption isotherms of DUT-163 at 307 K a) under exclusion of light, b) under continuous irradiation with nominal wavelength of 365 nm, c) overlap of adsorption branches of a) (blue) and b) (blue), and d) normalized experimental adsorption isotherm of a) in comparison to simulated isotherms of DUT-163*op* (purple) and DUT-163*cp* (orange) (Normalization to the uptake of the simulated isotherm of the *op* phase in the relative pressure range <0.15) given in relative pressure. Adsorption and desorption in filled and empty symbols, respectively.

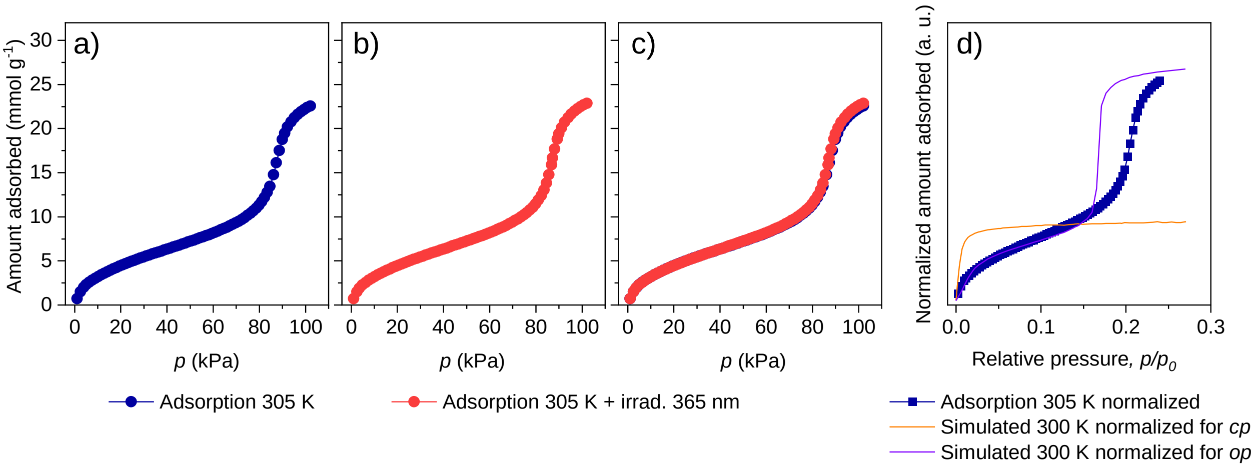

Supplementary Figure 35. MP physisorption isotherms of DUT-163 at 305 K a) under exclusion of light, b) under continuous irradiation with nominal wavelength of 365 nm, c) overlap of adsorption branches of a) (blue) and b) (blue), and d) normalized experimental adsorption isotherm of a) in comparison to simulated isotherms of DUT-163*op* (purple) and DUT-163*cp* (orange) (Normalization to the uptake of the simulated isotherm of the *op* phase in the relative pressure range <0.15) given in relative pressure. Adsorption and desorption in filled and empty symbols, respectively.

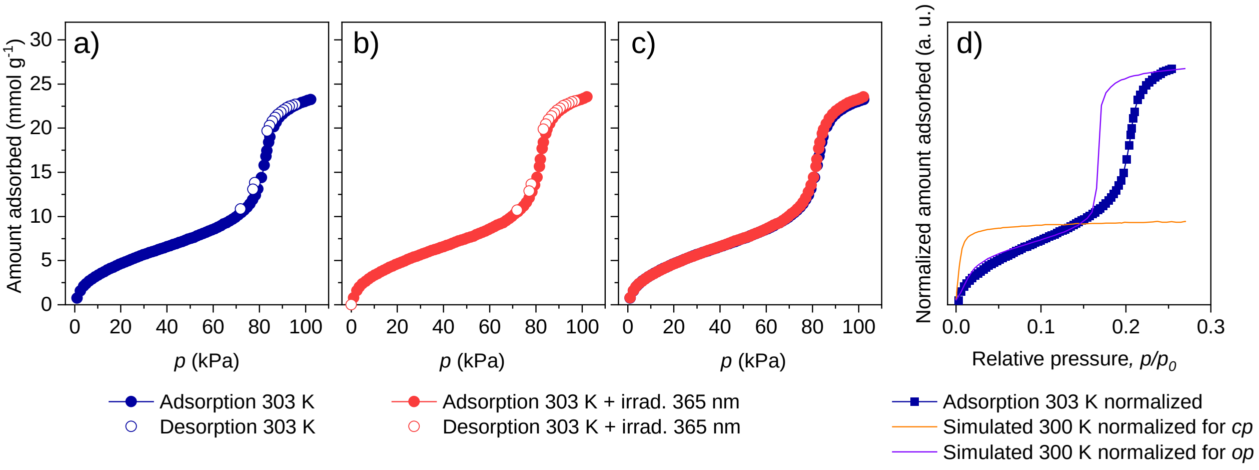

Supplementary Figure 36. MP physisorption isotherms of DUT-163 at 303 K a) under exclusion of light, b) under continuous irradiation with nominal wavelength of 365 nm, c) overlap of adsorption branches of a) (blue) and b) (blue), and d) normalized experimental adsorption isotherm of a) in comparison to simulated isotherms of DUT-163*op* (purple) and DUT-163*cp* (orange) (Normalization to the uptake of the simulated isotherm of the *op* phase in the relative pressure range <0.15) given in relative pressure. Adsorption and desorption in filled and empty symbols, respectively.

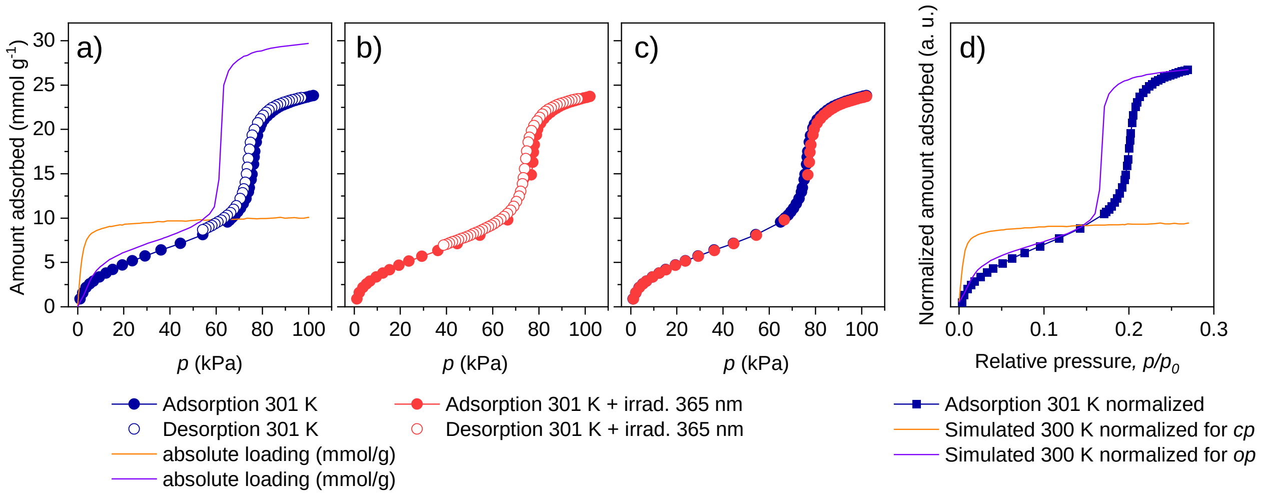

Supplementary Figure 37. MP physisorption isotherms of DUT-163 at 301 K a) under exclusion of light, b) under continuous irradiation with nominal wavelength of 365 nm, c) overlap of adsorption branches of a) (blue) and b) (blue), and d) normalized experimental adsorption isotherm of a) in comparison to simulated isotherms of DUT-163*op* (purple) and DUT-163*cp* (orange) (Normalization to the uptake of the simulated isotherm of the *op* phase in the relative pressure range <0.15) given in relative pressure. Adsorption and desorption in filled and empty symbols, respectively.

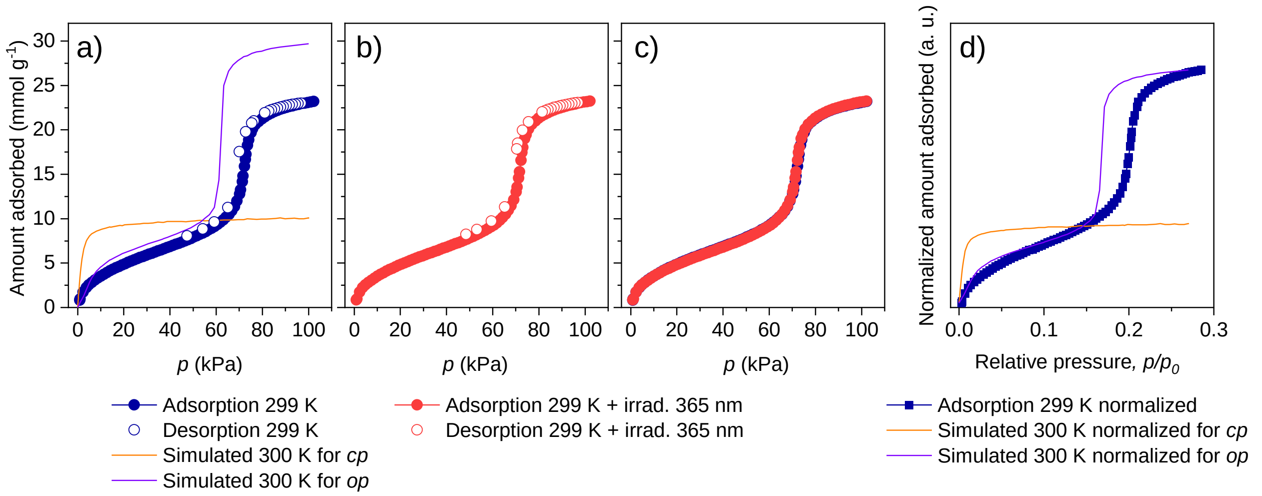

Supplementary Figure 38. MP physisorption isotherms of DUT-163 at 299 K a) under exclusion of light, b) under continuous irradiation with nominal wavelength of 365 nm, c) overlap of adsorption branches of a) (blue) and b) (blue), and d) normalized experimental adsorption isotherm of a) in comparison to simulated isotherms of DUT-163*op* (purple) and DUT-163*cp* (orange) (Normalization to the uptake of the simulated isotherm of the *op* phase in the relative pressure range <0.15) given in relative pressure. Adsorption and desorption in filled and empty symbols, respectively.

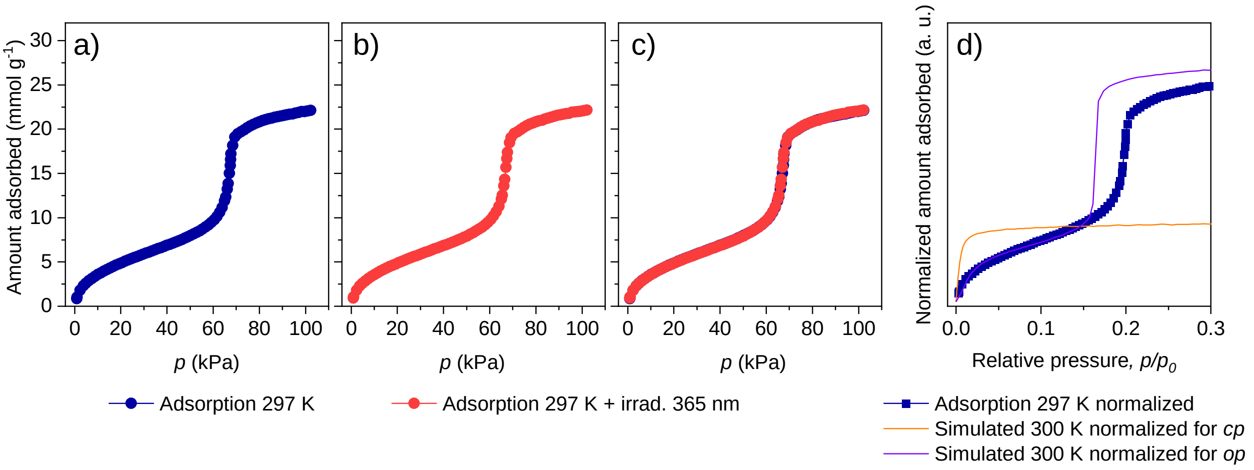

Supplementary Figure 39. MP adsorption isotherms of DUT-163 at 297 K a) under exclusion of light, b) under continuous irradiation with nominal wavelength of 365 nm, c) overlap of adsorption branches of a) (blue) and b) (blue), and d) normalized experimental adsorption isotherm of a) in comparison to simulated isotherms of DUT-163*op* (purple) and DUT-163*cp* (orange) (Normalization to the uptake of the simulated isotherm of the *op* phase in the relative pressure range <0.15) given in relative pressure.In this experiment a desorption branch was not recorded due to an instrument failure. The desorption process was conducted manually following the instrument protocol.

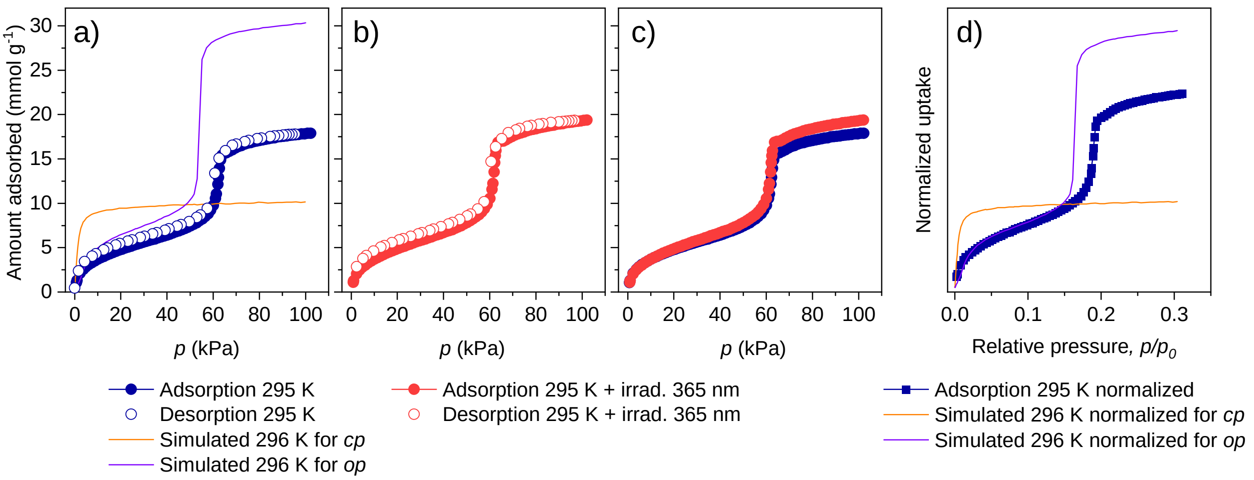

Supplementary Figure 40. MP physisorption isotherms of DUT-163 at 295 K a) under exclusion of light, b) under continuous irradiation with nominal wavelength of 365 nm, c) overlap of adsorption branches of a) (blue) and b) (blue), and d) normalized experimental adsorption isotherm of a) in comparison to simulated isotherms of DUT-163*op* (purple) and DUT-163*cp* (orange) (Normalization to the uptake of the simulated isotherm of the *op* phase in the relative pressure range <0.15) given in relative pressure. Adsorption and desorption in filled and empty symbols, respectively.

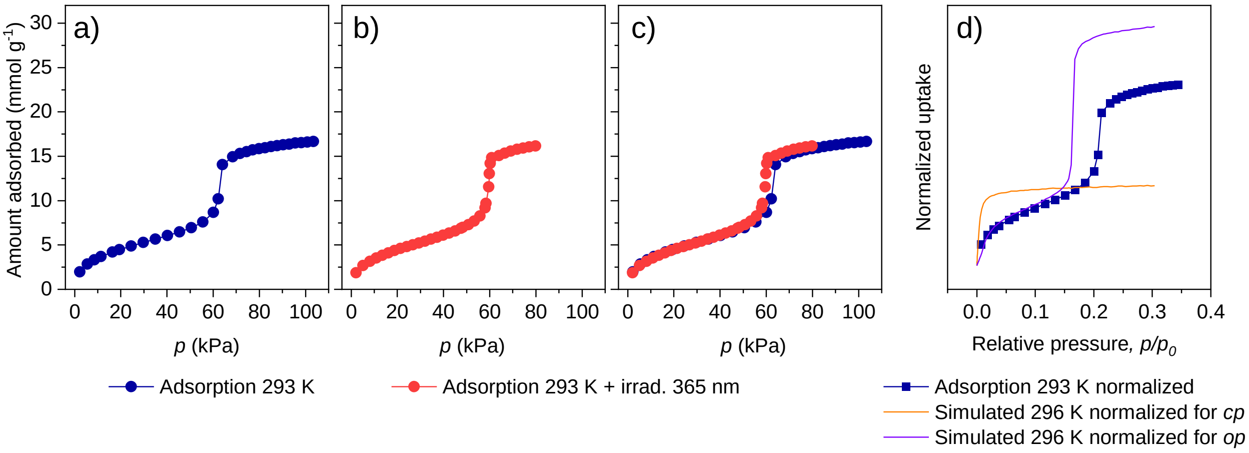

Supplementary Figure 41. MP physisorption isotherms of DUT-163 at 293 K a) under exclusion of light, b) under continuous irradiation with nominal wavelength of 365 nm, c) overlap of adsorption branches of a) (blue) and b) (blue), and d) normalized experimental adsorption isotherm of a) in comparison to simulated isotherms of DUT-163*op* (purple) and DUT-163*cp* (orange) (Normalization to the uptake of the simulated isotherm of the *op* phase in the relative pressure range <0.15) given in relative pressure. Adsorption and desorption in filled and empty symbols, respectively.

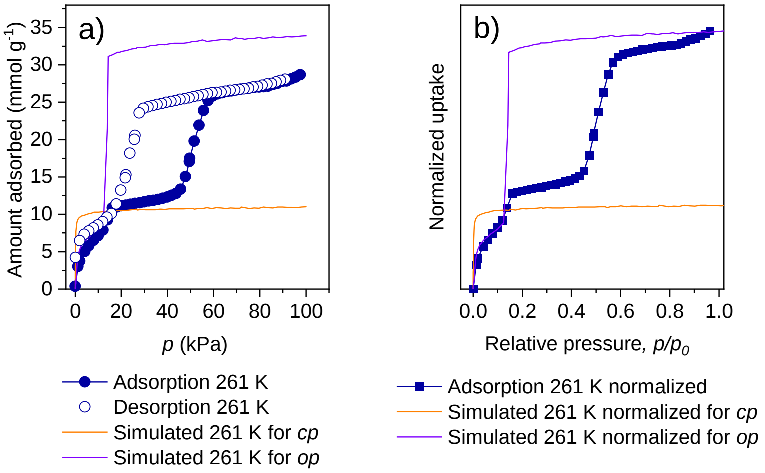

Supplementary Figure 42. MP physisorption isotherms of DUT-163 at 261 K under exclusion of light and b) normalized experimental adsorption isotherm of a) in comparison to simulated isotherms of DUT-163*op* (purple) and DUT-163*cp* (orange) (Normalization to the uptake of the simulated isotherm of the *op* phase in the relative pressure range <0.15) given in relative pressure. Adsorption and desorption in filled and empty symbols, respectively.

Adsorbed amounts in mmol g^{-1} can be transferred into units of molecules per unit cell by multiplying the adsorbed amount in mmol g^{-1} with the molar mass of one unit cell in g $mmol^{-1}$. For DUT-163 ($C_{40}H_{20}Cu_2N_4O_8$, 811.70 g mol^{-1} per formular unit, Z = 24) this is 19.48°g $mmol_{UC}^{-1}$.

The deviation of simulated isotherms for the op phase to experimental adsorption isotherms occurs in three different ways. First, we observe a general deviation in adsorbed amount by ca. 22% reduction in the experimental data. This is observed throughout all sets of experiments/simulations. We associate this to

factors such as crystal size which has been reported to cause changes in the gas capacity of real-world materials in comparison to simulations conduced on “perfect” crystal structures.

The second deviation is caused by structural transitions in the solid. As the op-cp transitions in DUT-49-type materials are known to occur upon mesopore filling (see https://www.nature.com/articles/s41467-019-11565-3) the pressure range in which the cp phase is present is quite defined (ca. p/p0 0.16 for DUT-163). Strong deviation in adsorbed amount in this pressure range (such as the deviations observed in the temperature range of 295 K and below for MP adsorption or 115 K upon methane adsorption) are consequently a result of structural contraction. These have been identified by *in situ* PXRD and are known to occur upon MP adsorption in the temperature range of 296 K and below. Hysteresis in MP isotherms in the temperature range of 299-293 K are not visible since the absolute pressure reached in these experiments is limited to 100 kPa and thus is not able to reach the gate opening relative pressure of 0.5 and above at these temperatures.

The third type of deviation observed between the simulated and experimental data originates from the lack of accurate description of the experimental adsorption process in comparison to the experiment and is found to occur specifically in the range of mesopore filling. It is well known that adsorption isotherms of mesoporous materials exhibit hysteresis and that capillary condensation (a kinetic phenomenon during the adsorption process) results in a shift of the mesopore filling during adsorption towards higher pressure, reflected by a steep increase in uptake in a narrow pressure range. The GCMC simulations conducted in this work and predominantly in most other studies do not capture these kinetic effects. We have observed this kind of deviation of simulation from experiment in isotherms of DUT-49 materials in particular for the adsorption of long chain alkanes such as n-butane (see https://www.nature.com/articles/s41467-019-11565-3 , https://pubs.rsc.org/en/content/articlelanding/2020/sc/d0sc03727c#!divAbstract)

11) In situ PXRD analysis under exclusion of light.

To characterize adsorption induced structural transitions of DUT-163 we used two different *in situ*-powder X-ray diffraction setups at KMC-2 beamline of the BESSY II synchrotron[15], operated by Helmholtz-Zentrum Berlin für Materialien und Energie. The first experimental setup allows to expose the MOF sample under defined pressures of non-corrosive gases up to 100 kPa at defined temperatures in the range of 300-15 K while recording PXRD patterns of the absorbent sample in parallel. This instrumentation is based on the volumetric adsorption instrument BELSORP-max and a closed-cycle Helium cryostat, equipped with an adsorption chamber with beryllium domes[16]. It was used to investigate structural transitions of DUT-163 upon adsorption of *n*-butane at 298 K and MP at 261 K. PXRD patterns were measured at constant wavelength λ = 0.15406 nm (E = 8048 eV) in transmission geometry. Because of the bulky cryostat, the sample holder cannot rotate during experiments, however an average crystallite size in the range of 2-5 µm and using an area 2D detector (Vantec 2000, Bruker) allowed to record diffraction images with reasonable particle statistics. Each 2D image was measured with 31 s exposure. For each experiment 10-12 mg of sample were used. In order cut off reflections coming from the crystalline Be-dome, tungsten slits with 5 mm aperture were mounted on the detector cone. The obtained diffraction images were integrated using DATASQUEEZE 2.2.9[17] with further processing in FITYK 0.9 software[18]. For these experiments no physisorption isotherms were measured in situ but defined dosing pressures were selected based on the previously recorded isotherms. For each pressure point the sample was equilibrated for 300 s before PXRD patterns were recorded. In case of the manual measurements, each pressure was set manually and PXRD patterns were measured after the pressure in the cell was stabilized for at least 300 s. PXRD patterns during adsorption and desorption of MP at 261 K and n-butane at 298 K on DUT-163 are displayed in Supplementary Figure 43, Supplementary Figure 44, respectively

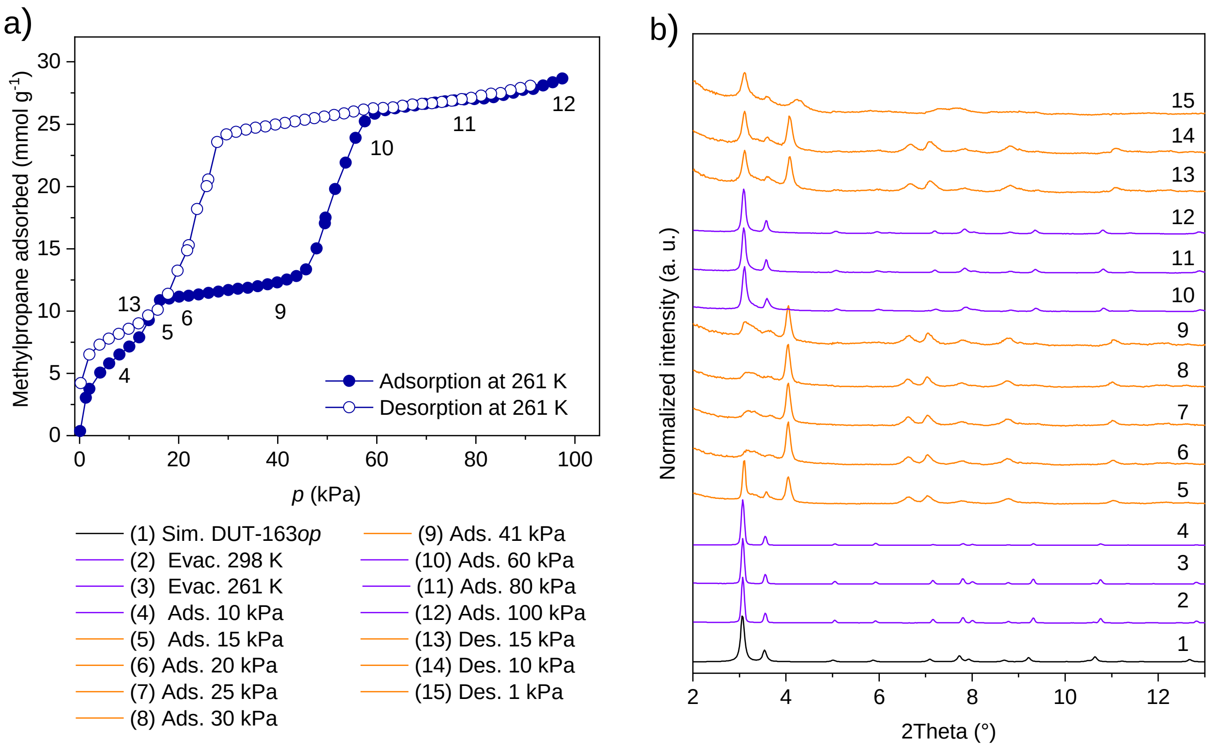

Supplementary Figure 43. a) MP physisorption isotherm (*ex situ*) at 261 K of DUT-163 and b) PXRD patterns recorded at different pressures of MP at 261 K.

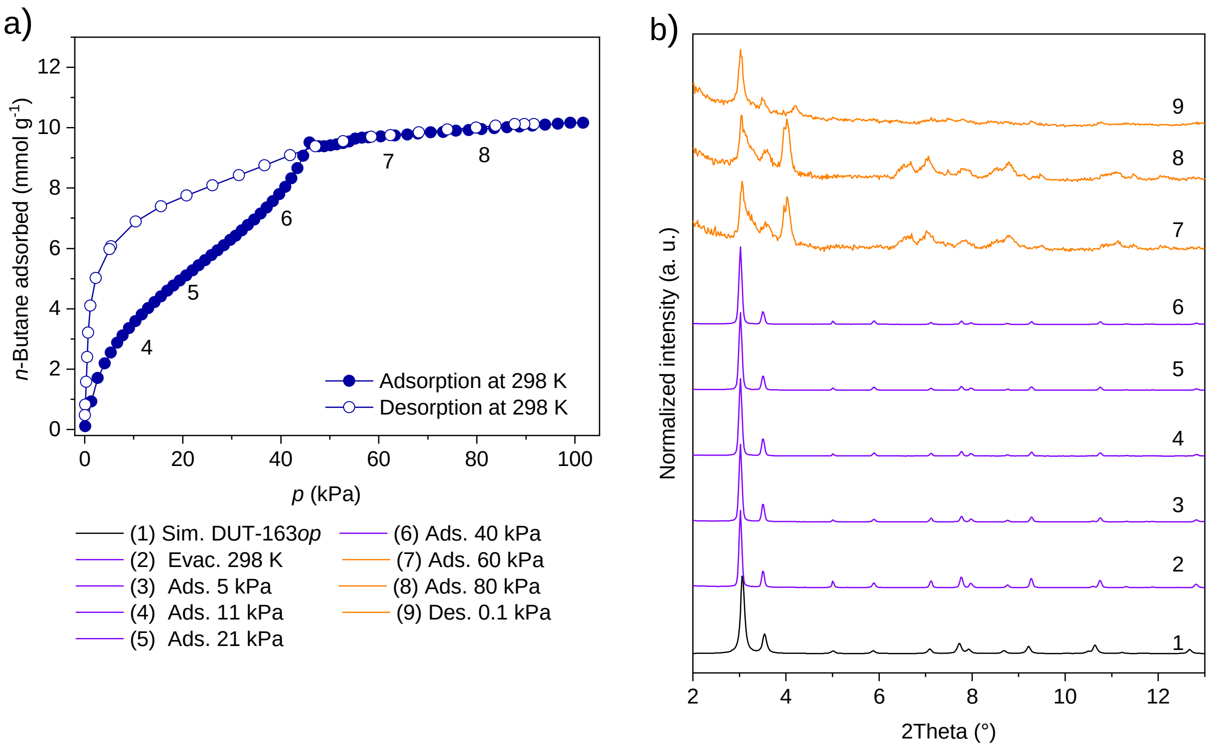

Supplementary Figure 44. a) *n*-Butane physisorption isotherm (*ex situ*) at 298 K of DUT-163 and b) PXRD patterns recorded at different pressures of *n*-butane at 298 K

12) *In situ* PXRD analysis under light irradiation

Gas-cell setup for irradiation at cryogenic temperatures

To characterize phase transitions by PXRD in parallel to gas adsorption and irradiation at cryogenic temperatures (in the conducted experiments methane physisorption in the range of 115-122 K) we modified the sample stage by introducing fiber optics. Experiments were conducted at KMC-2 beamline, operated by HZB.[15] An instrumentation, based on the closed-cycle helium cryostat, gas dosing apparatus and specially designed *in situ* cell was used. A flat sample holder for reflection geometry was designed to facilitate the simultaneous sample irradiation from the top and PXRD measurements. The sample cell was mounted on the cryostat, which was fixed on the goniometer of the beamline. The goniometer was equipped with XYZ-table allows to precisely adjust the sample cell in the centrum of the goniometer (Supplementary Figure 45).

a) b)

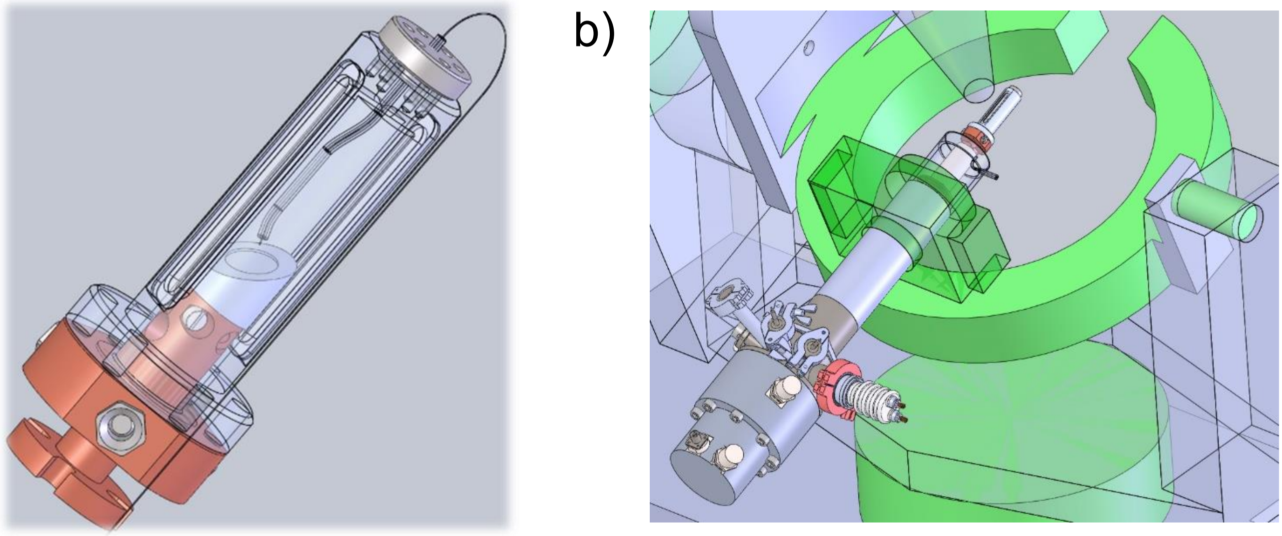

Supplementary Figure 45. Technical drawings of a) the sample cell and b) the goniometer (green) with installed cryostat (lower left) and sample cell (top)

The LEDs M365FP1 (*λ*= 365 nm, 15.5 mW) and M455F3 (*λ*= 455 nm, 24.5 mW) in combination with glass fibre optic from Thorlabs GmbH were used for the sample irradiation. BELSORP-max instrument was used for precise regulation of the pressure in the cell in range of 0.1 Pa to 100 kPa. After each gas dosing step, the sample was equilibrated for at least 300 s to establish adsorption equilibrium conditions.

PXRD patterns were measured using irradiation with E = 8048 eV (λ = 0.15406 nm) and beam size of 0.3x0.3 mm in reflection geometry and ω-2θ scans with step scans with $\Delta 2\theta$ = 1° and 15 s exposure per step. VANTEC 2000 2D detector from Bruker AXS was used for the data collection. The data were automatically integrated using GADDS software and scaled using the powder software, developed at the beamline for this purpose. Because of thermal expansion of the cryostat and contraction / expansion effects of the sample, an automatic height correction was performed before each scan. The thickness of the sample bed in these experiments is estimated to be in the range of 0.8-1.3 mm and cannot be precisely adjusted.

Based on the previously collected methane adsorption data (Supplementary Figure 30) four experiments were conducted.

1. Analysis of methane adsorption at 115 K without irradiation → sample change
2. Analysis of methane adsorption at 122 K without irradiation
3. Analysis of methane adsorption at 120 K with irradiation (365 nm, 455 nm)
4. Analysis of methane adsorption at 120 K without irradiation

Experiments 2-4 were conducted on the same sample using the recycling procedures described above.

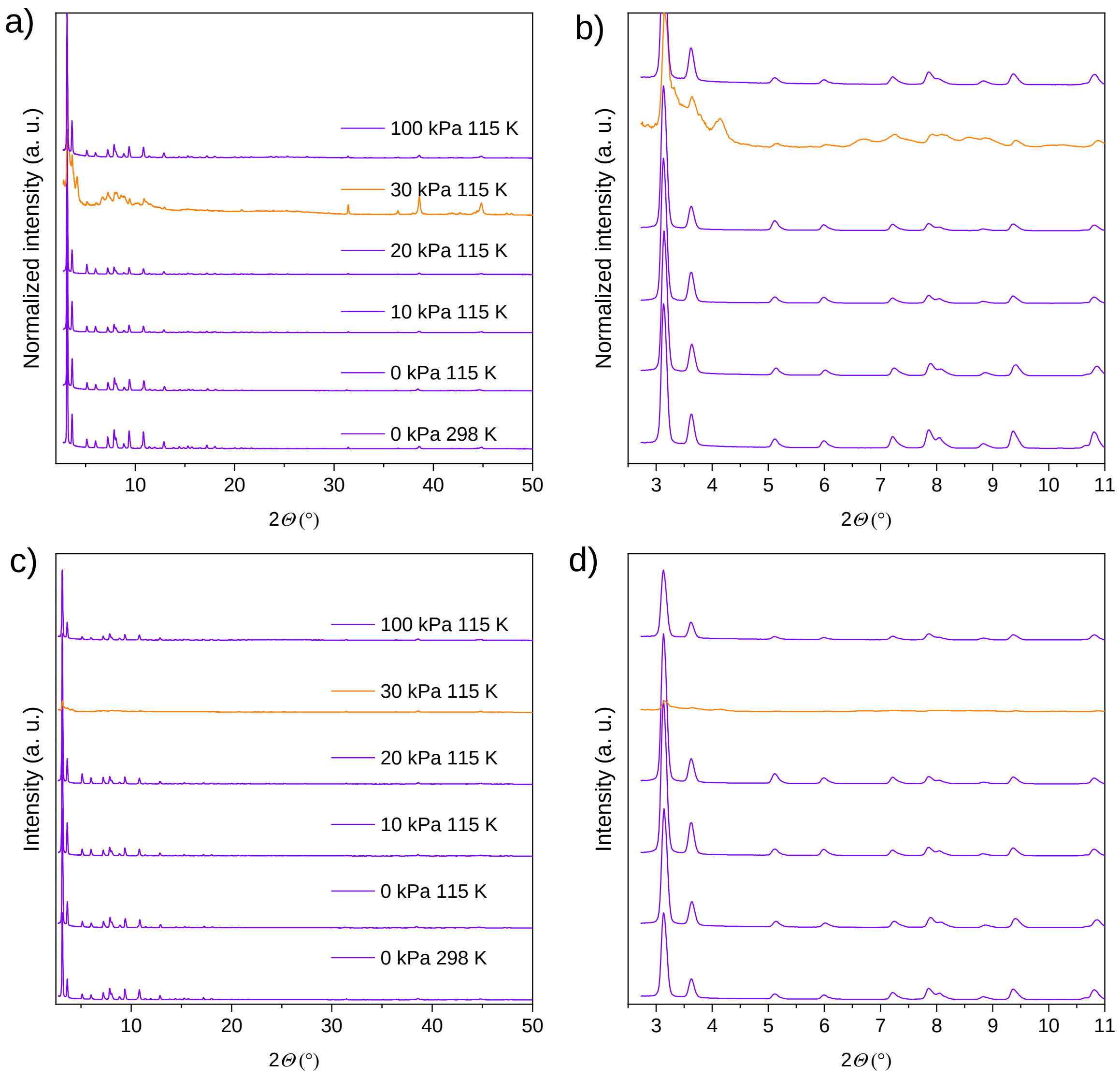

Supplementary Figure 46. a-d) PXRD patterns of DUT-163 recorded in parallel to adsorption of methane at 115 K and different pressures. a,b) intensities normalized to the maximum intensity, c,d) intensities as obtained from the experiment. PXRD pattern after structural contraction is colored orange.

In situ PXRD experiments of methane adsorption in DUT-163 at 115 K show an almost complete disappearance of the peak intensity at 30 kPa and the appearance of reflections with low intensity that can be assigned to the formation of the *cp* phase. At higher pressures of 100 kPa the peaks of the op reappear and the *cp* phase is found to be reversibly transferred back to the gas-filled *op* phase. This reversable breathing is typical for DUT-49 type frameworks and validates the experimental setup.

The second experiment was conducted at 122 K, a temperature at which no adsorption-induced contraction is found to occur (Supplementary Figure 30). In fact, up to a pressure of 55 kPa no structural contraction indicated by peaks of the cp phase is observed by *in situ* PXRD (Supplementary Figure 47).

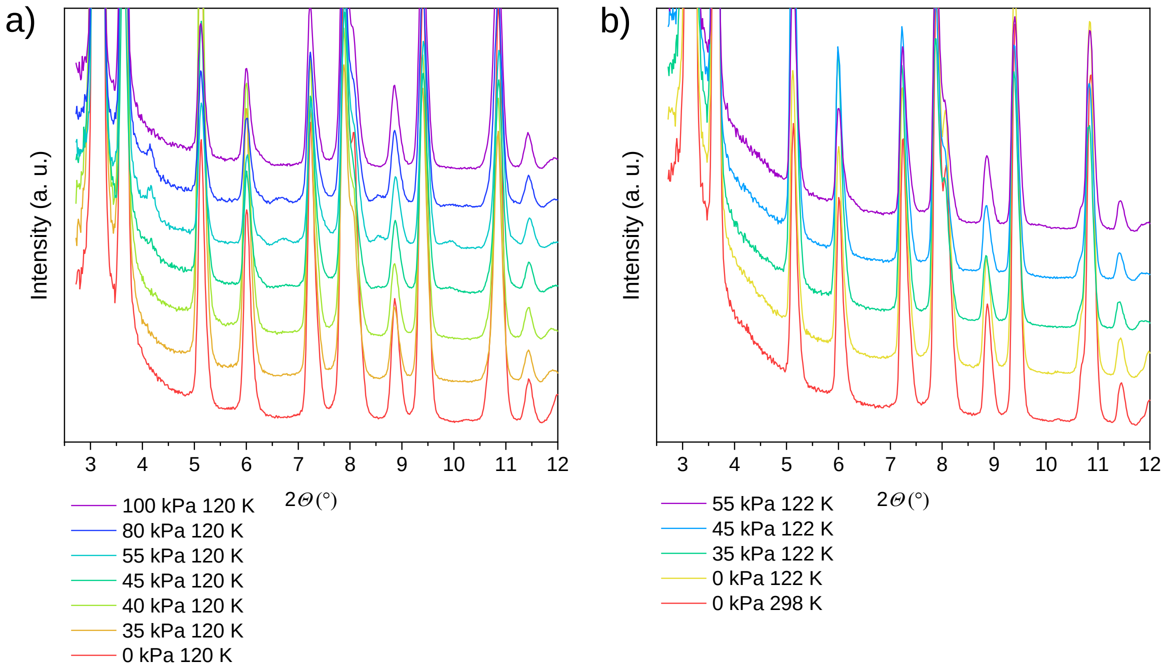

Supplementary Figure 47. a) PXRD patterns of DUT-163 recorded in parallel to adsorption of methane at 120 K and different pressures, b) PXRD patterns recorded in parallel to adsorption of methane at 122 K and different pressures.

The third experiment was conducted in parallel to methane adsorption at 120 K, a temperature close to the temperature range at which adsorption-induced contraction is found to occur (Supplementary Figure 30). First the sample was irradiated at 365 nm and 455 nm in parallel to the adsorption process up to a pressure of 40 kPa.

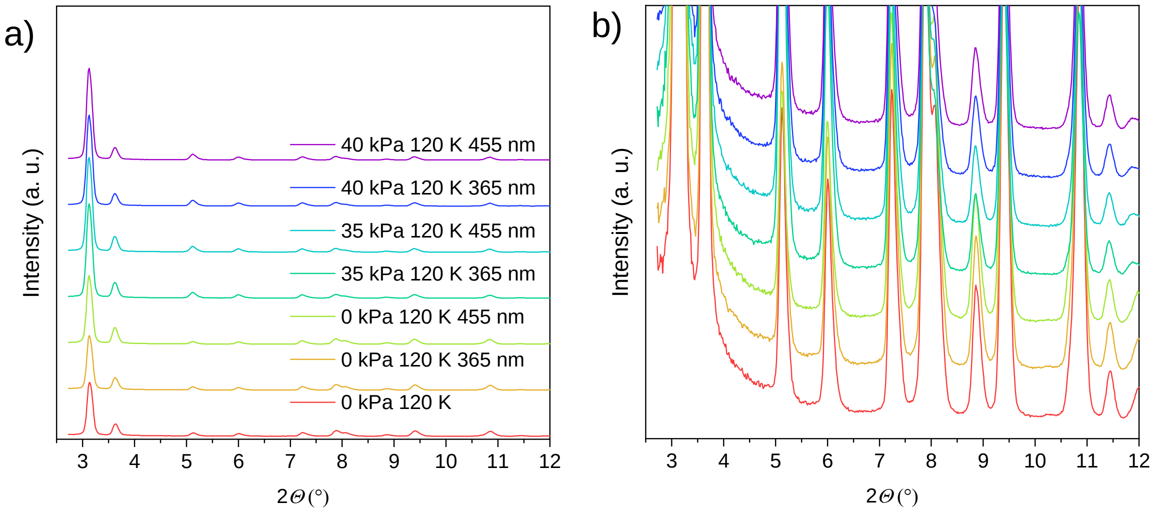

Supplementary Figure 48. a-b) PXRD patterns of DUT-163 recorded in parallel to adsorption of methane at 120 K and different pressures with irradiation at 365 nm and 455 nm. b) is enlargement with enhanced intensity of a).

No reflections other than the op phase are observed. It was found that the fiber optics used to connect strongly absorb 365 nm light and the experiments were continued using a 455 nm LED exclusively. Interestingly the presence of the *cp* phase is observed in the pressure range of 45-80 KPa. At 100 kPa peaks of the *cp* phase disappeared and only peaks of the *op* phase are present indicating a complete

reopening of the partially contracted framework (Supplementary Figure 49). The sample was subsequently recycled by increasing the temperature to 130 K and desorbing the methane in dynamic vacuum.

The fourth experiment was conducted on the same sample in parallel to methane adsorption at 120 K without irradiation. Interestingly, small peaks of the *cp* phase occur in the pressure range of 45-80 KPa but with peak intensity inferior to the previously conducted experiment with irradiation. At 100 kPa peaks of the *cp* phase disappeared and only peaks of the *op* phase are present indicating a complete reopening of the partially contracted framework.

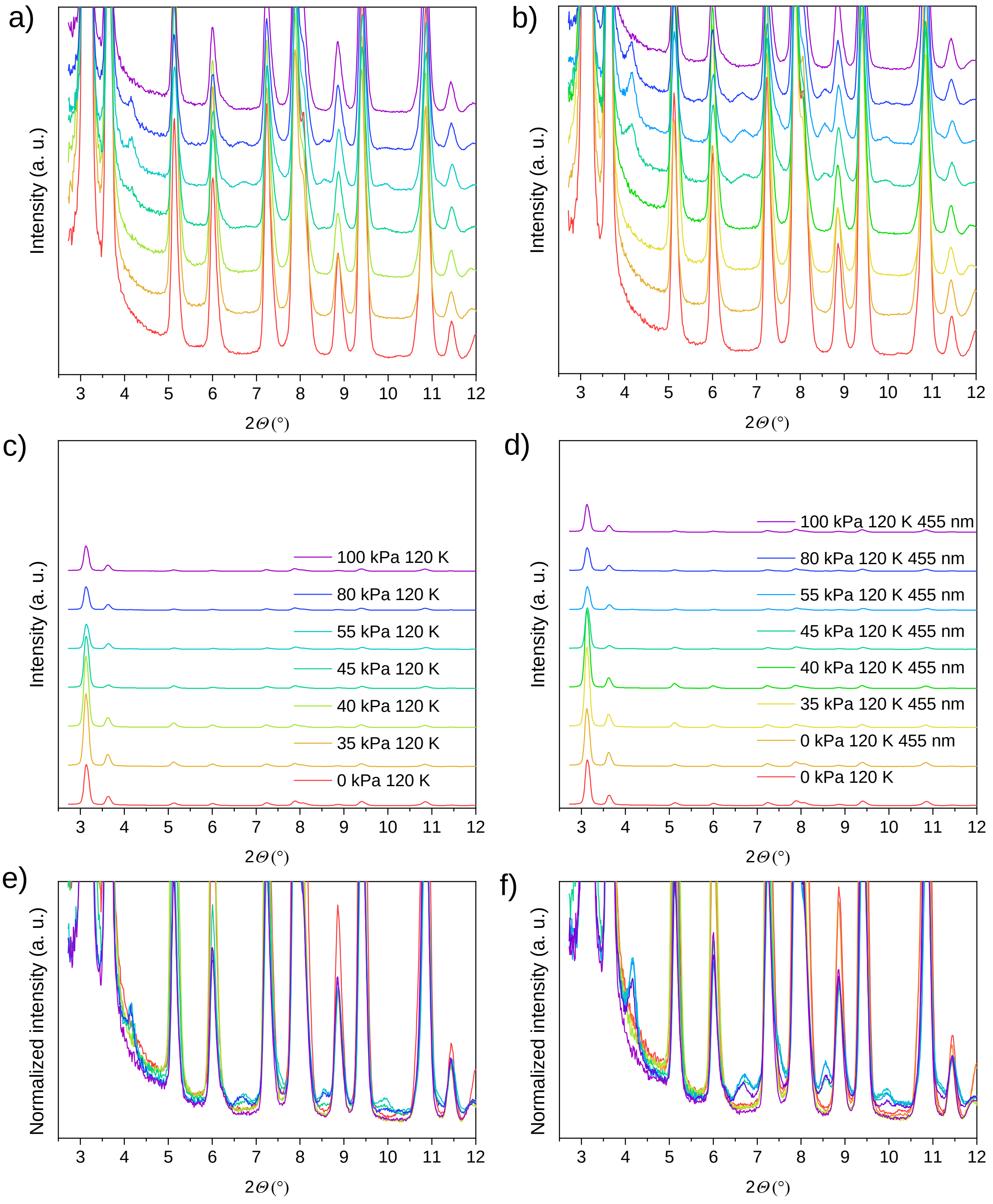

Supplementary Figure 49. a-f) PXRD patterns of DUT-163 recorded in parallel to adsorption of methane at 120 K and different pressures without irradiation (a,c,e) and irradiation with 455 nm (b,d,f). a,b) magnified intensities, c,d) scaled intensities to largest peak, e,f) intensities normalized to the maximum intensity.

To better illustrate the evolution of the different phases peak intensities for the op (3.128°) and cp (4.177°) were extracted and plotted against the pressure. It is obvious that peak intensities of the op phase change as a function of methane pressure. This can be explained by the incorporation of methane molecules in the pores of DUT-163. These methane molecules, although not spatially fixed, transiently occupy different crystallographic positions and thus contribute to the scattering intensity of the framework. This property is well known from X-ray studies in parallel to Ar adsorption[19] in mesoporous materials in which an increase in scattering intensity is observed upon mesopore filling which is then again reduced with increasing pressure and pore saturation. Comparison with the dataset recorded at 115 K shows a sharp drop in signal intensity of the *op* phase due to structural contraction.

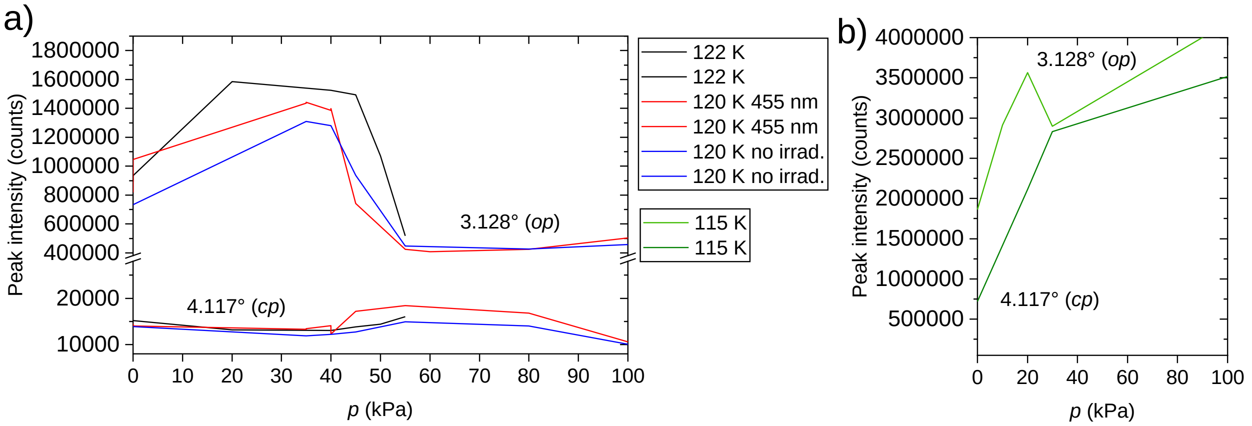

Supplementary Figure 50. Evolution of intensity of 111 reflection of DUT-163cp and op as a function of methane pressure at a) 122 K without irradiation and 120 K with and without irradiation, and b) 115 K without irradiation.

The low scattering intensity of DUT-163*cp* is in line with previous investigations of DUT-49 related mateirals, in particular those that exhibit elongated ligand backbones. Low scattering intensity of the cp phase can be caused by a combination of structural disorder and the saturation with methane.

The experiments clearly demonstrate the reversibility of the *op-cp* transition in DUT-163. Furthermore, the sample can be recycled by applying a protocol that involves desorption of the gas at elevated temperatures. Undoubtful analysis of the photo-induced structural contraction cannot be delivered from this experiment since peaks of the cp phase are also present without the application of a lightsource. In addition, it is unclear to what extend the fibre optics required in this experimental setup reduce the light intensity that hits the sample and which penetration depth can be expected from a powdered sample with a thickness of ca. 1 mm. To analyze these factors, we decided to apply a second experimental setup specifically designed to analyze photoirradiation at ambient conditions.

Capillary setup for irradiation at ambient temperature

Because the Be-domes used in the above described setup do not allow to irradiate the samples and the light intensity and penetration depth in the cryogenic fibre-optics-based setup were insufficient a different setup was used based on an instrumentation originally built for high pressure adsorption[20]. In this setup <0.2 mg of DUT-163 powder is placed in quartz capillary (diameter <1.5 mm) which are connected to a gas dosing system via a VCR valve and Cu-capillary and a cryojet system for temperature control (setup shown in Supplementary Figure 51). The sample is rotated and can be axially irradiated by light in the UV-Vis range and in parallel investigated by PXRD (Supplementary Figure 51).

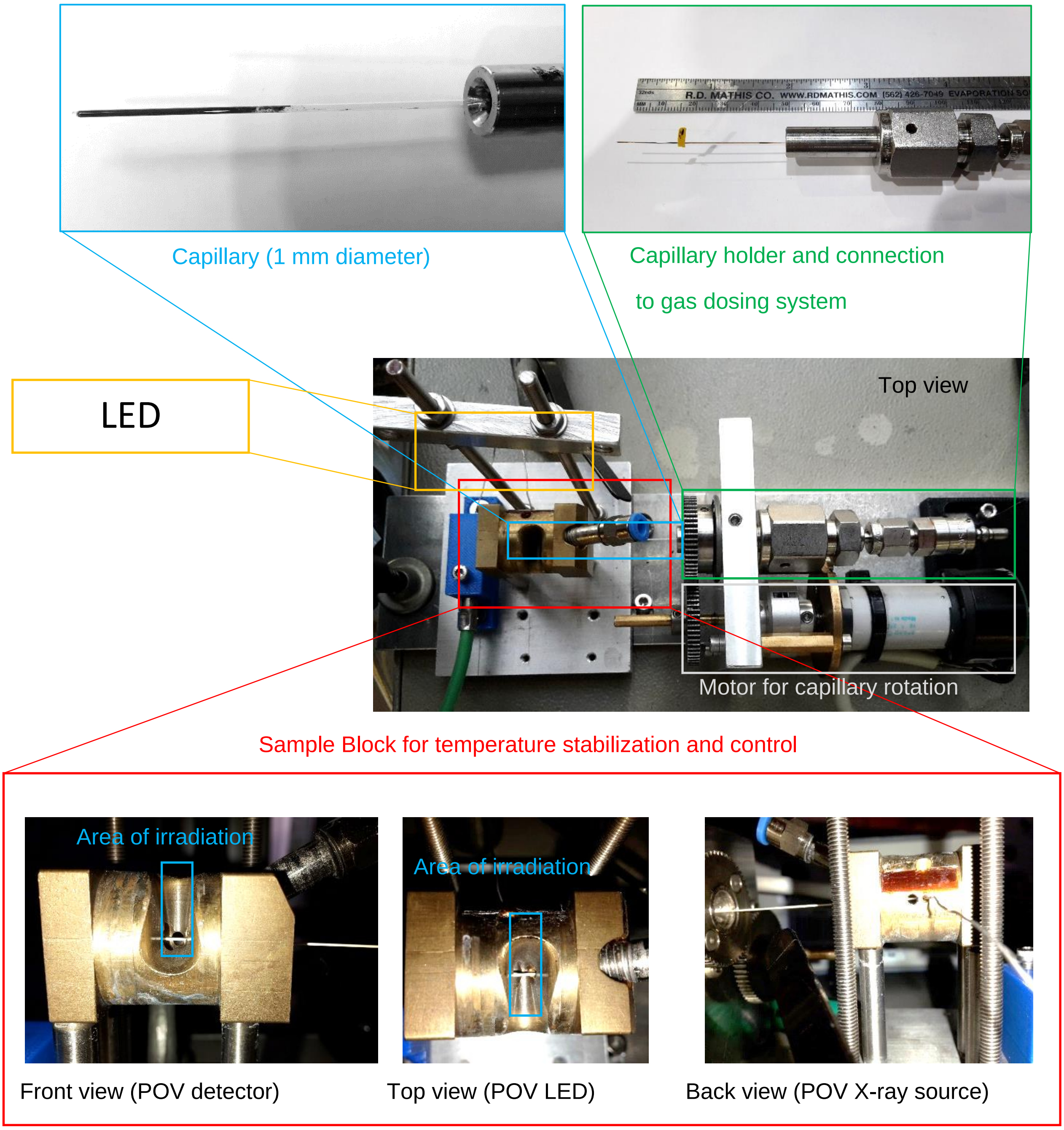

Supplementary Figure 51. Experimental setup used to investigate structural transitions in DUT-163 by PXRD in parallel to adsorption of MP in the temperature range of 296 – 300 K with and without irradiation of the sample with nominal wavelength of 365 nm or 455 nm.

The capillary can be rotated for improved diffraction statistics and uniform irradiation. The sample temperature is controlled via an Oxford cryojet system that allows to set the sample temperature in the range of 270-310 K with a temperature fluctuation below ±0.1 K. The sample temperature is monitored by a sensor attached adjacent to the sample capillary and recorded at a frequency of 0.5 Hz. To stabilize the adsorption temperature the sample-filled capillary is placed in a copper block with holes drilled for the capillary, the cryojet inlet for temperature control, the temperature sensor, the X-ray beam, and for light irradiation.

The setup is installed at KMC-2 beamline of the BESSY II synchrotron[15] and PXRD patterns were measured at constant wavelength $\lambda = 0.15406$ nm (E = 8048 eV) in transmission geometry using the same detector system as descried above. However, in this experimental setup the detector was kept static to allow continuous recording of PXRD data of the 2θ-range of 2-20 ° required for the dynamic gas dosing applied in these experiments. The sample capillary is rotated at a frequency of 0.016 Hz and PXRD patterns were recorded continuously with exposure times of 20s exposure, 10s blank (experiment CAP1-CAP9) and 55 s exposure,

5 s blank (CAP10-CAP20). To compare the absolute intensity evolution of different experiments the PXRD patterns from experiment CAP1-CAP9 were adjusted to represent the same exposure time compared to CAP10-CAP20. Quartz capillaries with diameters of 0.3 mm, 0.7 mm, and 1 mm were used to assure maximum exposure of the sample with light and a high transmission and good resolution of X-ray diffraction. No adsorption isotherms could be recorded *in situ* due to the small sample amount that fits inside the capillaries.

Gas dosing was performed continuously and not stepwise as compared to the previously mentioned setup for analysing MP adsorption at 262 K. All experiments were conducted using MP of 99.9% purity. To assure quasi-equilibrated adsorption conditions over the whole bulk sample, small sample amounts below 0.3 mg (compared to 10-12 mg in the traditional Be-dome setup) and the pressure increase was kept below a rate of 10 Pa s^{-1}. This results in a pressure resolution of 0.3 (at low pressure)-1.2 kPa (at high pressure) per PXRD pattern (exposure 50s, deadtime 10s). The gas dosing was performed using a syringe pump (Modell 260D from TELEDYNE ISCO) with a maximum cylinder volume of 260 cm^3. The decrease of this volume by moving the piston at a certain fixed rate generated a constant gas flow from the gas-filled cylinder volume to the sample capillary resulting in a hyperbolic pressure increase. After each experiment, the whole dosing system was evacuated and flushed with fresh methylpropane (MP) to avoid contamination. Experiments with 0.3 mm and 1 mm capillary were conducted in the following order:

1) Quartz capillary was glued in a Swagelok sample holder
2) Sample was placed in capillary under inert conditions in an Ar-filled glovebox
3) Capillary was placed in the sample stage and connected to the gas dosing system
4) Sample was evacuated in dynamic vacuum at $p<10^{-2}$ Pa for at least 20 min
5) Sample temperature was set and equilibrated
6) PXRD pattern of evacuated sample (p = 0 kPa) was recorded
7) Sample was exposed to $p \approx 30$ kPa MP
8) External light source (LED with corresponding wavelength) was installed and operated
9) Continuous PXRD recording and pressure increase (adsorption) were started simultaneously until a pressure in the range of 100 kPa was reached where PXRD recording was halted
10) Continuous PXRD recording (55 s exposure, 5 s blank) and pressure decrease (desorption) were started simultaneously until the initial pressure of 20 kPa was reached where PXRD recording was halted
11) Depending on the nature of the experiment (summary of experiments in Supplementary Table 2) the sample was recycled, evacuated or exchanged

Supplementary Table 2. Summary of *in situ* PXRD/irradiation experiments conducted with a rotating capillary-based setup. Experiments that were performed on the same sample are grouped in double-lined rows.

Experiment ID	Figure	*T* (K)	Excitation wavelength (nm)	Pressure (kPa)	p_{op-cp} (kPa)	Cappilary diameter (mm)
CAP1	Supplementary Figure 52	296	No irradiation	Ads. 30-106		0.7
CAP2	Supplementary	296	365	Ads. 106		0.7
CAP3	Figure 52	296	455	Ads. 106		0.7
CAP4	Supplementary Figure 52	296	No irradiation	Des. 106-30		0.7
CAP5	Supplementary Figure 52	300	365	Evacuated		0.7
CAP6	Supplementary Figure 52	300	No irradiation	Ads. 20-97		0.7
CAP7	Supplementary Figure 52	300	No irradiation	Des. 97-20		0.7
CAP8	Supplementary Figure 52	300	365	Ads. 20-107		0.7
CAP9	Supplementary Figure 52	300	455	Ads. 107		0.7
CAP10	Supplementary Figure 52	300	No irradiation	Des. 107-20		0.7
CAP11	Supplementary Figure 64	300	No irradiation	Ads. 28-111		1
CAP12	Supplementary Figure 64	300	No irradiation	Des. 111-28		1
CAP13	Supplementary Figure 64	300	365	Ads. 28-109		1
CAP13	Supplementary Figure 64	300	365	Des. 109-38		1
CAP14	Supplementary Figure 64	300	No irradiation	Ads. 29-111		0.3
CAP15	Supplementary Figure 64	300	No irradiation	Des. 111-29		0.3
CAP16	Supplementary Figure 64	300	365*	Ads. 29-111		0.3
CAP17	Supplementary Figure 64	300	No irradiation	Des. 111-29		0.3

* Irradiation only in the pressure range of 70-110 kPa

Supplementary Table 3. Summary of axial capillary scans by *in situ* PXRD

Experiment ID	Figure	*T* (K)	Excitation wavelength (nm)	Pressure range (kPa)	Cappilary diameter (mm)
Cap18		300	No light	Des. 20	0.7
Cap29		300	No light	Des. 20	1

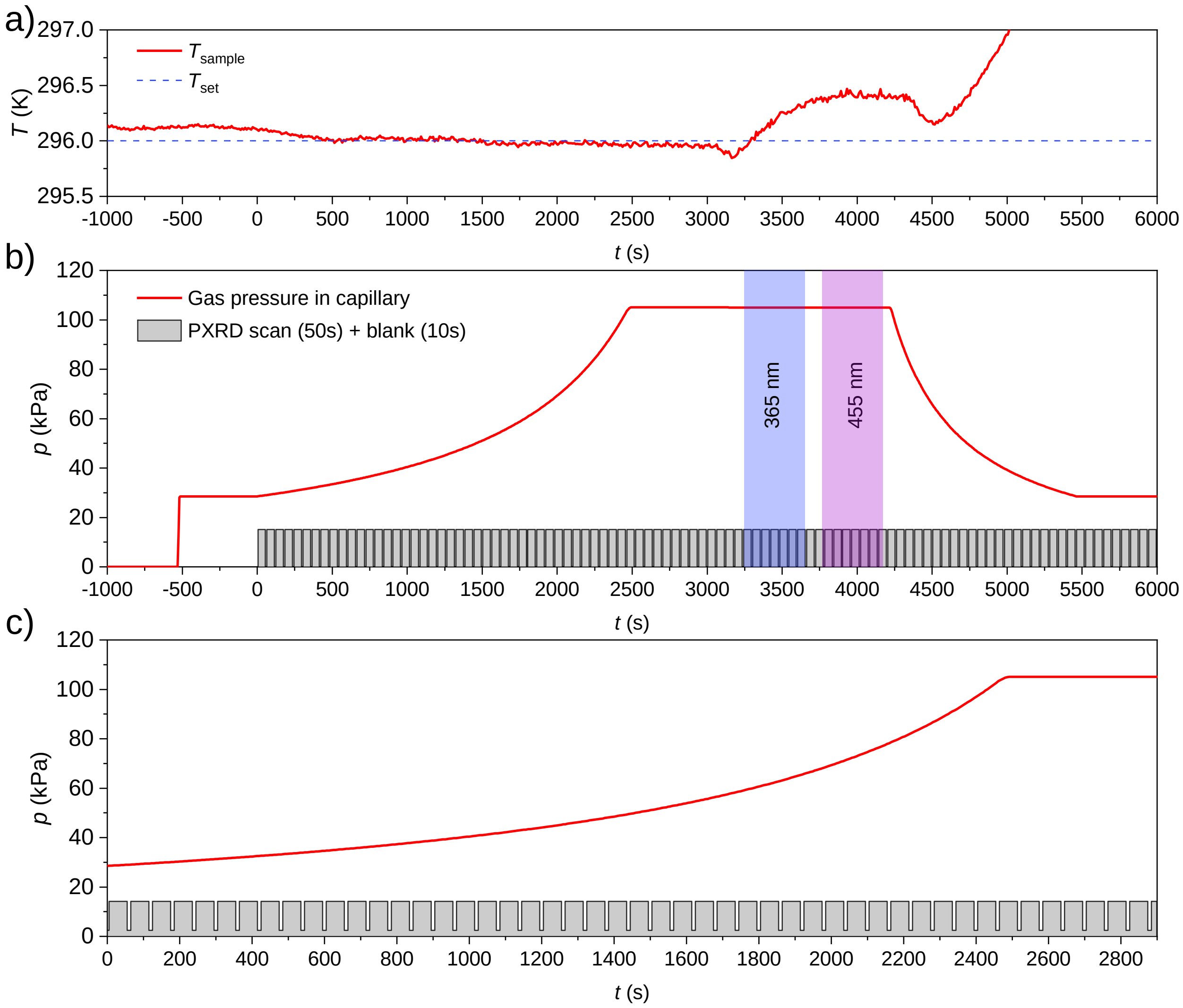

Supplementary Figure 52. a) Time-dependent temperature evolution during *in situ* PXRD experiment CAP1-CAP3, b) time-dependent pressure evolution during *in situ* PXRD experiment CAP1-CAP3 (Supplementary Table 2), and c) magnified region during adsorption. Grey area indicates exposure period of detector for recording of diffraction images.

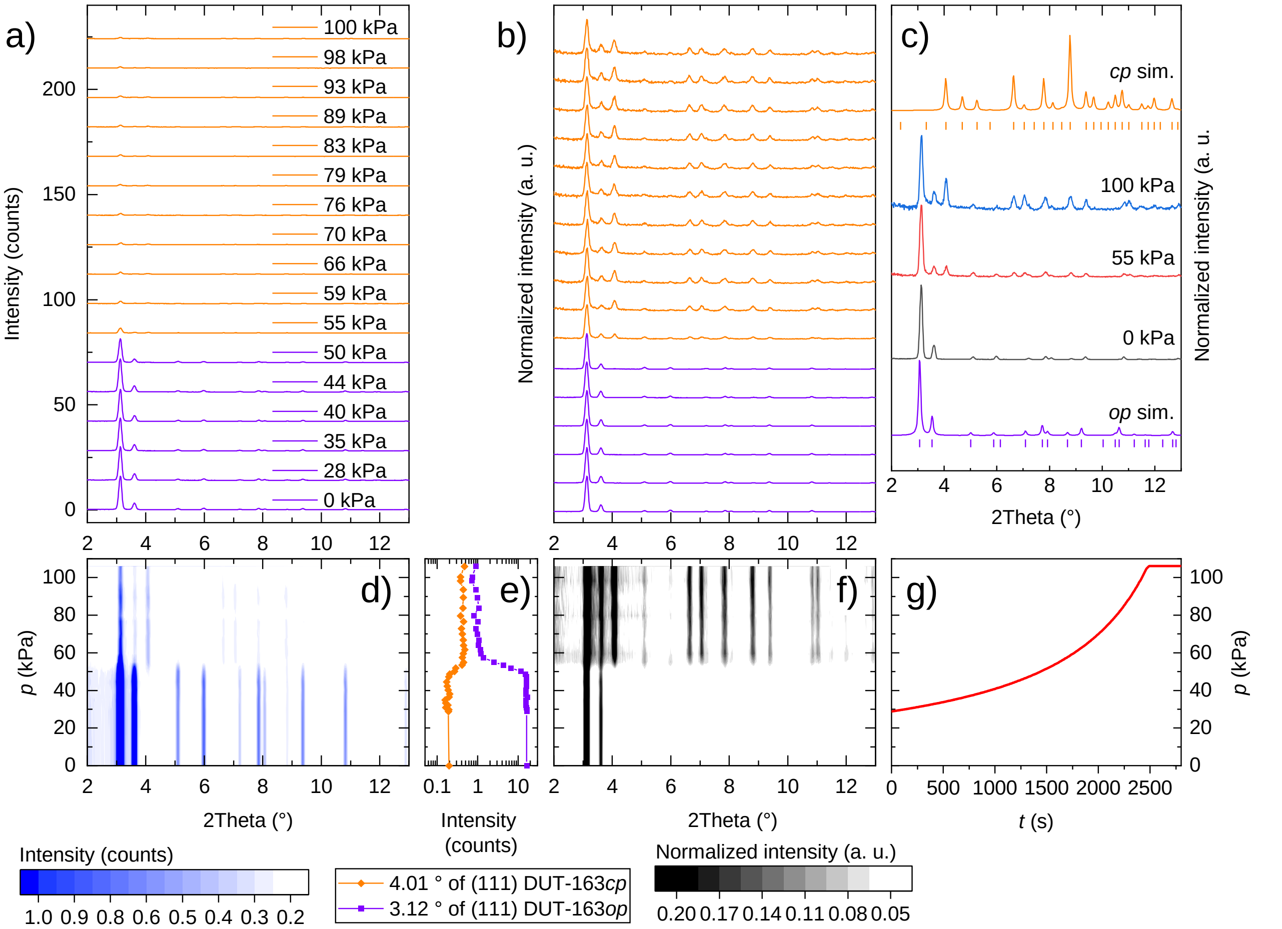

Supplementary Figure 53. a-b) PXRD patterns recorded at 296 K with increasing pressure of MP and no irradiation (CAP1 Supplementary Table 2). c) selected PXRD patterns in comparison with simulated PXRD patterns of DUT-163*op* (purple) and DUT-163*cp* (orange) and theoretical peak positions as vertical lines. d,f) Contour plots based on PXRD data with absolute intensity (a,d) and normalized to the highest intensity (b,f). e) Intensity evolution at the peak positions of the (111) reflections of DUT-163*op* (purple) and DUT-163*cp* (orange). g) Evolution of MP pressure during the experiment.

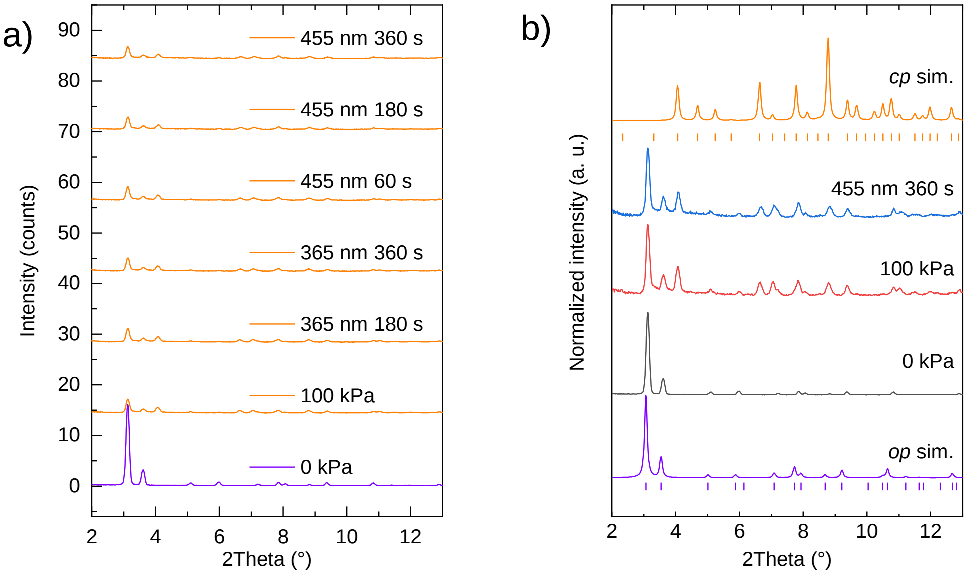

Supplementary Figure 54. a,b) PXRD patterns recorded at 296 K at different pressure of MP and irradiation with 365 (experiment CAP2 Supplementary Table 2) and 455 nm (experiment CAP3 Supplementary Table 2). b) selected PXRD patterns in comparison with simulated PXRD patterns of DUT-163*op* (purple) and DUT-163*cp* (orange) and theoretical peak positions as vertical lines.

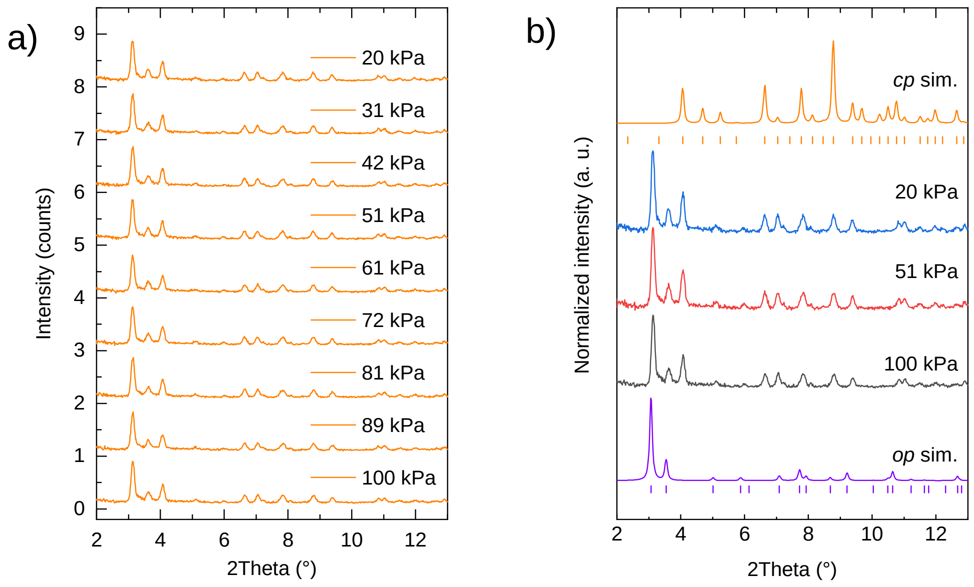

Supplementary Figure 55. a,b) PXRD patterns recorded at 296-298 K at different pressure of MP during desorption without irradiation (experiment CAP4 Supplementary Table 2). b) selected PXRD patterns in comparison with simulated PXRD patterns of DUT-163*op* (purple) and DUT-163*cp* (orange) and theoretical peak positions as vertical lines.

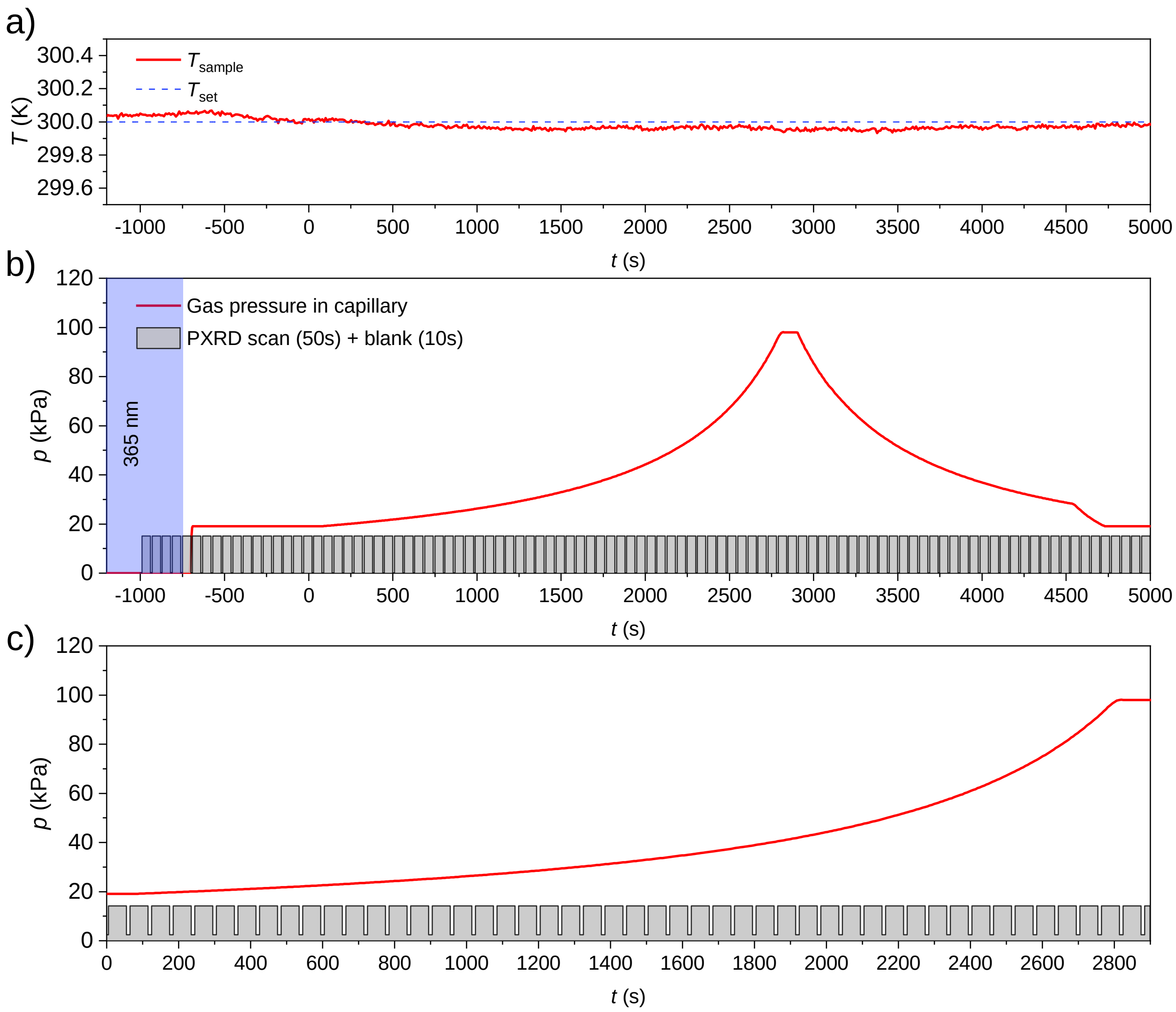

Supplementary Figure 56. a) Time-dependent temperature evolution during the experiment, b) pressure evolution during *in situ* PXRD experiment Cap-5- (Supplementary Table 2) and c) magnified region during adsorption. Grey area indicates exposure period of detector for recording of diffraction images.

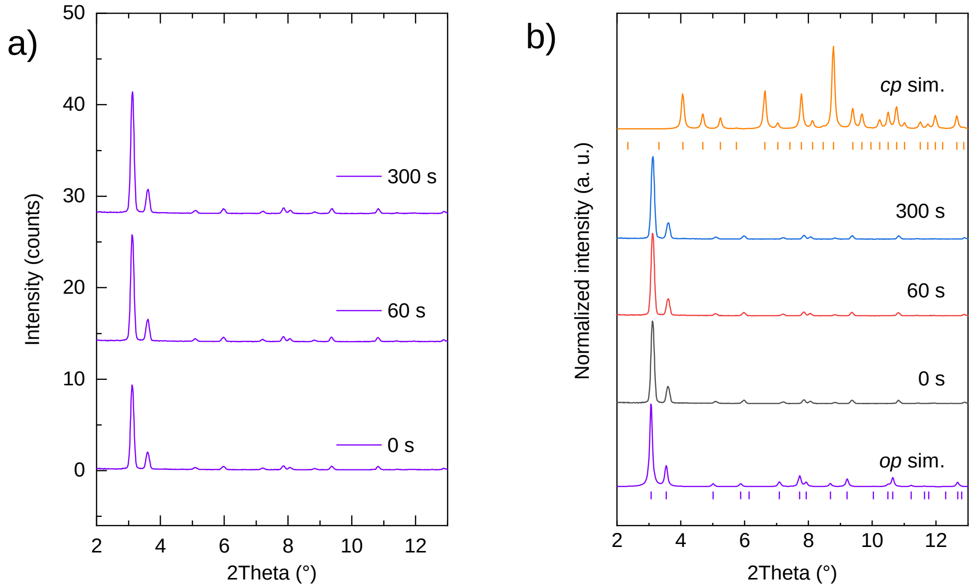

Supplementary Figure 57. a,b) PXRD patterns recorded at 300 K of evacuated DUT-163 irradiated with 365 nm for different durations (experiment CAP5 Supplementary Table 2). b) selected PXRD patterns in comparison with simulated PXRD patterns of DUT-163*op* (purple) and DUT-163*cp* (orange) and theoretical peak positions as vertical lines.

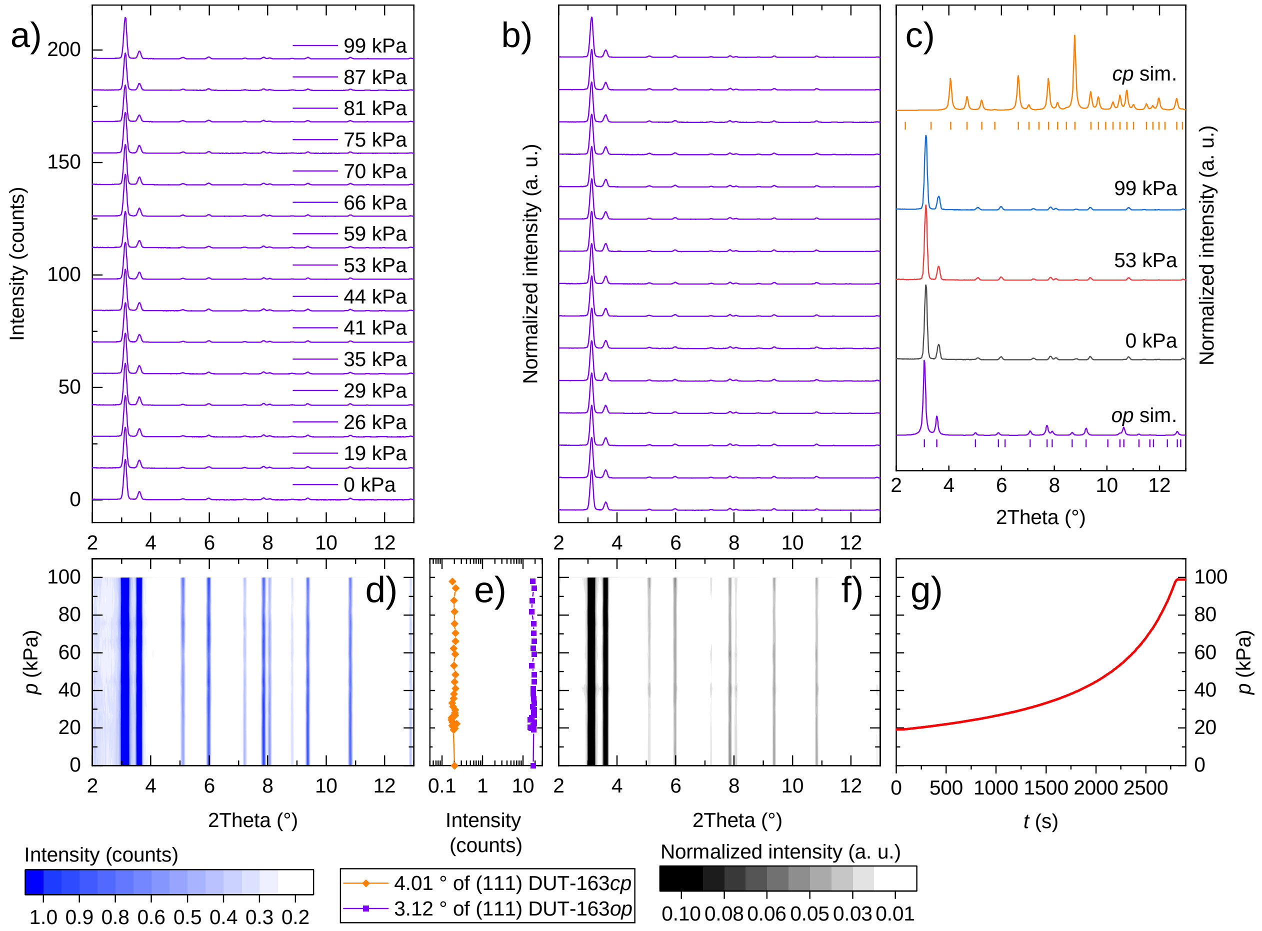

Supplementary Figure 58. a-b) PXRD patterns recorded at 300 K with increasing pressure of MP and no irradiation (experiment Cap-6). c) selected PXRD patterns in comparison with simulated PXRD patterns of DUT-163*op* (purple) and DUT-163*cp* (orange) and theoretical peak positions as vertical lines. d,f) Contour plots based on PXRD data with absolute intensity (a,d) and normalized to the highest intensity (b,f). e) Intensity evolution at the peak positions of the (111) reflections of DUT-163*op* (purple) and DUT-163*cp* (orange). g) Evolution of MP pressure during the experiment.

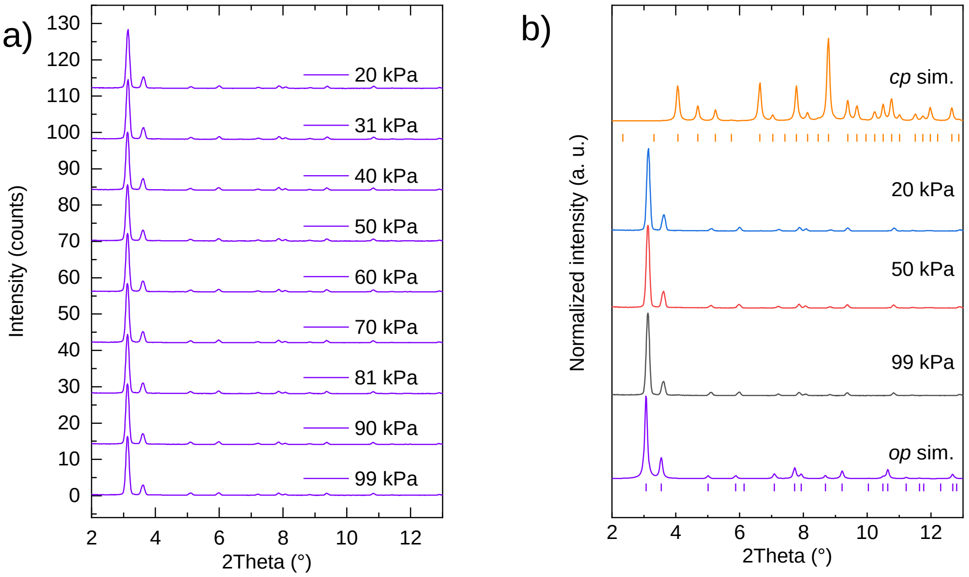

Supplementary Figure 59. a,b) PXRD patterns recorded at 300 K at different pressure of MP during desorption without irradiation (experiment CAP7 Supplementary Table 2). b) selected PXRD patterns in comparison with simulated PXRD patterns of DUT-163*op* (purple) and DUT-163*cp* (orange) and theoretical peak positions as vertical lines.

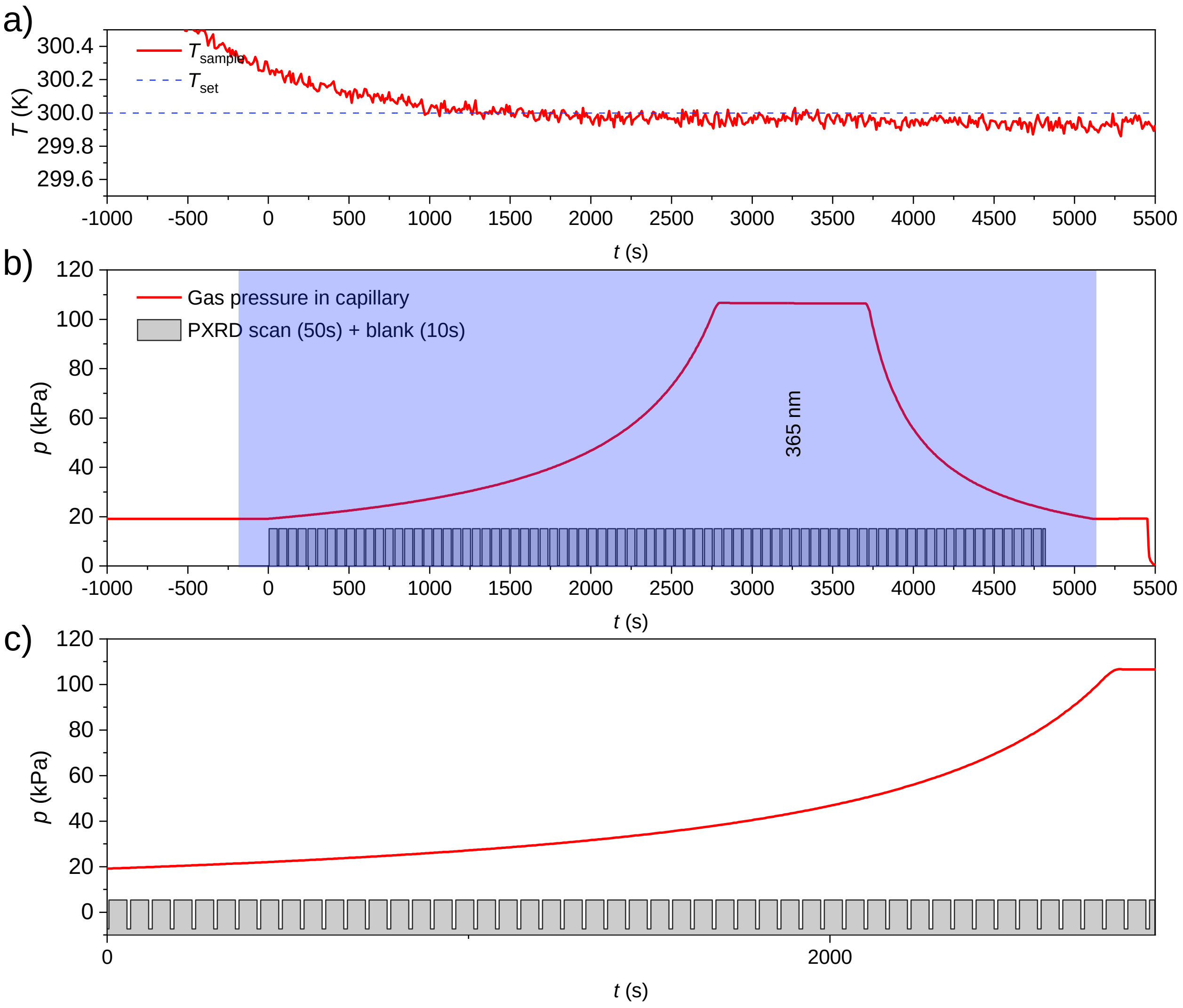

Supplementary Figure 60. a) Time-dependent temperature evolution during the experiment, b) time-dependent pressure evolution during *in situ* PXRD experiment Cap-8-10 (Supplementary Table 2) and c) magnified region during adsorption. Grey area indicates exposure period of detector for recording of diffraction images.

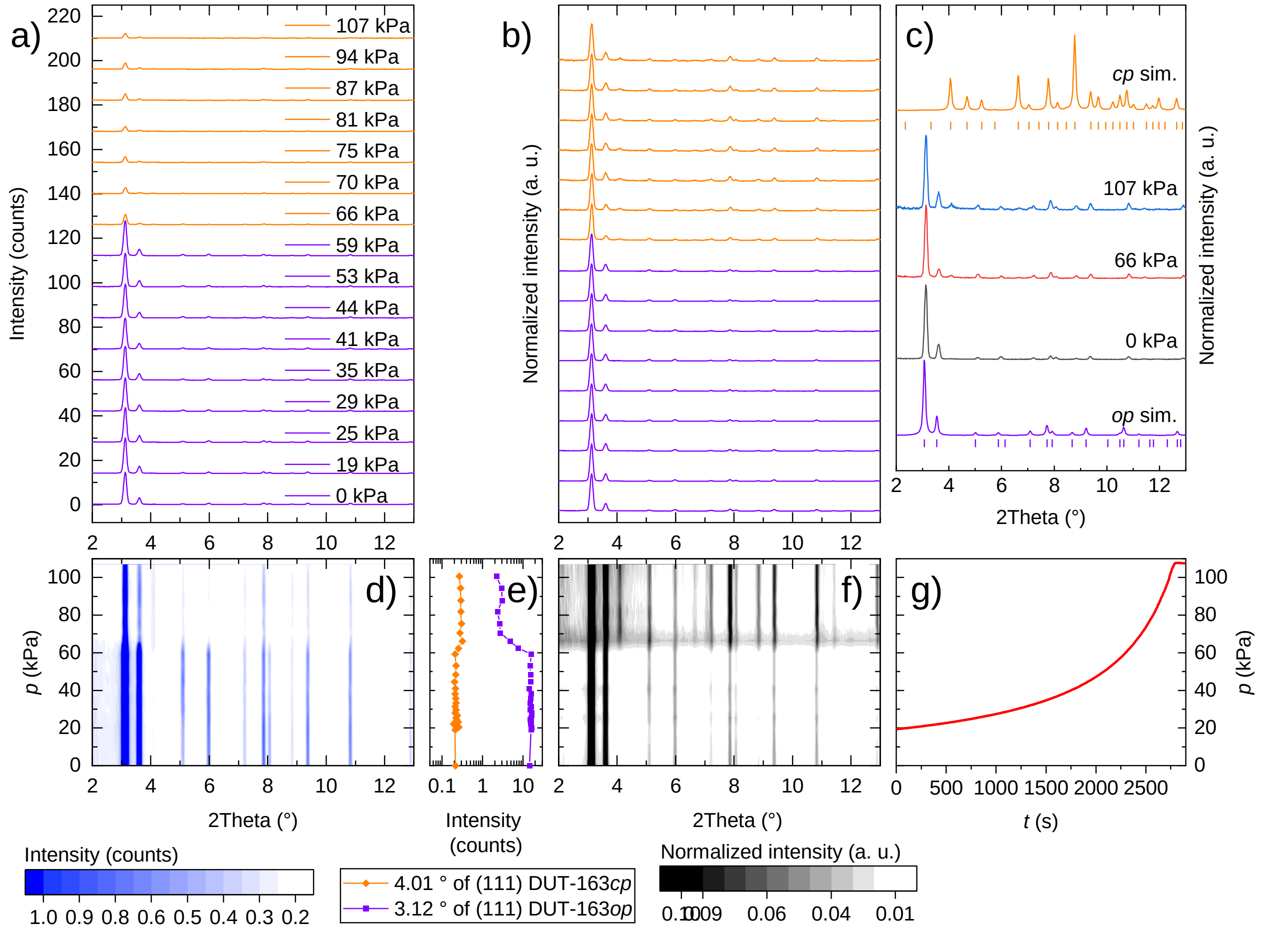

Supplementary Figure 61. a-b) PXRD patterns recorded at 300 K with increasing pressure of MP and irradiation at 365 nm (experiment CAP8,CAP9). c) selected PXRD patterns in comparison with simulated PXRD patterns of DUT-163*op* (purple) and DUT-163*cp* (orange) and theoretical peak positions as vertical lines. d,f) Contour plots based on PXRD data with absolute intensity (a,d) and normalized to the highest intensity (b,f). e) Intensity evolution at the peak positions of the (111) reflections of DUT-163*op* (purple) and DUT-163*cp* (orange). g) Evolution of MP pressure during the experiment.

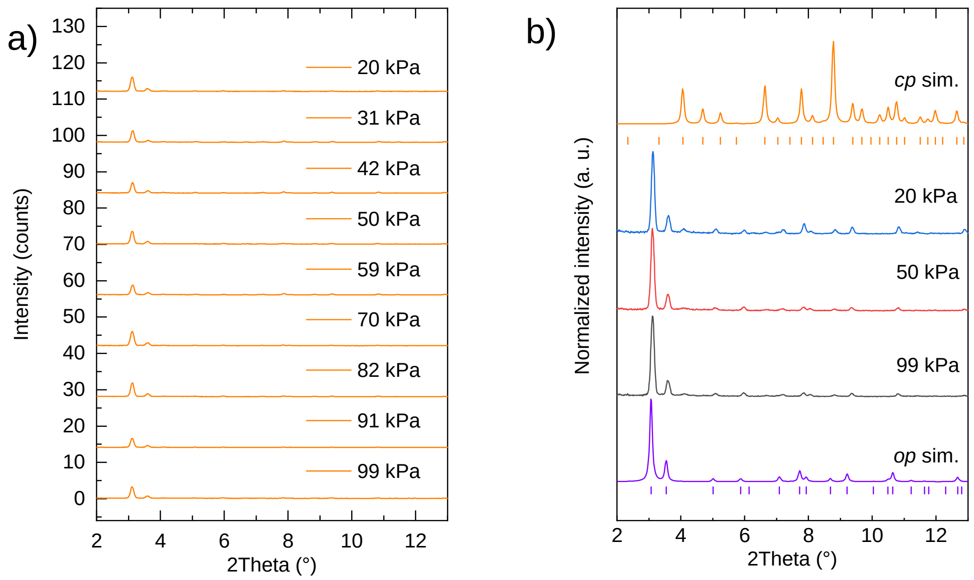

Supplementary Figure 62. a,b) PXRD patterns recorded at 300 K at different pressure of MP during desorption with no irradiation (experiment CAP10 Supplementary Table 2). b) selected PXRD patterns in comparison with simulated PXRD patterns of DUT-163*op* (purple) and DUT-163*cp* (orange) and theoretical peak positions as vertical lines.

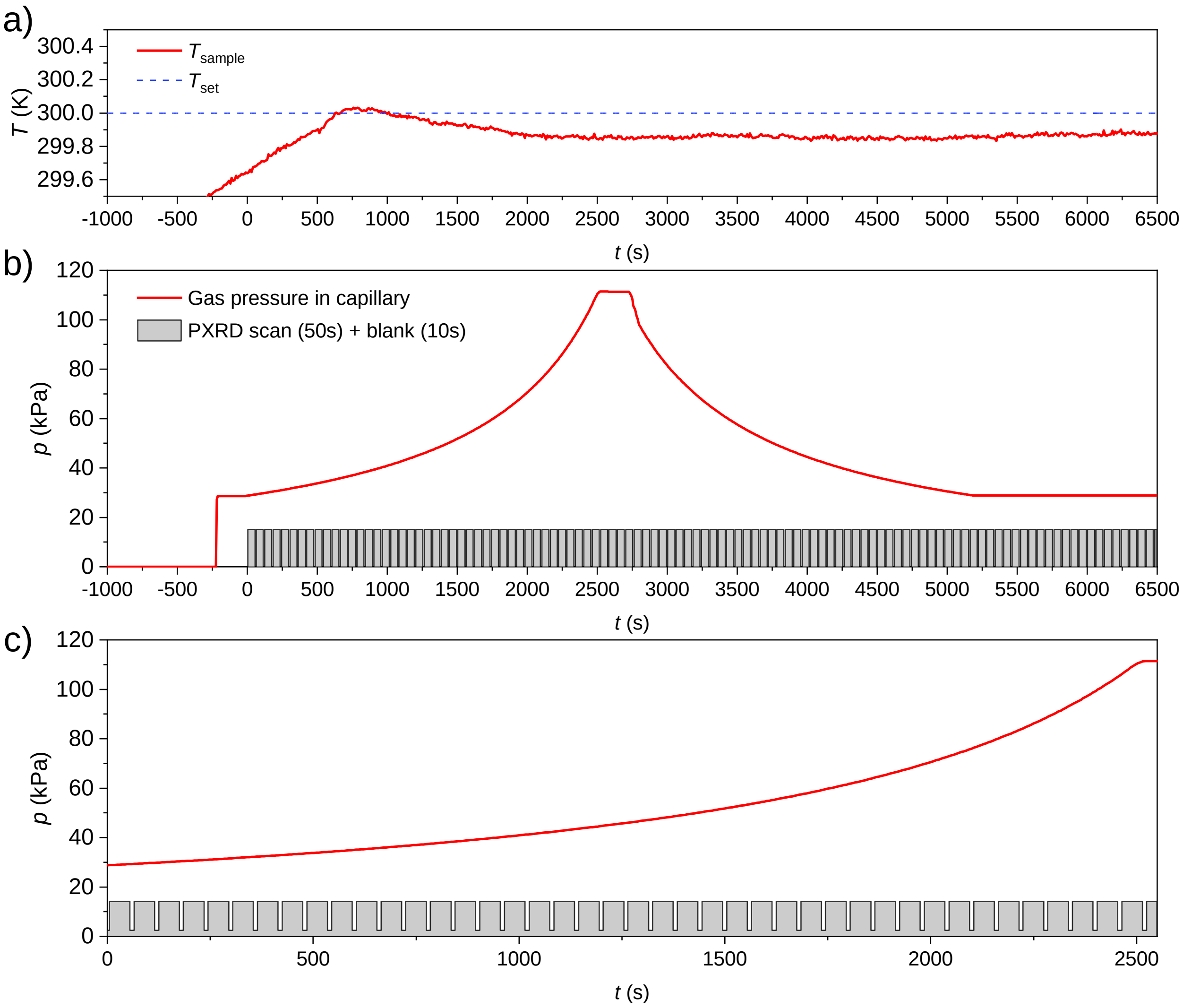

Supplementary Figure 63. a) Time-dependent temperature evolution during the experiment, b) time-dependent pressure evolution during *in situ* PXRD experiment Cap-11-12 (Supplementary Table 2) and c) magnified region during adsorption. Grey area indicates exposure period of detector for recording of diffraction images.

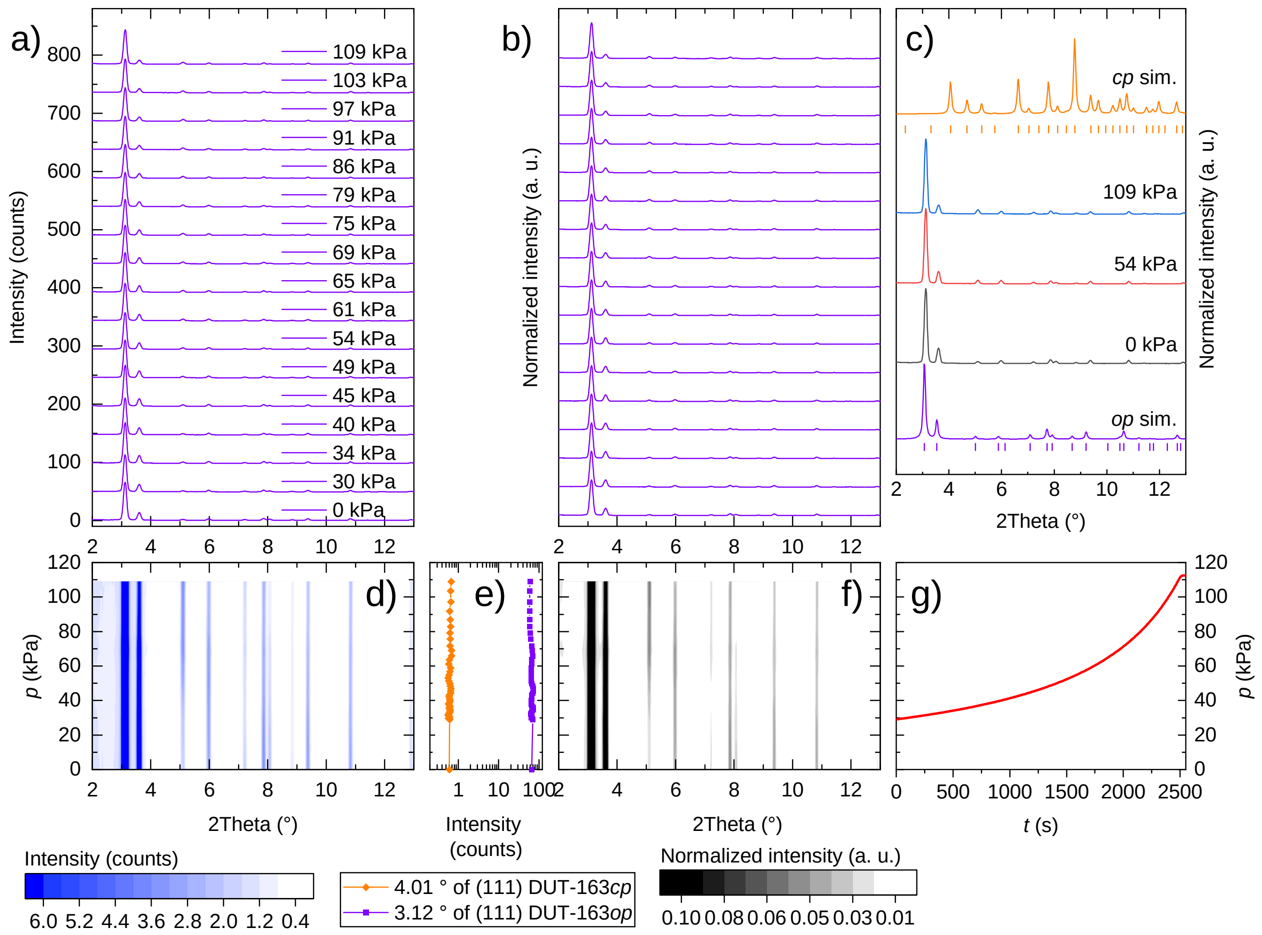

Supplementary Figure 64. a-b) PXRD patterns recorded at 300 K with increasing pressure of MP under light exclusion (experiment CAP11). c) selected PXRD patterns in comparison with simulated PXRD patterns of DUT-163*op* (purple) and DUT-163*cp* (orange) and theoretical peak positions as vertical lines. d,f) Contour plots based on PXRD data with absolute intensity (a,d) and normalized to the highest intensity (b,f). e) Intensity evolution at the peak positions of the (111) reflections of DUT-163*op* (purple) and DUT-163*cp* (orange). g) Evolution of MP pressure during the experiment.

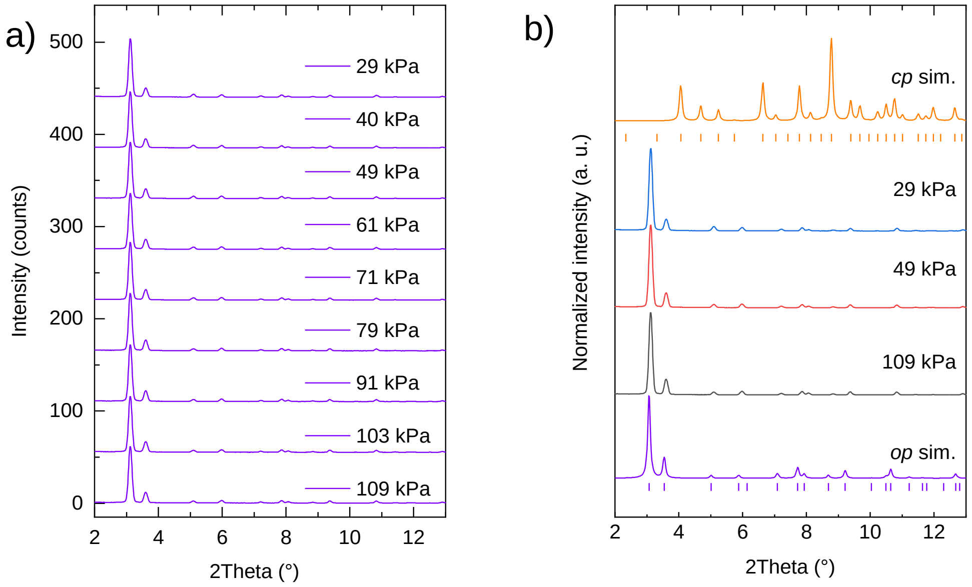

Supplementary Figure 65. a,b) PXRD patterns recorded at 300 K at different pressure of MP during desorption with no irradiation (experiment CAP12 Supplementary Table 2). b) selected PXRD patterns in comparison with simulated PXRD patterns of DUT-163*op* (purple) and DUT-163*cp* (orange) and theoretical peak positions as vertical lines.

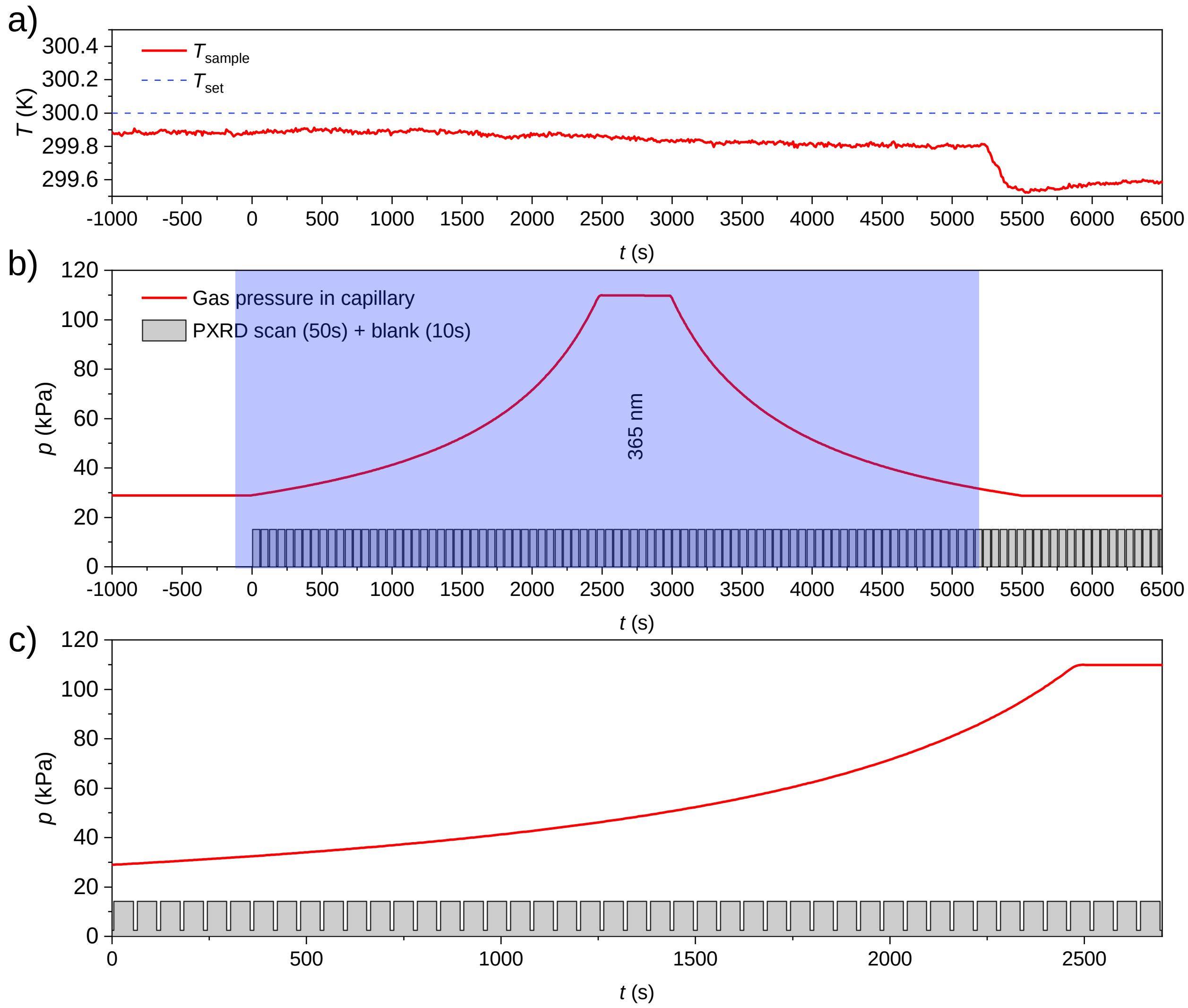

Supplementary Figure 66. a) Time-dependent temperature evolution during the experiment, b) time-dependent pressure evolution during *in situ* PXRD experiment Cap-13 (Supplementary Table 2) and c) magnified region during adsorption. Grey area indicates exposure period of detector for recording of diffraction images.

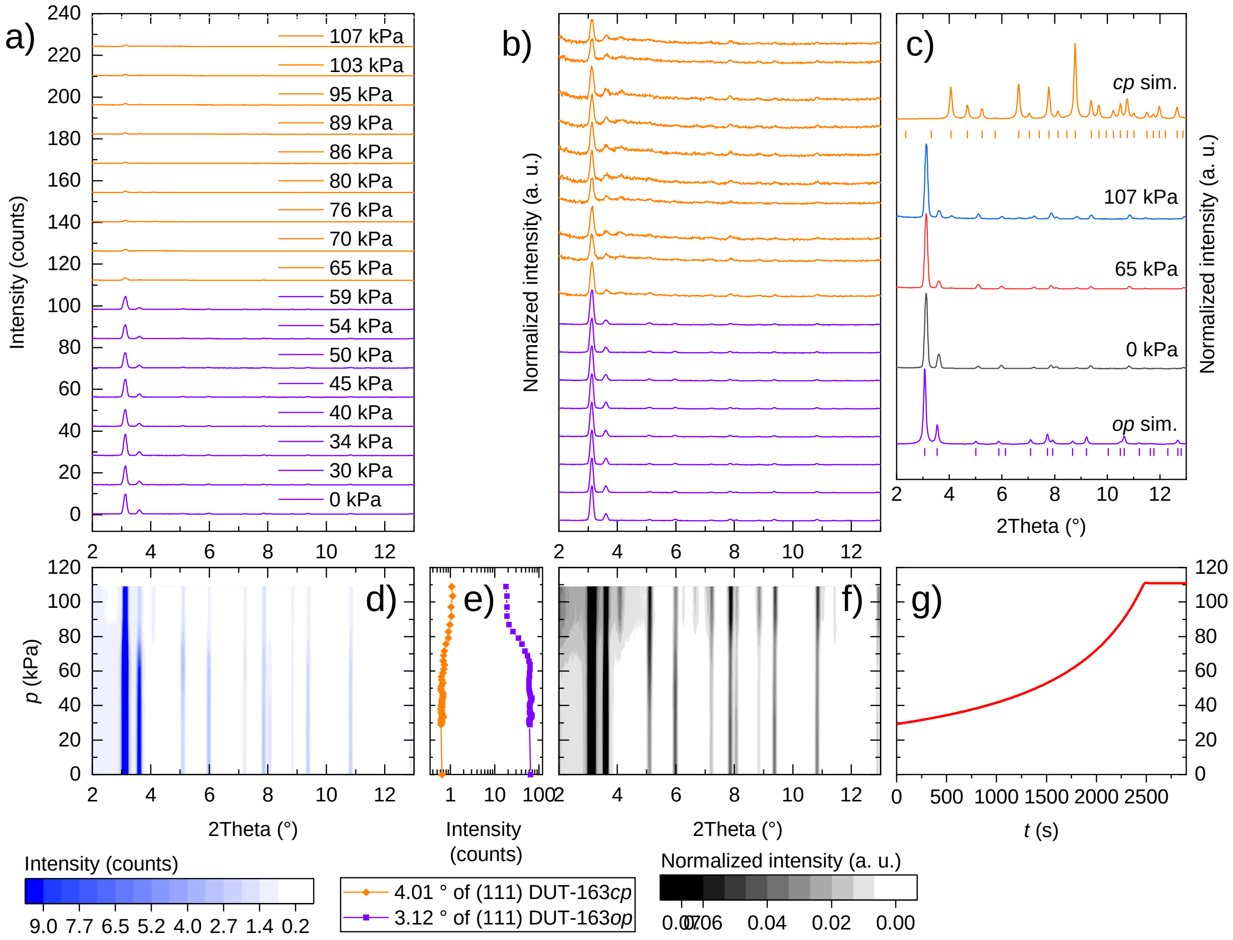

Supplementary Figure 67. a-b) PXRD patterns recorded at 300 K with increasing pressure of MP and irradiation at 365 nm (experiment CAP13). c) selected PXRD patterns in comparison with simulated PXRD patterns of DUT-163*op* (purple) and DUT-163*cp* (orange) and theoretical peak positions as vertical lines. d,f) Contour plots based on PXRD data with absolute intensity (a,d) and normalized to the highest intensity (b,f). e) Intensity evolution at the peak positions of the (111) reflections of DUT-163*op* (purple) and DUT-163*cp* (orange). g) Evolution of MP pressure during the experiment.

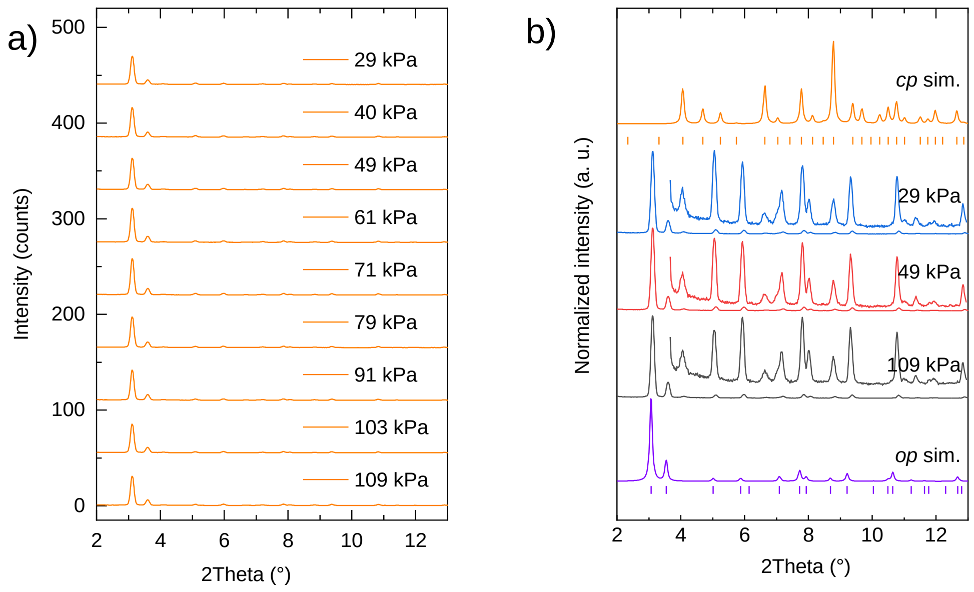

Supplementary Figure 68. a,b) PXRD patterns recorded at 300 K at different pressure of MP during desorption with irradiation at 365 nm (experiment CAP14 Supplementary Table 2). b) selected PXRD patterns in comparison with simulated PXRD patterns of DUT-163*op* (purple) and DUT-163*cp* (orange) and theoretical peak positions as vertical lines.

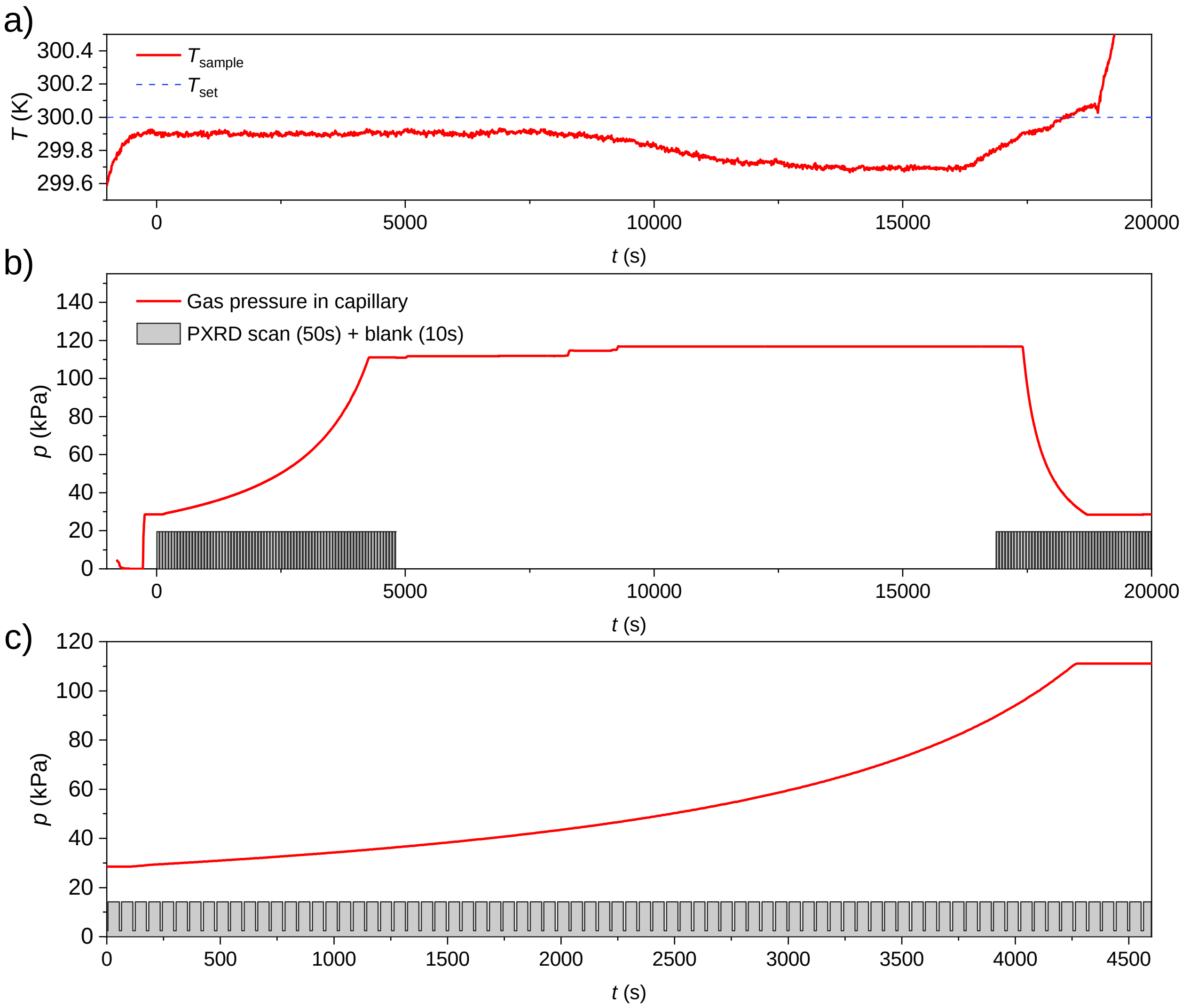

Supplementary Figure 69. a) Time-dependent temperature evolution during the experiment, b) time-dependent pressure evolution during *in situ* PXRD experiment (Cap-15 Supplementary Table 2) and c) magnified region during adsorption. Grey area indicates exposure period of detector for recording of diffraction images.

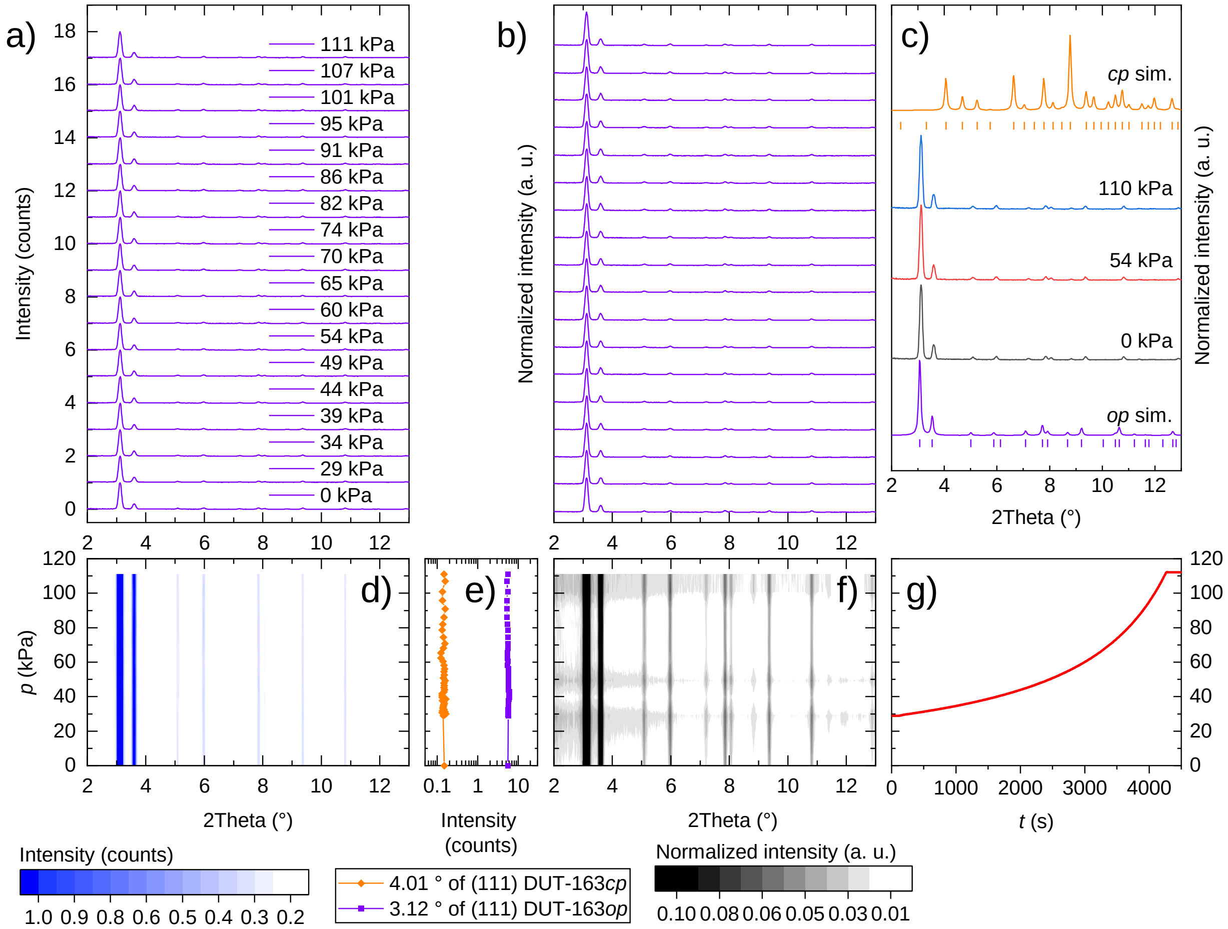

Supplementary Figure 70. a-b) PXRD patterns recorded at 300 K with increasing pressure of MP without irradiation (experiment CAP-14 Supplementary Table 2). c) selected PXRD patterns in comparison with simulated PXRD patterns of DUT-163*op* (purple) and DUT-163*cp* (orange) and theoretical peak positions as vertical lines. d,f) Contour plots based on PXRD data with absolute intensity (a,d) and normalized to the highest intensity (b,f). e) Intensity evolution at the peak positions of the (111) reflections of DUT-163*op* (purple) and DUT-163*cp* (orange). g) Evolution of MP pressure during the experiment.

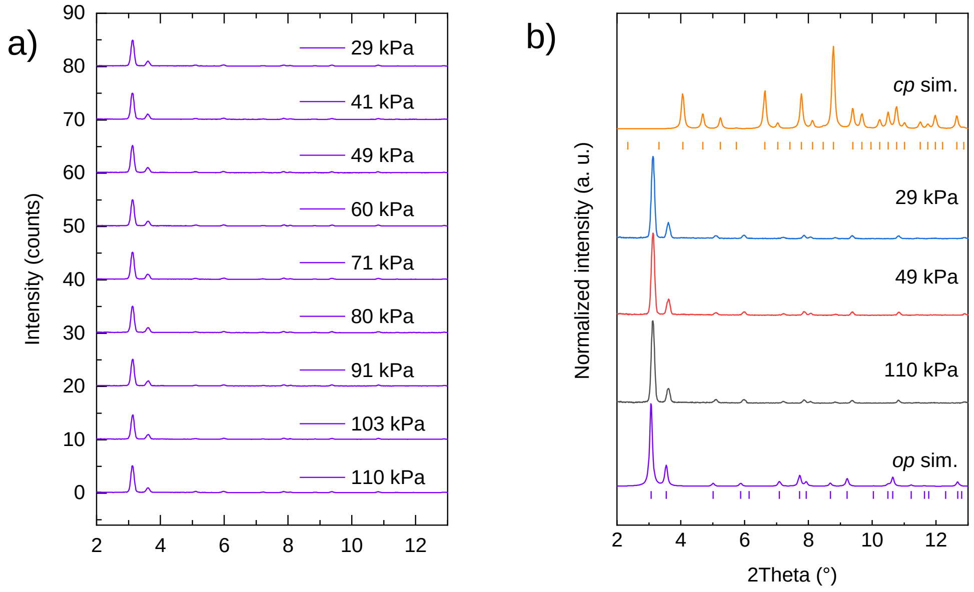

Supplementary Figure 71. a,b) PXRD patterns recorded at 300 K at different pressure of MP during desorption without irradiation (experiment CAP15 Supplementary Table 2). b) selected PXRD patterns in comparison with simulated PXRD patterns of DUT-163*op* (purple) and DUT-163*cp* (orange) and theoretical peak positions as vertical lines.

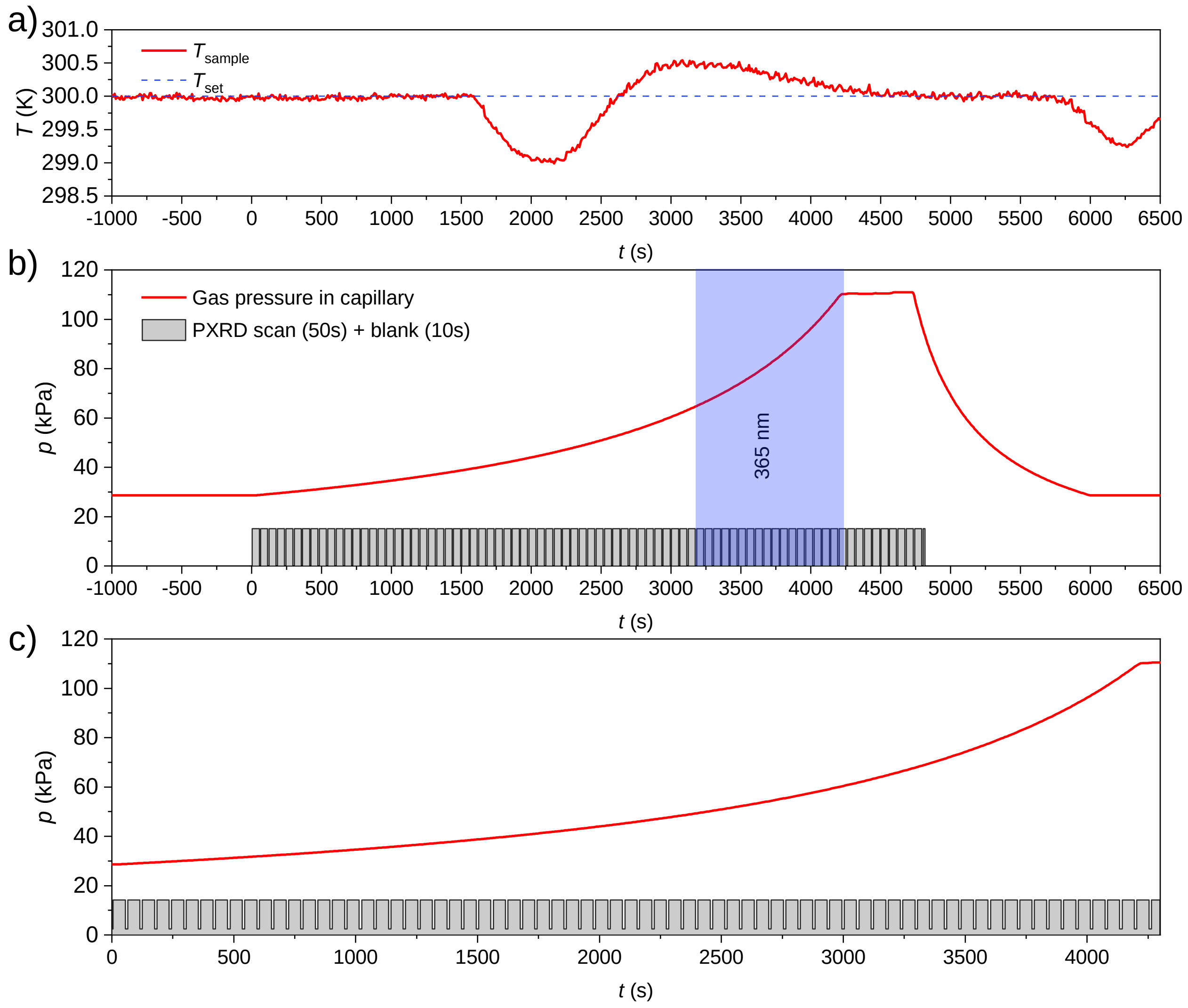

Supplementary Figure 72. a) Time-dependent temperature evolution during the experiment, b) time-dependent pressure evolution during *in situ* PXRD experiment (Cap-16 Supplementary Table 2) and c) magnified region during adsorption. Grey area indicates exposure period of detector for recording of diffraction images.

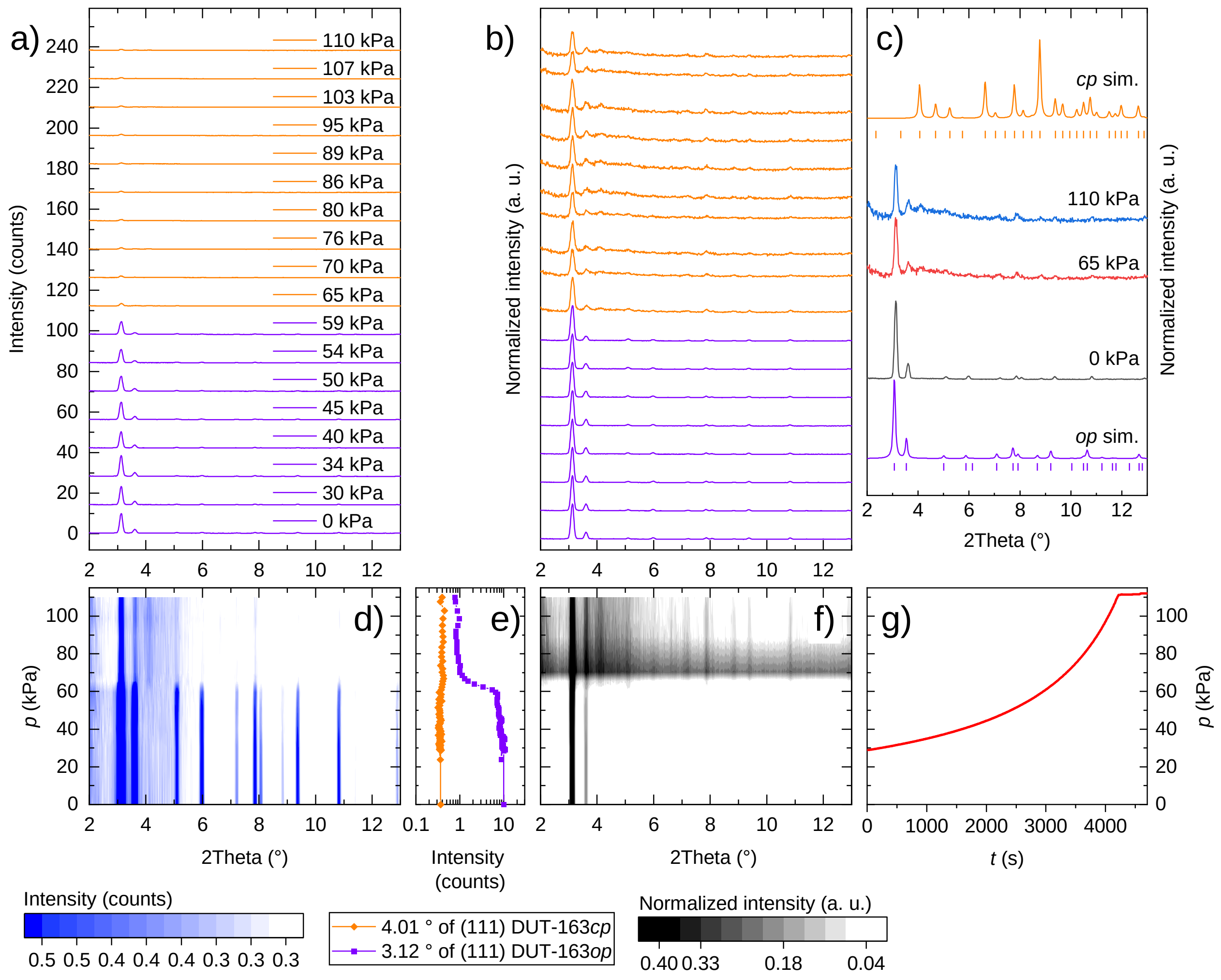

Supplementary Figure 73. a-b) PXRD patterns recorded at 300 K with increasing pressure of MP with partial irradiation at 365 nm (experiment CAP-16 Supplementary Table 2). c) selected PXRD patterns in comparison with simulated PXRD patterns of DUT-163*op* (purple) and DUT-163*cp* (orange) and theoretical peak positions as vertical lines. d,f) Contour plots based on PXRD data with absolute intensity (a,d) and normalized to the highest intensity (b,f). e) Intensity evolution at the peak positions of the (111) reflections of DUT-163*op* (purple) and DUT-163*cp* (orange). g) Evolution of MP pressure during the experiment.

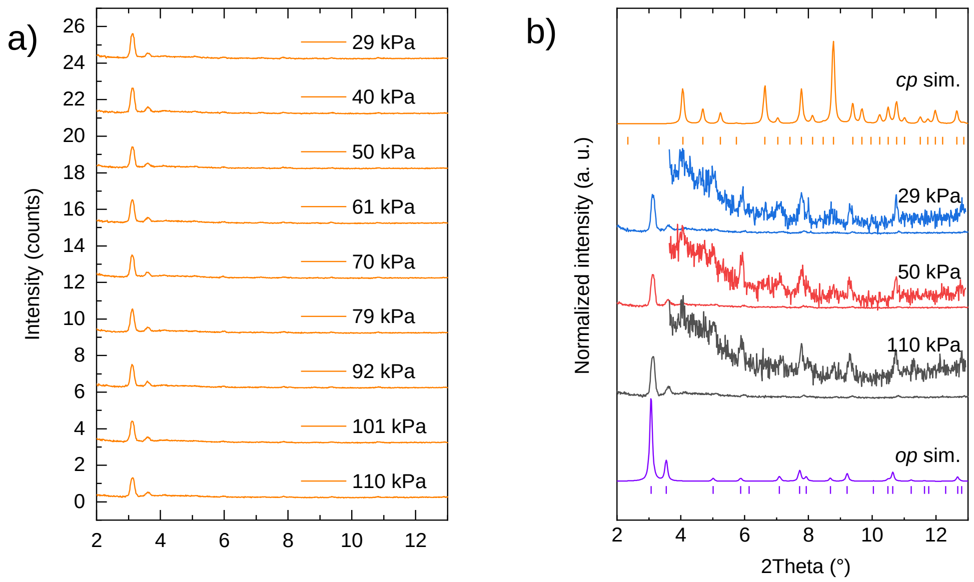

Supplementary Figure 74. a,b) PXRD patterns recorded at 300 K at different pressure of MP during desorption without irradiation (experiment CAP17 Supplementary Table 2). b) selected PXRD patterns in comparison with simulated PXRD patterns of DUT-163*op* (purple) and DUT-163*cp* (orange) and theoretical peak positions as vertical lines.

To investigate the local initiation of structural contraction by irradiation a series of diffraction experiments was performed in which the capillary was scanned axially by successively moving the capillary in and out of the sample holder and recording PXRD patterns as a function of capillary position. Here, 0 mm represents the front end of the capillary. This allows to generate an axial profile of the phase composition of DUT-163 inside the capillary for the three in situ PXRD experiments which are displayed in Supplementary Figure 75-Supplementary Figure 77.

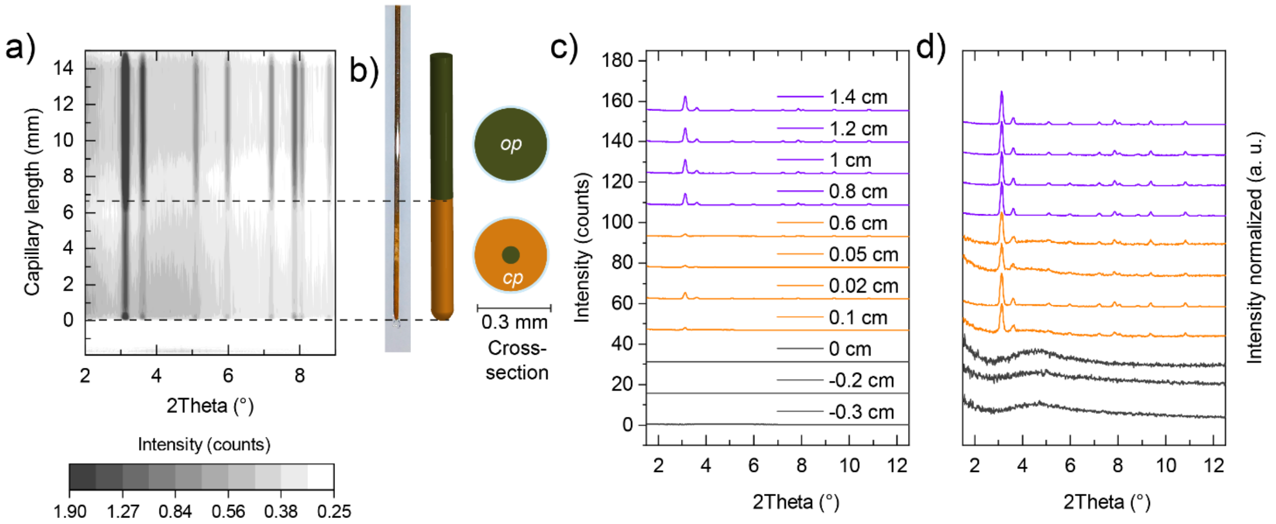

Supplementary Figure 75. Sample investigated after experiment CAP17 Supplementary Table 2 a) Contour plot based on PXRD data with absolute intensity obtained as a function of capillary length, b) image of the capillary investigated (Due to the smaple contraction the sample bad actualy exhibits gaps in the lower section) and a cartoon that displays the phase composition, c) PXRD patterns that make up a), and d) PXRD patterns plotted with intensity normalized to the highest value.

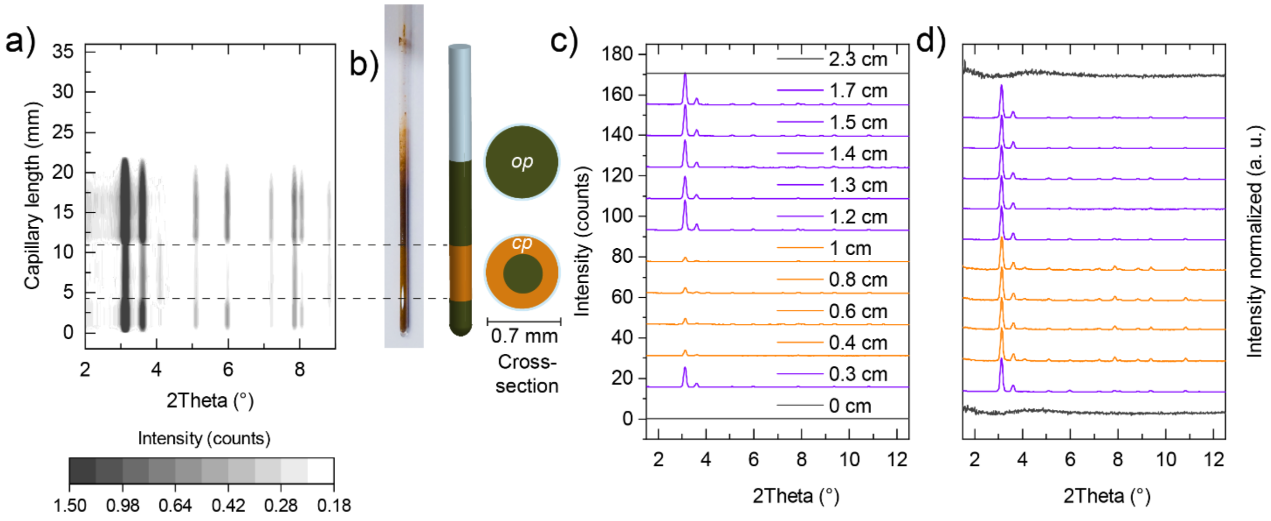

Supplementary Figure 76. Sample investigated after experiment CAP10 Supplementary Table 2 a) Contour plot based on PXRD data with absolute intensity obtained as a function of capillary length, b) image of the capillary investigated (Due to the smaple contraction the sample bad actualy exhibits gaps in the lower section) and a cartoon that displays the phase composition, c) PXRD patterns that make up a), and d) PXRD patterns plotted with intensity normalized to the highest value.

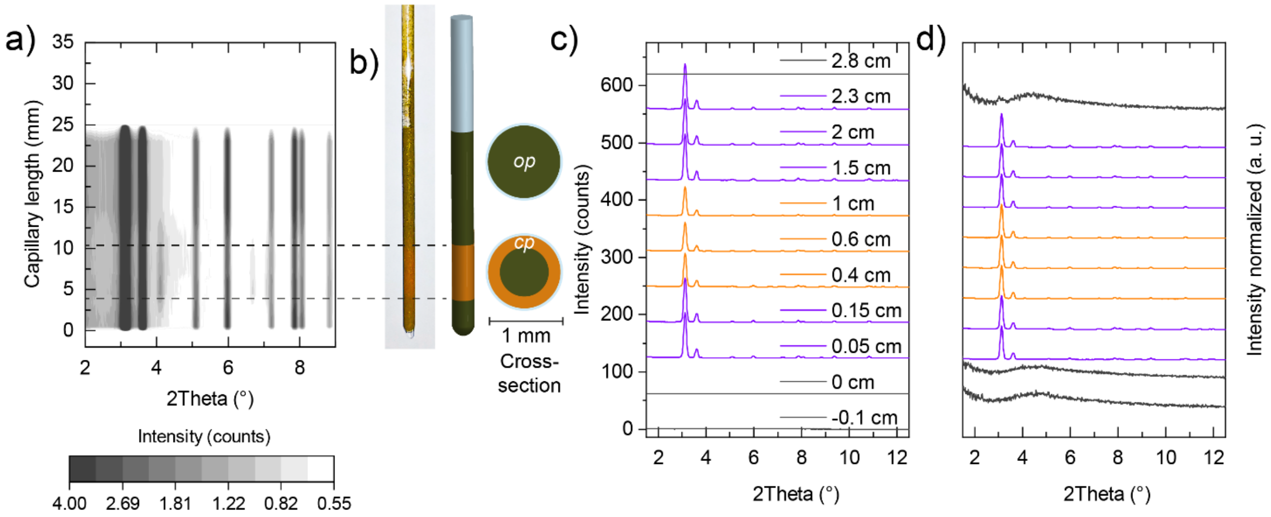

Supplementary Figure 77. Sample investigated after experiment CAP13 Supplementary Table 2 a) Contour plot based on PXRD data with absolute intensity obtained as a function of capillary length, b) image of the capillary investigated (Due to the smaple contraction the sample bad actualy exhibits gaps in the lower section) and a cartoon that displays the phase composition, c) PXRD patterns that make up a), and d) PXRD patterns plotted with intensity normalized to the highest value.

13) Rietveld refinement of PXRD data

To obtain the crystal structure of DUT-163*cp* we analyzed *in situ* PXRD patterns, measured in the thermodynamic equilibria at 20 kPa during the adsorption of methylpropane at 261 K, by Rietveld method. The refinement was performed using the Reflex tool of Materials Studio 5.0. A structural model of the (*E*)-DUT-163*cp* that contains (*E*)-dacdc was generated in the space group $Pa\bar{3}$. Paddle-wheel units, carbazoles, phenyl rings, nitrogen atoms of the diazo group, and MP molecules in the pores were defined as rigid bodies, respectively. Rietveld refinement with energy option (contribution of UFF ~1%) was used in the structure

refinement to maintain framework connectivity. The final structural parameters are given in the Supplementary Table 4 and the corresponding Rietveld plot is shown in the Supplementary Figure 78.The crystal structure of (*E*)-DUT-163*cp* is submitted in CSD database and available under CCDC-2040811.

It must be noted that diffraction patterns of DUT-163*cp* exhibit severe peak broadening and a drastic reduction in peak intensity. The origin for this loss in diffraction intensity is based on the presence of non-ordered gas molecules in the pores and the structural disorder of the cp phase due to the highly strained ligand. As a result quantitative Rietveld analysis, in particular of diffraction patterns that contain mixtures of *op*-*cp* phases.

Supplementary Table 4. Experimental data on Rietveld refinement of DUT-163cp.

	**DUT-163*cp* ⊃ 216 *i*-C_4H_{10}**
	Supplementary Figure 78
Formula unit	$C_{40}H_{20}Cu_2N_4O_8$ ⊃ 9 *i*-C_4H_{10}
Z	24
Symmetry, space group	cubic, $Pa\bar{3}$
Unit cell parameter, *a* (Å)	37.55861
Unit cell volume, (Å^3)	126200
Wave length (Å)	1.5406
2θ range (°)	3.8 - 38
Instrument geometry	Bragg-Brentano
Zero point line shift (°)	0
Profile function	Thompson-Cox-Hastings
U	0.47739
V	0.01091
W	0.00167
X	0.37449
Y	0.10656
Asymmetry correction	Berar-Baldinozzi
P1	-0.00606
P2	0.00267
P3	-0.00073
P4	0.00006
Final R_{wp}	0.0745
Final R_p	0.0565

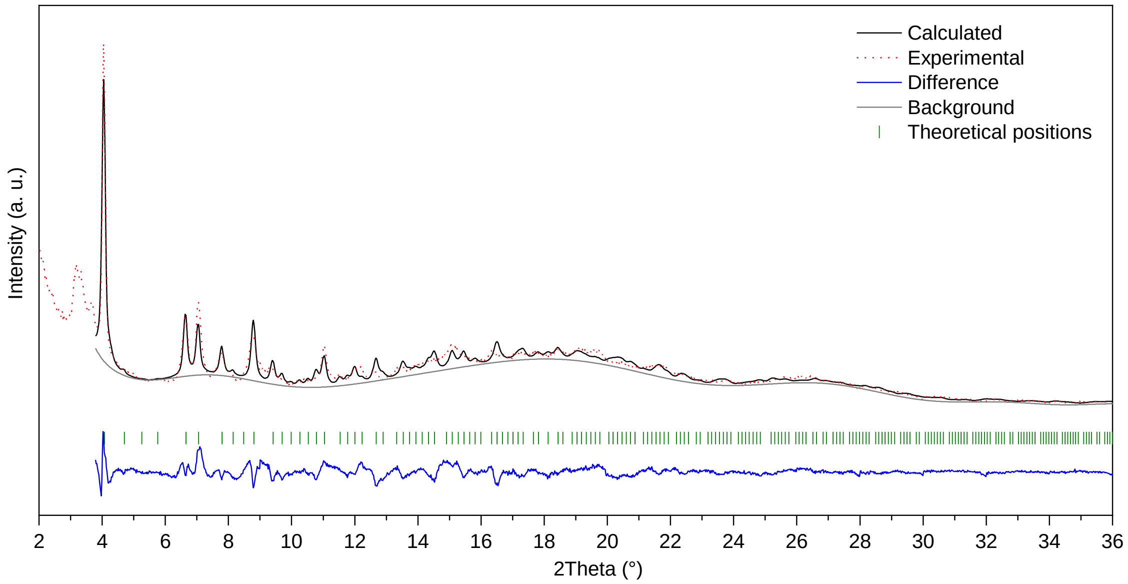

Supplementary Figure 78. Rietveld plot of refinement of DUT-163. Experimental PXRD pattern was obtained upon adsorption of MP at 261 K and 20 kPa. Low angle region 2-3.8° 2Theta was omitted from refinement due to residual reflections from *op* phase. Theoretical PXRD pattern of DUT-163cp does not contain reflections below 4° 2Theta.

Attempts to perform

14) *In situ* UV/Vis spectroscopy of solid MOF samples

Solid state UV/Vis spectra were recorded on a VARIAN CARY 4000. For DUT-49 0.5-2 mg of sample were mixed with 20-35 mg dry $BaSO_4$ and pressed in the sample cell under inert atmosphere in an argon filled glove box. Alternatively, due to the low mechanical stability of DUT-163, a thin film of powder of the MOF was placed on top of a $BaSO_4$ bed which was previously compressed. To analyze MOF samples under inert atmosphere and *in situ* under various concentrations of *n*-butane, a HARRICK Praying Mantis reaction chamber with a dome with UV/Vis-transparent quartz windows and a third window for external irradiation. For the analysis of *n*-butane adsorption, the gas composition was controlled via two mass flow controllers (MFC) and *n*-butane was mixed with non-adsorbing nitrogen in defined ratios under a constant flow of 50 ml min^{-1}. If not otherwise noted samples were equilibrated for at least 10 min when gas loading was changed. DUT-49 was investigated as a reference material.

Supplementary Table 5. Summary of *in situ* solid state UV/Vis experiments.

Experiment ID	Material	Figure	*T* (K)	Excitation wavelength (nm)	Gas loading* (Vol-%)
UV/Vis1	DUT-49	Supplementary Figure 79	297	No irradiation	0-100
UV/Vis2	DUT-49	Supplementary Figure 81	297	365	
UV/Vis3	DUT-49	Supplementary Figure 82	297	365	100
UV/Vis4	DUT-163	Supplementary Figure 83	297	No irradiation	0-100
UV/Vis5	DUT-163	Supplementary Figure 84	297	365-455	
UV/Vis6	DUT-163	Supplementary Figure 84	297	365	100
UV/Vis6	DUT-163	Supplementary Figure 85	300	365	100#
UV/Vis6	DUT-163	Supplementary Figure 85	300	365	100#

* samples were analyzed in a constant flow of gas with 50 ml min^{-1}, Vol.-% given as *n*-butane concentration in nitrogen.

# 2-Methylpropane was used instead of *n*-butane.

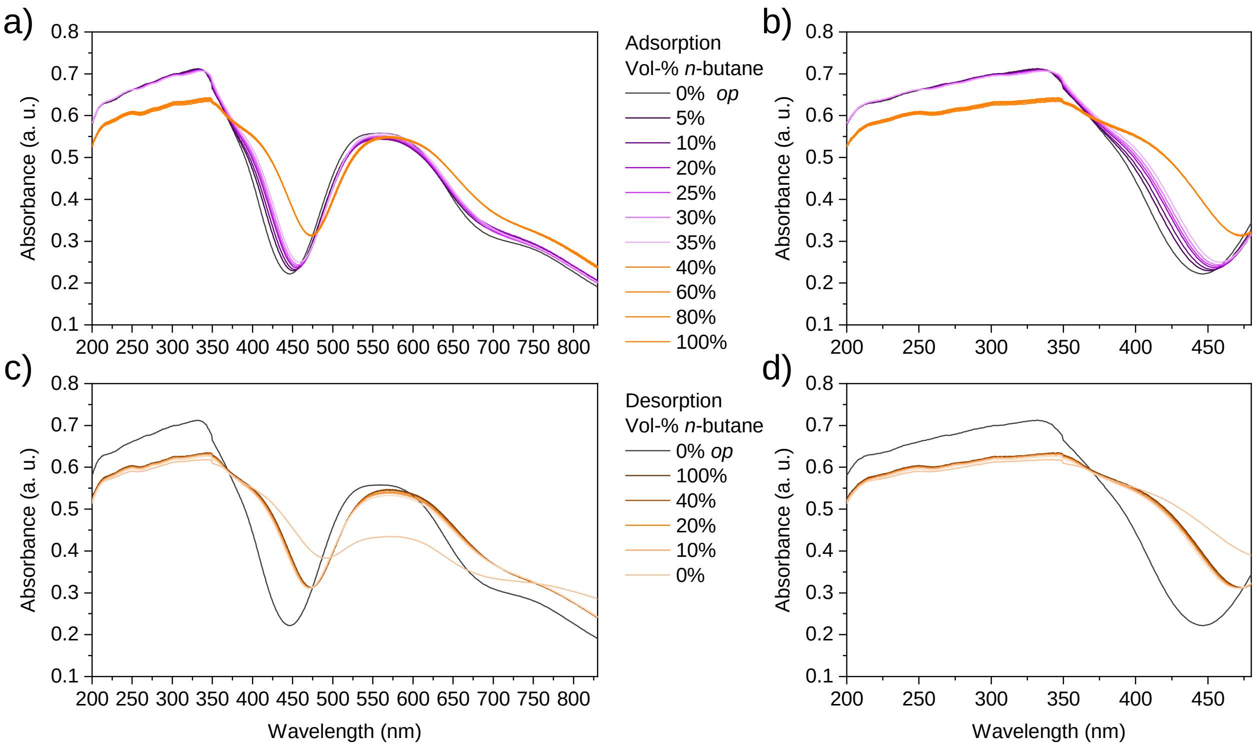

Supplementary Figure 79. a,b) UV-Vis absorption spectra of DUT-49 with increased loading of *n*-butane (adsorption process). a,b) UV-Vis absorption spectra of DUT-49 with increased loading of *n*-butane (adsorption process), c,d) UV-Vis absorption spectra of DUT-49 with decreasing loading of *n*-butane (desorption process). Full spectrum (a) and UV-region (b).

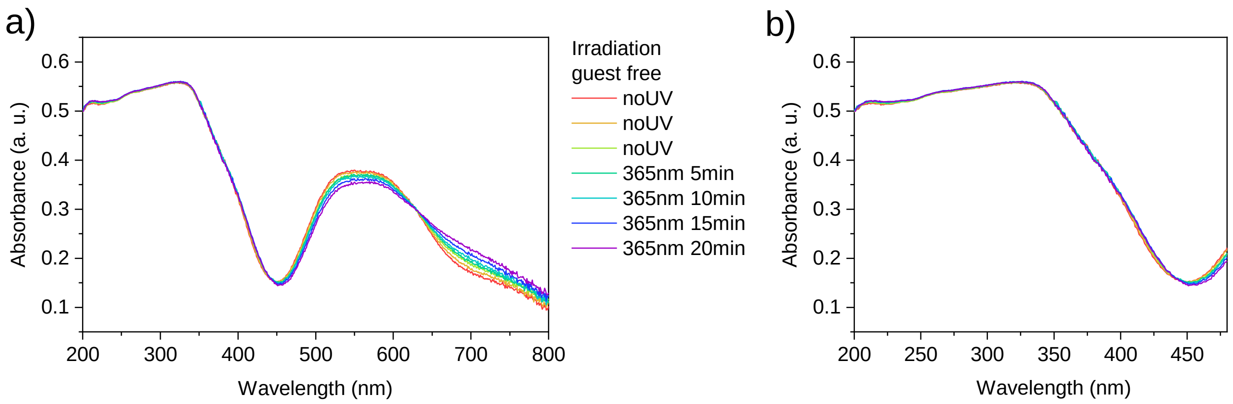

Supplementary Figure 80. a,b)UV-Vis absorption spectra of DUT-49 under nitrogen atmosphere during irradiation with 365 nm, full spectrum (a) and UV-region (b).

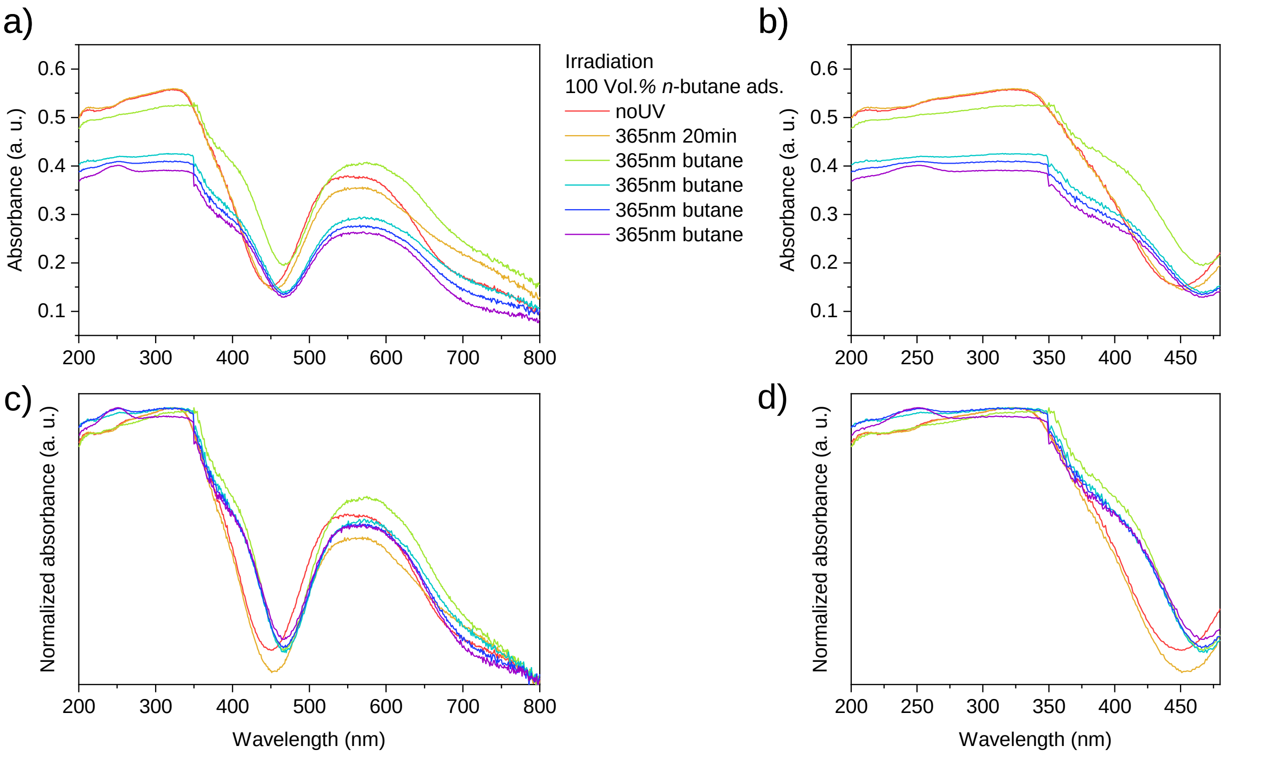

Supplementary Figure 81. a,b) Absolute UV-Vis absorption spectra of DUT-49 under nitrogen and *n*-butane atmosphere during irradiation with 365 nm. c,d) Spectra normalized to the highest absorptivity. Full spectrum (a,c) and UV-region (b,d).

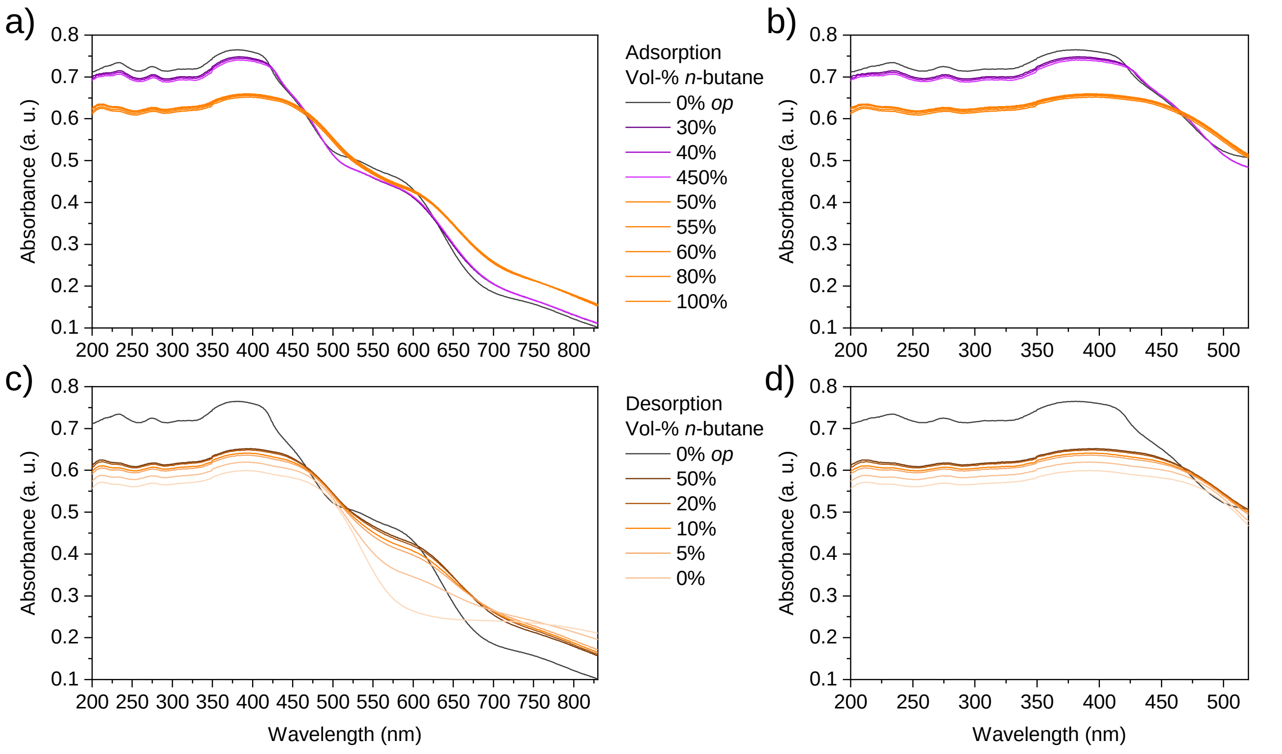

Supplementary Figure 82. a,b) UV-Vis absorption spectra of DUT-163 with increased loading of *n*-butane (adsorption process). a,b) UV-Vis absorption spectra of DUT-163 with increased loading of *n*-butane (adsorption process), c,d) UV-Vis absorption spectra of DUT-49 with decreasing loading of *n*-butane (desorption process). Full spectrum (a) and UV-region (b).

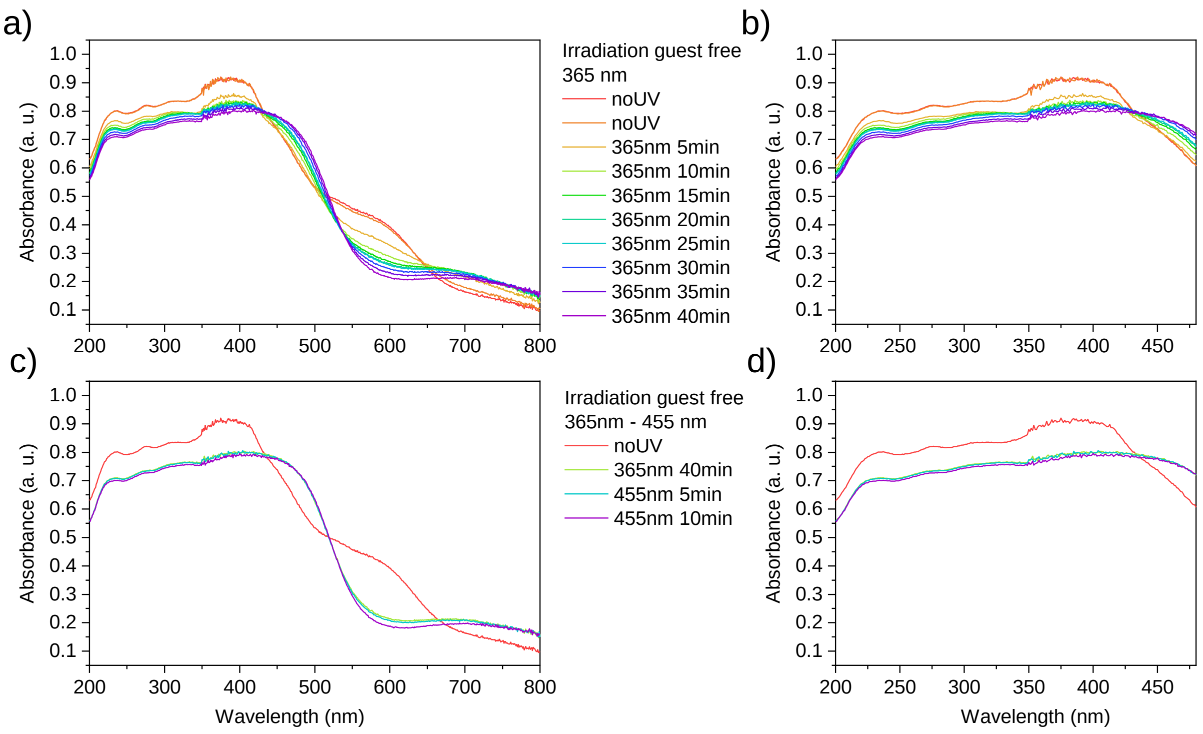

Supplementary Figure 83. a,b) UV-Vis absorption spectra of DUT-163 under nitrogen atmosphere during irradiation with 365 nm. c,d) UV-Vis absorption spectra of DUT-163 under nitrogen atmosphere during irradiation with 455 nm conduced after irradiation with 365 nm. Full spectrum (a,c) and UV-region (b,d).

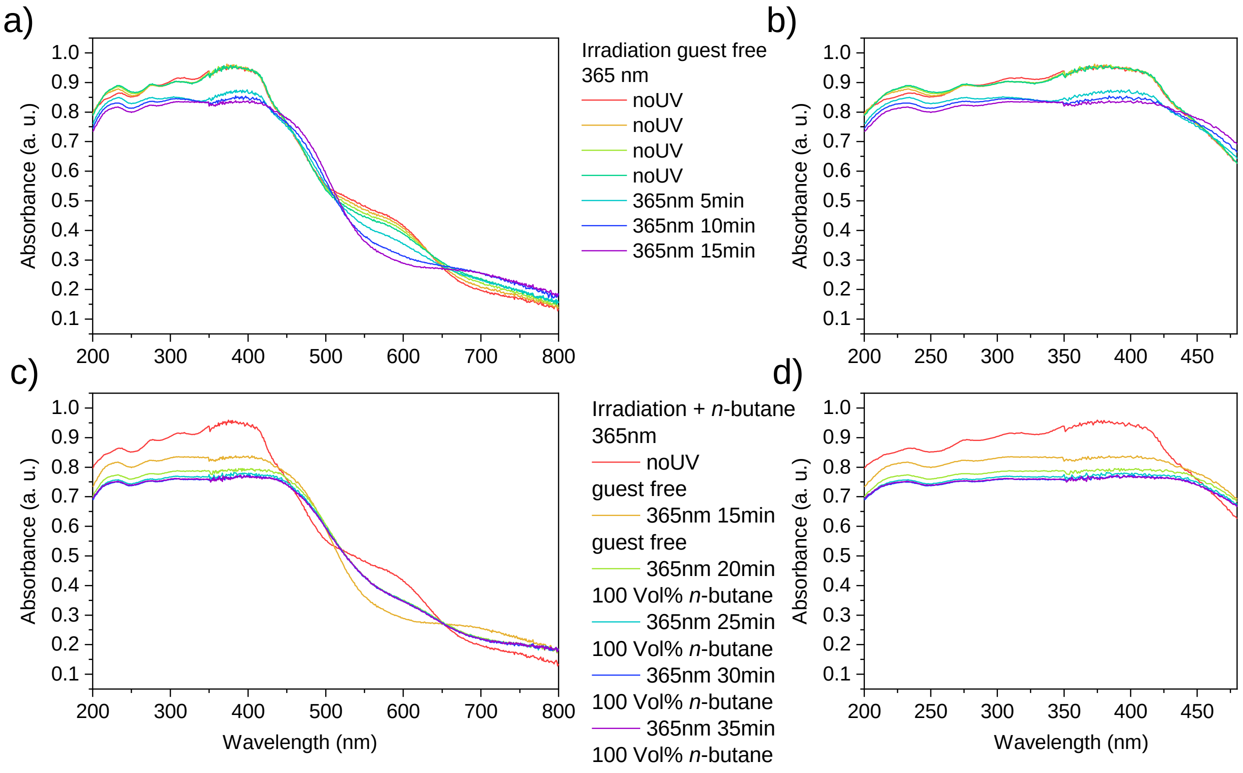

Supplementary Figure 84. a,b) UV-Vis absorption spectra of DUT-163 under nitrogen atmosphere during irradiation with 365 nm (different sample than in Supplementary Figure 83). c,d) UV-Vis absorption spectra of DUT-163 under nitrogen and n-butane atmosphere during irradiation with 365 nm. Full spectrum (a,c) and UV-region (b,d).

To recreate the sample environment applied in the *in situ* PXRD studies we extended the DR-UV-Vis analysis using 2-Methylpropane as gas and performed the measurement at 300 K. Because the experiments were conducted under gas flow, the feedstock 2-Methylpropane bottle was kept at 301 K guaranteeing equilibrated temperatures of 300±1 K of the sample, crucial for the experiment. A set of two experiments were conducted on two individual samples.

First, DUT-163 was dosed with 2-Methylpropane under exclusion of light (Supplementary Figure 85a,b). Only a very minor chane in the UV-Vis specta is observed indicating the absence of structural transitions. After 20 min of equilibration the sample was irradiatied with 365 nm and a redshift of the spectrum was observed similar to the previous analysis using *n*-butane as gas. This observation indicates structural contraction by parallel application of light and 2-Methylpropane while gas dosing alone at 300 K was insufficient to trigger structural contraction.

In a second experiment on a fresh sample we continuously irradiated the sample before and during the dosing with 2-Methylpropane at 300 K (Supplementary Figure 85c,d). similar to previous observations we identify a decrease in absorbance around 550 nm upon application of 365 nm and a strong redshift upon exposure to 2-Methylpropane. Similar to the first experiment both, only gas dosing and application of light under these conditions yielded structural contraction.

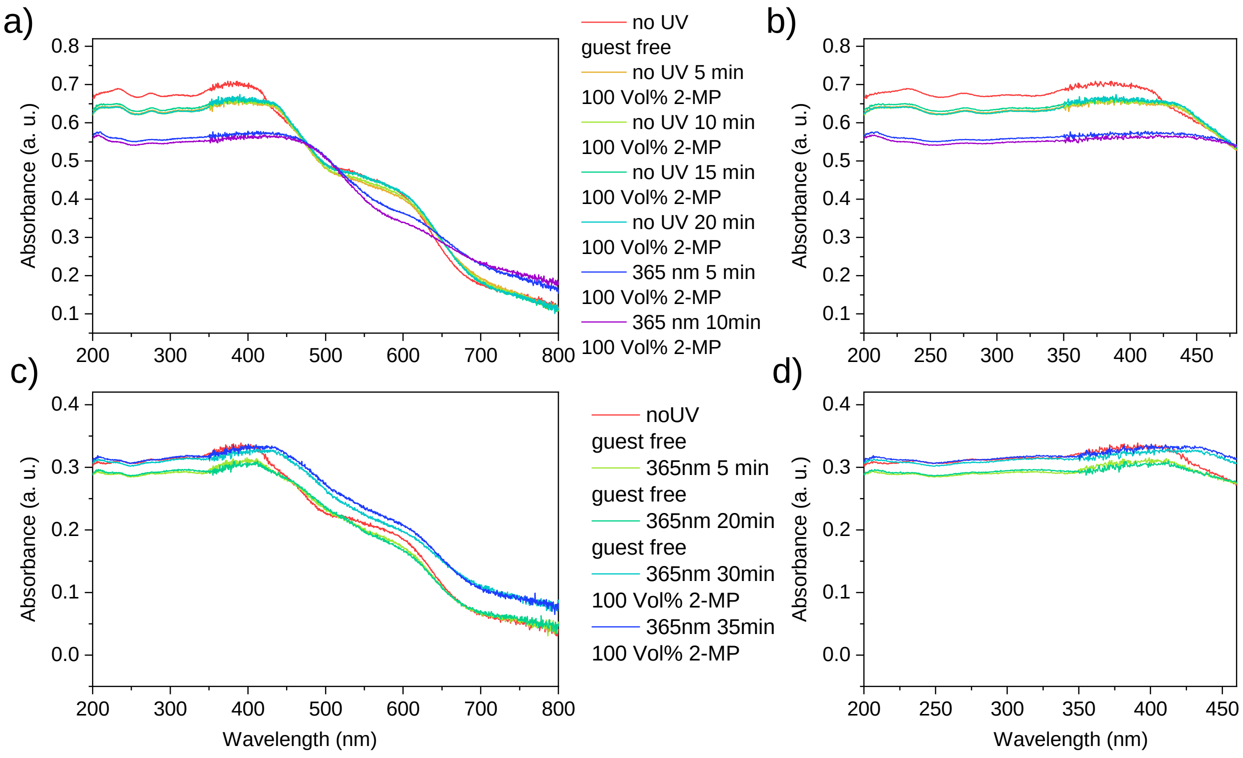

Supplementary Figure 85. a,b) UV-Vis absorption spectra of DUT-163 under nitrogen atmosphere during exposure to 2-methylpropane followed by irradiation with 365 nm under 2-methylpropane atmosphere at 300 K (different sample than in Supplementary Figure 83). c,d) UV-Vis absorption spectra of DUT-163 under nitrogen and n-butane atmosphere during irradiation with 365 nm. Full spectrum (a,c) and UV-region (b,d).

Changes in the overall signal intensity between different loadings can occur due to sample contraction upon structural transitions. This is a common feature that all spectroscopic techniques that rely on a constant exposed area. These effects can to some degree be minimized by normalizing the spectra to the highest intensity. The spectra themselves should not suffer from these effects and shifts in the signal are attributed to changes in the electronic structure rather than the sample are. In some measurements an artificial step in the UV-Vis spectra between 349 and 350 nm is observed which is caused by the instrument due to the change of radiation source. These steps were corrected by aligning the signal intensities <350 nm by subtraction or addition of a constant value in the range of 0.01-0.03 depending on the magnitude of the step.

15) In situ DRIFT spectroscopy

In situ DRIFT spectroscopy on MOF samples was conducted using a HARRICK Praying Mantis reaction chamber with ZnSe windows. Samples were either investigated under inert atmosphere (N_2) or by applying a constant flow of a mixture of inert carrier gas (N_2) and vaporized CCl_4 by bubbling. CCl_4 was selected due to the low absorptivity and small range of IR-active vibrations compared to *n*-butane (Supplementary Figure 84). The temperature of the sample was found to be in the range of 297 K. The composition during *in situ* adsorption of vaporized CCl_4 was estimated by two valves controlling the flux of inert and CCl_4 saturated inert gas and tested on a blank measurement. The concentrations could internally be estimated by the signal intensity in the DRIF spectrum. DUT-49 was investigated as a reference material.

Supplementary Table 6. Summary of *in situ* DRIFTS experiments.

Experiment ID	Material	Figure	*T* (K)	Excitation wavelength (nm)	CCl_4 loading* (Vol-%)
DRIFT1	Blank	Supplementary Figure 84	297	No irradiation	0-60
DRIFT2	DUT-49	Supplementary Figure 79	297	No irradiation	0-80
DRIFT3	DUT-163	Supplementary Figure 81	297	No irradiation	0-80
DRIFT4	DUT-49	Supplementary Figure 82	297	365	0
DRIFT5	DUT-163	Supplementary Figure 83	297	365	0
DRIFT6	DUT-163	Supplementary Figure 84	297	365, 455	0-80
DRIFT7	DUT-163	Supplementary Figure 84	297	365	0-80

* samples were analyzed in a constant flow of vapor/N_2 mixture with ca. 50 ml min^{-1}, Vol.-% given as CCl_4 concentration in nitrogen.

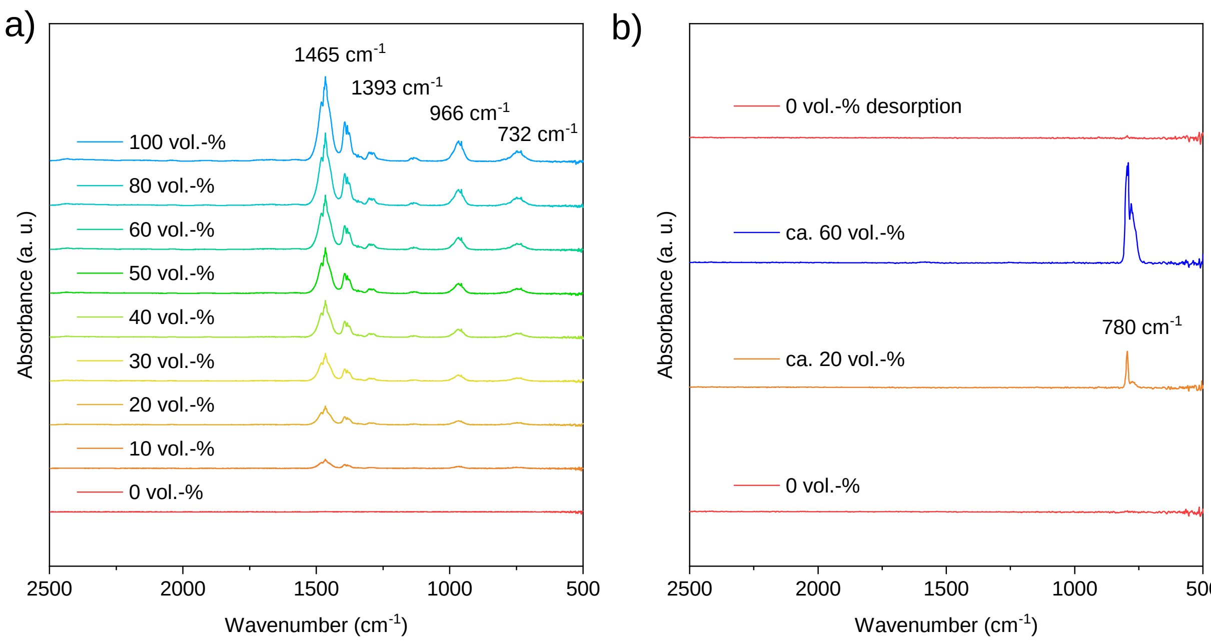

Supplementary Figure 86. DRIFT Spectra of the sample stage at different loadings of a) *n*-butane and b) CCl_4. Figure recreated from reference [1].

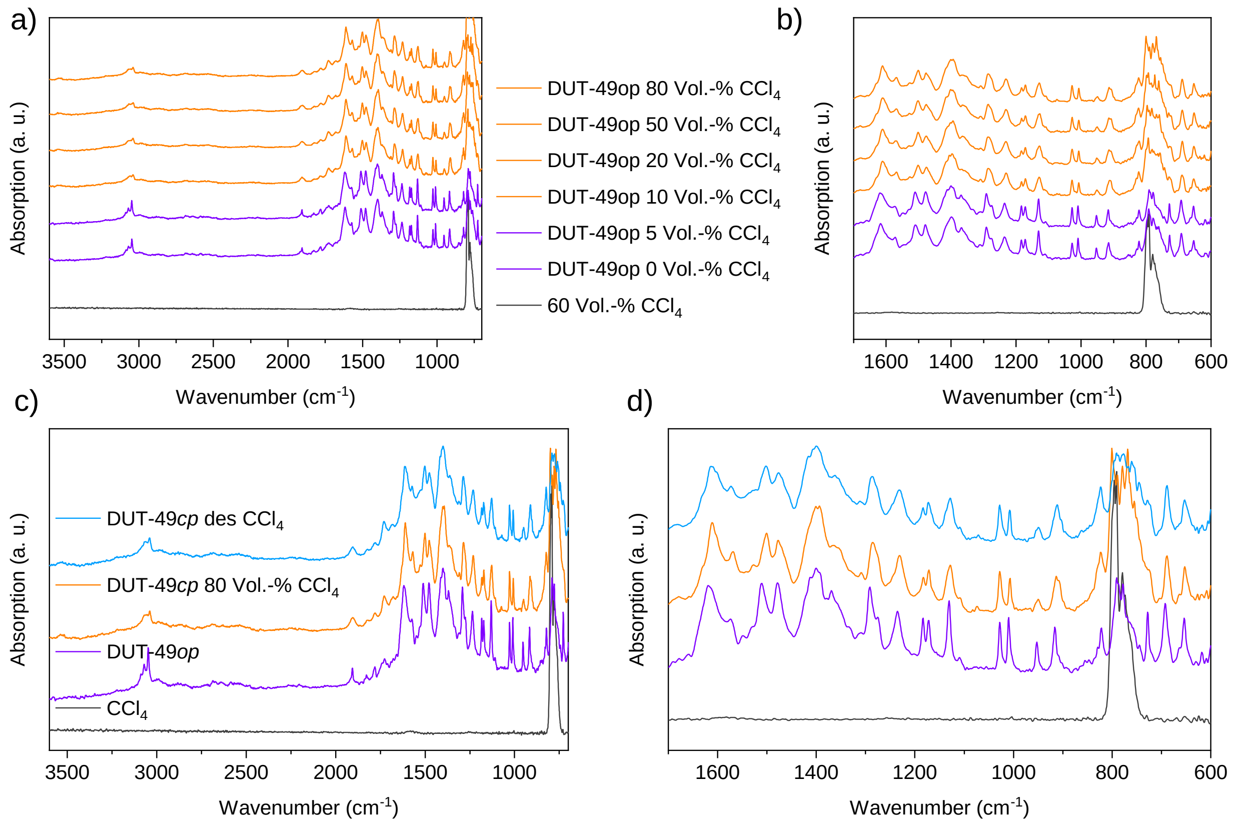

Supplementary Figure 87. a,b) DRIFT spectra of DUT-49 during loading with CCl_4. c,d) DRIFT spectra of DUT-49 under nitrogen and CCl_4 atmosphere during irradiation with 365 nm. Full spectrum (a,c) and magnified region of small wavenumber (b,d). Data was obtained from reference [1]. Comparison to blank 60 vol.-% CCl_4 as reference in black.

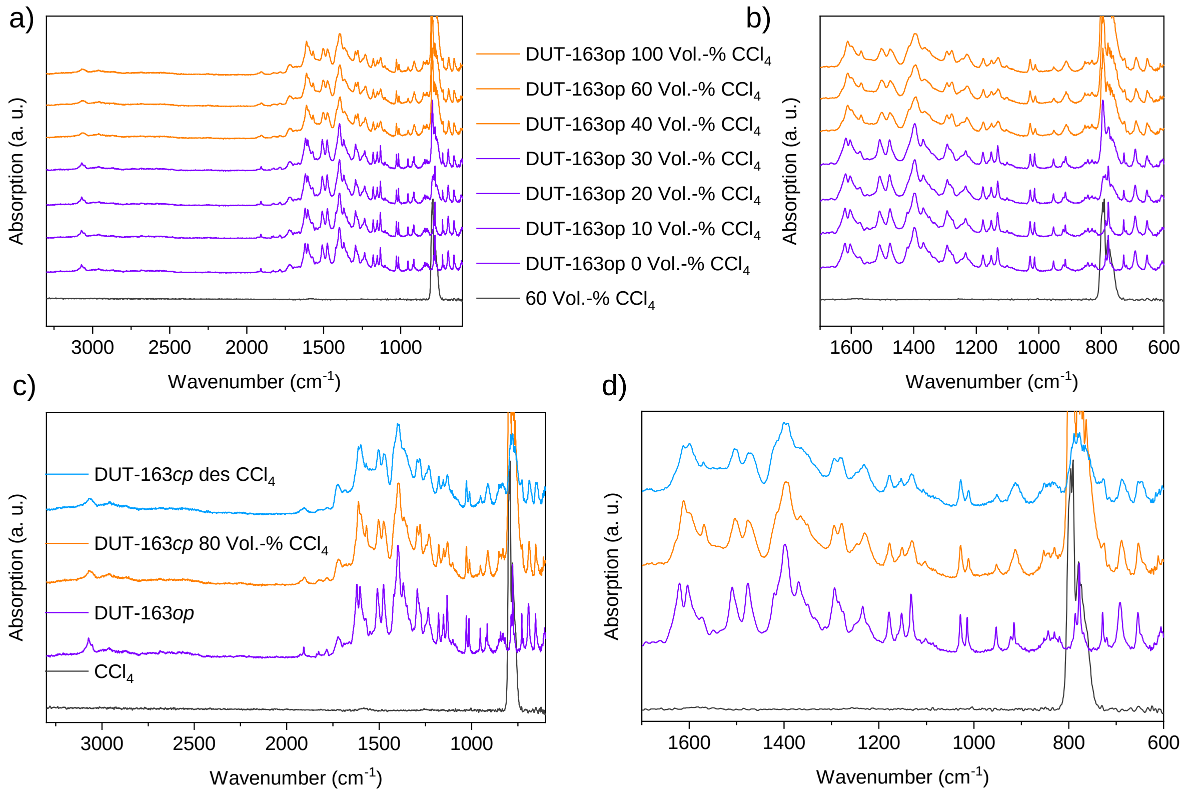

Supplementary Figure 88. a,b) DRIFT spectra of DUT-163 during loading with CCl_4. c,d) selected DRIFT spectra of DUT-163 under nitrogen and CCl_4 atmosphere. Full spectrum (a,c) and magnified region of small wavenumber (b,d). Comparison to blank 60 vol.-% CCl_4 as reference in black.

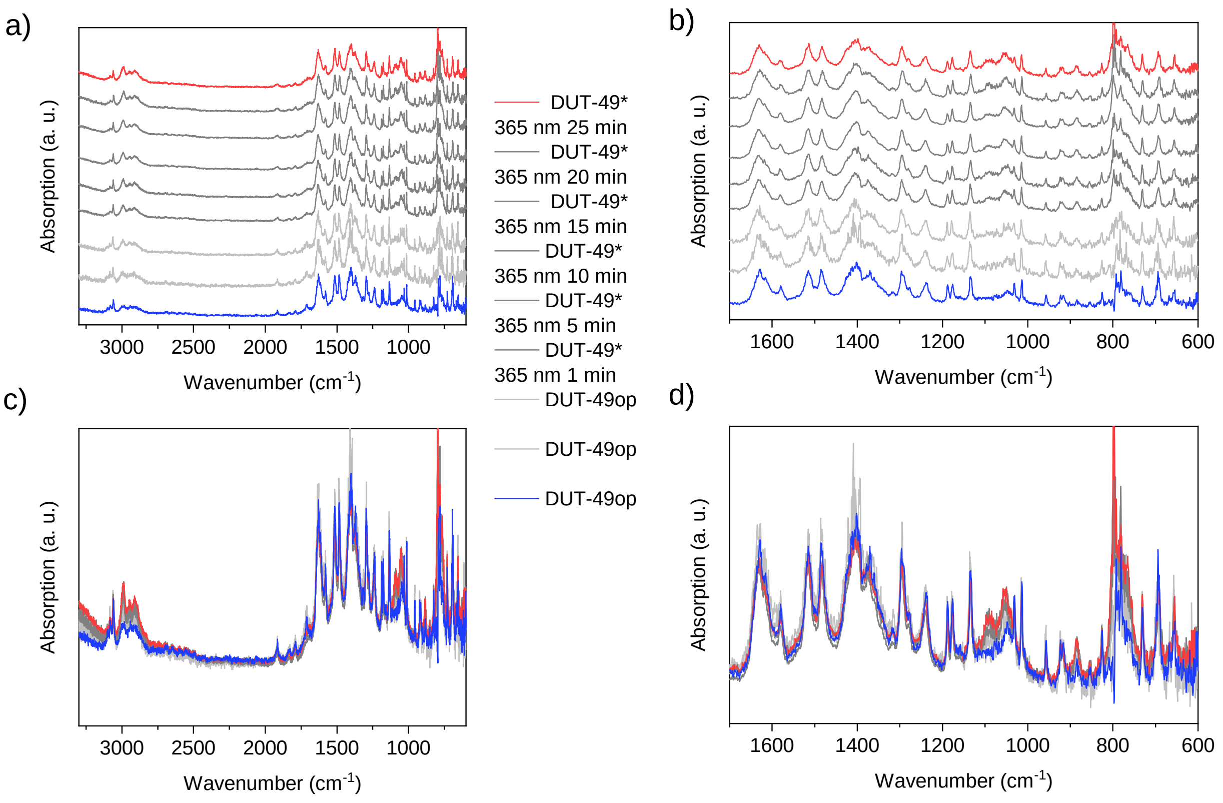

Supplementary Figure 89. a,b) DRIFT spectra of DUT-49 during irradiation at 365 nm under inert N_2 atmosphere. c,d) Direct comparison of DRIFT spectra of DUT-49 under during irradiation with 365 nm. Full spectrum (a,c) and magnified region of small wavenumber (b,d).

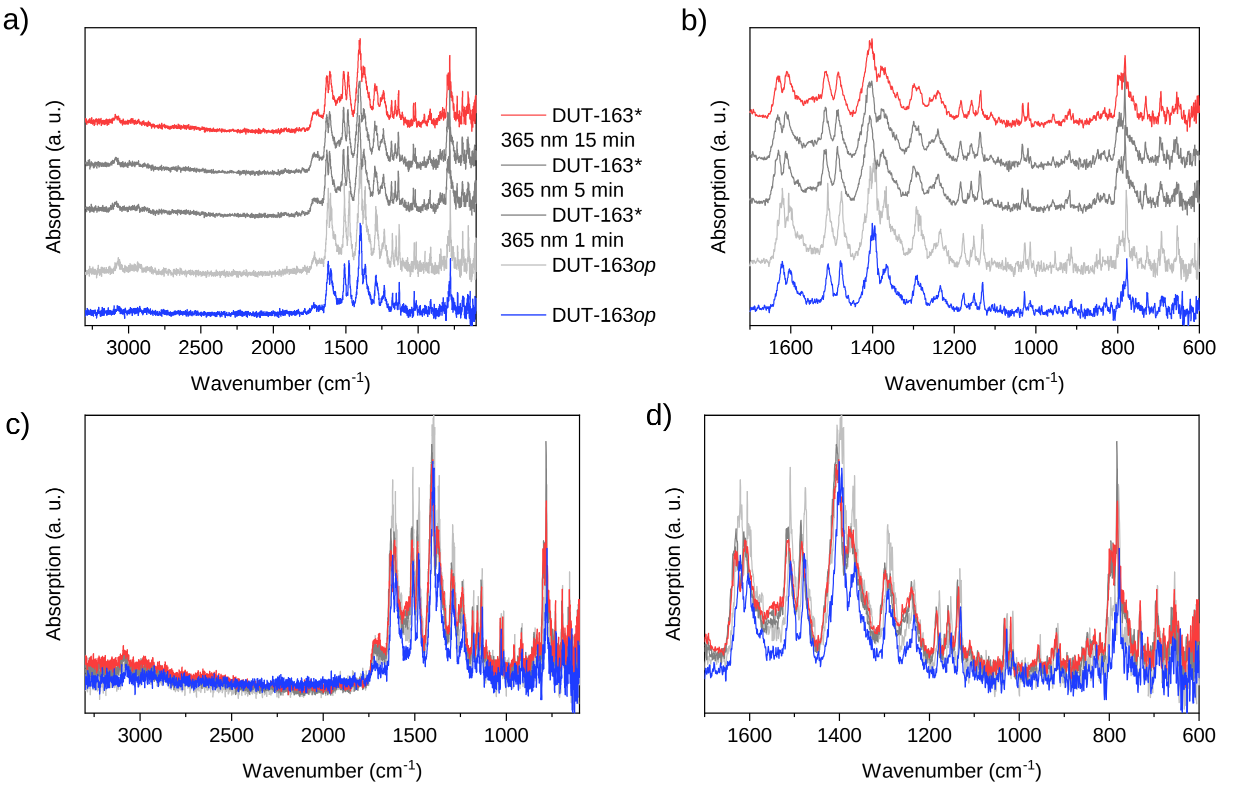

Supplementary Figure 90. a,b) DRIFT spectra of DUT-163 during irradiation at 365 nm under inert N_2 atmosphere. c,d) Direct comparison of DRIFT spectra of DUT-163 under during irradiation with 365 nm. Full spectrum (a,c) and magnified region of small wavenumber (b,d).

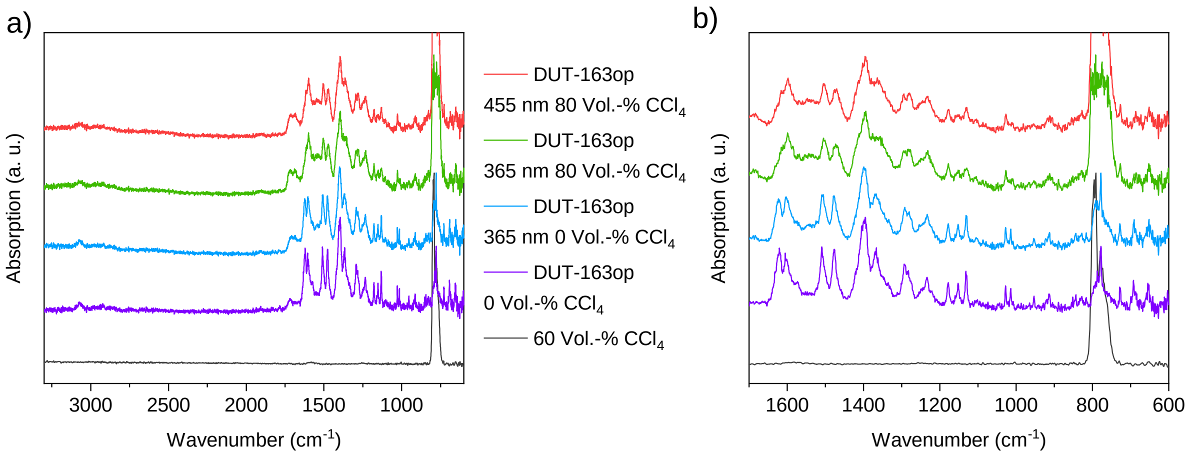

Supplementary Figure 91. a,b) DRIFT spectra of DUT-163 during irradiation at 365 nm and 455 nm and during CCl_4 loading. Full spectrum (a) and magnified region of small wavenumber (b). Comparison to blank 60 vol.-% CCl_4 as reference in black.

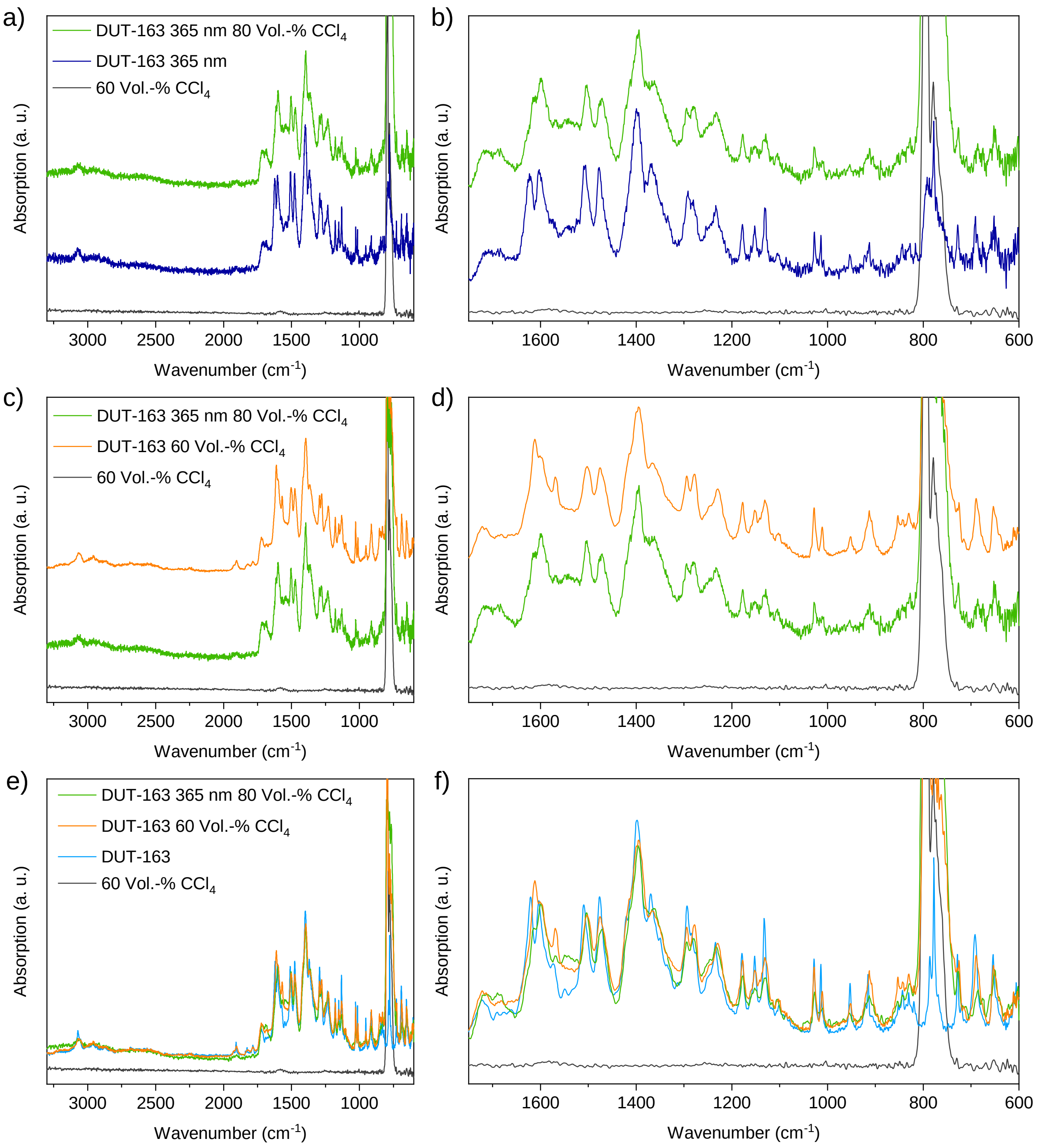

Supplementary Figure 92. a,b) DRIFT spectra of DUT-163 during irradiation at 365 nm under inert N_2 atmosphere and during irradiation at 365 nm and during CCl_4 loading. c,d) DRIFT spectra of DUT-163 during CCl_4 loading and during irradiation at 365 nm and during CCl_4 loading. Direct comparison of DRIFT spectra of DUT-163 under different conditions. Full spectrum (a,c,e) and magnified region of small wavenumber (b,d,f). Comparison to blank 60 vol.-% CCl_4 as reference in black.

16) In situ Raman spectroscopy

Raman experiments were conducted on 2 different Raman microscopes using a Raman microscope and 785 nm and 633 nm lasers for excitation of solid samples. For investigation of solid MOF Powders low sample amounts (<0.2 mg) were placed in a quartz cuvette under dry nitrogen atmosphere in a glovebox and sealed. Samples were irradiated with the same LED setup (365 and 455 nm) previously used for solution and solid-state UV/Vis and DRIFTS experiments.

Structural contraction without irradiation was investigated by CCl_4 adsorption in accordance to the DRIFTs experiments. In addition, Raman spectra were recorded of the samples previously analyzed by *in situ* PXRD which were sealed in an Ar-filled glovebox. To investigate effects of local irradiation on the structural transition capillaries from the PXRD experiments (CAP4, CAP10, CAP13 and CAP17) were used.

Supplementary Table 7. Summary of *in situ* DRIFTS experiments.

Experiment ID	Material	Figure	*T* (K)	Excitation wavelength (nm)	guest
Raman1	DUT-163	Supplementary Figure 84	295	365	CCl_4 0-60
Raman2	DUT-163	Supplementary Figure 79	295	365	0
Raman3	DUT-163	Supplementary Figure 81	295	365	MP
Raman4	DUT-163	Supplementary Figure 82	295	365	MP

* samples were analyzed in a constant flow of vapor/N_2 mixture with ca. 50 ml min^{-1}, Vol.-% given as CCl_4 concentration in nitrogen.

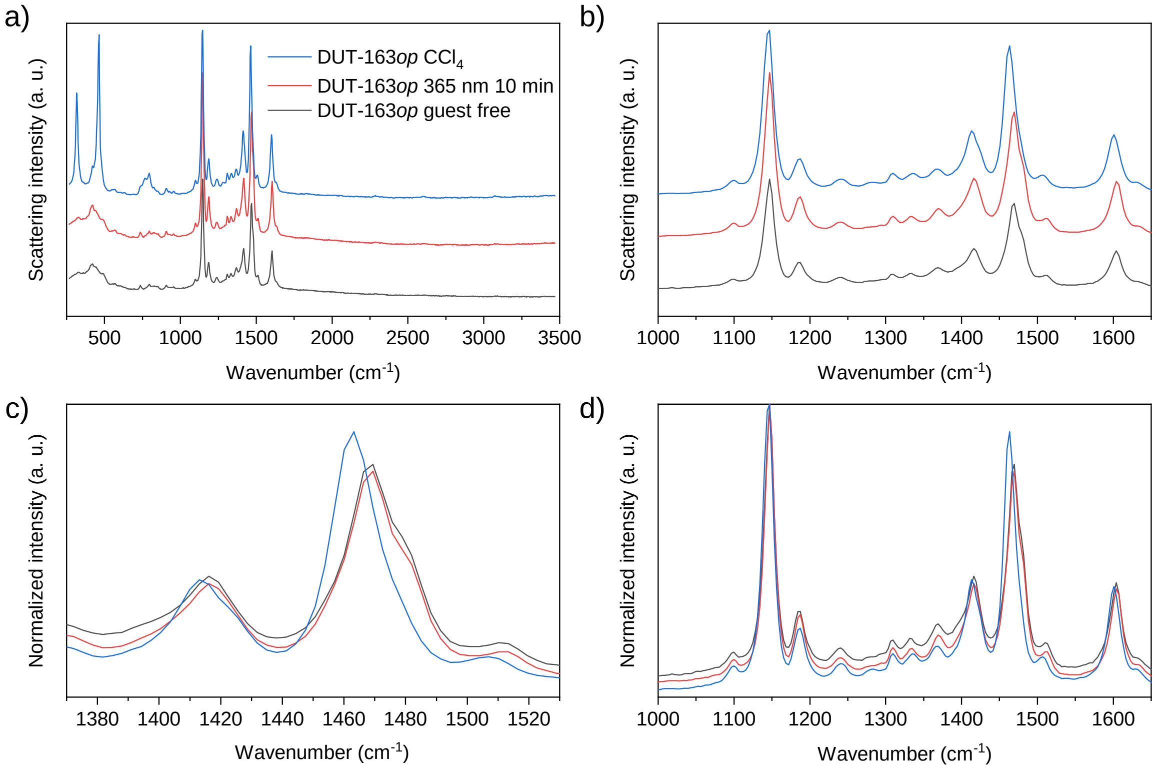

Supplementary Figure 93.a-d) *In situ* Raman (633 nm laser for excitation) spectra of DUT-163 recorded after irradiation with 365 nm and loading with CCl_4. a) Full range, b) selected region of modes relevant to the ligand in DUT-163, c,d) normalized intensity.

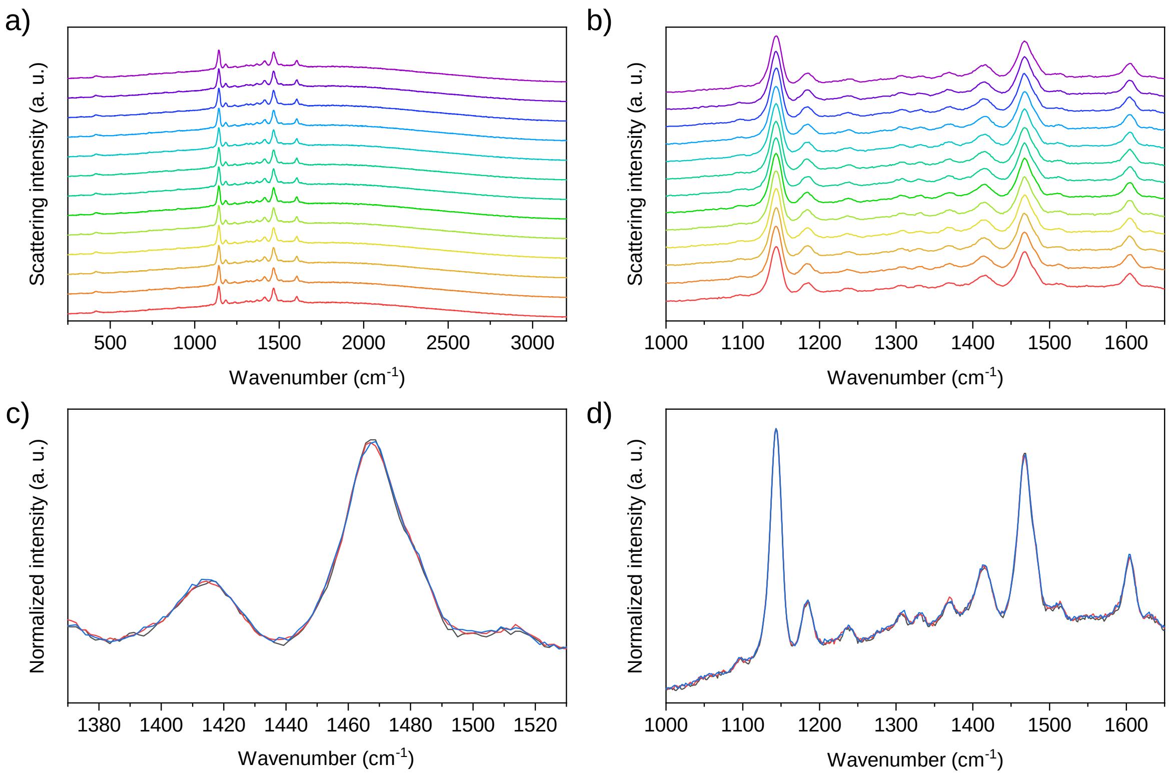

Supplementary Figure 94. a-d) *In situ* Raman (785 nm laser for excitation) spectra of DUT-163 recorded during irradiation with 365 nm. a) Full range, b) selected region of modes relevant to the ligand in DUT-163, c,d) normalized intensity.

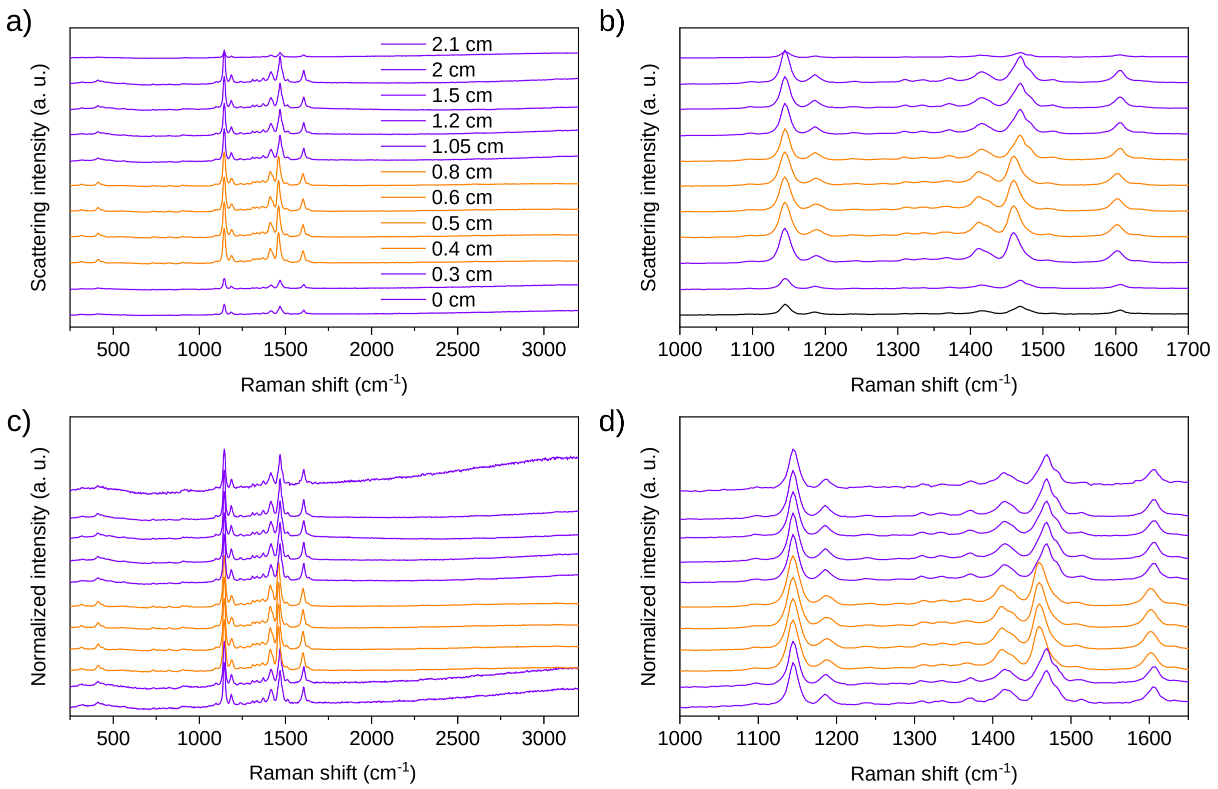

Supplementary Figure 95. a-d) *In situ* Raman (633 nm laser for excitation) spectra of DUT-163 in capillary retained from in situ PXRD experiment CAP5-CAP10. a) Full range, b) selected region of modes relevant to the ligand in DUT-163, c,d) normalized intensity

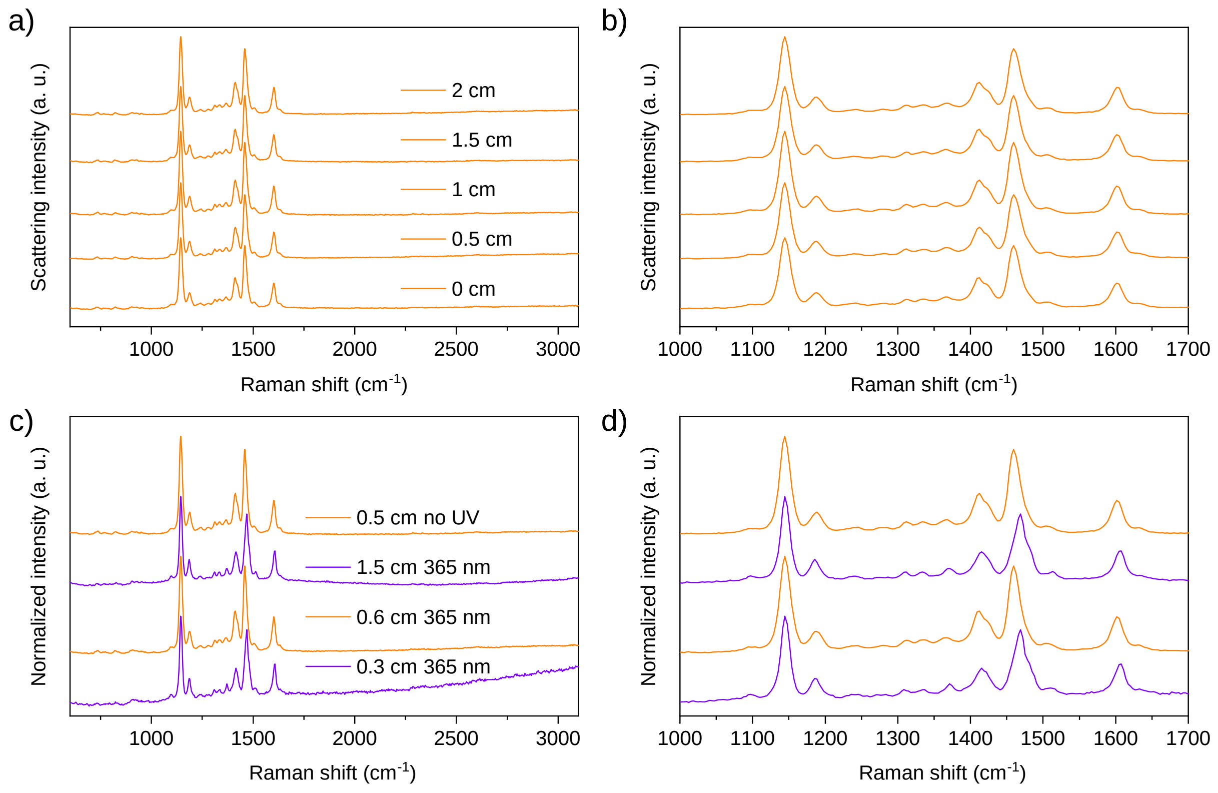

Supplementary Figure 96. a-b) *In situ* Raman (633 nm laser for excitation) spectra of DUT-163 in capillary retained from in situ PXRD experiment CAP1-CAP4 (MP loading at 296 K). a) Full range, b) selected region of modes relevant to the

ligand in DUT-163, c,d) Comparison to Raman spectra recorded for capillary of irradiation experiment displayed in Supplementary Figure 95 in normalized intensity.

17) Computational analysis of porosity and adsorption properties

Pore characteristics such as density, specific geometrical pore volume, and specific geometrical accessible surface area were calculated using Zeo++[21] in the default settings. Simulations were performed on the simulated crystal structures of DUT-163*op*, (*E*)-DUT-163*cp*, and (*Z*)-DUT-163*cp*.

Supplementary Table 8. Specific surface area, pore volume and density of the series of materials determined by Zeo++ based on simulated crystal structures of *op* and corresponding *cp* phases. Experimental pore volumes were determined from nitrogen adsorption isotherms at 77 K and a relative pressure of 0.95.

Material	Unit cell Volume (Å^3)	Specific geometric surface area simulated (m^2 g^{-1})	Specific pore volume simulated (cm^3 g^{-1})	Specific pore volume experimental (cm^3 g^{-1})	Density (g cm^{-3})
(*E*)-DUT-163*op sim.*	121496	5250	3.24		0.27
(*E*)-DUT-163*cp sim.*	53357	3589	1.12		0.61
(*Z*)-DUT-163*cp sim.*	69485	3887	1.61		0.47
(*E*)-DUT-163*op exp*	119386	5112	3.20		0.27
(*Z*)-DUT-163*cp exp.*	52982	3709	1.12		0.61

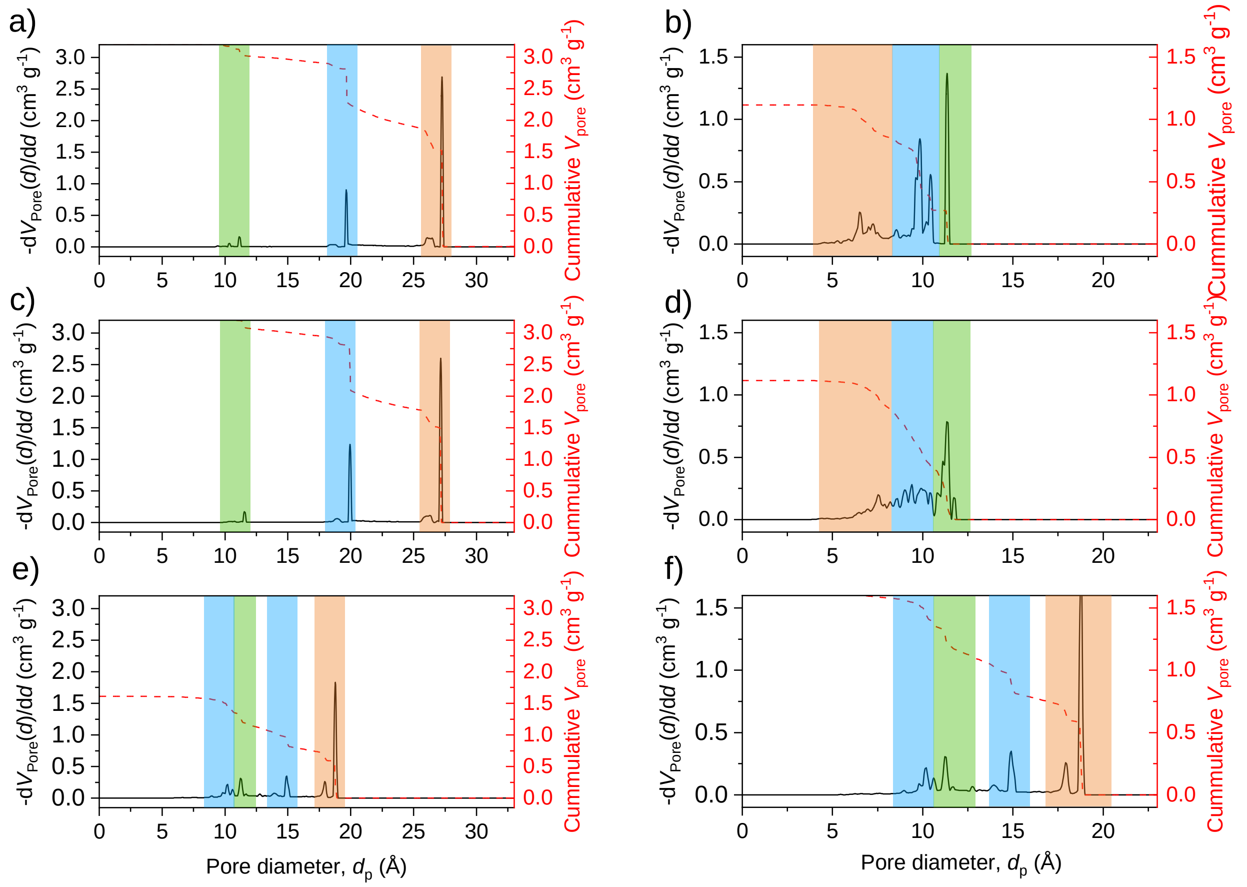

Supplementary Figure 97. Pore size distribution (PSD, black lines) and cumulative pore volume (V_p, red dashed lines) calculated by Zeo++ based on a) the experimental crystal structure of (*E*)-DUT-163*op*, b) the experimental crystal structure of (*E*)-DUT-163*cp*, c) the simulated crystal structure of (*E*)-DUT-163*op*, d) the simulated crystal structure of (*E*)-DUT-163*cp*, e,f) the simulated crystal structure of (*Z*)-DUT-163*cp*,

Grand canonical Monte Carlo (GCMC) simulations used the RASPA2.0 code[22] and simulated the adsorption for the op and cp structures of DUT-163. Each isotherm was simulated with an equilibration of 5×10^5 cycles and the subsequent 1×10^6 cycles were sampled, for each pressure point. A total of 50 pressures were investigated for each temperature and were uniformly distributed in log space between 1×10^{-2} Pa and 1×10^6 Pa. For each simulation, the van der Waals interactions for the framework used the UFF force field[23] and MP the united-atom TraPPe force field[24].

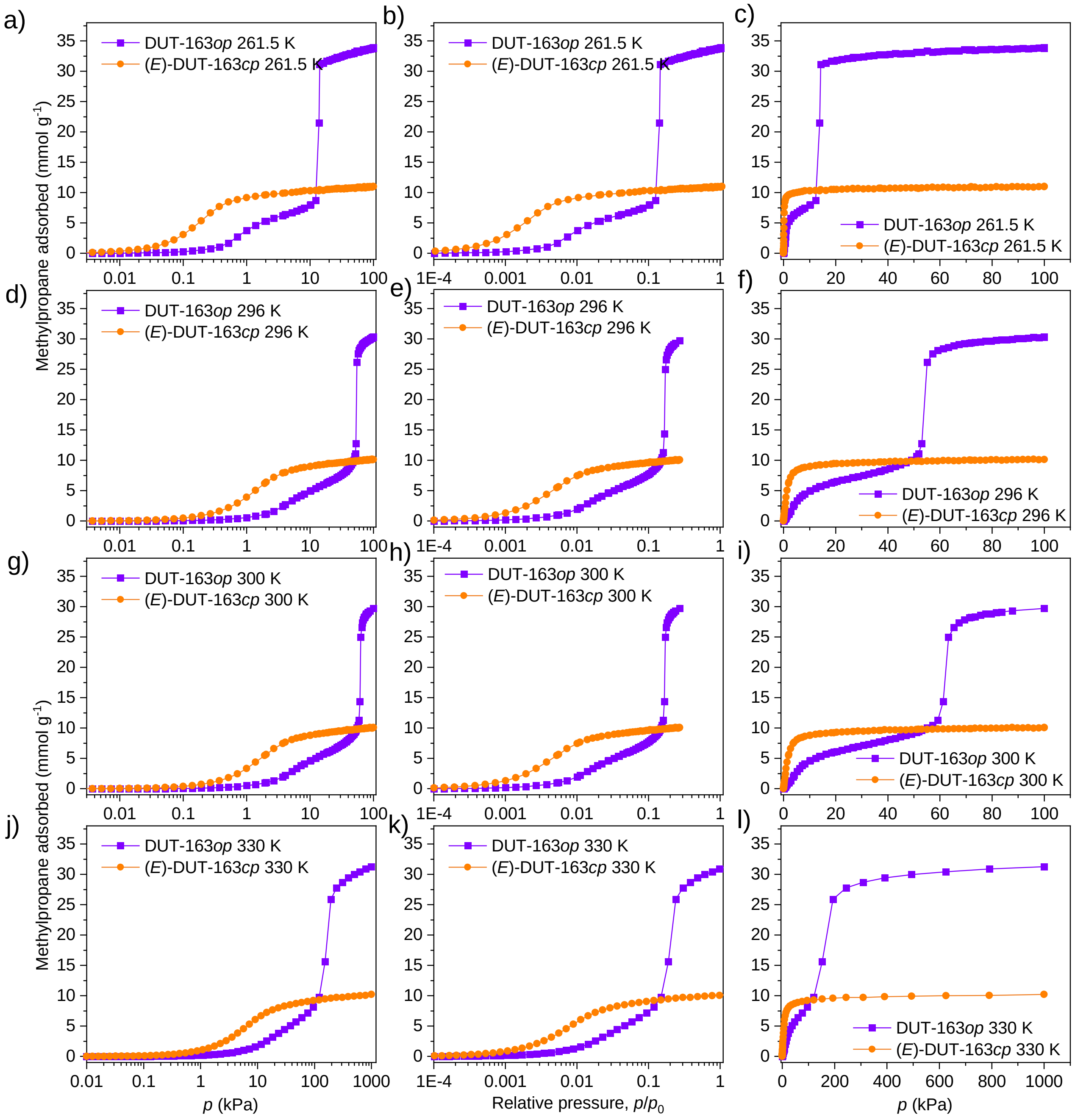

Supplementary Figure 98. Simulated MP adsorption isotherms based on the experimental crystal structures of DUT-163*op* (purple squares) and (*E*)-DUT-163*cp* (orange circles) at a-c) 261.5 K, d-f) 296 K, g-i) 300 K, and j-l) 330 K. Isotherms are shown in semilogarithmic absolute pressure (a,d,g,j), in semilogarithmic relative pressure (b,e,h,k), and absolute pressure (c,f,I,l).

To consider the propensity for adsorption-induced transitions the osmotic ensemble can be computed[25], $\Delta\Omega_{OS}(T,p)$, as a function of adsorption temperature, *T*, and pressure, *p*, the free energy difference between

the open and contracted state, $F_{op\text{-}cp}$, and change in volume, $V_{op\text{-}cp}$, the adsorbed amount, n_{ads}, as well as the molar fluid volume V_m, as described in supplementary equation (1).

$$\Delta\Omega_{OS}(T,p) = \Delta F_{op-cp} + p\Delta V_{op-cp} - \int_0^P n_{cp}(T,p)V_m(T,p)dp \quad (1)$$

All these values except ΔF_{op-cp} can be found in the above simulated isotherms so to consider the energetic contributions to the osmotic potential Δf_i is computed as described in supplementary equation 2.

$$\Delta f_i = \Delta\Omega_{OS}(T,p) - \Delta F_{op-cp} \quad (2)$$

This difference in osmotic potential function (Δf_i) allows us to characterize the energetic conditions for temperature dependent adsorption-induced contractions[12] as contraction will only occur when $\Delta f_i > \Delta F_{op-cp}$. The potential for contraction is observed to decrease with increasing adsorption temperature, as ΔF_{op-cp} is considered temperature independent[26].

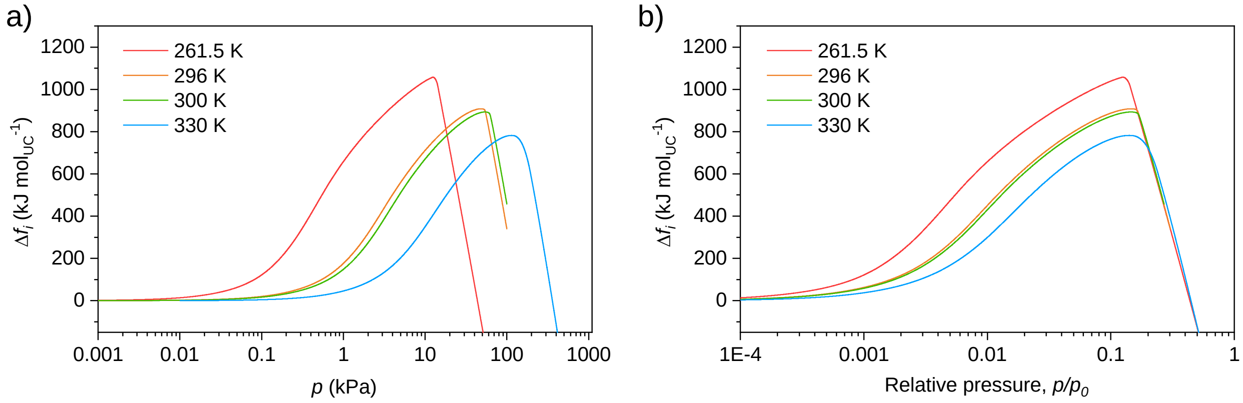

Supplementary Figure 99. Δf_i computed for four temperatures using GCMC simulations of the MP adsorption isotherms. Displayed as a function of pressure (a) and relative pressure (b).

18) Computational analysis of framework mechanics

Molecular dynamics simulations were used to compute the structures of DUT-163 using the MOF-FF forcefield[27]. Parameters missing from MOF-FF were adapted from the MM-3 forcefield[28] and previously reported parameters for azobenzene[29]. The lammps code was employed for computing the molecular dynamics and optimizations[30]. Op and cp structures were simulated starting from an idealized crystal structure. The conjugate gradient method was used for geometry optimization and steepest descent to relax the lattice parameter to a threshold root-mean-squared value of 0.0001Kcal/mole-Angstrom.

Free energy profiles were computed using a prior established protocol with the approximation of a cubic cell[31]. Bulk moduli were computed from the slope of the pressure-volume equations of state. The free energy profiles for MP included structures were computed using an identical approach employing the lammps "deposit" fix to randomly place gas molecules into the cell. MP molecules were treated as rigid particles using the same van der Waals forcefield parameters as the GCMC simulations.

E-Z isomerization is based on an excited transition state and to probe the influence of this on the framework properties simulations were completed using a modified classical potential. The DUT-163 classical potential parameters were modified by halving the dihedral parameters of C-N-N-C and turning off the improper dihedral potential imposed on the two carbon atoms of the azobenzene unit. This is one of the criteria reported by Böckmann et al. to emulate the NN-twist mechanism observed in nonadiabatic ab initio MD [10.1063/1.4818489]. Unfortunately, computation of the entire inversion transition state could so far not be

conducted. According to the DFT analysis of Me₄dacdc similar activation barriers are to be expected for both transitions.

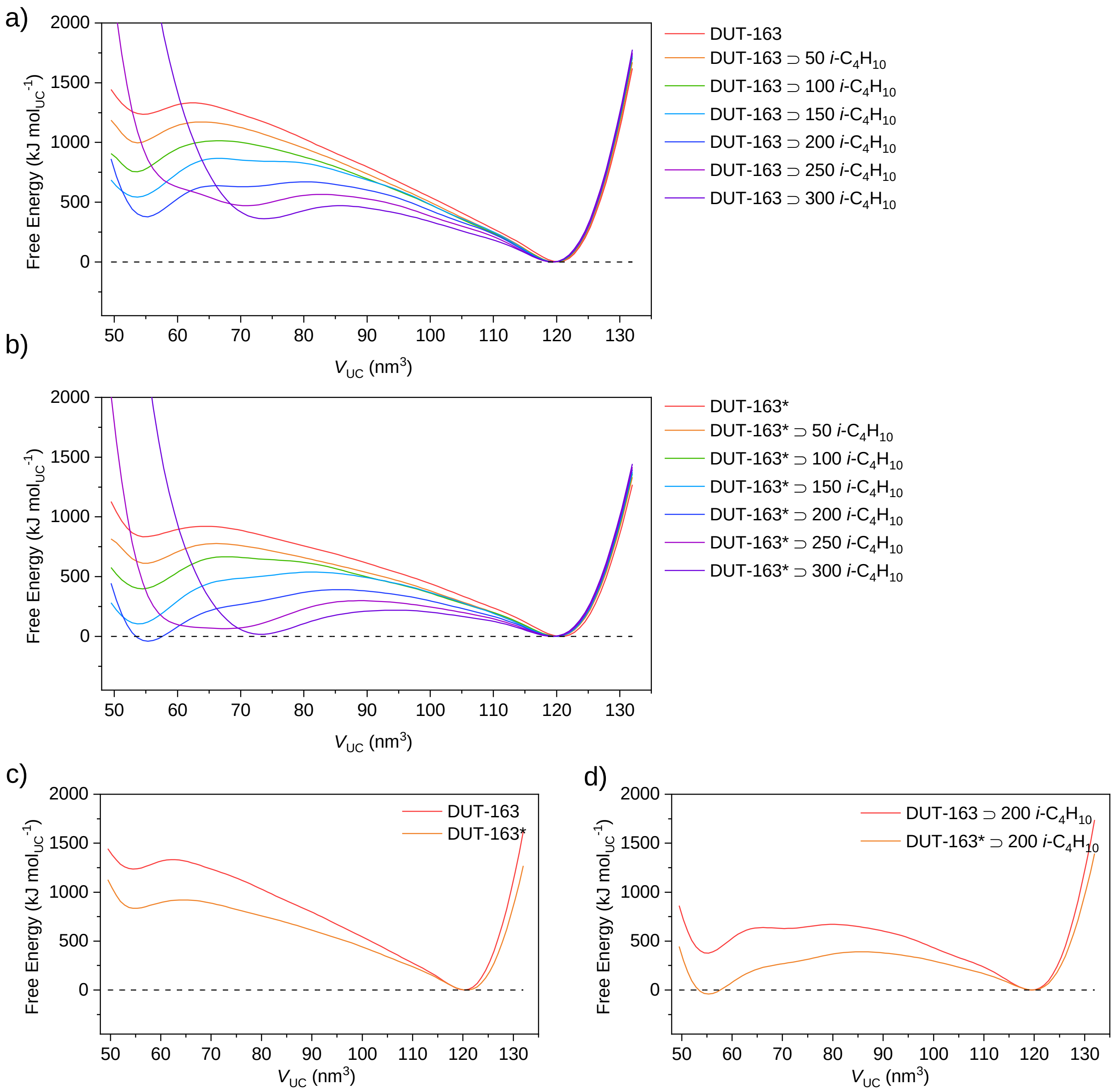

Supplementary Figure 100. Free energy landscape of a) DUT-163 and b) DUT-163* at dfferent loadings of MP (i-C_4H_{10}). c) comparison of guest free DUT-163 and DUT-163* and d) with a loading of 200 molecules MP per unit cell.

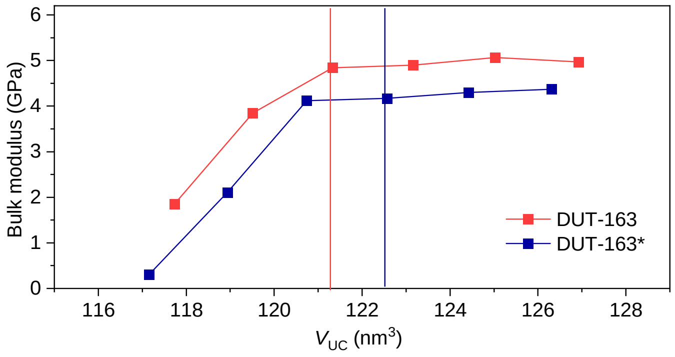

Supplementary Figure 101. Bulk modulus of DUT-163 and DUT-163*, the same structure but using the excited state modifications to the potential parameters. The bulk modulus reported here was determined from the slope of the pressure-volume equations of state used for the free energy calculations.

19) Computational analysis of single ligand ligand buckling

Ligand buckling simulations were performed according to a strategy previously applied to ligands in DUT-49-type frameworks.[14,26] The ligand dacdc was simulated using density functional theory (DFT) simulations employed by the ORCA 4.2.1 software.[32,33] The ωB97X-D/def2-TZVP//B3LYP/6-31G(d,p) level of theory consistent with the analysis of the E-Z isomerization described below. Dispersion corrections were included using the Grimme "D3-BJ" approach.[34,35] The optimized structure was strained by decreasing the N–N length from the identified local minimum of 11.84 Å to 11.04 Å, in 20 steps. No other constraints to the structure were applied. For each step, the structure was optimized with default convergence criteria and with this N–N length fixed.

20) Computational analysis of the thermal *E-Z* isomerization of Me_4dacdc

All the calculations for the thermal isomerization of Me_4dacdc were carried out using the Gaussian 16 Rev. B.01[36] at the ωB97X-D/def2-TZVP//B3LYP/6-31G(d,p) level of theory. To take into account the diradical nature of the rotational transition state, a broken-symmetry approach was included. All the optimizations were confirmed to be stationary points by the number of imaginary frequencies found (0 for the minima,1 for the transition state). The cartesian coordinates (in Å) and energies (in Hartrees) for all the relevant structures are reported in the following section.

Supplementary Table 9. Cartesian coordinates in Å for (*Z)*-Me_4dacdc.

N	-4.532126098	2.6144150529	4.472869138
N	-4.942977692	1.4857104404	4.8174997195
C	-4.7441254436	0.2885186185	4.0542895036
C	-5.8173717287	-0.6120030929	4.0176791009
C	-5.6727827071	-1.8551438106	3.4119112759
C	-4.4354450734	-2.2311588877	2.8716689487
C	-3.3488330399	-1.3494758838	2.9475677385
C	-3.4992064075	-0.0976451357	3.5351229498
N	-4.2783895145	-3.5066062087	2.2665787609
C	-3.3300677709	-4.4707574326	2.6240769117
C	-3.4915080252	-5.6064630315	1.7891055744
C	-4.5870241881	-5.3108072838	0.886156775
C	-5.0476731258	-4.0090890865	1.2124673543
C	-5.1928329249	-6.0156718109	-0.1513432533
C	-6.2443928238	-5.4222225374	-0.8571198343
C	-6.6803270295	-4.1216508963	-0.5263313034
C	-6.0894697141	-3.4014191724	0.5048716448
C	-2.369157807	-4.4376658047	3.6394795748
C	-1.5542590658	-5.5520902995	3.7973487268
C	-1.6902643908	-6.6851462683	2.967502198

C	-2.6642810139	-6.7135140086	1.9643716191
C	-6.8621453198	-6.2094499463	-1.956687211
C	-0.8270345605	-7.8860835727	3.1200675395
C	-3.997784143	2.9114141274	3.177129076
C	-1.6877403447	4.2625226201	-2.6721332462
C	-2.1666448462	3.4492113243	-1.61278627
N	-2.4979604334	4.266508005	-0.5271006021
C	-2.2388967123	5.595922452	-0.8762849937
C	-1.7350898619	5.633670814	-2.2021808953
C	-1.2801369498	3.6698752158	-3.8652174
C	-1.3456363557	2.2787467497	-3.9931946327
C	-1.8115388809	1.485458736	-2.9234232389
C	-2.2236156792	2.0566214633	-1.7255256995
C	-2.4433779831	6.7642949299	-0.1356958176
C	-2.1160264417	7.9757250006	-0.7324774763
C	-1.5989979887	8.0323635933	-2.0441110555
C	-1.412660141	6.8588196138	-2.7816458904
C	-3.0053072531	3.818183375	0.7210429255
C	-2.3598424165	4.1707479571	1.9139740896
C	-2.877397297	3.7520423169	3.1348598846
C	-4.6496322479	2.5657059269	1.9836469772
C	-4.1648123991	3.0316365108	0.7666640291
C	-1.2403405726	9.3187162826	-2.6975474823
C	-0.9016407317	1.6855109995	-5.2823204049
O	-0.4927285568	2.3249056172	-6.2327277067
O	-0.7956254593	9.4210596065	-3.8249540579
O	-6.5142858517	-7.3277186272	-2.2850706975
O	-0.9099391755	-8.8878351486	2.4352599677
O	0.0800584619	-7.7512036085	4.1182200141
O	-7.8712692524	-5.5439616442	-2.5700659963
O	-1.4630476738	10.3887596306	-1.8955475514
O	-0.9990923008	0.3337616802	-5.2925215315
C	-0.5823624973	-0.2983544348	-6.5121695274
C	-1.136535106	11.6612841649	-2.473876521
C	-8.5044110409	-6.2552386286	-3.6442777637
C	0.9412796079	-8.8827402384	4.3146705896
H	-6.7573286714	-0.3251614701	4.4780045019
H	-6.5032965891	-2.5523431635	3.3844558439
H	-2.3906796402	-1.6454480218	2.5337504436
H	-2.6525488417	0.5768209136	3.593237897
H	-4.8699812999	-7.0125197468	-0.4317865915
H	-7.4906494576	-3.6799251241	-1.0937003812
H	-6.4263741671	-2.3996759624	0.7460492034
H	-2.2638083582	-3.5769733562	4.2899553525
H	-0.799186585	-5.5586484155	4.5741488994
H	-2.7555386852	-7.6009515506	1.3473299728
H	-0.9089866775	4.2566058095	-4.6986762237
H	-1.8434965486	0.409395687	-3.0444417713
H	-2.5717787771	1.4372723334	-0.9068552921
H	-2.8502167831	6.7330507744	0.8685519091
H	-2.2613600907	8.8998037349	-0.1861359397
H	-1.0236898896	6.9300815976	-3.791736387
H	-1.4620064073	4.7782668936	1.8784233688
H	-2.4093263741	4.0500099122	4.067406505
H	-5.5488460834	1.960790856	2.0123814117
H	-4.6865176381	2.7968542827	-0.1549729951
H	-0.7221051521	-1.3672903499	-6.3520283725
H	-1.1897014663	0.0467142614	-7.3525417632
H	0.4664522996	-0.0755986886	-6.723213402

H	-1.3740242752	12.4013209342	-1.709763662
H	-0.0763141528	11.7058460412	-2.734949524
H	-1.7271270254	11.8379058941	-3.376174587
H	-9.2822594546	-5.5902681933	-4.0193053239
H	-7.7831690061	-6.4852296933	-4.4323912753
H	-8.9395136119	-7.190603338	-3.2839551134
H	1.598780708	-8.6121036601	5.1406913962
H	0.3579858196	-9.7725822164	4.5638571469
H	1.5223738711	-9.0863512988	3.4119222058

Energy (B3LYP/6-31G(d,p))= -2516.8540202
Energy (ωB97X-D/def2-TZVP)= -2516.8835301
Zero-point correction= 0.675133 (Hartree/Particle)
Thermal correction to Gibbs Free Energy= 0.584766
Sum of electronic and zero-point Energies= -2516.178887
Sum of electronic and thermal Energies= -2516.130718
Sum of electronic and thermal Enthalpies= -2516.129774
Sum of electronic and thermal Free Energies= -2516.269254
Imaginary Frequencies 0

Supplementary Table 10. Cartesian coordinates in Å for (*E)*-Me_4dacdc

C	3.6052307431	-6.2624851819	-3.3355583982
C	3.3540322939	-4.9587337769	-2.8357652692
N	2.0790715391	-4.9310708404	-2.2593644477
C	1.5079793784	-6.2025937808	-2.3842610595
C	2.425949444	-7.0561010572	-3.0489853119
C	4.825299425	-6.5436086589	-3.946168656
C	5.7851090573	-5.531788454	-4.0502200794
C	5.5236543767	-4.2456368757	-3.5324086901
C	4.3138034376	-3.9449949165	-2.918563064
C	0.2405533869	-6.6496026756	-1.997577382
C	-0.0903062728	-7.9724984766	-2.2646910486
C	0.8158663859	-8.8406620043	-2.9095561146
C	2.074903079	-8.3792287695	-3.3066812768
C	1.470078268	-3.8031533256	-1.6507321009
C	0.957391791	-3.8946309876	-0.3506082177
C	0.3543157125	-2.7853409636	0.2326504469
C	0.2723823327	-1.5704924137	-0.4607670903
C	0.7920131452	-1.4804825586	-1.7644473813
C	1.3779135675	-2.5897926303	-2.3550005844
N	-0.351974591	-0.5094097071	0.236137418
N	-0.3720641422	0.5898769309	-0.3841769794
C	-1.0050528624	1.6485073045	0.3086585842
C	-0.9895830088	2.8910370356	-0.3388586113
C	-1.5727764882	4.0059784762	0.2537683989
C	-2.1990703588	3.8822152665	1.5003542821
C	-2.2285136064	2.6342944322	2.1473158554
C	-1.6308438716	1.5283284519	1.562310438
N	-2.8055800978	5.0108392898	2.1101557055
C	-3.7562535211	5.8498759826	1.5179111717
C	-4.1182928442	6.8613789361	2.4446211479
C	-3.3483461046	6.6217342466	3.6497796871
C	-2.5524066067	5.4727983526	3.4066991955
C	-3.2598707994	7.2747463147	4.8770491108
C	-2.3801259285	6.7857274253	5.8480026857
C	-1.5843170569	5.6514398913	5.5820081095
C	-1.6564689205	4.9864523803	4.3640109676

C	-4.3497281607	5.7680164982	0.2542669283
C	-5.2971502398	6.7276242654	-0.0810172516
C	-5.6575298966	7.7509022477	0.8210561435
C	-5.069804763	7.8143192794	2.0884786668
C	0.4806277857	-10.2593630613	-3.2011676383
C	7.0723336766	-5.8714240714	-4.7119217813
C	-2.3182058875	7.5087893026	7.1454987967
C	-6.6668149146	8.7874892458	0.4790531335
O	7.3504010525	-6.9665641585	-5.1621266544
O	1.2241775106	-11.0452422901	-3.7569187761
O	-7.0138420109	9.6860631099	1.2216626422
O	-2.97285685	8.496917742	7.4182111082
O	-7.1730177227	8.6285771318	-0.7685143807
O	-1.4422337125	6.9452868983	8.0132819916
O	-0.762087343	-10.5974918086	-2.7777917973
O	7.9229754585	-4.817200415	-4.7660598439
C	9.18487089	-5.0852428781	-5.395357073
C	-1.1434234398	-11.9571170789	-3.0353171261
C	-1.339155318	7.6012724988	9.2857990331
C	-8.1565315434	9.600314658	-1.1541879891
H	5.0548385615	-7.5274393385	-4.3411782175
H	6.2877719098	-3.4821877077	-3.6149173502
H	4.1290870319	-2.9570557723	-2.513005347
H	-0.4679207908	-5.9887932591	-1.5117024871
H	-1.0639089172	-8.3506888422	-1.9772574108
H	2.7490484813	-9.0627359247	-3.8116594828
H	1.047985006	-4.8254605663	0.1983144936
H	-0.047770096	-2.8318862729	1.2392783086
H	0.7106418338	-0.5401689177	-2.2967278193
H	1.7547796558	-2.5357579805	-3.3708695298
H	-0.496826104	2.9633498237	-1.3027572617
H	-1.5332935977	4.9737749907	-0.2338564189
H	-2.7381829123	2.5425183501	3.1006006383
H	-1.6492149221	0.559668735	2.0475879694
H	-3.8520245807	8.1555216748	5.1011746047
H	-0.9020583833	5.2990510411	6.3461305558
H	-1.0309080632	4.1238012201	4.1662040092
H	-4.0890416925	4.9799457481	-0.4426306062
H	-5.7751778285	6.6932608878	-1.0525402477
H	-5.3725825918	8.6033691844	2.7684660762
H	9.7351191803	-4.1449527508	-5.3636387445
H	9.7272541653	-5.865547343	-4.8556934017
H	9.039257254	-5.4105428584	-6.4283257114
H	-2.1528669635	-12.0599812186	-2.6373661389
H	-1.1307094633	-12.1645664512	-4.1081599702
H	-0.4615775486	-12.6502983926	-2.5365712833
H	-0.6115258984	7.0241682659	9.8562645402
H	-0.9989674127	8.6324100882	9.1620290185
H	-2.306248591	7.6103348465	9.7944207715
H	-8.4536655918	9.3318510265	-2.1678747674
H	-9.0163544168	9.5670184021	-0.4805525912
H	-7.7332895672	10.6075595327	-1.1321527723

Energy (B3LYP/6-31G(d,p))= -2516.8795007
Energy (ωB97X-D/def2-TZVP)= -2516.9032754
Zero-point correction= 0.675734 (Hartree/Particle)
Thermal correction to Gibbs Free Energy= 0.585102
Sum of electronic and zero-point Energies= -2516.203766
Sum of electronic and thermal Energies= -2516.155545

Sum of electronic and thermal Enthalpies= -2516.154601
Sum of electronic and thermal Free Energies= -2516.294399
Imaginary Frequencies 0

Supplementary Table 11. Cartesian coordinates in Å for transition state *inversion*-Me_4dacdc

N	-0.4857451254	-3.7371779722	-1.0239071536
N	0.2394259787	-3.6787328912	-2.0110190534
C	1.4825843442	-2.9523501109	-1.8786451613
C	2.2759253156	-2.9291406361	-3.028276722
C	3.4986981959	-2.2662951303	-3.0182501478
C	3.9234888137	-1.6082014179	-1.8555121441
C	3.1160850101	-1.6263671799	-0.7032942855
C	1.904157832	-2.3014452348	-0.7118720734
N	5.1610827809	-0.9218229588	-1.8386095002
C	6.1727639377	-1.0806952032	-0.8811881627
C	7.251772982	-0.2207640462	-1.2077248078
C	6.8738785768	0.4911130701	-2.4134248169
C	5.5818620402	0.0327217946	-2.7753558433
C	7.502313525	1.464698118	-3.1865347021
C	6.840343938	1.9786250596	-4.3058117658
C	5.5477510327	1.524138854	-4.639829927
C	4.9036878517	0.5533707411	-3.8815831766
C	6.2322823729	-1.9460734698	0.2153579257
C	7.3789551406	-1.9207517825	1.0006776316
C	8.4541537347	-1.0583810228	0.7021556543
C	8.3924298076	-0.2107410811	-0.4081791745
C	7.5470735931	3.014306577	-5.1065206658
C	9.6883392992	-1.0149312181	1.5318235302
C	-1.2727618107	-3.8081322352	0.046376274
C	-4.7751637004	-3.6376523967	5.4790464584
C	-3.7666661577	-3.2413562247	4.5603971757
N	-3.8207601722	-4.064754291	3.4385550972
C	-4.8481695365	-4.9884553347	3.6168732181
C	-5.4676537483	-4.7597466112	4.874493822
C	-4.9109411674	-2.9537203688	6.6849405916
C	-4.0476084168	-1.8890916068	6.9674678044
C	-3.0486253947	-1.5141089859	6.042122306
C	-2.8958805274	-2.1821244639	4.83409641
C	-5.2800226488	-6.0018770548	2.7557656296
C	-6.341851923	-6.794776481	3.1714353345
C	-6.9687051155	-6.58766445	4.4201055613
C	-6.5311007918	-5.5671113685	5.2717084335
C	-2.9652009701	-3.9785767329	2.2989846857
C	-1.9712297419	-4.9403819815	2.0799627535
C	-1.1349616935	-4.8769844956	0.9762045895
C	-2.2800868572	-2.8279562981	0.2708843441
C	-3.106262273	-2.9321455924	1.3791656352
C	-8.104261768	-7.4256104954	4.8818290454
C	-4.2266887804	-1.1865971826	8.2631918072
O	-5.067774051	-1.4685436647	9.0960622057
O	-8.6807967132	-7.2896701716	5.9447672161
O	8.6568283679	3.4423846838	-4.8539336897
O	10.6427864796	-0.29491283	1.3099588528
O	9.6406125245	-1.8737465215	2.578223199
O	6.8164273006	3.4401002217	-6.1647965454
O	-8.4465486081	-8.3823390406	3.981628271

O	-3.3391977555	-0.1734382807	8.4345747009
C	-3.4659401179	0.5411960531	9.6716706951
C	-9.5404905392	-9.2223340727	4.3750877241
C	7.4466209724	4.443468239	-6.9761888535
C	10.8064129476	-1.878096349	3.4168372377
H	1.9234175842	-3.4503436478	-3.9124418247
H	4.134876187	-2.2684731744	-3.8961222909
H	3.4416901437	-1.0924875581	0.1827513448
H	1.2727022577	-2.3149911216	0.1706950834
H	8.4903398407	1.840075128	-2.9427158092
H	5.0503960593	1.946753317	-5.504416
H	3.9048720959	0.2231035271	-4.1419685006
H	5.4196551479	-2.6243767657	0.447988212
H	7.456022069	-2.577396961	1.8587828395
H	9.237989513	0.4332997672	-0.6245100191
H	-5.6670829647	-3.2228232851	7.4147648518
H	-2.391749028	-0.6883054303	6.287795645
H	-2.1271439002	-1.8938227647	4.1256111168
H	-4.8021049765	-6.1609105259	1.7953939236
H	-6.7034986929	-7.5894505109	2.529989591
H	-7.0329077494	-5.4275832382	6.2233024367
H	-1.8520079112	-5.7531569539	2.7900780657
H	-0.3739691619	-5.6308829438	0.812022162
H	-2.4000943688	-2.0159593248	-0.436693886
H	-3.8810298264	-2.1875917807	1.5354189056
H	-2.692022521	1.3086093045	9.649432116
H	-4.4556130083	0.9972632112	9.7555310253
H	-3.3172521388	-0.1285211514	10.522481606
H	-9.6833078281	-9.9244114539	3.5534927668
H	-9.3054077352	-9.7563936701	5.2992226116
H	-10.4450939935	-8.6301466524	4.534800225
H	6.735049403	4.6689441468	-7.7702351757
H	7.6599269187	5.3379910137	-6.3860128073
H	8.3833897399	4.06776122	-7.3948417898
H	10.6046557689	-2.611989052	4.1968049688
H	11.6924032436	-2.1607050464	2.8431478077
H	10.971032942	-0.8896355359	3.8524874195

Energy (B3LYP/6-31G(d,p))= -2516.8158442
Energy (ωB97X-D/def2-TZVP)= -2516.8411473
Zero-point correction= 0.673117 (Hartree/Particle)
Thermal correction to Gibbs Free Energy= 0.581193
Sum of electronic and zero-point Energies= -2516.142727
Sum of electronic and thermal Energies= -2516.094450
Sum of electronic and thermal Enthalpies= -2516.093506
Sum of electronic and thermal Free Energies= -2516.234651
Imaginary Frequencies 1

Supplementary Table 12. Cartesian coordinates in Å for transition state *rotation*-Me_4dacdc

N	1.0727942756	-5.9023503736	2.0360695192
N	0.0039766411	-5.7466587827	2.7704382348
C	-1.1528005188	-5.2232197049	2.2680049699
C	-2.2520228561	-5.1115143181	3.1601785424
C	-3.4611026937	-4.5965732027	2.7304560288
C	-3.6192024418	-4.1786312965	1.3957087331
C	-2.5419191788	-4.2954042533	0.4984219616
C	-1.3289921642	-4.8136848316	0.9174974381

N	-4.8584029672	-3.6501103872	0.9559328191
C	-5.5914258228	-4.0935115987	-0.1512777437
C	-6.7710660464	-3.3138665439	-0.2650330924
C	-6.747721352	-2.3559428703	0.8234885438
C	-5.5557468218	-2.5944564722	1.5548321362
C	-7.6074363408	-1.3344744514	1.2203802507
C	-7.2741697248	-0.5564218121	2.3338054478
C	-6.0762758507	-0.7976675626	3.0389432001
C	-5.2048519959	-1.81134722	2.6590129315
C	-5.320927639	-5.1456882259	-1.0318300057
C	-6.2365371971	-5.3931419696	-2.0475291486
C	-7.4061843563	-4.6167208148	-2.1872854616
C	-7.6762649698	-3.5783931482	-1.2901045311
C	-8.2200306878	0.5215361505	2.7261284962
C	-8.3959081528	-4.864536926	-3.2688555762
C	1.9660900949	-4.8907671327	1.8312899954
C	5.86816406	0.0556180595	0.4488513954
C	4.6367756739	-0.6359297331	0.5821350058
N	4.8899722536	-1.9279617813	1.0579747033
C	6.2716438902	-2.0725994156	1.2329241047
C	6.911729781	-0.8612010425	0.8654847542
C	5.8758552046	1.3647481232	-0.026669594
C	4.6641475548	1.9725485607	-0.3709547103
C	3.4509007101	1.2632629564	-0.2478516027
C	3.4216921714	-0.0435741616	0.2240479348
C	6.9952350272	-3.163731807	1.7242072456
C	8.375811435	-3.0364475185	1.8197993378
C	9.0315801234	-1.8458249611	1.4420402897
C	8.2965905446	-0.7539789452	0.9690969868
C	3.9131937688	-2.9208059309	1.316807469
C	4.0394468083	-4.2041631716	0.7524680716
C	3.0927225162	-5.1768518261	1.0153078557
C	1.8514678091	-3.592331497	2.4007958306
C	2.815217838	-2.6321640423	2.1483709421
C	10.5075483149	-1.6916726676	1.5346736876
C	4.715456646	3.3716524566	-0.8716620158
O	5.7350459303	4.0218671383	-1.0015809963
O	11.1176037713	-0.6851668122	1.2280012236
O	-9.2596571721	0.7728969695	2.1471416246
O	-9.4202771455	-4.2281892719	-3.426985389
O	-8.0357439919	-5.8908133138	-4.077706636
O	-7.8002034889	1.2105150881	3.8154299017
O	11.1180647422	-2.8069627997	2.0042816476
O	3.4881315005	3.8603455756	-1.1750352676
C	3.474284525	5.2092293942	-1.6660723587
C	12.5462668311	-2.7173031109	2.1175424143
C	-8.6742592528	2.2664903366	4.241596774
C	-8.9553542436	-6.1789510489	-5.1416380851
H	-2.1186810133	-5.4510739007	4.1816539057
H	-4.3022671723	-4.5316458117	3.4121115996
H	-2.6669977232	-3.9629211479	-0.5267602292
H	-0.5037913132	-4.9107042914	0.2213375935
H	-8.5280710172	-1.1212767316	0.6878370386
H	-5.8351976448	-0.1740053151	3.89128381
H	-4.2817522425	-1.9800610641	3.201323582
H	-4.4327054984	-5.7576951662	-0.9260897208
H	-6.0563556622	-6.2001310064	-2.7474095443
H	-8.5888220332	-3.0045787075	-1.4108379205
H	6.7963540401	1.9268260041	-0.1422909751

H	2.5268930069	1.7519983775	-0.5322729263
H	2.4848627037	-0.5828862505	0.3025281797
H	6.5013334844	-4.078669245	2.0300763058
H	8.966093588	-3.8636429359	2.1950797383
H	8.8227811465	0.1548441069	0.6974923659
H	4.878666541	-4.4189519536	0.0998309111
H	3.1765339409	-6.1702911685	0.5881136263
H	1.019071793	-3.3748725358	3.0603562062
H	2.7428819492	-1.652873794	2.6097885474
H	2.4268935066	5.441735344	-1.8578018156
H	3.890032824	5.8943177168	-0.923153373
H	4.0602151574	5.2912170706	-2.5848144446
H	12.8718823656	-3.6829496109	2.5040175646
H	12.9989348853	-2.5215128421	1.1424001926
H	12.8291825671	-1.914336257	2.8026207779
H	-8.1957592794	2.7101847391	5.1145520258
H	-8.7949099736	3.0103590793	3.4502393668
H	-9.6586013672	1.8704484188	4.5030064679
H	-8.5211841136	-7.0168529753	-5.6868710369
H	-9.9359840058	-6.4480153107	-4.7415174446
H	-9.0703308349	-5.31233034	-5.7972963926

Energy (B3LYP/6-31G(d,p))= -2516.8284333
Energy (ωB97X-D/def2-TZVP)= -2516.8431238
Zero-point correction= 0.672441 (Hartree/Particle)
Thermal correction to Gibbs Free Energy= 0.582975
Sum of electronic and zero-point Energies= -2516.155992
Sum of electronic and thermal Energies= -2516.108118
Sum of electronic and thermal Enthalpies= -2516.107173
Sum of electronic and thermal Free Energies= -2516.245458
Imaginary Frequencies 1

Based on these calculations Raman and Infrared absorption spectra could be simulated which are displayed in the following in comparison to experimental data obtained for *n*-Bu_4dacdc.

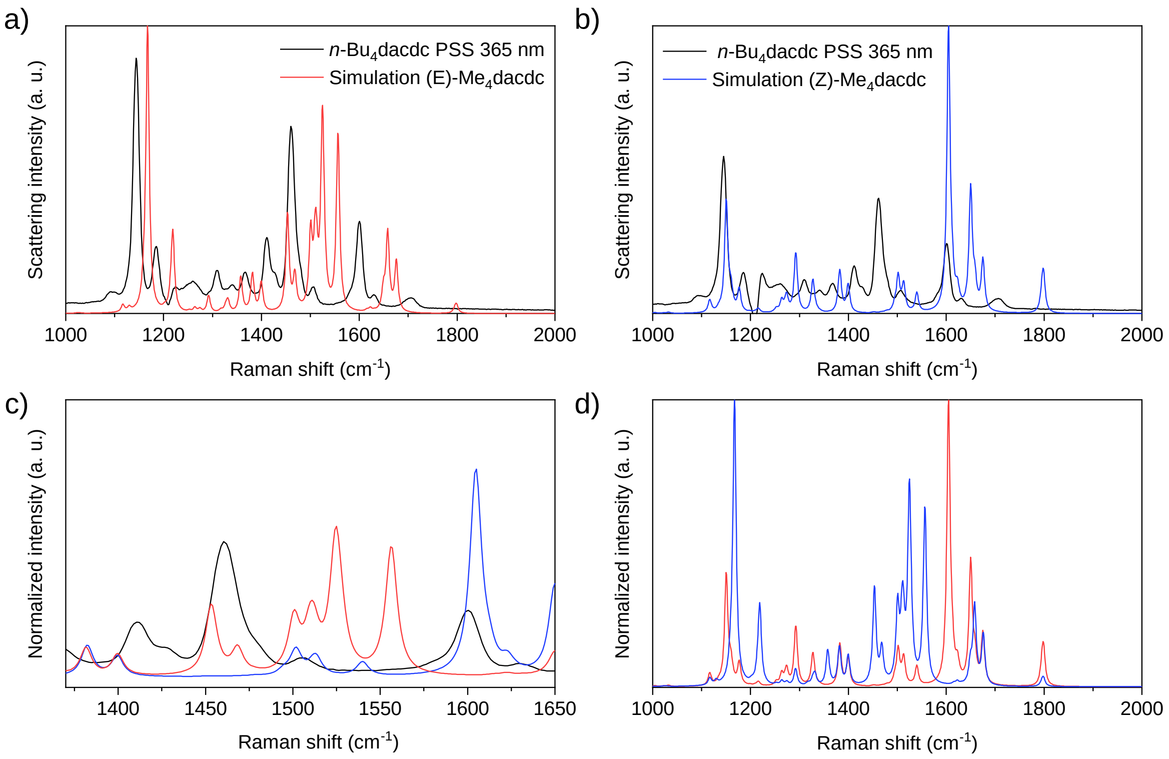

Supplementary Figure 102. a-c) Experimental Raman spectra at the photo stationary state (PSS) of *n*-Bu_4dacdc in $CHCl_3$ (black) in comparison to DFT simulated Raman spectra for the *E* (red) and *Z*-isomer (blue) of the corresponding methyl ester, d) comparison of simulated spectra.

In the simulated Raman spectra two particularly interesting bands distinguish the *E* from the Z-isomer which are observed at 1453 and 1468 cm-1. Both of these bands involve an N-N stretching of the central diazo-bond. And only occur in the E isomer. In fact, in the experimental Raman spectra upon irradiation at 365 nm the band at 1460 cm^{-1} with a small shoulder at 1475 cm^{-1} undergo a strong decrease in intensity allowing to use this band as a fingerprint for the *E*-isomer.

Due to the nature of the isomerization infrared spectroscopy is less sensitive to track changes in the molecular structure compared to Raman spectroscopy. However, from the DFT simulation the band at 1604 cm^{-1} is prominent for the N-N stretch vibration for the (*Z*)-isomer and not present in the Z-isomer. Although no pronounced changes in the FTIR spectra could be observed that would allow to definitely describe the presence of the Z-isomer, the studies of the ligand by FTIR are in particular useful for the investigation of the MOF material at a later stage.

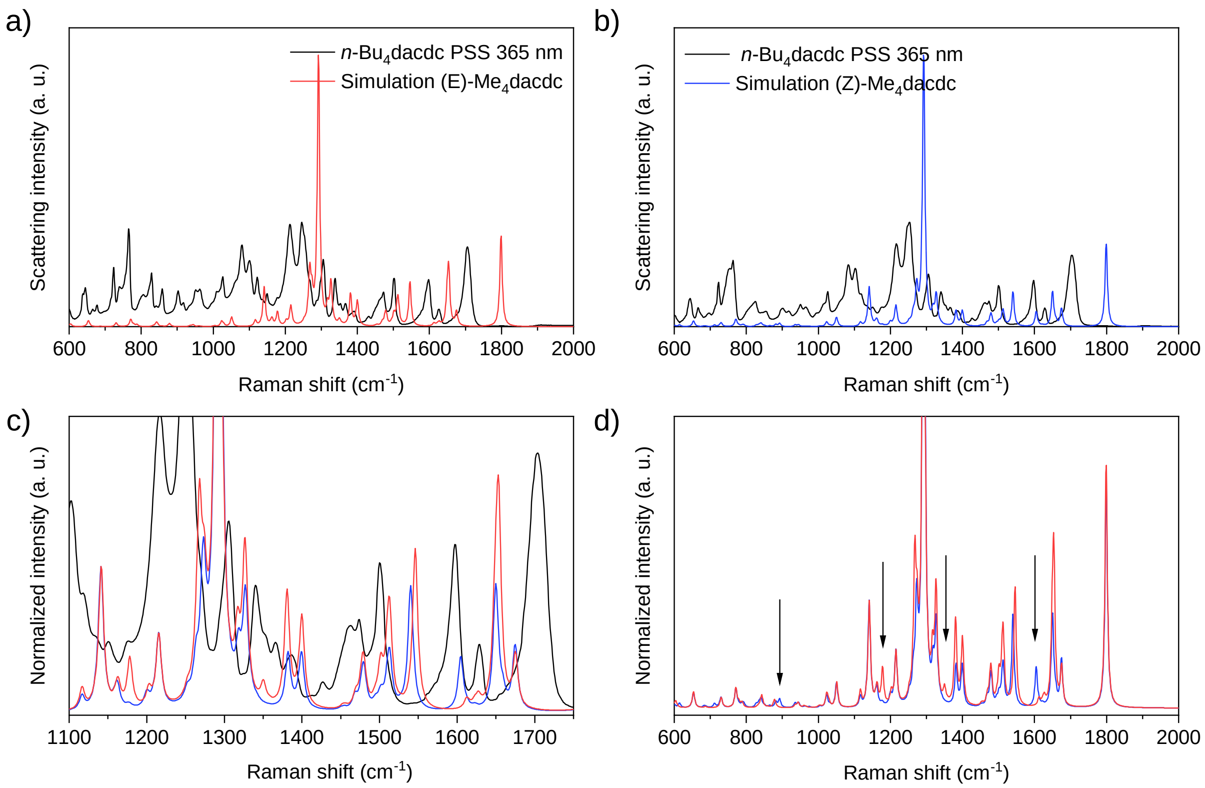

Supplementary Figure 103. Arrows indicate changes in spectrum

21) Computational analysis of the charge transfer in Cu_2dacdc

In order to compute the possibility of a charge transfer between the HOMO and the LUMO of the chromophore in DUT-163, we approximated the photoswitchable core to a triacid with only a copper dimer attached to one of the acid sites. All the calculations were performed using ORCA 4.1.2[32,33]. The starting geometry was obtained from the X-Ray diffraction optimized structure. We removed three copper dimers and substituted those cores with a proton. The fourth copper dimer was capped with formates. All the oxygens and the copper cluster were fixed while the other atoms were optimized at the B97-3c level. Spin-flip DFT was computed at the PBE0/def2-TZVP level.

Supplementary Table 13. Cartesian coordinates in Å for $(HCOO)_3Cu_2$dacdc

C	24.163200000	10.841300000	10.151300000
C	24.108200000	9.892900000	9.152300000
C	25.019100000	7.090600000	4.547500000
C	24.973300000	7.012700000	5.938100000
C	24.985000000	8.224100000	6.675400000
C	25.005200000	9.464100000	6.049900000
C	25.010200000	9.508500000	4.666400000
C	25.034200000	8.338200000	3.895600000
C	25.166600000	8.351100000	2.394300000
C	24.995100000	17.389300000	19.759400000
C	24.968000000	17.426300000	18.367700000
C	24.956800000	16.200400000	17.656600000
C	24.998300000	14.976900000	18.310000000
C	25.067900000	14.974300000	19.692700000
C	25.053100000	16.163400000	20.433800000
C	24.952600000	22.142500000	16.310400000
C	24.713200000	2.278500000	7.925800000
C	24.845000000	20.624300000	16.229700000
C	24.893300000	3.770400000	8.051400000
C	24.723700000	19.954300000	15.004800000

C	24.982400000	4.425400000	9.287600000
C	24.718500000	18.571800000	14.927300000
C	25.030700000	5.806500000	9.374300000
C	24.793500000	17.847100000	16.108300000
C	25.027100000	6.547500000	8.199400000
C	24.887300000	18.486400000	17.369900000
C	24.974400000	5.928500000	6.924200000
C	24.934000000	19.877800000	17.411100000
C	24.886900000	4.538600000	6.871700000
C	24.781600000	15.493800000	15.271400000
C	25.075100000	8.886900000	9.076600000
C	23.748600000	15.521300000	14.337900000
C	23.696000000	14.567900000	13.339000000
C	24.659500000	13.560100000	13.259300000
C	25.184900000	10.797700000	11.108400000
N	24.499600000	12.644700000	12.204600000
C	25.696200000	13.536700000	14.201200000
N	25.343200000	11.708200000	12.169500000
C	26.138000000	9.779900000	11.035500000
C	26.091100000	8.836400000	10.026300000
C	25.757100000	14.495200000	15.189600000
H	23.415300000	11.616800000	10.217900000
H	23.305400000	9.908400000	8.428700000
H	25.056500000	6.192800000	3.948000000
H	25.034500000	10.377600000	6.624700000
H	25.029400000	10.467800000	4.171600000
H	24.960100000	18.300300000	20.339300000
H	24.962600000	14.047100000	17.762400000
H	25.104800000	14.026500000	20.210000000
H	24.661900000	20.526600000	14.090400000
H	24.980800000	3.842100000	10.196100000
H	24.671500000	18.077600000	13.968500000
H	25.053400000	6.292100000	10.338500000
H	25.050900000	20.401000000	18.349100000
H	24.800800000	4.029400000	5.923000000
H	22.980600000	16.277000000	14.414400000
H	22.895800000	14.572200000	12.612200000
H	26.926100000	9.760000000	11.775000000
H	26.850600000	8.071400000	9.955300000
H	26.453100000	12.770400000	14.128600000
H	26.574300000	14.497600000	15.896700000
N	24.845600000	16.465200000	16.291800000
N	25.023200000	7.931200000	8.036900000
O	25.577100000	7.321200000	1.870700000
O	25.472100000	22.588200000	17.327400000
O	24.255100000	1.868000000	6.864600000
O	25.185300000	1.625500000	9.107200000
H	25.025000000	0.676400000	8.979400000
O	24.709000000	9.601800000	1.873400000
H	24.835400000	9.562300000	0.911500000
O	24.445400000	22.681300000	15.292600000
H	24.538300000	23.649400000	15.343600000
Cu	25.066700000	14.841300000	24.585400000
Cu	24.914700000	17.465300000	24.598000000
O	25.101200000	15.050400000	22.643900000
O	24.972100000	17.279700000	22.657600000
C	25.050900000	16.160000000	22.070300000
O	24.788400000	17.261800000	26.504400000
O	24.900800000	15.031600000	26.509600000
C	24.848300000	16.157700000	27.087500000
H	24.802900000	16.145900000	28.060100000

O	22.866500000	17.166400000	24.519400000
O	22.998300000	14.937200000	24.521900000
C	22.354500000	16.027200000	24.571000000
H	21.383800000	15.958000000	24.602500000
O	27.344500000	15.280900000	24.626300000
O	27.140500000	17.503900000	24.570600000
C	27.820400000	16.436800000	24.632200000
H	28.788900000	16.537500000	24.635200000

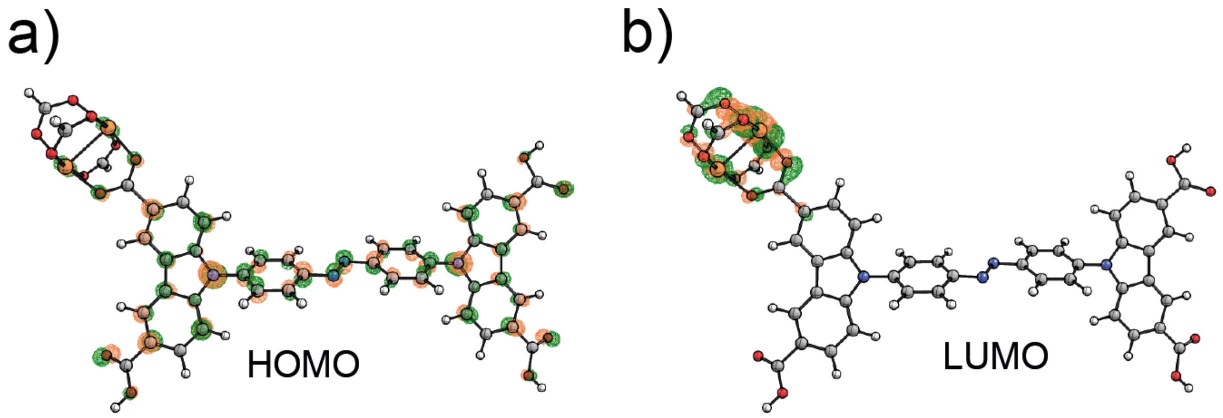

Supplementary Figure 104 a) Higher occupied alpha molecular orbital of Cu_2dacdc (E=-0.2291 eV) and b) Lower unoccupied alpha molecular orbital of Cu_2dacdc (E=-0.1519 eV), calculated at the PBE0/def2-TZVP//B97-3c level.

22) TD-DFT analysis of the excited state of H_4dacdc

In order to compute the buckling motion at the excited state we picked 20 geometries of the dacdc chromophore obtained from the MD run describing the buckling geometries of DUT-163 at the ground state. The carboxylates were functionalized with protons to obtain the tetraacid H_4dacdc and the geometries were reoptimized at the B97-3c level, fixing the carboxylate oxygens to preserve the distortion derived from the buckling. Both CNN angles were further fixed in order to scan different elongations-contractions of the central azo bond, performing a redundant optimization scan of 5° from 100 to 155 degrees. TD-DFT calculations were performed following the Tamm-Dancoff approximation on 10 different states at the PBE0/def2-TZVP level. All these calculations were performed via the ORCA 4.1.2 suite.[32,33] All the coordinates are given as a separated compressed file as additional supplementary information.

23) Supplementary references

1 Krause, S. *et al.* Towards general network architecture design criteria for negative gas adsorption transitions in ultraporous frameworks. *Nat. Commun.* **10**, 3632, doi:10.1038/s41467-019-11565-3 (2019).

2 Barman, D. C., Saikia, P., Prajapati, D. & Sandhu, J. S. HETEROGENEOUS PERMANGANATE OXIDATIONS. A NOVEL METHOD FOR THE DEAMINATION USING SOLID SUPPORTED IRON-PERMANGANATE. *Synthetic Communications* **32**, 3407-3412, doi:10.1081/SCC-120014768 (2002).

3 Weseliński, Ł. J., Luebke, R. & Eddaoudi, M. A Convenient Preparation of 9H-Carbazole-3,6-dicarbonitrile and 9H-Carbazole-3,6-dicarboxylic Acid. *Synthesis* **46**, 596-599, doi:10.1055/s-0033-1340557 (2014).

4 Krause, S. *et al.* The impact of crystal size and temperature on the adsorption-induced flexibility of the Zr-based metal-organic framework DUT-98. *Beilstein J. Nanotechnol.* **10**, 1737–1744, doi:10.3762/bxiv.2019.52.v1 (2019).

5 Mueller, U. *et al.* The macromolecular crystallography beamlines at BESSY II of the Helmholtz-Zentrum Berlin: Current status and perspectives. *EPJ Plus* **130**, 141, doi:10.1140/epjp/i2015-15141-2 (2015).

6 Battye, T. G. G., Kontogiannis, L., Johnson, O., Powell, H. R. & Leslie, A. G. W. iMOSFLM: a new graphical interface for diffraction-image processing with MOSFLM. *Acta Crystallogr. D* **67**, 271-281, doi:10.1107/S0907444910048675 (2011).

7 Sparta, K. M., Krug, M., Heinemann, U., Mueller, U. & Weiss, M. S. XDSAPP2.0. *J. Appl. Crystallogr.* **49**, 1085-1092, doi:10.1107/S1600576716004416 (2016).

8 Sheldrick, G. Crystal structure refinement with SHELXL. *Acta Crystallogr. C* **71**, 3-8, doi:10.1107/S2053229614024218 (2015).

9 Spek, A. PLATON SQUEEZE: a tool for the calculation of the disordered solvent contribution to the calculated structure factors. *Acta Crystallogr. C* **71**, 9-18, doi:doi:10.1107/S2053229614024929 (2015).

10 Bon, V. *et al.* Massive pressure amplification by stimulated contraction of mesoporous frameworks. *Angew. Chem. Int. Ed.* **n/a**, doi:https://doi.org/10.1002/anie.202100549.

11 Krause, S. *et al.* A pressure-amplifying framework material with negative gas adsorption transitions. *Nature* **532**, 348-352, doi:10.1038/nature17430 (2016).

12 Krause, S. *et al.* The Role of Temperature and Adsorbate on Negative Gas Adsorption in the Mesoporous Metal-Organic Framework DUT-49. *Faraday Discuss.*, doi:10.1039/D0FD00013B (2020).

13 Krause, S. *et al.* The effect of crystallite size on pressure amplification in switchable porous solids. *Nat. Commun.* **9**, 1573, doi:10.1038/s41467-018-03979-2 (2018).

14 Krause, S. *et al.* Engineering micromechanics of soft porous crystals for negative gas adsorption. *Chem. Sci.* **11**, 9468-9479, doi:10.1039/D0SC03727C (2020).

15 Helmholtz-Zentrum Berlin für Materialien und Energie. KMC-2: an X-ray beamline with dedicated diffraction and XAS endstations at BESSY II. *JLSRF* **2**, A49, doi:10.17815/jlsrf-2-65 (2016).

16 Bon, V. *et al.* In situ monitoring of structural changes during the adsorption on flexible porous coordination polymers by X-ray powder diffraction: Instrumentation and experimental results. *Microporous Mesoporous Mater.* **188**, 190-195, doi:10.1016/j.micromeso.2013.12.024 (2014).

17 Heiney, P. A. Datasqueeze 2.2.9 Graphical Tool for X-ray Data Analysis. *Datasqueeze 2.2.9 Graphical Tool for X-ray Data Analysis* (2012).

18 Wojdyr, M. Fityk: a general-purpose peak fitting program. *J. Appl. Crystallogr.* **43**, 1126-1128, doi:10.1107/S0021889810030499 (2010).

19 Sung Cho, H. *et al.* Extra adsorption and adsorbate superlattice formation in metal-organic frameworks. *Nature* **527**, 503, doi:10.1038/nature15734

20 Borchardt, L. *et al.* Illuminating solid gas storage in confined spaces – methane hydrate formation in porous model carbons. *Phys. Chem. Chem. Phys.* **18**, 20607-20614, doi:10.1039/C6CP03993F (2016).

21 Willems, T. F., Rycroft, C. H., Kazi, M., Meza, J. C. & Haranczyk, M. Algorithms and tools for high-throughput geometry-based analysis of crystalline porous materials. *Microporous Mesoporous Mater.* **149**, 134-141, doi:10.1016/j.micromeso.2011.08.020 (2012).
22 Dubbeldam, D., Calero, S., Ellis, D. E. & Snurr, R. Q. RASPA: molecular simulation software for adsorption and diffusion in flexible nanoporous materials. *Molecular Simulation* **42**, 81-101, doi:10.1080/08927022.2015.1010082 (2016).
23 Rappe, A. K., Casewit, C. J., Colwell, K. S., Goddard, W. A. & Skiff, W. M. UFF, a full periodic table force field for molecular mechanics and molecular dynamics simulations. *J. Am. Chem. Soc.* **114**, 10024-10035, doi:10.1021/ja00051a040 (1992).
24 Jorgensen, W. L., Madura, J. D. & Swenson, C. J. Optimized intermolecular potential functions for liquid hydrocarbons. *J. Am. Chem. Soc.* **106**, 6638-6646, doi:10.1021/ja00334a030 (1984).
25 Coudert, F.-X., Jeffroy, M., Fuchs, A. H., Boutin, A. & Mellot-Draznieks, C. Thermodynamics of Guest-Induced Structural Transitions in Hybrid Organic−Inorganic Frameworks. *J. Am. Chem. Soc.* **130**, 14294-14302, doi:10.1021/ja805129c (2008).
26 Evans, J. D., Bocquet, L. & Coudert, F.-X. Origins of Negative Gas Adsorption. *Chem* **1**, 873-886, doi:10.1016/j.chempr.2016.11.004 (2016).
27 Bureekaew, S. *et al.* MOF-FF – A flexible first-principles derived force field for metal-organic frameworks. *Phys. Status Solidi B* **250**, 1128-1141, doi:10.1002/pssb.201248460 (2013).
28 Allinger, N. L., Yuh, Y. H. & Lii, J. H. Molecular mechanics. The MM3 force field for hydrocarbons. 1. *J. Am. Chem. Soc.* **111**, 8551-8566, doi:10.1021/ja00205a001 (1989).
29 Kolodzeiski, E. & Amirjalayer, S. Atomistic Insight Into the Host–Guest Interaction of a Photoresponsive Metal–Organic Framework. *Chem. Eur. J.* **26**, 1263-1268, doi:10.1002/chem.201905139 (2020).
30 Plimpton, S. Fast Parallel Algorithms for Short-Range Molecular Dynamics. *J. Comput. Phys.* **117**, 1-19, doi:https://doi.org/10.1006/jcph.1995.1039 (1995).
31 Rogge, S. M. J., Waroquier, M. & Van Speybroeck, V. Reliably Modeling the Mechanical Stability of Rigid and Flexible Metal–Organic Frameworks. *Acc. Chem. Res.* **51**, 138-148, doi:10.1021/acs.accounts.7b00404 (2018).
32 Neese, F. Software update: the ORCA program system, version 4.0. *WIREs Computational Molecular Science* **8**, e1327, doi:10.1002/wcms.1327 (2018).
33 Neese, F. The ORCA program system. *WIREs Computational Molecular Science* **2**, 73-78, doi:10.1002/wcms.81 (2012).
34 Grimme, S., Antony, J., Ehrlich, S. & Krieg, H. A consistent and accurate ab initio parametrization of density functional dispersion correction (DFT-D) for the 94 elements H-Pu. *The Journal of Chemical Physics* **132**, 154104, doi:10.1063/1.3382344 (2010).
35 Grimme, S., Ehrlich, S. & Goerigk, L. Effect of the damping function in dispersion corrected density functional theory. *J. Comput. Chem.* **32**, 1456-1465, doi:https://doi.org/10.1002/jcc.21759 (2011).
36 Gaussian 16, Revision B.01 (Gaussian, Inc., , Wallingford CT, 2016).
