# Supplementary material for: Cooperative light-induced breathing of soft porous crystals via azobenzene buckling
Source: Nat Commun. 2022 Apr 12;13:1951. doi: 10.1038/s41467-022-29149-z (PMC9005654; doi:10.1038/s41467-022-29149-z)
Supplement: Supplementary file 2 — Description of Additional Supplementary Files [file 41467_2022_29149_MOESM2_ESM.pdf]

Description of Additional Supplementary Files

Supplementary Movie 1: Buckling of Azobenzene

Supplementary Movie 2: Contraction of DUT-163 via Buckling

Supplementary Movie 3: *E-Z* isomerization of Azobenzene

Supplementary Movie 4: Contraction of DUT-163 via *E-Z* isomerization
